# Transcriptome and Expression Patterns of Chemosensory Genes in Antennae of the Parasitoid Wasp *Chouioia cunea*

**DOI:** 10.1371/journal.pone.0148159

**Published:** 2016-02-03

**Authors:** Yanni Zhao, Fengzhu Wang, Xinyue Zhang, Suhua Zhang, Shilong Guo, Gengping Zhu, Qiang Liu, Min Li

**Affiliations:** 1 Tianjin Key Laboratory of Animal and Plant Resistance, Tianjin Normal University, 300387, Tianjin, China; 2 Natural Enemy Breeding Center of Luohe Central South Forestry, 462000, Henan, China; USDA-ARS, UNITED STATES

## Abstract

*Chouioia cunea* Yang is an endoparasitic wasp that attacks pupae of *Hyphantria cunea* (Drury), an invasive moth species that severely damages forests in China. Chemosensory systems of insects are used to detect volatile chemical odors such as female sex pheromones and host plant volatiles. The antennae of parasite wasps are important for host detection and other sensory-mediated behaviors. We identified and documented differential expression profiles of chemoreception genes in *C*. *cunea* antennae. A total of 25 OBPs, 80 ORs, 10 IRs, 11 CSP, 1 SNMPs, and 17 GRs were annotated from adult male and female *C*. *cunea* antennal transcriptomes. The expression profiles of 25 OBPs, 16 ORs, and 17 GRs, 5 CSP, 5 IRs and 1 SNMP were determined by RT-PCR and RT-qPCR for the antenna, head, thorax, and abdomen of male and female *C*. *cunea*. A total of 8 OBPs, 14 ORs, and 8 GRs, 1 CSP, 4 IRs and 1 SNMPs were exclusively or primarily expressed in female antennae. These female antennal-specific or dominant expression profiles may assist in locating suitable host and oviposition sites. These genes will provide useful targets for advanced study of their biological functions.

## Introduction

Insects use chemoreception when searching for food, oviposition sites, and mates, as well as for social communication [1]. Chemoreception refers to the classical senses of smell (olfaction, the detection of volatile chemical stimuli) and taste (gustation, or ‘contact chemoreception’ for the detection of dissolved or solid chemicals) [2, 3]. It is often stated that “insects live in a chemical world” with chemical messages from host plant volatiles, pheromones, and predator odors captured by chemoreceptors and translated into physiological signals (chemical and electric) that modify behavior [4]. Chemoreceptors are mainly located on antennae, maxillary palps, and labial palps. Antennae are the principal location of insect olfactory receptors [5].

During the last 30 years, knowledge of the molecular and cellular basis of insect chemoreception has greatly expanded. Several multi-gene families encode proteins with crucial roles in chemoreception systems. This list includes both receptor and non-receptor proteins. The receptors located mainly on the antennae, and to a lesser extent on other sensory appendages, are encoded by three large gene families [6]: odorant receptors (ORs), gustatory receptors (GRs), and ionotropic receptors (IRs) [7–9]. The ORs and GRs belong to the same receptor superfamily [10]. The ORs detect volatile chemicals (odors) while the GRs are responsible for contact chemoreception and detection of carbon dioxide [6, 9]. IRs belong to an ancient family of chemosensory receptors that are relatives of ionotropic glutamate receptors (iGluR), and are divided into two subfamilies, the conserved “antennal” IRs and the species-specific “divergent” IRs [11, 12]. The IRs have been identified in several insect species from different orders [13–17]. However, IRs have only been functionally characterized in *Drosophila*. Benton et al. [7] demonstrated that several *Drosophila* IRs are expressed at the ciliated endings of some antennal receptor neurons. The ectopic expression of some IRs in specific sensillae triggers novel odor-evoked responses, suggesting a functional connection between IRs and odor perception [7].

The non-receptor proteins are encoded by three gene families, the odorant binding proteins (OBPs), chemosensory proteins (CSPs) and sensory neuron membrane proteins (SNMP). The OBPs are small (10 to 30 kDa) soluble proteins that are highly abundant in the sensillum hemolymph [18–19]. OBPs bind odor molecules, most of which are hydrophobic, and transport them through the hydrophilic environment in the sensillum to the membrane-bound receptor. Additionally, OBPs may filter or purify odorants, act as activator factors of ORs (after conformational change), or as carriers expressed in non-olfactory tissues. Insect CSPs, which are also called OS-D like proteins [20] or sensory appendage proteins (SAPs) [21], represent a novel group of olfactory proteins involved in insect olfaction. CSPs have shown broad expression profiles in chemosensory tissues, including antennae [22–27], maxillary palps [28], labial palps [28–29], and the proboscis [30]. However, these proteins are also found in non-chemosensory organs, such as legs [31, 32], wings [33, 34], and pheromone glands [25]. Insect CSPs have multiple functions in insect chemoreception, growth and development. In different species, variable expression of the genes occurs depending on, for instance, sex, tissue, or life stage [24, 35]. The sensory neuron membrane proteins (SNMP) are expressed in pheromone-responding neurons in *Drosophila* and Lepidoptera and are, in some cases, important for proper pheromone responses [36–38].

Transcriptomes have been used to identify chemosensory genes based on next-generation sequencing data in species for which complete genomic sequence is unavailable. High-throughput transcriptomic approaches are more efficient for large-scale gene discovery, compared to conventional homology-cloning methods, and are especially useful for the identification of highly diverse gene families [39]. Transcriptomes assembled from high-throughput sequencing data have been used to identify protein families involved in chemoreception in many pest insects, such as *Anopheles gambiae* [40], *Manduca sexta* [41], *Cydia pomonella* [13], *Helicoverpa armigera* [42], *Agrilus planipennis* [43], *Aphis gossypii* [44], *Spodoptera littoralis* [45], *Ips typographus* and *Dendroctonus ponderosae* [46]. However, identification of chemosensory proteins among natural insect enemies/predators (e.g. parasitoids) is much more limited. Up to now, only a few species, *Nasonia vitripennis* [47], *Cotesia vestalis* [48] and *Microplitis mediator* [49] have been studied.

Parasitic wasps (parasitoids) serve as important natural agents that play an important role in the biological control of insect pests [50]. The success of parasitic wasps in suppressing pest populations depends on their ability to locate hosts in a complex chemical environment [50, 51]. Like most insects, parasitic wasps locate their hosts by foraging and reproduction occurs through a series of behavioral activities, regulated mainly by chemoreception [52–54]. The identification of chemosensory genes in parasitoid wasps is crucial, both to address the mechanisms controlling intraspecific or interspecific chemical communication and for potential genetic manipulation of parasitoid behavioral responses via modification of their ability to discriminate the chemical cues used in biological control strategies [49].

The parasitoid wasp *Chouioia cunea* Yang (Hymenoptera: Eulophidae), is an endoparasitic chalcid wasp, native to China, that parasitizes the fall webworm, *Hyphantria cunea* Drury, [55]. *H*. *cunea* is a worldwide pest that has invaded China that utilizes > 175 different plant species in 49 families and 108 genera as acceptable hosts [56, 57]. *C*. *cunea* also parasitizes other Lepidoptera defoliators, including *Clostera anachoreta* F., *Micromilalopha troglodyta* (Graeser) (Notodontidae), *Stilpnotia salicis* (L.), *S*. *candida* Staudinger, *Ivela ochropoda* Fabricius (Lymantriidae) and *Clania variegeta* Snelleny (Psychidae) [55].

*C*, *cunea* are small, with adults on the scale of 1.1–1.5 mm long. As many as 365 adult wasps can be reared from a single *H*. *cunea* pupa with a high percentage (98–99%) of females [58]. In China, *C*. *cunea* has shown great promise for reducing *H*. *cunea* populations [56, 58, 59–61]. While previous research with *C*. *cunea* has focused primarily on ecology, behavior and anatomy [61], there is no information regarding its chemosensory abilities. This study investigated the antennal chemosensory gene families expressed in *C*. *cunea* via transcriptomic analysis using a next-generation sequencing (NGS) 454 GS FLX platform. Identification of members of the primary chemosensory families (including OBPs, GRs, ORs, IRs, CSPs and SNMPs) from female and male *C*. *cunea* antennae will permit a better understanding of the molecular basis of *C*. *cunea* chemoreception. Using RT-PCR and real-time quantitative-PCR (RT-qPCR), we screened many antennae-specific or enriched chemosensory genes from the assembled antennal transcriptomes that may have important functions in *C*. *cunea* chemoreception. This information could lead to the identification of targets for novel control strategies and an improved understanding of how *C*. *cunea* recognizes, locates and parasitizes hosts.

## Materials and Methods

### Ethics statement

*Antheraea pernyi* and *C*.*cunea* are common insects and are not included in the ‘‘List of Endangered and Protected Animals in China”. All operations were performed according to ethical guidelines in order to minimize pain and discomfort to the insects.

### Insect rearing and tissue collection

*C*. *cunea* were obtained in 2012 from the Natural Enemy Breeding Center of Luohe Central South Forestry (Henan, China). The tussah, *Antheraea pernyi* Guerin-Meneville (Lepidoptera: Saturniidae), an alternate host of *C*. *cunea*, was obtained from the Benxi Tussah Breeding Base (Liaoning, China). To obtain several generations of *C*. *cunea*, adult wasps were placed with *A*. *pernyi* pupae in erlenmeyer flasks and the openings sealed with cotton wool. They were maintained at 25°C, 75% RH, and a 14:10 light: dark cycle and incubated until the adults emerged (17–20 days). Emerged adult wasps were collected within 24 h. Parts of *C*. *cunea* (antenna, heads without antennae, thoraxes, and abdomens) were excised from 1-day-old male and female wasps, immersed in RNA Later (Ambion, AM7020) and collected in Eppendorf tubes. The tubes contained either 50 antennae, 50 heads, 50 thoraxes, or 50 abdomens and each tube constituted a unit sample. A total of 1500 females and 1500 males were sampled. All tubes were stored at -20°C until processing.

### RNA preparation and cDNA library construction

A total of 500 antennae from each sex were pooled for total RNA extraction using TRIzol reagent (Invitrogen, Carlsbad, CA, USA) according to themanufacturer’s instructions. RNA was quantified using a NanoDrop spectrophotometer (Thermo Scientific, Wilmington, DE, USA) and quality checked using electrophoresis through a 1.1% agarose gel. Approximately 500 ng messenger RNA was further purified from 50 μg total RNA using a PolyATtract mRNA Isolation System III (Promega, Madison, WI, USA). The mRNAs were then sheared into approximately 800 nucleotide lengths via RNA Fragmentation Solution (Autolab, Beijing, China) at 70 uC for 30 sec, then cleaned and condensed using an RNeasy MinElute Cleanup Kit (Qiagen, Valencia, CA, USA). The first-strand cDNA was synthesized using N6 random primers and MMLV reverse transcriptase (TaKaRa, Dalian, China). Then, the second strand cDNAs were synthesized using secondary strand cDNA synthesis enzyme mixtures (Autolab, Beijing, China). The cDNAs with the desired length were purified using a QIAquick PCR Purification Kit (Qiagen, Valencia, CA, USA) and eluted with 10 μl elution buffer. After blunting and appending with a poly-A tail at the 3’ end according to Roche Rapid Library Preparing protocols (Roche, USA), the purified cDNAs were linked to GS-FLX Sequencing Adaptors (Roche, USA). Finally, cDNAs shorter than 500 bp were removed using AMPure Beads according to the manufacturer's instructions (Beckman, USA) prior to cDNA library preparation.

### 454 de novo transcriptome assembly and analysis

Pyrosequencing of the cDNA library was performed using a 454 GS-FLX sequencer (Roche, IN, USA) according to the manufacturer’s instructions. All raw reads were processed to remove low quality and adaptor sequences. Cleaned reads shorter than 60 bases were discarded based on the assumption that these reads represent sequencing artifacts [62]. Sequence reads were then clustered and assembled using the Trinity short reads assembling program (version: 2013-08-14)(http://sourceforge.net/projects/trinityrnaseq/files/PREV_CONTENTS/previous releases/) [63, 64] with a minimum sequence overlap of 49 nt and a minimum percentage overlap identity of 80%. The resulting contigs and singletons >100 bp in length were retained as unigenes and annotated as described below. Following assembly, homology searches of all unigenes were performed using the BLASTx and BLASTn programs against the NCBI non-redundant protein (nr) and nucleotide sequence (nt) databases [65]. Matches with an E-value less than 1.0E-5 were considered significant [66]. Gene names were assigned to each unigene based on the best BLASTx hit with the highest score value.

Gene Ontology terms were assigned using Blast2GO [67] through the BLASTx program with an E-value less than 1.0E-5. WEGO software [68] was used to assign each GO ID to the related ontology entries. The longest open reading frame (ORF) for each unigene was determined with the ORF finder tool (http://www.ncbi.nlm.nih.gov/gorf/gorf.html).

### Identification of *C*. *cunea* chemosensory genes

A tBLASTn analysis was performed using available OBP, CSP, OR, GR, IR and SNMP protein sequences from hymenopteran species as ‘‘queries” to identify candidate unigenes in *C*. *cunea*. All candidate OBPs, CSPs, ORs, GRs, IRs, and SNMPs were manually checked using BLASTx program. To compare the differential expression of chemosensory genes in the *C*. *cunea* male and female antennal transcriptomes, the read number for each chemosensory gene between male and female antennae was converted to RPKM (Reads Per Kilobase per Million mapped reads) [69], using the formula: RPKM (A) = (1,000,000×C×1,000)/(N×L), where RPKM (A) is the expression of chemosensory gene A, C is the number of reads that are uniquely mapped to chemosensory gene A, N is the total number of reads that are uniquely mapped to all unigenes, and L is the number of bases in chemosensory gene A. C: the number of reads that are mapped to a known set of chemosensory genes were determined by the software SOAP(v2.21t) http://soap.genomics.org.cn/ [70].

### Sequence and phylogenetic analysis

The putative N-terminal signal peptides and the most likely cleavage sites were predicted using SignalP V3.0 (http://www.cbs.dtu.dk/services/SignalP/). Sequence alignments were performed using ClustalX 2.1 [71] with default gap penalty parameters of gap opening 10 and extension 0.2, and were edited using GeneDoc 2.7.0. A neighbor-joining tree [72] was constructed using MEGA 5.0 [73] with a p-distance model and a pairwise deletion of gaps. The bootstrap support of tree branches was assessed by re-sampling amino acid positions 1000 times.

### RT-PCR and RT-qPCR analysis

Samples of 400 male antennae, 400 female antennae, 200 male heads, 200 female heads, 100 male thoraxes, 100 female thoraxes, 100 male abdomens, and 100 female abdomens were used for RNA extraction using TRIzol reagent. Before transcription, total RNA was treated with RQ1 RNase-Free DNase (Promega, Madison, USA) to remove residual genomic DNA. First-strand cDNA was synthesized using a TransScript RT reagent Kit (TransGen, Beijing, China). An equal amount of cDNA (200 ng) was used as templates for RT-PCR and RT-qPCR. The specific primer pairs used for RT-PCR and RT-qPCR were designed with Primer 5 (http://frodo.wi.mit.edu/). GADPH was used as the control gene to test the integrity of the cDNAs. Primers used for this study are shown in S1 Table. The RT-PCR was performed using the following conditions: 94°C for 30 sec, followed by 35 cycles of 94°C for 30 sec, 60°C for 20 sec, 72°C for 40 sec, and a final extension for 3 min at 72°C. PCR products were analyzed on 1.2% agarose gels and visualized after staining with ethidium bromide. To evaluate reproducibility, amplification of each target gene was performed three times with three biological samples. RT-qPCR analysis was conducted using an ABI 7500 Real-Time PCR System (Applied Biosystems, Carlsbad, CA). The reference gene GADPH was used to normalize target gene expression and for correcting for sample-to-sample variation. Each RT-qPCR reaction was conducted in a 20 μL reaction containing 10 μL of TransStart TopGreen qPCR SuperMix (TransGen, Beijing, China), 0.4 μL of each primer (10μM), 1 μL of sample cDNA, and 8.2 μL of sterilized H_2_O. The RT-qPCR cycling parameters were: 94°C for 30 sec, followed by 40 cycles of 94°C for 5 sec, 55°C for 15 sec and 72°C for 10 sec. The PCR products were heated to 95°C for 15 sec, cooled to 60°C for 1 min, heated to 95°C for 30 sec and cooled to 60°C for 15 sec to measure the dissociation curves. Negative controls without either template or transcriptase were included in each experiment. To check reproducibility, each RT-qPCR reaction for each sample was performed in three technical replicates using three biological replicates. The comparative 2^-△△CT^ method was used to calculate the relative quantification between tissues [74]. Comparative analyses of each target gene among the various tissues were determined using a one-way nested analysis of variance (ANOVA), followed by Tukey’s honestly significant difference (HSD) test using SPSS Statistics 18.0 (SPSS Inc., Chicago, IL, USA). When applicable, values are presented as the mean ± SE. To explore the pairwise relationship of RPKM value and quantitative Real-Time PCR, correlation analyses were calculated using the Pearson test in SPSS Statistics 18.0.

## Results

### 454 sequencing and de novo assembly

**Fig 1:**
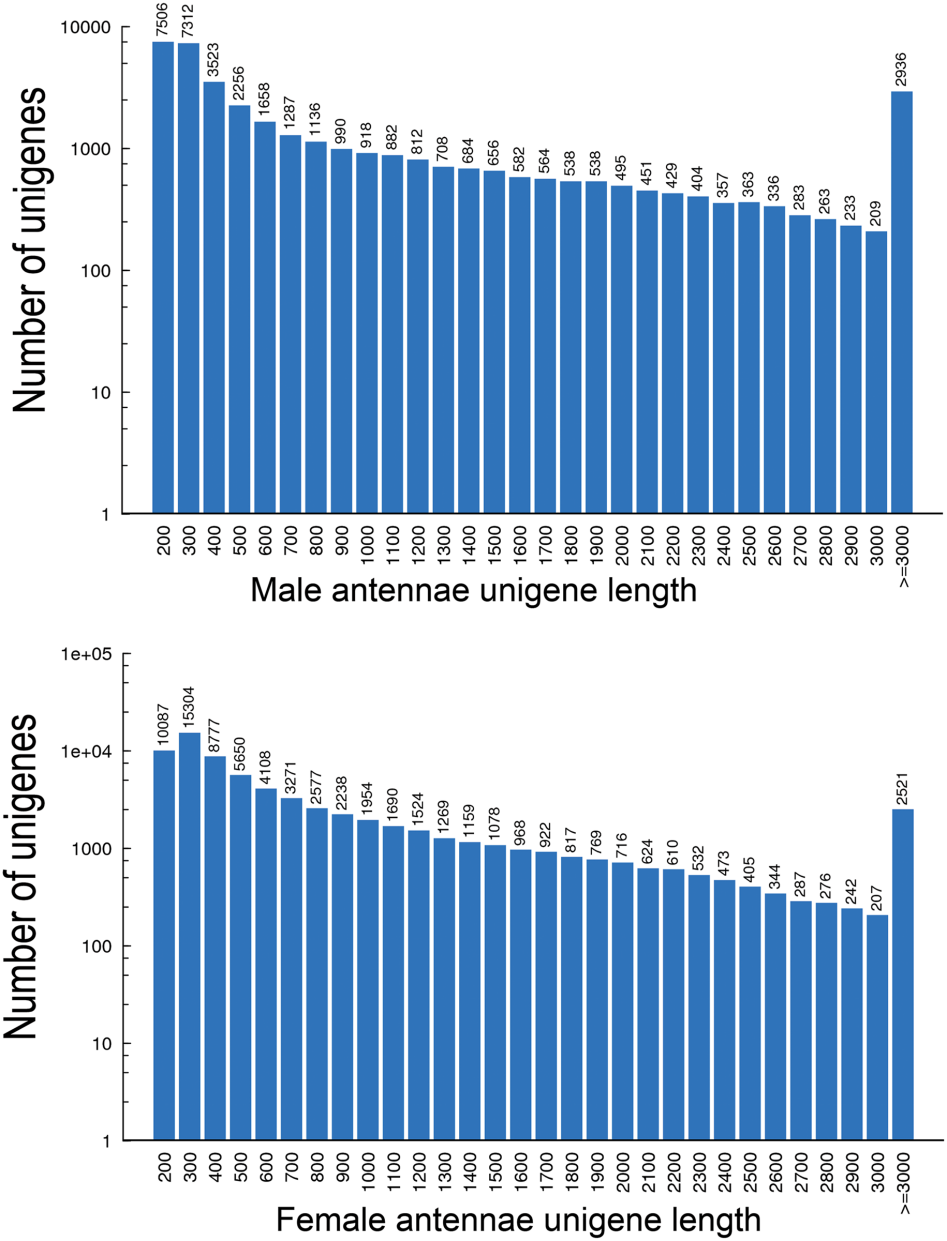
The size distuibution of the assembled unigenes from *C*.*cunea* male and female antennal transcriptomes.

Two non-normalized cDNA libraries for male and female *C*. *cunea* antennae were constructed. Sequencing using a 454 GS FLX platform yielded a total of 87,079,732 and 84,677,350 raw reads for the male and female antennae samples respectively. After eliminating adaptor sequences, contaminating sequences and low quality sequences, 79,802,284 (Total Clean Nucleotides (nt): 7,182,205,560) and 82,208,700 (Total Clean Nucleotides (nt): 7,398,783,000) clean reads were obtained from male and females respectively and used in transcriptome assembly. All clean reads from male and female antennae were assembled to generate 39,309 (mean length 1005 bp) and 71,399 (mean length 794 bp) unigenes. We assembled all clean reads from male and female antennae together and generated 50,061 unigenes (mean length 1116 bp). Among these unigenes, 15,293 were Distinct Clusters (30.5%) and 34,768 were Distinct Singletons (69.5%). The assembled unigene lengths ranged from 200 bp to 17,890 bp. The size distribution of the assembled unigenes is shown in Fig 1.

### Homology search of *C*. *cunea* antennal unigenes with other insect species

**Fig 2:**
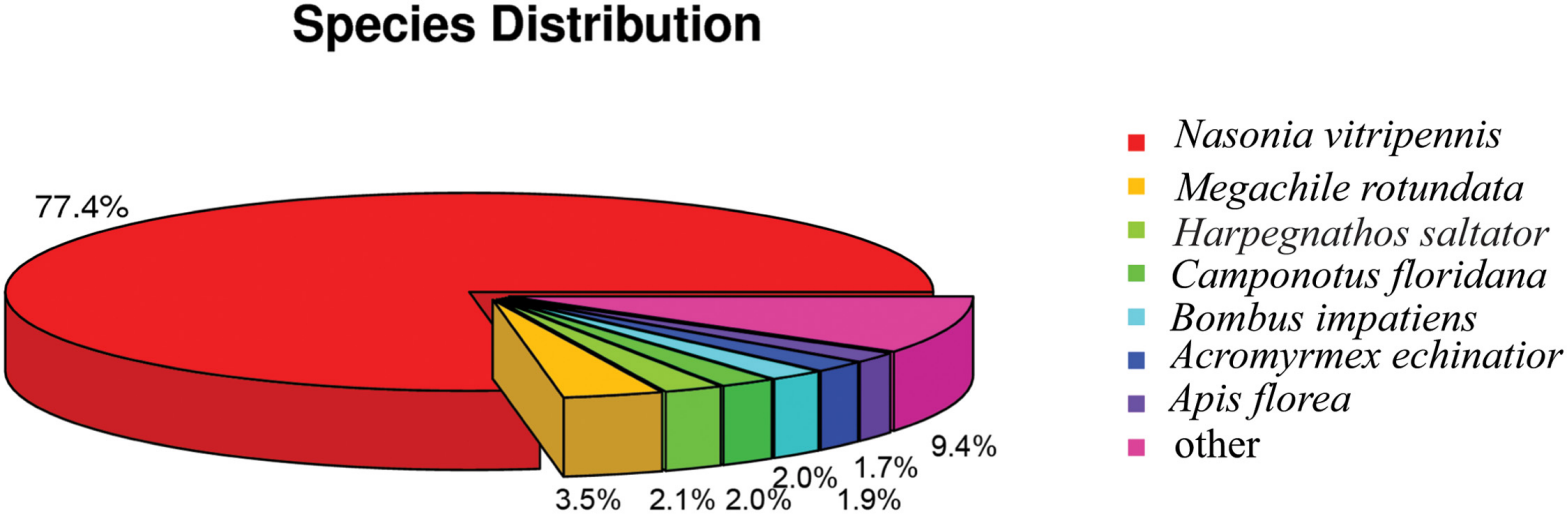
The best hits of the BLASTn results. All *C*. *cunea* antennal unigenes were used in BLASTn to search the GenBank entries. The best hits with an E-value = 1.0E-5 for each query were grouped according to species.

We searched for homologs in other insect species using the BLASTx and BLASTn programs with an E-value cut-off of 1E-5. The results indicated that 25,721 of the 50,061 unigenes (51.4%) had BLASTx hits in the non-redundant (nr) databases and that 21,809 (43.6%) had BLASTn hits in the non-redundant nucleotide sequence (nt) databases. Some unigenes are homologous to genes from other species with best BLASTx hits to hymenopteran insect genes (24,497 of the 25,721 nr-hits), which included 19,916 unigenes most homologous to *N*. *vitripennis*. The second highest hits corresponded to 902 unigenes homologous with *Megachile rotundata* sequences. The other unigenes were homologous to genes from *Harpegnathos saltator* (536 hits), *Camponotus floridanus* (506 hits), *Bombus impatiens* (506 hits), *Acromyrmex echinatior* (482 hits), and *Apis florae* (446 hits) (Fig 2).

### Functional annotation of the *C*.*cunea* antennal unigenes

**Fig 3:**
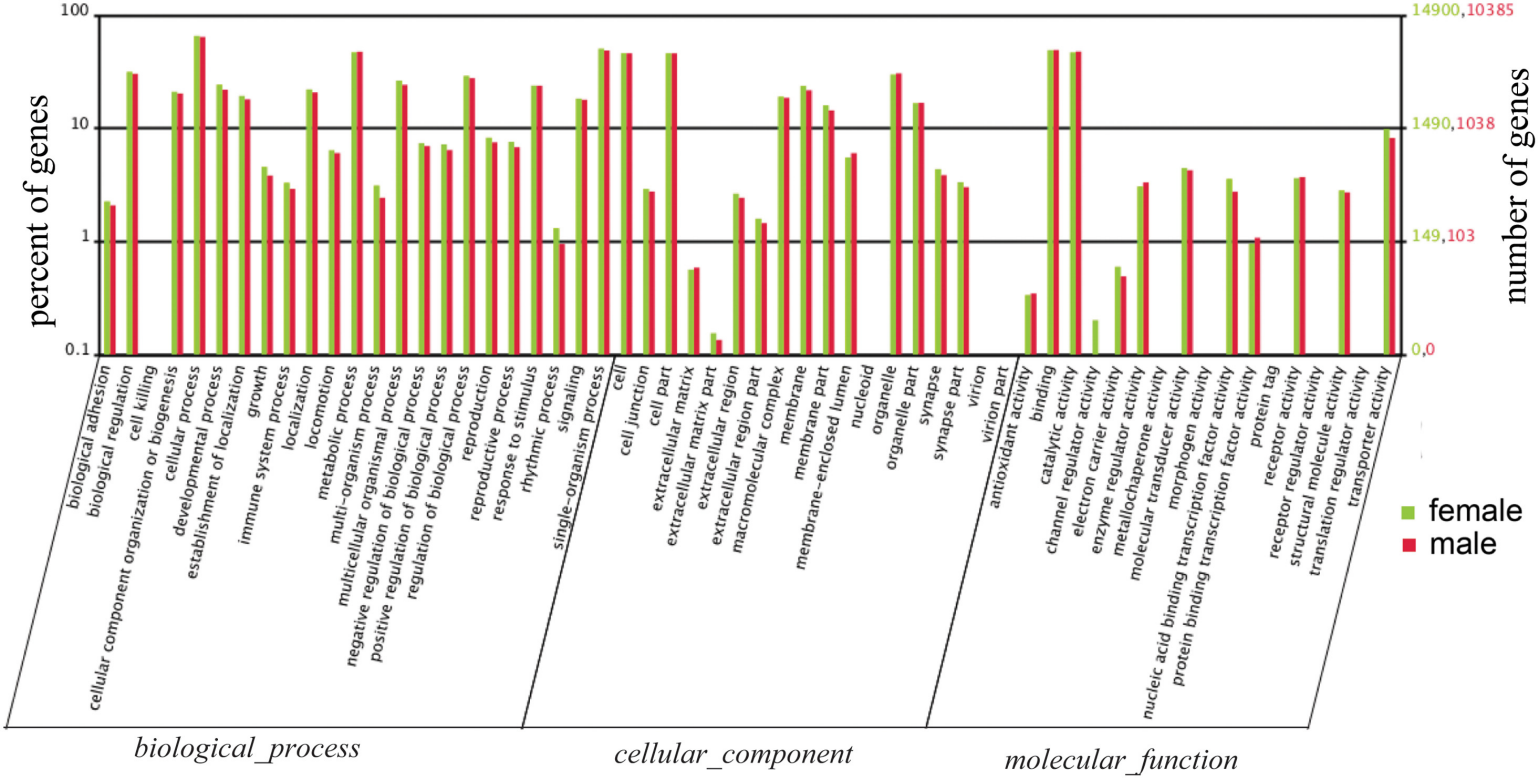
Gene Ontology (GO) classifications of the male and female *C*. *cunea* antennal unigenes according to their involvement in biological processes, cellular component and molecular function.

A total of 10,385 male antennal unigenes and 14,900 female antennal unigenes were annotated with the resulting functional groups classified via GO analyses [75] into one of three groups: biological process, cellular components or molecular functions (Fig 3). The abundance of annotated transcripts for each GO category was similar between the male and female antennal transcriptomes (Fig 3). Among the biological process category, cellular and metabolic processes were the two largest groups in both data sets. The molecular function category was mainly comprised of sequences involved in binding and catalytic activities. Under the cellular component category, cell and cell part were the most abundant GO terms (Fig 3). These GO annotations provide insights into the global gene expression profile for male and female antenna of *C*. *cunea*.

### Candidate odorant binding proteins in the *C*. *cunea* antennae

**Fig 4:**
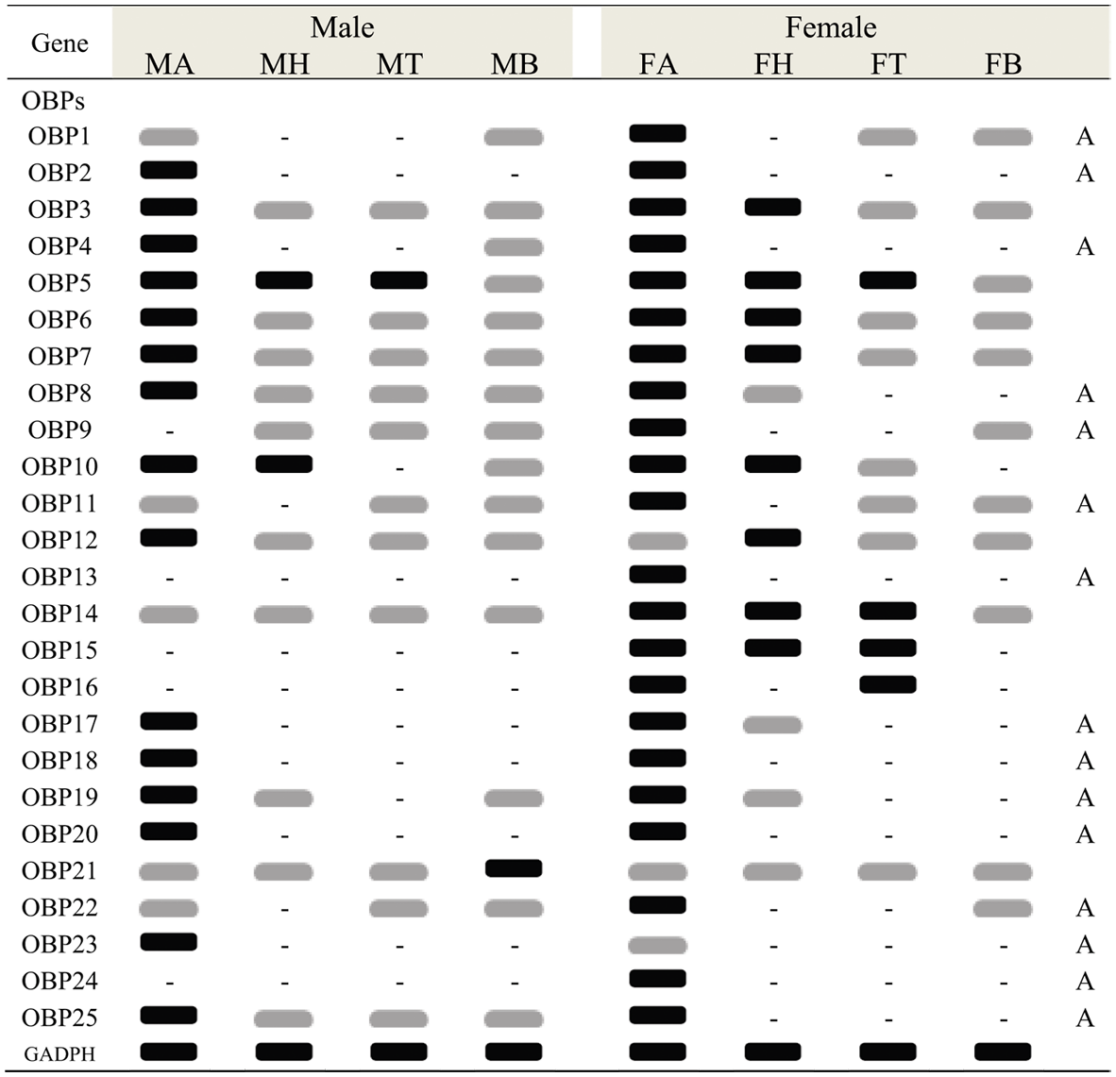
*C*. *cunea* OBPs transcript levels in different body parts as evaluated by RT-PCR. GADPH was used as a control for the integrity of each cDNA template. MA: male antennae; FA: female antennae; MH: male heads; FH: female heads; MT: male thoraxes; FT: female thoraxes; MB: male abdomen; FB: male abdomen. Black graphic indicats robust amplification defined as a clearly detectable amplimer in the agarose gel, gray graphic indicates faint amplication, “-” indicates no amplification. Antennae specific or enriched genes are labeled with a capital letter “A”.

**Fig 5:**
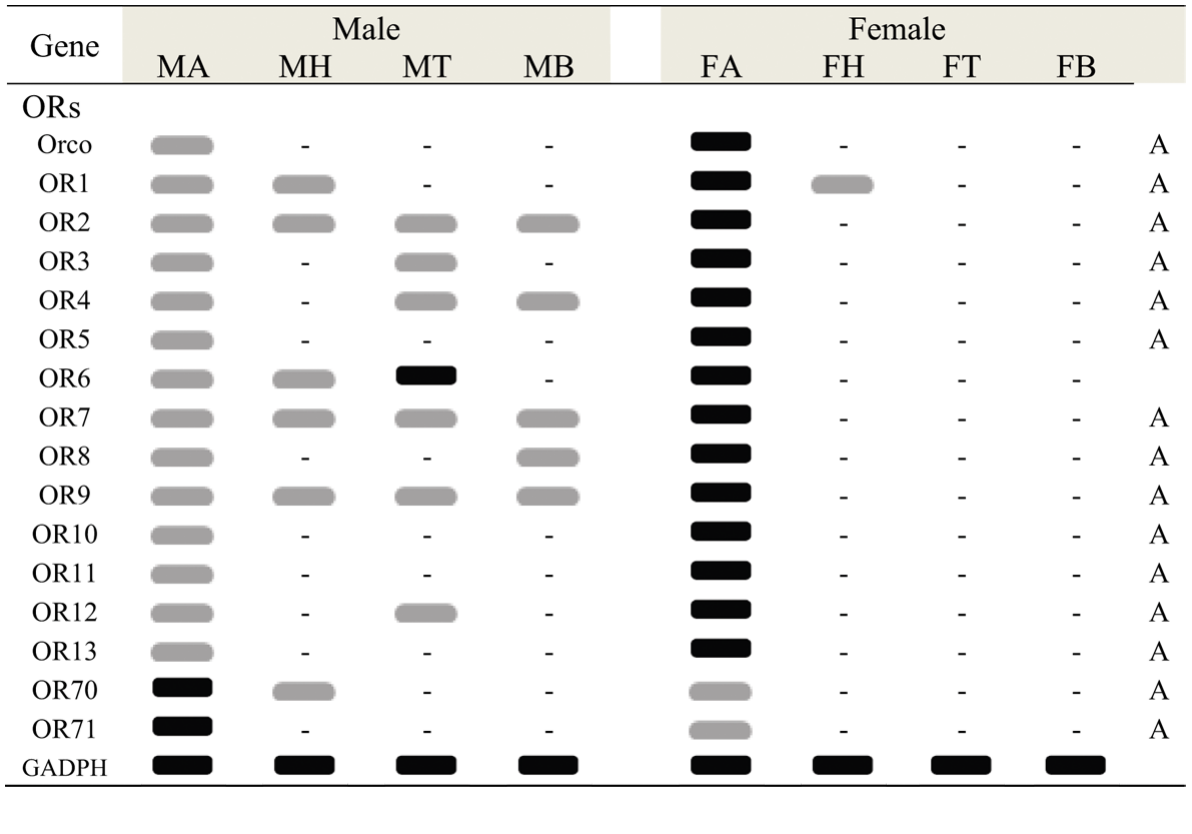
*C*. *cunea* ORs transcript levels in different body parts as evaluated by RT-PCR. GADPH was used as a control for the integrity of each cDNA template. MA: male antennae; FA: female antennae; MH: male heads; FH: female heads; MT: male thoraxes; FT: female thoraxes; MB: male abdomen; FB: male abdomen. Black graphic indicats robust amplification defined as a clearly detectable amplimer in the agarose gel, gray graphic indicates faint amplication, “-” indicates no amplification. Antennae specific or enriched genes are labeled with a capital letter “A”.

**Fig 6:**
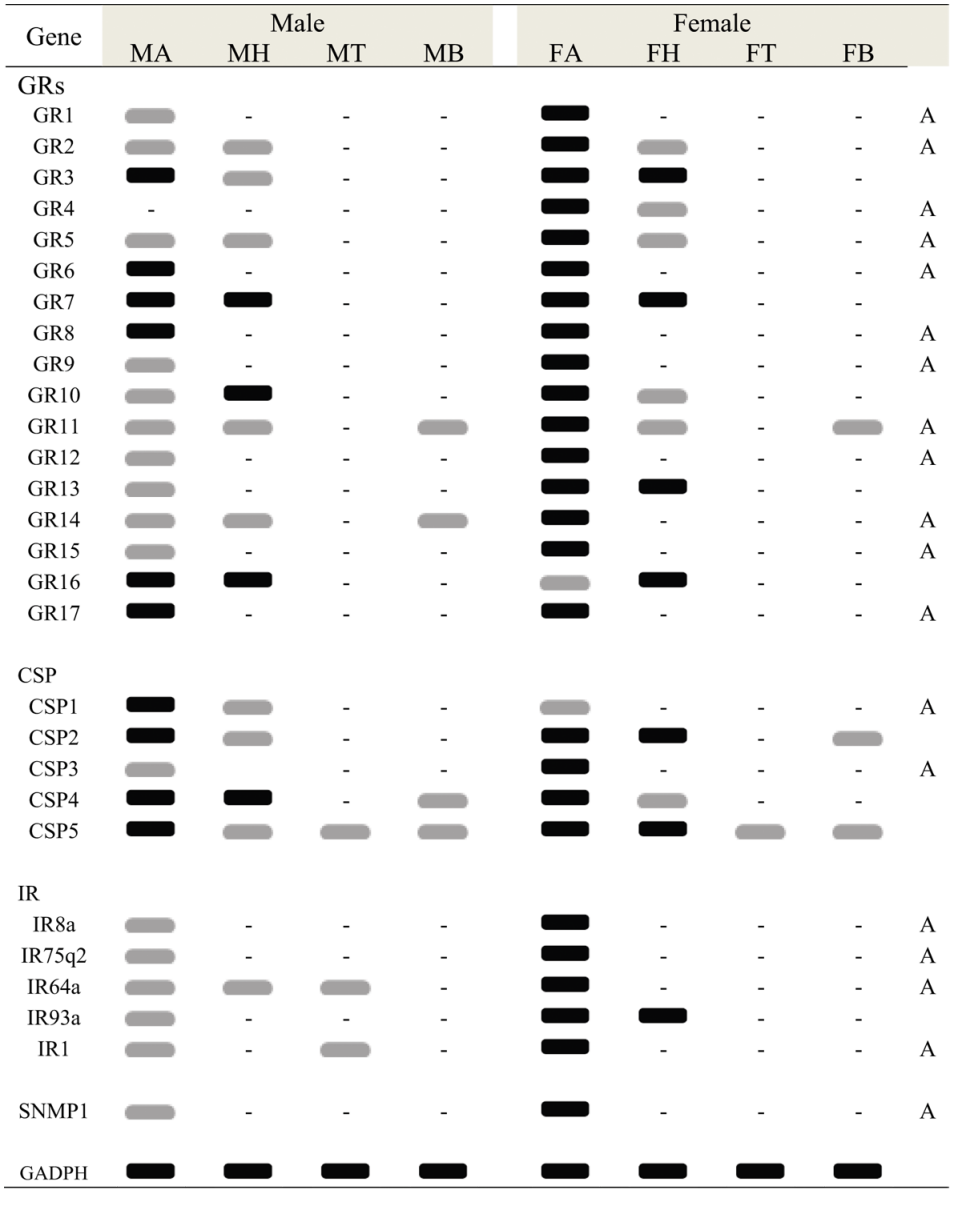
*C*. *cunea* GRs, CSPs, IRs and SNMP1 transcript levels in different body parts as evaluated by RT-PCR. GADPH was used as a control for the integrity of each cDNA template. MA: male antennae; FA: female antennae; MH: male heads; FH: female heads; MT: male thoraxes; FT: female thoraxes; MB: male abdomen; FB: male abdomen. Black graphic indicats robust amplification defined as a clearly detectable amplimer in the agarose gel, gray graphic indicates faint amplication, “-” indicates no amplification. Antennae specific or enriched genes are labeled with a capital letter “A”.

In the *C*. *cunea* antennal transcriptomes, a total of 25 OBP genes were annotated based on tBLASTn results with an E-value of 1E-5 or lower (S2 Table). The nucleotide sequences of the OBP genes identified are listed in S3 Table. Among the 25 OBP genes, all have full-length ORFs that ranging in size from 330–681 bp. A neighbor-joining tree of OBPs from three hymenopteran insects showed that most *C*. *cunea* OBPs, with the exception of CcOBP14 and CcOBP15, which had high bootstrap support (98), did not form monophyletic groups (S1 Fig). RPKM analysis revealed that 12 OBP genes (CcOBP1- CcOBP12) are relatively abundant in the male and female antennal transcriptomes with value > 100 (S2 Table). RT-PCR analyses indicated 15 OBP genes (CcOBP1, CcOBP2, CcOBP4, CcOBP8, CcOBP9, CcOBP11, CcOBP13, CcOBP17, CcOBP18, CcOBP19, CcOBP20, CcOBP22, CcOBP23, CcOBP24 and CcOBP25) were uniquely or primarily expressed in the male and female antennae (Figs 4–6).

**Fig 7:**
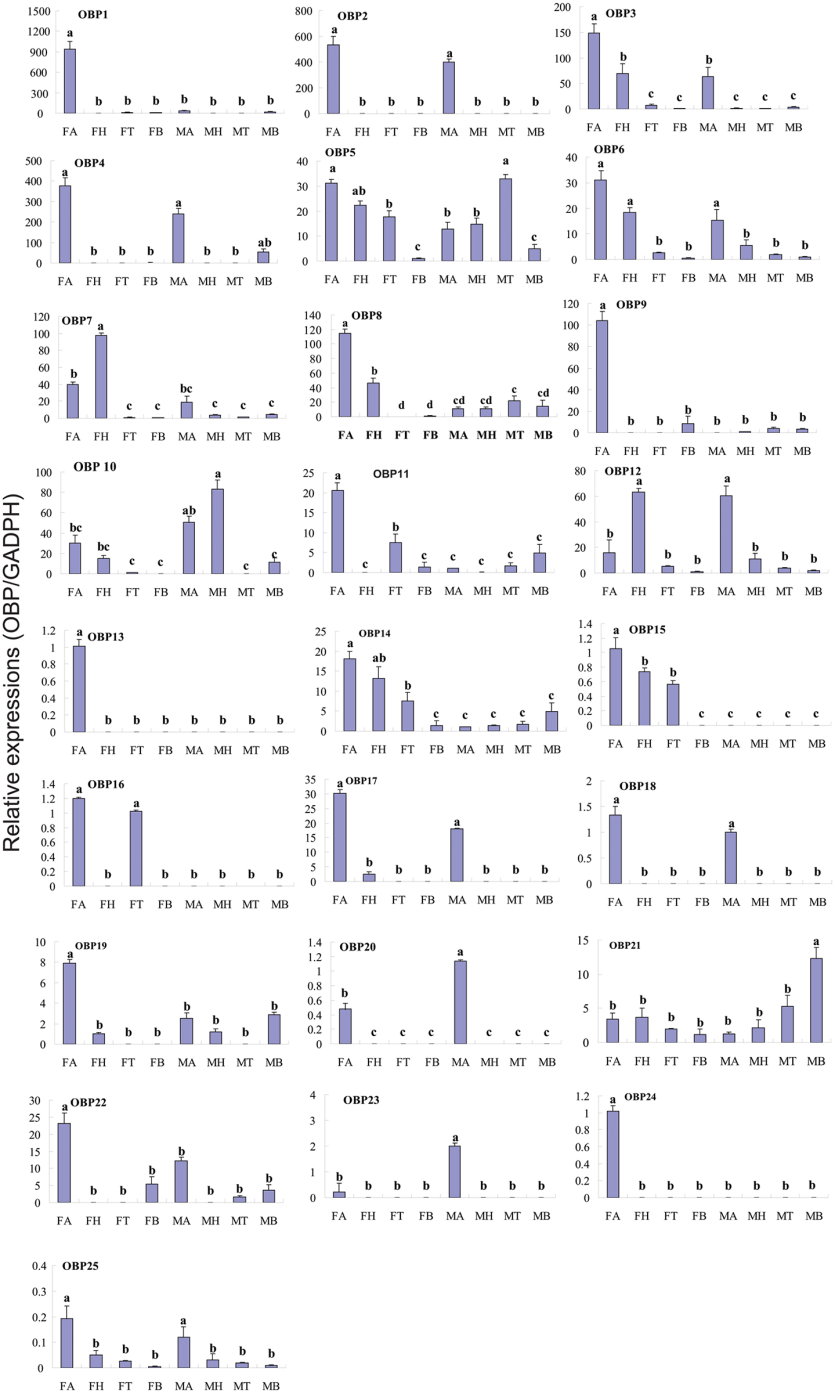
*C*.*cunea* OBPs transcript levels in different tissues as measured by RT-qPCR. MA: male antennae; FA: female antennae; MH: male head; FH: female head; MT: male thorax; FT: female thorax; MB: male abdomen; FB: female abdomen. The glyceraldehyde-3-phosphate dehydrogenase (GADPH) was used to normalize transcript levels in each sample. The standard error is represented by the error bar, and the different letters (a, b, c) above each bar denote significant differences (p<0.05).

Real-time quantitative PCR (RT-qPCR) analysis was also performed to compare the accurate quantitative expression levels of these OBP genes among different tissues between the sexes. The OBPs had a wide range of expression patterns (Fig 7): Eight genes, OBP1, OBP8, OBP9, OBP11, OBP13, OBP19, OBP22 and OBP24 had significantly greater difference levels of mRNA expression in female than in male antennae (p<0.05). OBP20 and OBP23, in contrast had significantly greater difference expression in male antennae than in female antennae (p<0.05). The expression levels of OBP2, OBP4, OBP17, OBP18 and OBP25 were similar in the antennae of two sexes. Ten OBPs (OBP3, OBP5, OBP6, OBP7, OBP10, OBP12, OBP14, OBP15, OBP16 and OBP21) were expressed at different levels in all tested tissues. Some OBPs (OBP7, OBP10, OBP12, OBP14 and OBP15) had relatively high expression in the head. OBP21 had significantly greater difference expression in male abdomen, a non-olfactory tissue than other tested tissues (p<0.05) (Fig 7). Pearson correlation coefficient analyses showed a significant positive correlation between qPCR results and RPKM values (Female: Pearson coefficient (r) = 0.918, p = 0.00 < 0.05; Male: Pearson coefficient = 0.743, p = 0.00 < 0.05) (S2 Table and Fig 7).

### Candidate odorant receptors in the *C*. *cunea* antennae

In the *C*. *cunea* antennal transcriptomes, a total of 80 OR genes (79 typical ORs and one atypical coreceptor Orco) were annotated based on the tBLASTn results with an E-value of 1E-5 or lower. Eight of the putative OR sequences had fully intact ORFs encompassing start and stop codons with lengths ranging from 1167–1440 bp (S4 Table). The *C*. *cunea* OR nucleotide sequences are listed in S3 Table. A neighbor-joining tree of ORs from three hymenopteran insects showed that most ORs from *C*. *cunea* did not form monophyletic groups. The lone exceptions were CcOR25 and CcOR48, which formed monophyletic groups with strong bootstrap support (100). The olfactory co-receptor family is highly conserved across the three hymenopteran species (S2 Fig). RPKM analyses revealed that Orco was the most highly expressed of the 80 CcORs, with RPKM values of 9.2183 and 30.2326 in male and female antennae, respectively. The other 79 typical ORs, however, had relatively low expression levels (RPKM values ranging from 0 to 10.6553) as compared with the Orco, OBP, and CSP sequences. CcOrco and CcOR1 through CcOR13 were selected for additional RT-PCR and quantitative RT-qPCR analyses to assess their expression in different tissues (antenna, head, thorax, and abdomen) of males and females. RPKM values suggested that all 14 ORs were more abundant in the female antenna compared to male. Two ORs, CcOR70 and CcOR71 were also chosen as representative OR with male dominant expression (S4 Table).

**Fig 8:**
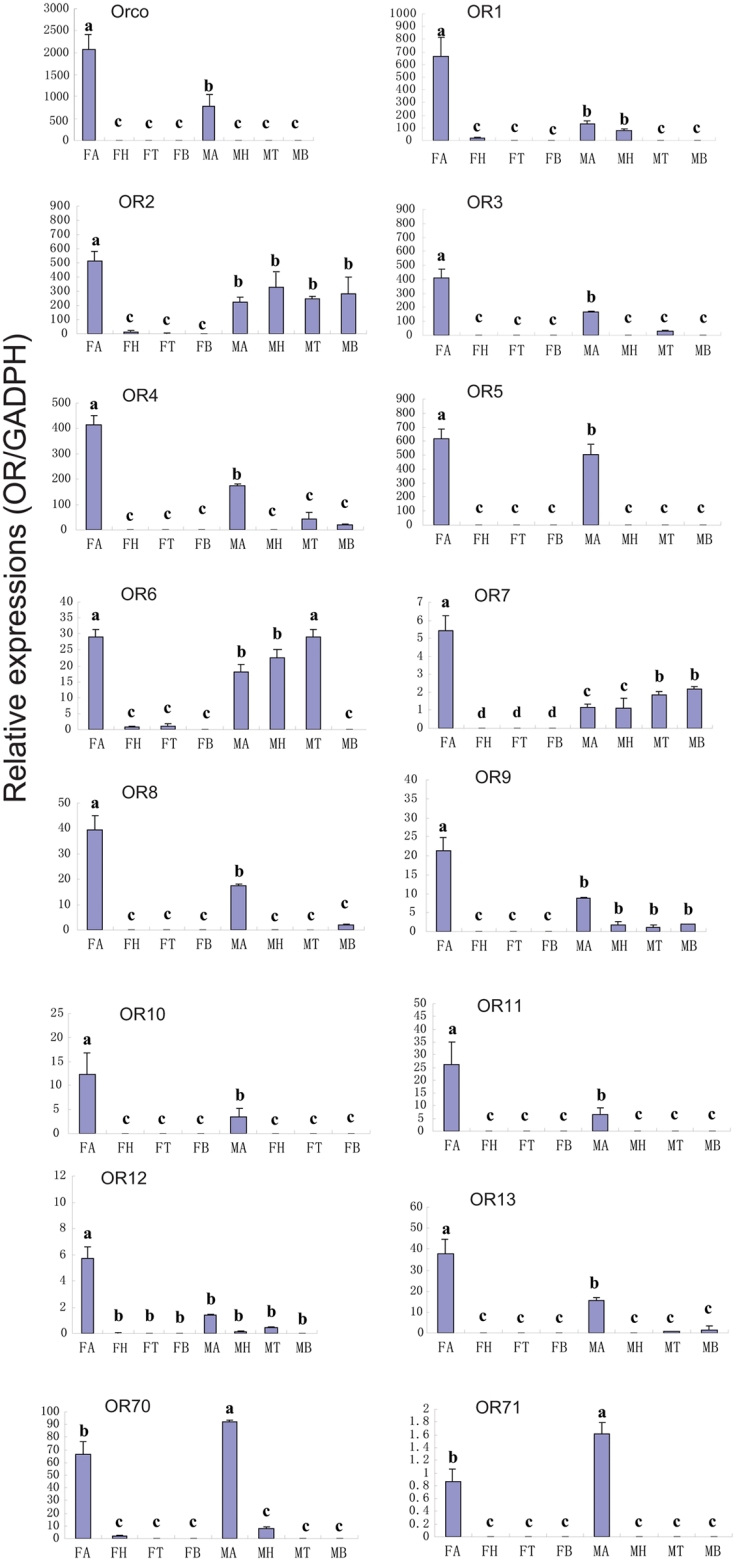
*C*.*cunea* ORs transcript levels in different tissues as measured by RT-qPCR. MA: male antennae; FA: female antennae; MH: male head; FH: female head; MT: male thorax; FT: female thorax; MB: male abdomen; FB: female abdomen. The glyceraldehyde-3-phosphate dehydrogenase (GADPH) was used to normalize transcript levels in each sample. The standard error is represented by the error bar, and the different letters (a, b, c) above each bar denote significant differences (p<0.05).

The RT-PCR and RT-qPCR results indicated that most of the OR genes (15 ORs: CcOrco, CcOR1-5, CcOR7-13, CcOR70-OR71) were exclusively or primarily expressed in the antennae (Figs 5 and 8). Futhermore, expression of 14 of these ORs (CcOrco, CcOR1-OR5 and CcOR7- OR13) was significantly greater difference in the antennae of the female wasps than in of male wasps (p<0.05). Consistent with the RNA sequencing data, CcOR70 and CcOR71 were expressed at significantly greater difference in males than in females (p<0.05). CcOR6 was expressed at varying levels in the other tested tissues. Pearson correlation coefficient analyses showed significant positive correlation of qPCR results and RPKM values (Female: Pearson coefficient (r) = 0.715, p = 0.03 < 0.05; Male: Pearson coefficient = 0.797, p = 0.00 < 0.05). These RT-qPCR results are consistent with RPKM values (Fig 8 and S4 Table).

### Candidate gustatory receptors (GRs) in *C*. *cunea* antennae

**Fig 9:**
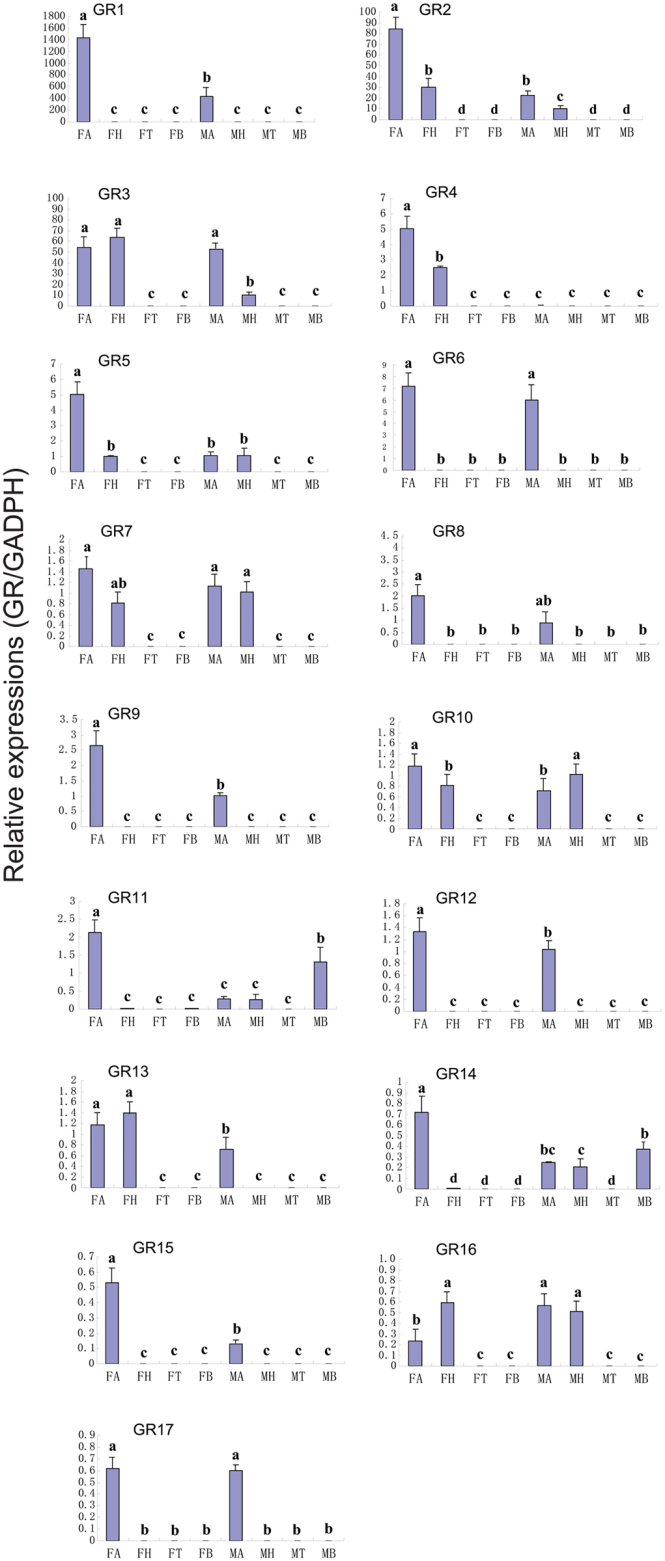
*C*.*cunea* GRs transcript levels in different tissues as measured by RT-qPCR. MA: male antennae; FA: female antennae; MH: male head; FH: female head; MT: male thorax; FT: female thorax; MB: male abdomen; FB: female abdomen. The glyceraldehyde-3-phosphate dehydrogenase (GADPH) was used to normalize transcript levels in each sample. The standard error is represented by the error bar, and the different letters (a, b, c) above each bar denote significant differences (p<0.05).

In the present study, we identified 17 GR genes, albeit none encompassing complete ORFs (S5 Table). The nucleotide sequences of the putative CcGR transcripts are listed in S3 Table. A neighbor-joining tree of GRs from four hymenopteran species showed that most GRs from *C*. *cunea* did not form monophyletic group, exceptions included two monophyletic branches comprised of CcGR3/CcGR14 and CcGR2/CcGR16 (S3 Fig). RT-PCR and RT-qPCR results revealed that most of the GRs (CcGR1, CcGR2, CcGR4-CcGR6, CcGR8, CcGR9,CcGR11, CcGR12,CcGR14, CcGR15 and CcGR17) were exclusively or primarily expressed in the antennae (Figs 6 and 9). The expression of 8 GRs (CcGR1, CcGR2, CcGR4, CcGR5,CcGR9,CcGR12, CcGR14 and CcGR15) in the antennae of female wasps was significantly greater difference than in the antennae of male wasps (p<0.05), whereas CcGR6,CcGR8 and CcGR17 genes were expressed at similar levels in females and males. CcGR3, CcGR7, CcGR10, CcGR13 and CcGR16 were also expressed in the heads of males and females at different levels. CcGR11 and CcGR14 were also expressed in the male abdomens. Pearson correlation coefficient analyses showed a significant correlation of qPCR results and RPKM values (Female: Pearson coefficient (r) = 0.999, p = 0.00 < 0.05; Male: Pearson coefficient = 0.795, p = 0.00 < 0.05) (Fig 9 and S5 Table).

### Candidates for other chemosensory genes

**Fig 10:**
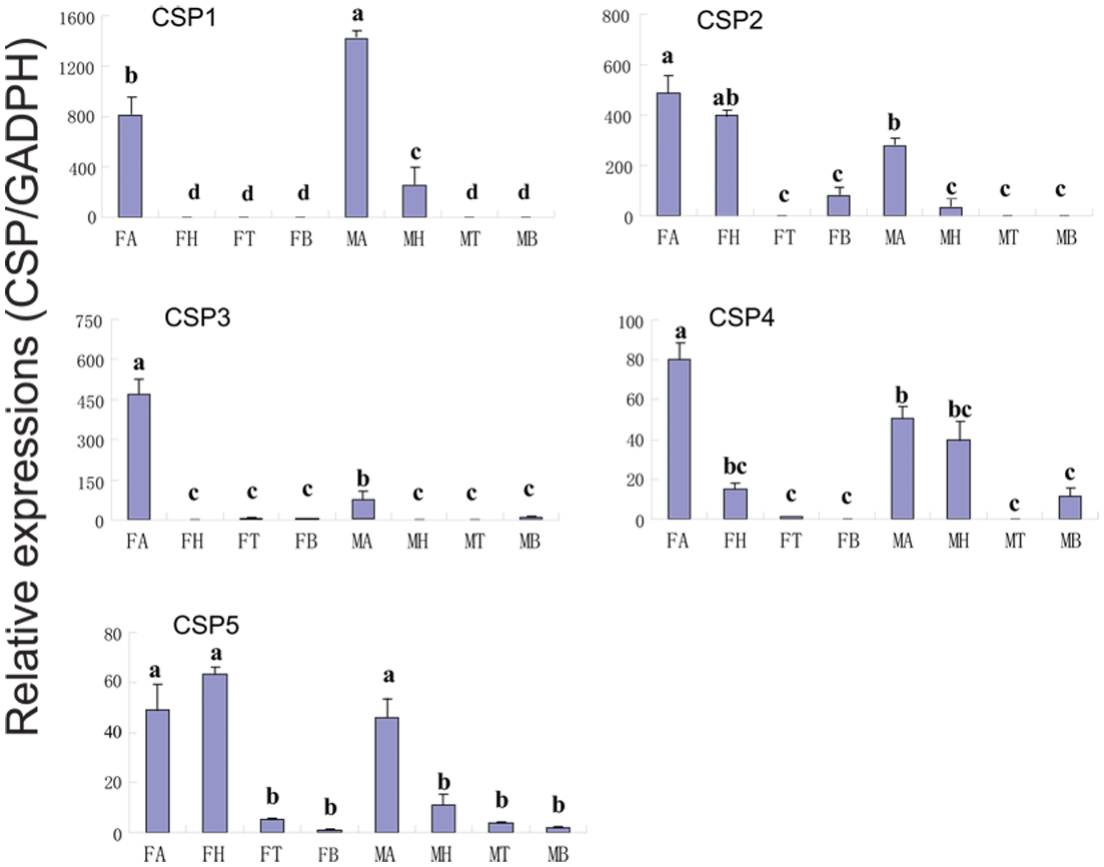
*C*.*cunea* CSPs transcript levels in different tissues as measured by RT-qPCR. MA: male antennae; FA: female antennae; MH: male head; FH: female head; MT: male thorax; FT: female thorax; MB: male abdomen; FB: female abdomen. The glyceraldehyde-3-phosphate dehydrogenase (GADPH) was used to normalize transcript levels in each sample. The standard error is represented by the error bar, and the different letters (a, b, c) above each bar denote significant differences (p<0.05).

We identified 11 putative CSP genes in *C*. *cunea* antennae, five of which encompass full-length ORFs (S6 Table). The nucleotide sequences of these transcripts are listed in S3 Table. A neighbor-joining tree of CSPs from four hymenopteran species suggested orthologous sequences in *C*. *cunea*. However, a number of the CcCSPs formed monophyletic groups: CcCSP4/CcCSP8 (bootstrap: 80), CcCSP11/CcCSP6 (bootstrap: 100) and CcCSP3/CcCSP5 (bootstrap: 100) (S4 Fig). We conducted RT-PCR and quantitative RT-PCR analyses using different tissues (antenna, head, thorax, and abdomen) of adult males and females to assess the expression of CcCSP1—CcCSP5. CcCSP1 and CcCSP3 were exclusively or primarily expressed in the antennae (Figs 6 and 10). Furthermore, the expression of CcCSP3 in the antennae of female wasps was significantly greater difference than in the antennae of male wasps, while CSP1 in the antennae of male wasps was significantly greater difference than in the antennae of female wasps (p<0.05). CcCSP2, CcCSP4 and CcCSP5 were expressed at varying levels in the other tissues tested. Pearson correlation coefficient analyses showed a significant positive correlation between qPCR results and RPKM values (Female: Pearson coefficient (r) = 0.984, p = 0.03 < 0.05; Male: Pearson coefficient = 0.993, p = 0.01 < 0.05) (Fig 10 and S6 Table).

**Fig 11:**
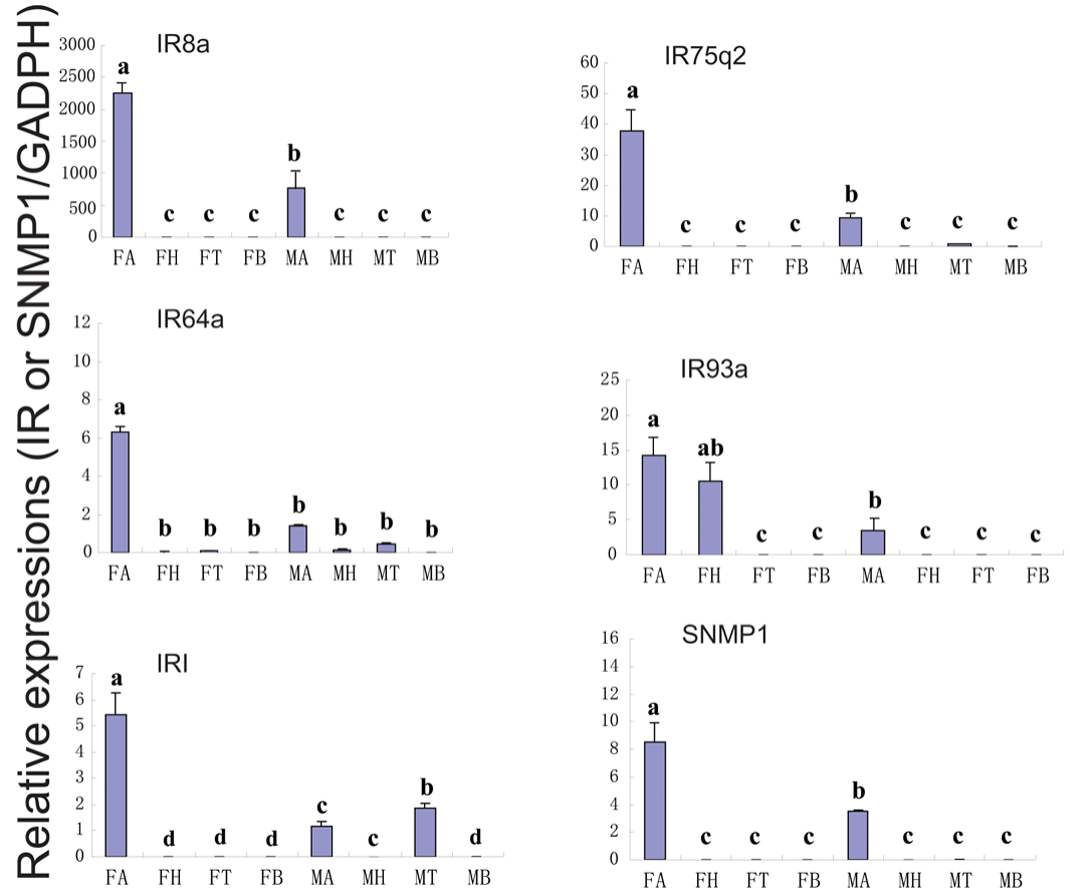
*C*.*cunea* IRs and SNMP transcript levels in different tissues as measured by RT-qPCR. MA: male antennae; FA: female antennae; MH: male head; FH: female head; MT: male thorax; FT: female thorax; MB: male abdomen; FB: female abdomen. The glyceraldehyde-3-phosphate dehydrogenase (GADPH) was used to normalize transcript levels in each sample. The standard error is represented by the error bar, and the different letters (a, b, c) above each bar denote significant differences (p<0.05).

In addition, we identified 10 putative IRs including three full length ORFs from the *C*. *cunea* antennal transcriptomes (S7 Table). The nucleotide sequences of the CcIR transcripts are listed in S3 Table. A neighbor-joining tree of IRs from two hymenopteran and one dipteran species suggested potential orthologs to the most of CcIR sequences (S5 Fig). Similar to the ORs, RPKM value analysis revealed that all CcIRs had relatively low expression levels (RPKM value ranged from 0 to 33) compared with the OBPs and CSPs (S7 Table). As before, we conducted RT-PCR and quantitative RT-PCR analyses using different tissues (antenna, head, thorax, and abdomen) of adult males and females to assess the expression of CcIR8a, CcIR75q2, CcIR64a, CcIR93a and CcIR1. The RT-PCR and RT-qPCR results showed that four IRs (CcIR8a, CcIR75q2, CcIR64a and CcIR1) were exclusively or primarily expressed in the antennae (Figs 6 and 11). The expression of CcIR8a, CcIR75q2, CcIR64a and CcIR1 in female antennae was significantly greater difference than in male antennae (p<0.05). CcIR93a was also expressed in female head. Pearson correlation coefficient analyses showed a significant positive correlation between qPCR results and RPKM values (Female: Pearson coefficient (r) = 0.997, p = 0.00 < 0.05; Male: Pearson coefficient = 0.978, p = 0.04 < 0.05) (Fig 11 and S7 Table). We also identified a single full length ORF (1050 bp) encoding a putative SNMP gene, CcSNMP1 (S7 Table), the nucleotide sequences of which SNMP genes that were identified from the *C*. *cunea* antennal transcriptomes is listed in S3 Table. It has fully intact ORF with a length of 1050 bp, and RPKM values of 0.3906 and 3.7699 in male and female antennae, respectively, suggest relatively low expression. Both RT-PCR and RT-qPCR showed that SNMP1 is exclusively expressed in the antennae (Figs 6 and 11) with significantly greater difference levels observed in female antennae than in male antennae (p<0.05).

## Discussion

The molecular basis of chemoreception in parasitoids is not well understood compared to pest insects. In the present study, we generated antennal transcriptomes of male and female *C*. *cunea* using next-generation sequencing technology. We identified 144 chemosensory genes encoding members of six protein families, including 25 OBPs, 80 ORs, 10 IRs, 11 CSP, 1 SNMPs, and 17 GRs. However, transcripts present in low abundance and those that were too divergent to be identified using a BLAST search may have been overlooked in transcriptome analysis [76]. Thus, it is unlikely that the identified genes represent the total number of the related chemosensory genes in *C*. *cunea*. However, this is the first comprehensive characterization of chemosensory genes in *C*. *cunea*. Our findings provide insight into the molecular mechanisms of chemoreception in *C*. *cunea*, and identify potential molecular targets for possible use in biological control strategies. *C*. *cunea* is an endoparasitic chalcid wasp that parasitizes the pupa of the fall webworm. Inside its host pupa, *C*. *cunea* develops from the egg to the pre-oviposition adult stage. The adult wasps mate inside the host pupa, and then chew a hole in the host pupal shell. The other wasps exit the pupa using this same hole. Thus, the adult females can parasitize new hosts soon after their ‘emergence’ [58]. In this study, the wasps we collected were emerged adult wasps within 24 h from the host pupa. So the emerging wasps had completed mating. We assume that, these chemosensory genes with female antennal-specific or dominant expression profiles may play important roles in locating suitable hosts and oviposition sites. This however, should be verified in further experiments.

In this study, Pearson correlation coefficient analyses showed significant positive correlation between qPCR results and RPKM values of all of the tested chemosensory genes. It shows that qPCR results agree well with the RPKM analyses and indicates that RNAseq count data on steady state transcript levels [77]. And it also suggests that the results of RNAseq count data are confident. A total of 25 OBPs were identified in the antennal transcriptome of *C*. *cunea*. The number of *C*. *cunea* OBP identified was smaller than that identified from the genomes of *B*. *mori* (44) [78], *A*. *gambiae* (57) [79], *D*. *melanogaster* (51) [80], and *A*. *ipsilon* (33) [81]. There may be OBP genes that are not yet identified in *C*. *cunea* antennae due to low expression levels. Moreover the lab-reared wasps had no exposure to host insect odors or plant odors in natural surroundings. There may be OBP genes not yet identified due to this lack of exposure [49]. Many OBPs in insects have a wide range of expression patterns among different tissues of males and females [48, 76, 81]. Here, 15 OBPs had obvious antennal-specific expression profiles. Based on the different expression profiles of these OBPs in male and female antennae, we suggest that these male or female antennae-enriched OBPs may play a role in sex-specific behaviors. A total of eight OBPs had obvious female antennal-specific expression. These genes may play important roles in locating suitable hosts and oviposition sites. The remaining 10 OBPs were expressed at different levels in a variety of tissues. These may be related to other functions [82]. A total of 4 OBPs had relatively high expression in the head. This expression may be in the maxillary or labial palps; however, this should be experimentally verified. One OBP (OBP21) was highly expressed in a non-olfactory body section (abdomen of the male) and may be a carrier of ligands other than odorants [83]. Future functional investigation of these genes is warranted to determine their specific roles.

Insect olfactory receptors (ORs) are the most important components in sex pheromone and general odorant detection [81]. In this study, a total of 80 ORs were identified in the antennal transcriptome of *C*. *cunea* (79 typical ORs and the atypical co-receptor Orco). The number of ORs identified in *C*. *cunea* is fewer than in *A*. *mellifera (*170) [84] and migratory locust (142) [85], but was much greater than in the parasitoids *M*. *mediator* (60) [49] and *C*. *vestalis* (6) [48]. Most ORs in insects are extensively expressed in the antennae [86]. In our study, most of the tested ORs showed an antennal-specific expression profile. Expression of a majority of the examined ORs (14/16) was much higher in the antennae of female wasps than in male antennae. The ORs with antennal-specific or dominant expression profiles may play crucial roles in the olfactory chemoreception of wasps. These female antennal-specific or dominant expression genes may play crucial roles in locating suitable hosts and oviposition sites. We also identified several ORs expressed in non-chemosensory tissues, similar to the findings in other studies [81, 87–89]. The expression of ORs in non-olfactory tissues suggests that they may have physiological functions in other organs. Unlike other studies that showed Orco expression in other tissues [88, 90], Cc Orco had a clear antennae-specific expression pattern. This is consistent with studies in *Agrotis ipsilon* [81]. The OR tree showed that the Orco are highly conserved (S2 Fig). Based on the analysis of selection pressure, CcOrco appears to be primarily negatively selected [91], which likely explain the conservation of CcOrco.

GRs are generally expressed in gustatory receptor neurons (GRNs) within gustatory organs [55] and are crucial for responses to soluble taste and contact pheromones [11, 56]. However, some GRs are also expressed in antennal dendrites and respond to carbon dioxide, potentially implicating them in olfaction [92, 93]. GRs are more highly conserved in sequence and structure than ORs [94, 95], a feature that has been suggested to be due to the comparatively smaller search space among cues associated with GRs than ORs. Because GR expression levels are quite low and mainly expressed in gustatory organs [96, 97], only 17 putative GR-encoding transcripts were identified from *C*. *cunea* antennae. However, this number is greater than that in *M*. *mediator* (2), which was the first report of GR genes in wasp antennae [49]. Similar to ORs, *C*. *cunea* GRs that were identified in the antennal transcriptome were primarily expressed in the antennae. Expression of eight CcGRs in the antennae of female wasps was much higher than in the antennae of male wasps. It is will be interesting to determine if these GRs function in locating oviposition sites. Another five GRs were expressed at varying levels in other tested tissues. These proteins may have multiple functions in insect chemoreception, growth, and development.

Chemosensory proteins (CSPs) represent a more recently discovered soluble carrier proteins that probably function in a manner similar to OBPs in insect chemoreception [98]. These proteins have broad expression profiles in chemosensory tissues, including antennae [24, 25, 28, 26], maxillary palps [29], labial palps [29, 30], and proboscis [31]. However, these proteins are also found in non-chemosensory organs, such as legs [32, 33], wings [34, 56], and pheromone glands [26]. We identified 11 CSPs in *C*. *cunea*. In our study, two of the five tested CSPs showed an obvious female antennal-specific expression profile. These CSPs may play important roles in odorant detection.

Insect chemosensory ionotropic receptors (IRs) belong to an ancient chemosensory receptor family that was first discovered in *D*. *melanogaster*. They are expressed in sensory neurons that respond to different odorants but do not express either ORs or gustatory receptors (GRs) [7]. At present, 66 IRs have been identified in *D*. *melanogaster* [7, 12], 17 IRs in the *Spodoptera littoralis* [16, 99], 21 IRs in *Manduca sexta* [100], 15 IRs in *Cydia pomonella* [13] and 12 IRs in *Helicoverpa armigera* [17] have been identified. Because of IRs have relatively well conserved sequence homology across insect orders compared to other chemosensory genes, the CcIRs were named based on homology to similar genes in other species. The neighbor-joining tree for IRs from four hymenopteran species showed that most IRs clustered with their respective orthologs. It also showed that the IRs are highly conserved. The RPKM value analysis revealed that all the CcIRs had relatively low expression levels compared with the OBPs and CSPs. Four of the five CcIRs tested by RT-PCR and RT-qPCR had female antennal-specific expression profiles, suggesting that these genes may play roles in locating suitable hosts and oviposition sites.

Insect SNMPs are two trans-membrane domain-containing proteins that may play significant roles in insect chemoreception [18, 29, 101]. Two SNMP subfamilies, SNMP1 and SNMP2, have been identified in insects; however, these subfamilies show different expression profiles in the antennae sensilla: SNMP1 proteins are detected in pheromone-sensitive olfactory receptor neurons (ORNs) [102–104]; however, SNMP2 proteins are expressed in the supporting cells [99, 100]. In the present study, we identified one SNMP gene, CcSNMP1 that was antennal-specific. The expression of CcSNMP1 in the antennae of female wasps was much higher than in the antennae of males. In certain insects, a high level of antenna-specific expression has been observed for SNMP1, whereas SNMP2 is expressed at various levels in a variety of tissues [19, 105]. However, there are few studies of SNMPs in Hymenoptera. We speculate that the level of SNMP2 expression in the antenna of *C*. *cunea* may be too low, and thus undetectable based on the transcriptome analysis.

In this study, we identified and annotated several groups of chemosensory genes in the antennae of *C*. *cunea*. An expression profile analysis revealed that some chemosensory genes are uniquely or primarily expressed in female antennae. One possible explanation for the observed female-biased expression genes is that female *C*. *cunea* have large numbers of sensilla trichodea present on their antennae that are much more than the male [106]. These female antennal-specific or dominant expression OBPs, CSPs, ORs, GRs, IRs, and SNMPs may be important for locating suitable hosts and oviposition sites. The biological functions of these genes and their products are still poorly known but our results should help pave the way for future studies of this nature.

## Supporting Information

S1 Fig Neighbor-joining tree of candidate OBPs from *C*.*cunea* (red), *Microplitis mediator* (green) and *Nasonia vitripennis* (blue). The protein names and sequences of OBPs that were used in this analysis are listed in S8 Table. Bootstrap supports are given at the node. The values greater than or equal to 50 were shown on the tree.
(TIF)

S2 Fig Neighbor-joining tree of candidate ORs from *C*.*cunea* (red), *Microplitis mediator* (green) and *Nasonia vitripennis* (blue). The protein names and sequences of ORs that were used in this analysis are listed in S9 Table. Bootstrap supports are given at the node. The values greater than or equal to 50 were shown on the tree.
(TIF)

S3 Fig Neighbor-joining tree of candidate GRs from *C*.*cunea* (red), *Microplitis mediator* (green), *Sclerodermus* sp. (purple) and *Nasonia vitripennis* (blue). The protein names and sequences of GRs that were used in this analysis are listed in S10 Table. Bootstrap supports are given at the node. The values greater than or equal to 50 were shown on the tree.
(TIF)

S4 Fig Neighbor-joining tree of candidate CSPs from *C*.*cunea* (pink), *Solenopsis invicta* (green), *Camponotus japonicus* (orange) and *A*. *cerana* (blue). The protein names and sequences of CSPs that were used in this analysis are listed in S11 Table. Bootstrap supports are given at the node. The values greater than or equal to 50 were shown on the tree.
(TIF)

S5 Fig Neighbor-joining tree of candidate IRs from *C*.*cunea* (red), *M*. *mediator* (blue), and *Drosophila melanogaster* (green). The protein names and sequences of IRs that were used in this analysis are listed in S12 Table. Bootstrap supports are given at the node. The values greater than or equal to 50 were shown on the tree.
(TIF)

S1 Table Primers used for RT-PCR and RT-qPCR analysis of olfactory genes of the *C*.*cunea*. (DOC)

S2 Table List of OBP genes in *C*.*cunea* antennae. (DOCX)

S3 Table The nucleotide sequences of 25 OBPs, 11 CSPs, 80 ORs, 10 IRs, 1 SNMPs and 17 GRs of *C*.*cunea* identified in present study. (DOC)

S4 Table List of OR genes in *C*.*cunea* antennae. (DOCX)

S5 Table List of GR genes in *C*.*cunea* antennae. (DOCX)

S6 Table List of CSP genes in *C*.*cunea* antennae. (DOCX)

S7 Table List of IR and SNMP genes in *C*.*cunea* antennae. (DOCX)

S8 Table The protein names and sequences of OBPs that were used in phylogentic tree analysis. (DOCX)

S9 Table The protein names and sequences of ORs that were used in phylogentic tree analysis. (DOCX)

S10 Table The protein names and sequences of GRs that were used in phylogentic tree analysis. (DOCX)

S11 Table The protein names and sequences of CSPs that were used in phylogentic tree analysis. (DOCX)

S12 Table The protein names and sequences of IRs that were used in phylogentic tree analysis. (DOCX)
