# Supplementary figures and images for: Transcriptome and Expression Patterns of Chemosensory Genes in Antennae of the Parasitoid Wasp Chouioia cunea
Source: PLoS One. 2016 Feb 3;11(2):e0148159. doi: 10.1371/journal.pone.0148159 (PMC4739689; doi:10.1371/journal.pone.0148159)

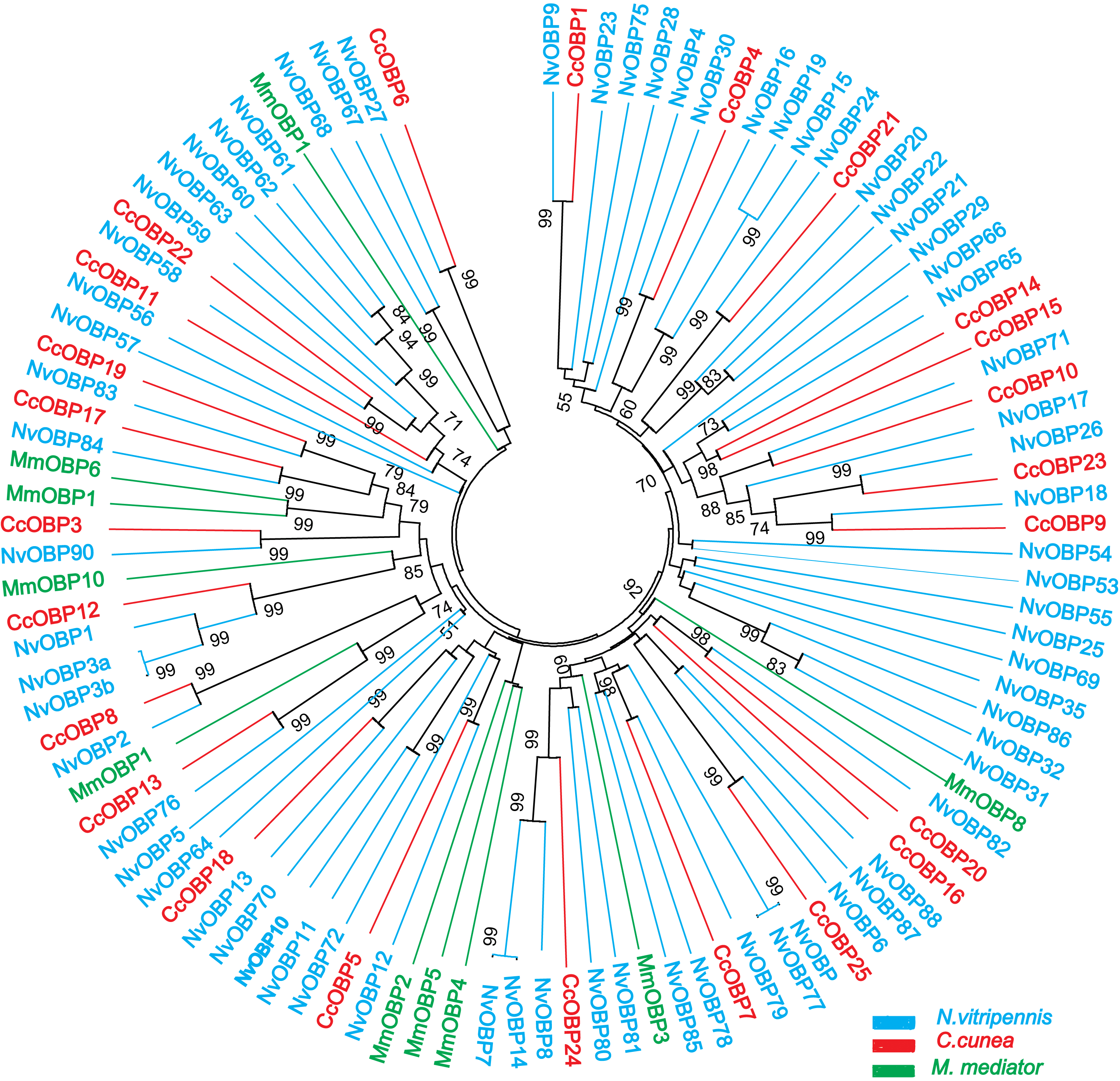

Supplement: S1 Fig — The protein names and sequences of OBPs that were used in this analysis are listed in S8 Table. Bootstrap supports are given at the node. The values greater than or equal to 50 were shown on the tree. (TIF) [file pone.0148159.s001.tif]

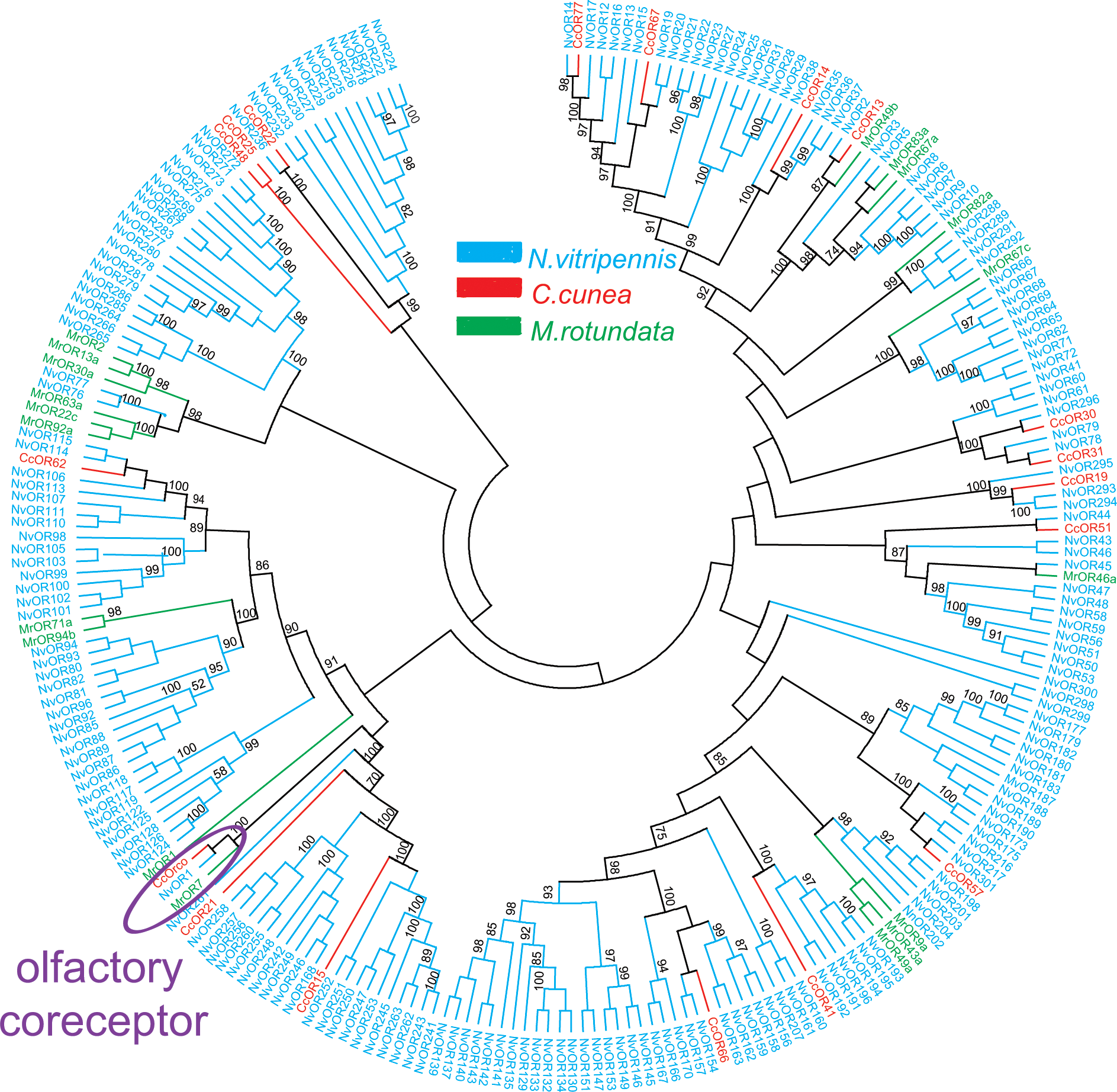

Supplement: S2 Fig — The protein names and sequences of ORs that were used in this analysis are listed in S9 Table. Bootstrap supports are given at the node. The values greater than or equal to 50 were shown on the tree. (TIF) [file pone.0148159.s002.tif]

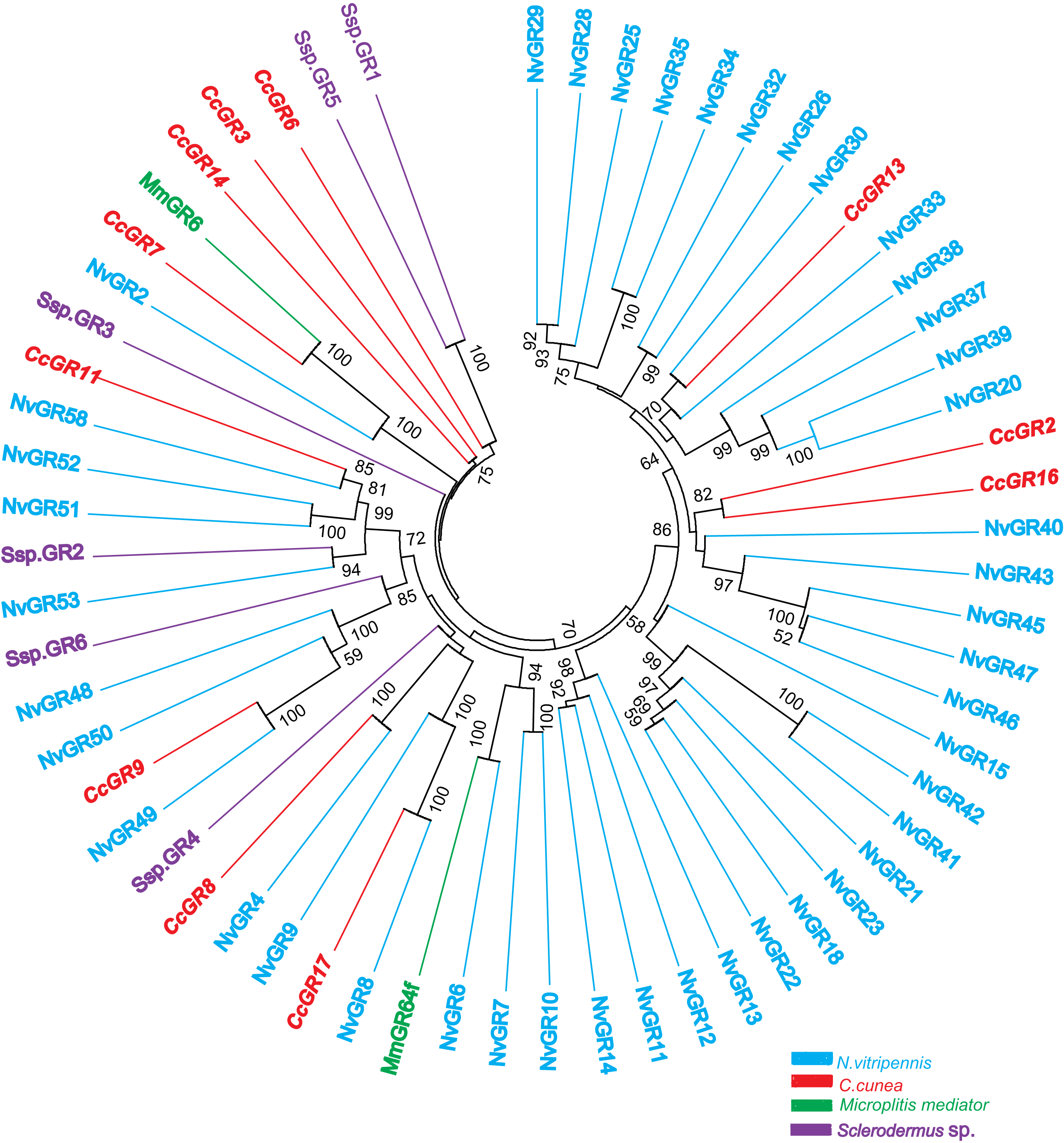

Supplement: S3 Fig — The protein names and sequences of GRs that were used in this analysis are listed in S10 Table. Bootstrap supports are given at the node. The values greater than or equal to 50 were shown on the tree. (TIF) [file pone.0148159.s003.tif]

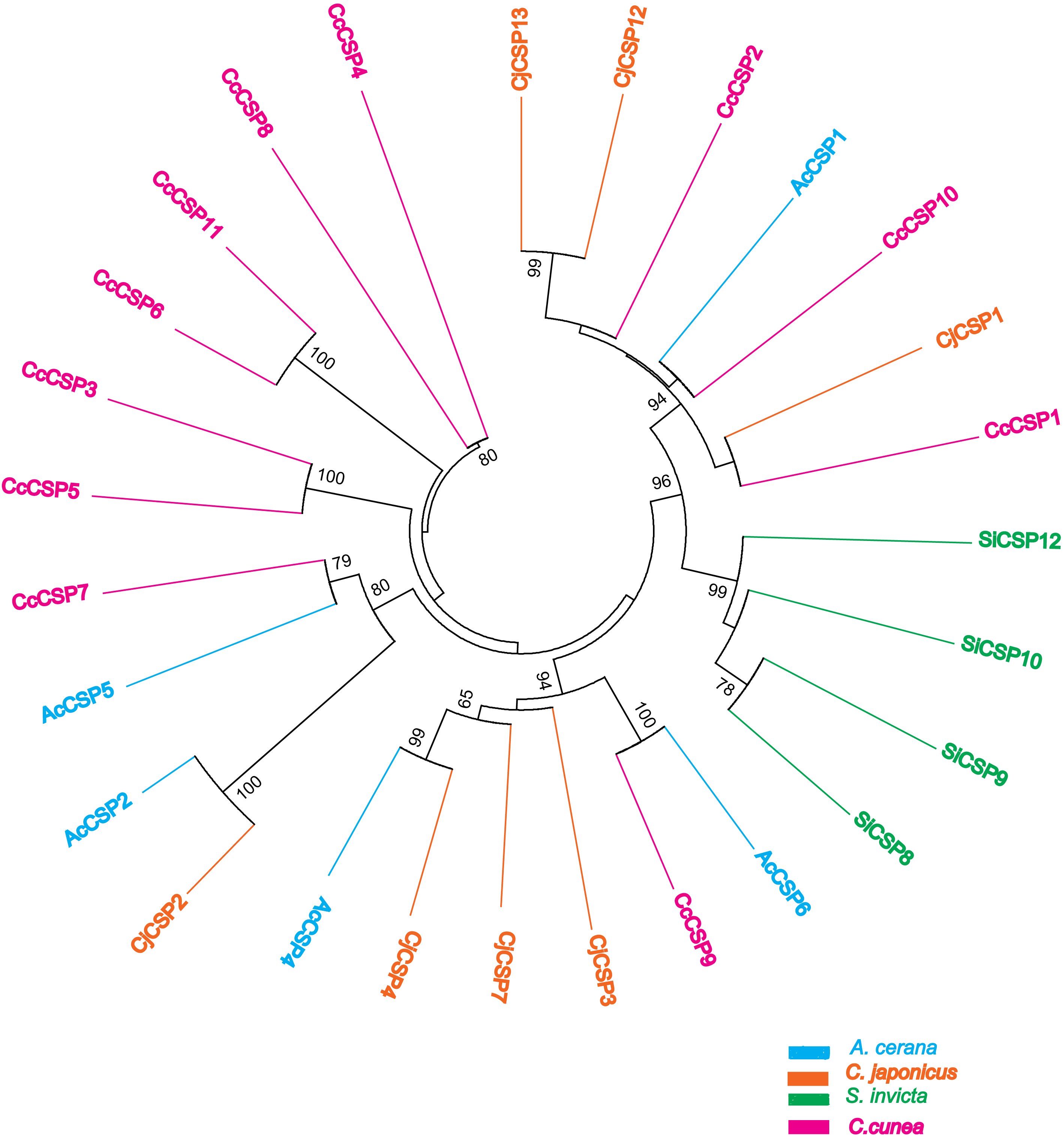

Supplement: S4 Fig — The protein names and sequences of CSPs that were used in this analysis are listed in S11 Table. Bootstrap supports are given at the node. The values greater than or equal to 50 were shown on the tree. (TIF) [file pone.0148159.s004.tif]

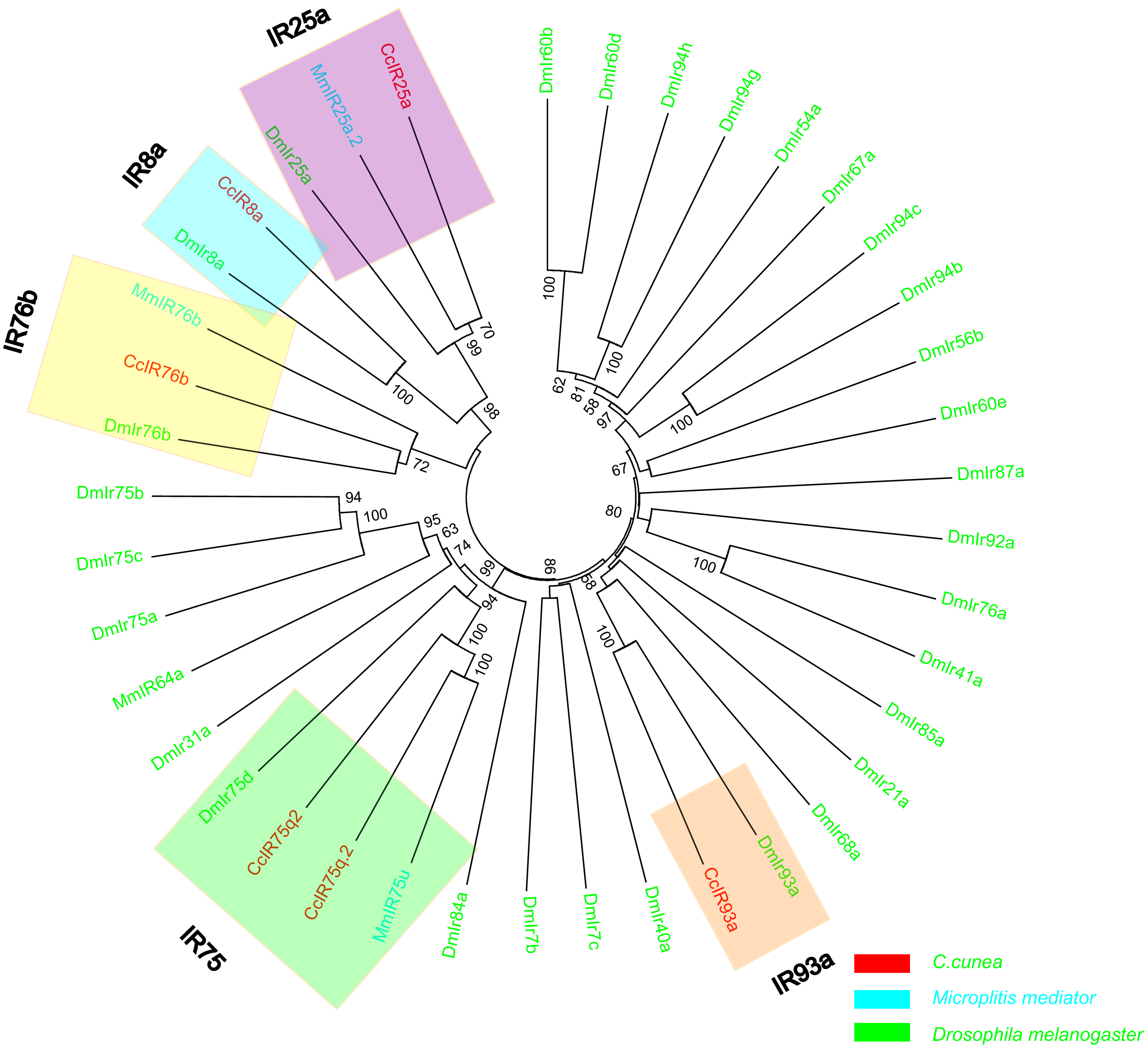

Supplement: S5 Fig — The protein names and sequences of IRs that were used in this analysis are listed in S12 Table. Bootstrap supports are given at the node. The values greater than or equal to 50 were shown on the tree. (TIF) [file pone.0148159.s005.tif]
