# Supplementary material for: Transcriptome and Expression Patterns of Chemosensory Genes in Antennae of the Parasitoid Wasp Chouioia cunea
Source: PLoS One. 2016 Feb 3;11(2):e0148159. doi: 10.1371/journal.pone.0148159 (PMC4739689; doi:10.1371/journal.pone.0148159)
Supplement: S1 Table — (DOC) [file pone.0148159.s006.doc]

**S1 Table.** Primers used for RT-PCR and RT-qPCR analysis of olfactory genes of the *C.cunea*.

| Primer name | Forward primer (5’-3’) | Reverse primer (5’-3’) |
| --- | --- | --- |
| Odorant binding proteins | | |
| OBP1 | TCATACAAGCGTAATAGCAGT | CCCTAAGGACCTCATCAA |
| OBP2 | TTGCGTTGTTGGAGCTTACGCTG | TTTGTCGCGCTTGATTTCTCC |
| OBP3 | GTAATTTGCATCTTGATACTAAGTG | GCTAAATCTGCTCCTGTT |
| OBP4 | ATGCAACTCTTCGACGTCGCTTGCA | TTCTTCGGTTAGATACTTCA |
| OBP5 | ATGCATTCCTTTACAGTAATCGTAG | GTGAGTCCTCCTTCTTTA |
| OBP6 | GTACAGAGATATTTCAATGTCAG | CGTACACGTGTATGTATAGGT |
| OBP7 | ATGAAATTCGTTATTTTCAGTTTTT | TCTTAGGGTCATCGTGCT |
| OBP8 | GCAGGCATTTACGACACT | TGCTGCTACTCCTCTTCG |
| OBP9 | AATCCTTGGGCAGTTTGT | CGTGTCCGAAGATGTGGT |
| OBP10 | AGTATTCCTGGGGTCTCA | ATATGGCTGGTGCTTTGG |
| OBP11 | CCAATCACGAAAGAGTAGACG | TGCCATCACACAATTCCCAGGAG |
| OBP12 | ATGAAGAACGTAGTTGTTTGTT | TCGGTATTTCACCAGCAT |
| OBP13 | TCCTATTGCGCCCTCGTGCTATTGT | CACAACAATAGCACGAGGG |
| OBP14 | GTCGGTAGGTTTGTGATTGG3 | CGAAGACTTGAAAGCTGGAA |
| OBP15 | ATGAAACTGTTACTCGCACTTTGTG | GTGCGAGTAACAGTTTCAT |
| OBP16 | ATGAAAGTTCTTCTCGTTCTTGTTT | AAGCAGCAAACAAGAACGAGAA |
| OBP17 | TCGTGGCACAAGTTCGTC | AACTGGCGTTTCGGATGA |
| OBP18 | ATGCAACTCTTCGACGTCGCTTGCA | ATGCAACTCTTCGACGTCGCTTGCA |
| OBP19 | ACATACTCATCGGGCATC | TATTGACTTAGTAGACGGAACT |
| OBP20 | ATGAAGCGCGCATTTTCCGTTCTTT | AGACGCAAAGAACGGAAA |
| OBP21 | TGCCTCGTAACTTTCTTTGACT3 | TATACTGCATTCCCGCTTGT |
| OBP22 | CTCGTCCTCGTGGATTTG | CATCGGCTCACGCTTTAT |
| OBP23 | TTGCGAACCCACCAAAAGTATT | TTGAATTTGTTCAGCATACAAAC |
| OBP24 | TTGCCTCAATATCATCGT | CATTATCCAGACCTCCAA |
| OBP25 | ACTTCTCTCCTCATGATCGTTAA | CCAACGGTGCACCGCTTAGCGATG |
| Odorant receptors | | |
| Orco | AGCACCGTGATTGTATTT | ACTGTATGGTTACCCTATTCC |
| OR1 | TGGAAGGTCGTACGCATTATTTTATC | CGAAGATTGCATCAACGCGAGGTT |
| OR2 | TTTCTTGGCGCTTGGGTAGAT | AAAACTTCGATTGTTTGTACGA |
| OR3 | TCTGGAGAAACGAGAGTAAAGC | GCGTTGGCGTCGATATTTCCCCA |
| OR4 | CAATGGCACGCGGAGATAATGT | AATCGGTGAGGTTTGTATAAGTA |
| OR5 | CTCGACTTTGATCGTTATTCCTGG | GTAGAGAAATTTCAACTTGTCTTC |
| OR6 | CTACAGCTATGCTTTACCTGATGA | TAATGAACCGGTCAACATCTGACTC |
| OR7 | ACGTTTCGTGGAAAAATTT | ACTTCCATGTATTGTTTCTGGAT |
| OR8 | GTGGCCTCACAAACCAAATGACAGC | CCGTGAGTACATCGATGTCCCGC |
| OR9 | TGGCAAAAGTACATTTCCAAT | TTGTACATCGGTGGCAAGCA |
| OR10 | ATGTTGTTCGCGTTGCTAAAACA | GAGTGTCCAGAATTTCAAGACC |
| OR11 | GGGCCAACCGTGAACTCAACCG | CAACTGTTTCAATACCTGCTATT |
| OR12 | CTTACGGAAAACATGGCGTTCTCGC | TTGCTTGGTGTAATCCTTCGAATCG |
| OR13 | GGCGATGGTCGACGTCGTCCTCTTT | GTCGTTTGCGCCCAATCGTCGCGA |
| OR70 | GGAATATTTGCTAAAGTGCCACT | TATCGACTGTAAATTCTGTGCTGCG |
| OR71 | GCACACTTGGATAGTTTGCTCAAT | ATAGTGTTCGTTGTCAAATTACG |
| Gustatory receptors | | |
| GR1 | ATATTTATAATTTGTTTACTTTTTT | GTGCGAATGTATGTAAGA |
| GR2 | TTTAGGTAGCGTATCTGG | ACGTCGTATTAACGAATG |
| GR3 | AATATATACCGGAGATACCTGG | CTCGTCCAAACGAATTGATTGAA |
| GR4 | TGCAAGACTGTTTGGAAATCCCT | TTGCAAAAGTATAATTGAGTACG |
| GR5 | AAACAGCTTATTAATTTTTCTCAAT | CGTTGCGGTACAAGACAA |
| GR6 | GATACTGCAAGTGATTTTCAATGT | ATATTGTGCATCCCATCTCGTCA |
| GR7 | CTGAATTGTTCGGCGAGA | TTCCCAGGGATGTTTACC |
| GR8 | GTTTCAGGAGCCTTCCTC | TGGCATTCACCATCACTA |
| GR9 | AAGACAGTTGGTGGATTT | CTTTAGCGGTATGGATTA |
| GR10 | GGCAACGACCTTAATCAAAT | GCGTAGTAAAGGGTCCAG |
| GR11 | ACGTGTTTTCAGCTGAACCAATTT | ACTTTTAAATTGTAATAGTATAAC |
| GR12 | TTTGGTATCGTCGGCATT | CACCGCTTGTGGACTTTT |
| GR13 | GAATTTGCTGCGAATGTCGCGAA | AAAATCGATACGCAACGGTCA |
| GR14 | TTCAATGGACGATGATTCTTTAT | TGCCAGCGTCTCATCTTG |
| GR15 | GATGTACATAACGATAGTTGTG | ACTGTAGTTTGACGCTATTTCCC |
| GR16 | TATGGTTAAGAGACGTTACGCAT | TTGGATCGCAAGGTTTTCCATTG |
| GR17 | TGCAATATATGACTTACGTTATGA | TTACGAACTGTAGTTTGACGCTATTT |
| Chemosensory proteins | | |
| CSP1 | TCGTTGCCGGTGTCGTTAGAGCCG | GTAACTACAGCCTCTGGTGCGTGG |
| CSP2 | CCAATATGATTACGTCGACGTTA | TGAAGTACTGAGCGTCCGCCGT |
| CSP3 | GGCTCTTTGCATTGCTCGTGCTGC | GATAGGATAGTGATCTTTCATA |
| CSP4 | TTGTACGATCAGCAGCAGCAACG | TTCACTTTATTGGCGTTAGCGGT |
| CSP5 | GTCGTTGCTCAAGATACCAGCGAAG | AATTTGTAGTTGTATTTAGTCCA |
| Ionotropic receptors | | |
| IR8a | GCTTCGCAAGCACCCGTTACCCT | GTCGACGAACTCGCGATCCACTTG |
| IR75q2 | AGGCCTGTCGACGTTCTCAAGGGT | GATCCGGTGTACTTGCTACTGATTT |
| IR64a | GGGATTTAGCCGATAGTGACCTAGAT | ACTCTATCCGGATCTTTCCTGTATG |
| IR93a | GATGACTGCTAACGCTACTATGG | CGTCCCAAATTGATATTTGTATCT |
| IR1 | ATCTCGTCGGCATATCAATTCTACG | GTACTTCTCGCGATTGTTCTGATAGC |
| Sensory neuron membrane proteins | | |
| SNMP1 | TAAGTCCAATAGCTAAAGATCCAT | TGGGTGACTGTTTCTTGATTAACG |

|  |  |
| --- | --- |
|  |  |
|  |  |
|  |  |
|  |  |
|  |  |
|  |  |
|  |  |
|  |  |
|  |  |
|  |  |
|  |  |
|  |  |
|  |  |
|  |  |
|  |  |
