# Supplementary material for: Transcriptome and Expression Patterns of Chemosensory Genes in Antennae of the Parasitoid Wasp Chouioia cunea
Source: PLoS One. 2016 Feb 3;11(2):e0148159. doi: 10.1371/journal.pone.0148159 (PMC4739689; doi:10.1371/journal.pone.0148159)
Supplement: S2 Table — (DOCX) [file pone.0148159.s007.docx]

S2 Table. List of OBP genes in *C.cunea* antennae

| Gene | Unigene | Length of Unigene  (bp) | ORF  (bp) | BLASTx annotation | Score | E-value | % Identify | RPKM value | |
| --- | --- | --- | --- | --- | --- | --- | --- | --- | --- |
|  |  |  |  |  |  |  |  | Male | Female |
| OBP1 | Unigene9686 | 636 | 378 | gi\|355389891 putative odorant binding protein 9 [Nasonia vitripennis] | 183.7 | 5.00E-45 | 82 | 520.1815 | 4809.613 |
| OBP2 | Unigene8360_All | 1181 | 411 | gi\|355389925\| putative odorant binding protein 26 [Nasonia vitripennis] | 205.3 | 5.00E-51 | 86 | 567.452 | 2390.522 |
| OBP3 | Unigene5449_All | 1016 | 453 | gi\|355390137\| putative odorant binding protein 90 [Nasonia vitripennis] | 198.4 | 5.00E-49 | 80 | 100.0076 | 602.3484 |
| OBP4 | Unigene1089_All | 566 | 390 | gi\|355389933\| putative odorant binding protein 30 [Nasonia vitripennis] | 131.7 | 1.00E-29 | 70 | 287.8697 | 284.6671 |
| OBP5 | CL4349.Contig1_All | 614 | 408 | gi\|355389897\| putative odorant binding protein 12 [Nasonia vitripennis] | 109.4 | 1.00E-22 | 63 | 203.0083 | 263.8593 |
| OBP6 | CL2352.Contig1_All | 1263 | 423 | gi\|355389991\|putative odorant binding protein 59 [Nasonia vitripennis] | 79 | 6.00E-13 | 57 | 47.8058 | 175.1434 |
| OBP7 | Unigene1130_All | 576 | 402 | gi\|345485797\|putative odorant binding protein 79  [Nasonia vitripennis] | 105.5 | 1.00E-21 | 66 | 189.8703 | 166.6506 |
| OBP8 | CL3237.Contig2_All | 1232 | 459 | gi\|355389877\| putative odorant binding protein 2 [Nasonia vitripennis] | 61.2 | 1.00E-08 | 58 | 32.1441 | 162.0076 |
| OBP9 | Unigene11079_All | 599 | 405 | gi\|355389909\| putative odorant binding protein 18 [Nasonia vitripennis] | 191 | 2.00E-47 | 81 | 67.0637 | 161.2255 |
| OBP10 | Unigene6810_All | 604 | 414 | gi\|355390015\|putative odorant binding protein 71 [Nasonia vitripennis] | 134 | 4.00E-30 | 66 | 163.7691 | 138.5083 |
| OBP11 | Unigene6884_All | 808 | 429 | gi\|355389884\|putative odorant binding protein 6 [Nasonia vitripennis] | 191.4 | 4.00E-47 | 79 | 7.8553 | 105.3028 |
| OBP12 | Unigene4672_All | 839 | 414 | gi\|355389875\| putative odorant binding protein 1 [Nasonia vitripennis] | 229.2 | 1.00E-58 | 84 | 287.6671 | 84.8697 |
| OBP13 | Unigene7471_All | 1674 | 447 | gi\|355390025\| putative odorant binding protein 76 [Nasonia vitripennis] | 220.7 | 1.00E-55 | 85 | 3.944 | 63.4842 |
| OBP14 | Unigene3310_All | 767 | 399 | gi\|355390003\| putative odorant binding protein 65 [Nasonia vitripennis] | 87 | 1.00E-15 | 54 | 16.4071 | 59.74 |
| OBP15 | Unigene8248_All | 581 | 375 | gi\|355390005\| putative odorant binding protein 66 [Nasonia vitripennis] | 69.3 | 1.00E-10 | 53 | 1.9723 | 50.7472 |
| OBP16 | Unigene11783_All | 1082 | 345 | gi\|355389919\| putative odorant binding protein 23 [Nasonia vitripennis] | 54.7 | 9.00E-06 | 50 | 1.8368 | 26.665 |
| OBP17 | Unigene9264_All | 1298 | 447 | gi\|355390125\| putative odorant binding protein 84 [Nasonia vitripennis] | 182.6 | 4.00E-44 | 81 | 12.5494 | 19.8892 |
| OBP18 | Unigene3932_All | 704 | 402 | gi\|355389899\| putative odorant binding protein 13 [Nasonia vitripennis] | 156.4 | 1.00E-36 | 75 | 5.7987 | 8.1073 |
| OBP19 | CL3850.Contig1_All | 2174 | 465 | gi\|355390123\| putative odorant binding protein 83 [Nasonia vitripennis] | 151 | 2.00E-34 | 70 | 1.0151 | 6.4005 |
| OBP20 | CL4422.Contig1_All | 647 | 465 | gi\|355390121\| putative odorant binding protein 82 [Nasonia vitripennis] | 103.2 | 9.00E-21 | 58 | 6.7675 | 4.5339 |
| OBP21 | CL3211.Contig1_All | 1430 | 432 | gi\|355389921\| putative odorant binding protein 24 [Nasonia vitripennis] | 143.7 | 2.00E-32 | 65 | 0.7018 | 3.8458 |
| OBP22 | CL1687.Contig1_All | 1152 | 405 | gi\|355389989\| putative odorant binding protein 58 [Nasonia vitripennis] | 134 | 1.00E-29 | 64 | 1.1066 | 2.767 |
| OBP23 | CL3850.Contig1_All | 2174 | 465 | gi\|355389900\|putative odorant binding protein 14 [Nasonia vitripennis] | 151 | 2.00E-34 | 70 | 6.4005 | 1.0151 |
| OBP24 | CL3215.Contig1_All | 731 | 423 | gi\|355389889\| putative odorant binding protein 8 [Nasonia vitripennis] | 125.9 | 2.00E-27 | 65 | 0.2968 | 0.7188 |
| OBP25 | CL3211.Contig2_All | 1311 | 444 | gi\|355389917\| putative odorant binding protein 22 [Nasonia vitripennis] | 56.6 | 3.00E-06 | 68 | 0.0541 | 0.2494 |
