# Supplementary material for: Transcriptome and Expression Patterns of Chemosensory Genes in Antennae of the Parasitoid Wasp Chouioia cunea
Source: PLoS One. 2016 Feb 3;11(2):e0148159. doi: 10.1371/journal.pone.0148159 (PMC4739689; doi:10.1371/journal.pone.0148159)
Supplement: S3 Table — (DOC) [file pone.0148159.s008.doc]

**S3 Table.** The nucleotide sequences of 25 OBPs, 11 CSPs, 80 ORs, 10 IRs, 1 SNMPs and 17 GRs of *C.cunea* identified in present study.

>CcOBP1 [ORF 378 bp]

ATGAAGACCTTCGCTATTGTTCTTGCCGTCTGCCTTGCCGTCGTATATGCTGATGACCCACTCAAGGACATCCCTAAGGACCTCATCAAGACCTGCTTGACCGAAAATGGCTTCGATGCCGCCCAATACCCCAACGGTTTGAGAAACGTCAAGGTTCCCGACAACCAGGAAAAGAACAGGGACTGCTATTACGCTTGTATGATGAAGAAGATGAACCTGATGAAGCCCGACGGTATGCTCATGGAGGATAACCTTAAGAGCAAATTCAACTTGAACCTCGAGACTCTTCAGAAGGCATTGAACACTTGCAAAGCCCAGGTTAAGGGAAACGACAGCTGCAAACTGGCTGCTTGCCTCATGGCCAACCGAGGAATCTAA

>CcOBP2 [ORF 411 bp]

ATGAAGTGCTTCGCTATCGTCCTGGCTCTTTGCGTTGTTGGAGCTTACGCTGCAACGCTTAGCGACGAACAGAAAGCTAAGTTGAAAGGATTCAAAGAGGCTTGTATCACCGAAAGTGGCGTTAATGCAGACCTCGTCAATTCTATCATCAAGGGAGGAGAAATCAAGCGCGACAAAAATCTTGATTGTTTTTCTGCTTGCATGTTAAAGAAGATAGGAATTATGCGCGACGATGGAACCATCGACGTAGAAACAACTCGCGCCAAAGCCCGAACCACTAGCGTGGACGTAGCCAAGGCCGACAAGATTATCGACAAGTGCAAAGAACTTGTCGGCAAGGATGCCTGCGAGACTGGTGGTAACGTTTTTGGTTGTTTCATCTTGGGCAAGGACTTTCCTGTCTTGGATTAG

>CcOBP3 [ORF 453 bp]

ATGGAAAAAGTATCCAATATGACAACTTACACAGTAATTTGCATCTTGATACTAAGTGTACATATTATCAATGAGGCTCACGCCGGCGCGAGCAGAGAACAAATGGAAAAAATATCCGAAGGATTTCGAAAAACATGCATTGGCAAAACAGGAGCAGATTTAGCTATCGTACAAGAAATACGGAATGGAAACTTTATCGTGGATCCGTTGGCAAAATGTTACACCAAATGTATCATGGGTCTCATGAAAACTCTTACAAAACAAGGGCAGATAGATGCTGAAATGATGATAAAACAAATAAATATAATGGTAAGTCCAGATATTGCTGGGCATATGATAGCGGGCGTGAGAAAATGTCATGTCGAAGTAAGTGCCGATGAACCGTGCGAGCTCGCTTGGCTTTTCACTAAATGTGTCCACGATGAAAATCCCGAGCTCTTCTTTTTCCCTTGA

>CcOBP4 [ORF 390 bp] ATGAAGGCTTTTATTGTGTTGTTTGTTATTTGCTGTTACTGCAATTTTGCAGCTTACTCTCGCAACATCACGTTCACCAACGATGAACTCGACGAAAACATACAGATGTGTCTAGCCAAAACAAGACTGAGTCAAGCTTTTTTCAAAAGCGGAGATGAGAATTTGAAGTATCTAACCGAAGAACAAAAATCGTGTTTTTTGGCTTGCATGTTTAAGAAAGCTGGTATTATAACACACGATGGTTCCGTTAGATCAGTCACCGAGGAGATGGACATAGCCACTGCTAACGCAATCGATAGATGTCAATCGTTGGCTGAAGGAAATCTTTGCAAATTGGCTTGGTGTCTATACAAGACGGACAAGTTCAGCATCCCCGTCATCTCCGACTAG

>CcOBP5 [ORF 408 bp]

ATGCATTCCTTTACAGTAATCGTAGTGTTTTGCGCGCTTTTCACGAAAAATGCGCTAGCAGATTCTTATAATGTCGAGTTGACAGAAGAGGATAGAGAAATATTTAAAAAATGTATTAAAGAAGGAGGACTCACAAAGGAAGAGCTTAATGCTGCCGTGCGCAATTTCGACAAAAATGCTGACCGAAAAGTAAAGTGTTTTAGAGGCTGTCTTCTTAGATCTCACAAAGTCATAAAAGATGACAATACAATTGATGGCAATGCAGCGGTTAATTATTATCACGTCGAAGACGTCGAGCCTCTGAAGAAGCTCATTTTAAAATGTAGTGCAAGTACAACTGGAAATGATTATTGTGACGTAGCTCAAAGTGTGGAATCTTGCTTTCGTGCATCTGATATTGAAAATTAA

>CcOBP6 [ORF 423 bp]

ATGATCTCGATGTCAGCAGCACCAAGTCCGCTCTCTTTGCTGCAATCGTCTTCGTATTCGTACAGAGATATTTCAATGTCAGCCGAAAAACTGGCCTATATAATATCGATCTTTTTGGCATTTCTTGAAAAGGGCTCTACATGTGTACCTATACATACACGTGTACGTCTATGTACTTACCAATGTCGTAGAGACGCTCACGAAAAGAAGGAACATCGAGAGAAGTGTCATTTTCGTCGTTACAGTTTGCAGCGACCAACGTGTTTATTGAAACAAGTGCCTAGACGAATGGATTATTTGCAACTACCAAGACGAAATGAAAATCGAGACGTATTTATAGATGCCGACTGTTATGAAACTCAACTGCAAAGTGTGAAAAATTTGTGGCTTTTTTTTATTGCGAGAGCCACCGATGTACATTGA

>CcOBP7 [ORF 402 bp]

ATGAAATTCGTTATTTTCAGTTTTTTCGCGCTTTTCCTTGCAGTACGAGATATCCACGGAAACGACGAATCCATACAAAACCTCAAGGAGTGTTTAAACGAAAATAACATCAAAGTTGATATTGAAAAATTGAAGGAGCACGATGACCCTAAGATTCGTTGCATTTTGGCATGTGTTATGGAAAAAGAAGGAATTCTGGAAAATGGCGACATACAATACGATCTATTGAAGGAAGATCTCTTGGAGGATGCTGAGCAGCTGGGCGAAAAGAAAATATCCGAAATAGTGGATTACTGTGGAAATTTAGCGAAAGAATTGACAGATGTCTGCGACAAAACAAATCTTATTGGAATGTGCCTCGAGGAAGAACTTGAAAAATATAATATTAAGTTTGAAAAATAA

>CcOBP8 [ORF 459 bp]

ATGCACTTCTGTTCACTCATCTTTTTTCACTTGGCCTTGCTGCTACTCCTCTTCGGCTTAACTAATGCGAAACCTGGTACGCGGCCCAGCTTTGTATCAGACAAGATGATTGCTACGGCTGCCAGTGTCGTAAATGCCTGCCAAACGCAGACGGGCGTGACCACCGCCGACATCGAGTTGGTGAGAAACGGTCAGTGGCCCGATTCTATGGAATTAAAGTGTTACATGTACTGTCTTTGGGAGCAATTCGGATTAATCGATGAAAAACGGGAGCTAAGTTTGAACGGCATGTTGACGTTTTTCCAACGAATACCAGCCTATCGAAACGAAGTCCAACAAGCAATTAGCGAGTGCAAGGCTTTGGGTAAATACTTTGCCACCGGTGATAGCTGCGAATACGCGTATACATTTAATAAATGCTACGCCGACAGATCACCAAGAACTTACTACCTCTTTTAA

>CcOBP9 [ORF 405 bp]

ATGAAATCCTTCGTGATCGTTTTTGCCTTGTGCATCGCCGGTGCTTTTGCCGGGCTTACCGATCAACAAATAGTTAAGCTCAGAGAATACAAAATTGGATGTCTTGCAGAAACTGGCGTGTCCGAAGATGTGGTTAACAAGCTTAAAGTGGGAGAGGCCGTAGTGTTCGACGAAAAATTGAACTGTTTCTCCGCTTGCATATTGAAGAAAGTTGGAATCATGAGACCAGACGGTAGCATTGACGAACAGGTAGCTCGCGACAAACTGCCCAAGGATTGGCCCCAAGACAAAGTTGATCACGTCGTCAACGCTTGCAAAGTTCAAGTTGGAAAGGACAGCTGCGAAACCGGTGGAAAAGTCTTGGGCTGTCTTGCAAAAACCAGAGCTATCGCCCTCGTTAACTAA

>CcOBP10 [ORF 414 bp]

ATGAAAACTGTAATAGTCAGCTTTTTATTTTGCATCGCCACTGTACTGGCTGAACTAACAGAAGAACAGAAGGAAATGATAAAACCGTACAAAGATGCATGTTTAGCCGAACTAAAATTGGACGAAGCTATTATTGAACAGTCGAAAAAAGAATACCTAGAGCAAGGTAAAACGGAATTTAGCGATCAACTTAACTGTTTTTCTGCTTGCATGTTTAAGAAGGTTGGAATCATGACAGAAGAAGGTAAATTCGATGAAGATATGGCTCGTGCTTTGGCTTCTGGTCAATTTCCTGAAGACGAAATAAACAAAGCTATTAATGCGTGCAAGAACGAAGTTGGAAAAGATATCTGTGAGACCGCAGGAATACTTTTCGAGTGCTTTTTGAAACAGAGAATTAGTGTGAGTTCATAA

>CcOBP11 [ORF 429 bp]

ATGAAACTTTTATTACTTACTTGTAGTTATCTCATCATTTGTATGAAAATGAGCACATCTGCGCCCAATCACGAAAGAGTAGACGATGTTGATTTGAACGATAGCATCGATCAATGCGCAGCACAAATTGGATTACTCATTGAGGAAACTAGAAGAAGTTTTACAATGCCTATAGAAGCTCCTGGGAATTGTGTGATGGCATGCGTTTGGGATAAAATTGGACTGATGGATATCGATGGAAAAATTATCAAAGAGGAAATGATTACGAGCATTCATCCCACGTTGGAATTATTGCCAAATATCACCAGAGTTACCGAAGATGATTTCTATCAATGCGTTGACGAAGCAAATCGATTCGGCGATCCTTGCACAGTGATATCCGAGTATTTTAAATGTTTAATCAAGGATCTATTTATGCACAATACATAG

>CcOBP12 [ORF 414 bp]

ATGAAGAACGTAGTTGTTTGTTTCATCGTGATAGTTTTTGGAGCTATTAACATTAATGCTGGTGAAATACCGAAAGAAATTGCACATATGGTCGCAGACGTTCGTGAAAAATGTCATCGCGAAACCGGAGTCGACATCGAGCATGTGGACAGAACGGCAGAAGGTTATTTCCATCCAACCGAAACTCTTGGATGTTATTTCTCTTGCATTTTCGGTCATTTCGACTTGCTCGATCATAATGGGCACATCGATTTCGACAAAATTATTCCAAAAATCCCTGAATCGTTCAAAGATCATGGTATGGAAATGATTACCGCTTGCCGTCACTTGACCGGAAAAAATCCTTGCGATATGGCATTTAACGTCGTGCAGTGCTTCCAGAAGACTAACCCCGAGAAATACTTTGTCATTTAG

>CcOBP13 [ORF 447 bp]

ATGGACAGCGGCGCCAGCACCAGTTACTCCTATTGCGCCCTCGTGCTATTGTTGTGCATGCAGGCGATCCTCTTGGTCCACGCCGGGCCGCCCGATTGGATCAGCCCCGAGATCCTGGAGATGGTACAGTCCGACAAGGCGAGATGCATGGGCGAGCACGGTACCACCGAGGCTCTGATCGAAGAAGTCAATCAAGGTCATCTCACCGACGACAGAGCCATCACCTGTTACATGTACTGCCTCTTCGAGGCCTTCAGCTTGGTGGACGAGGACGGTGAACTCGAGGTTGAGATGTTGGTTGGATTCCTGCCGGAGCACATGCAAGGAGTCGCCAACGAGCTCATCGACGCGTGCGCCAAGGAGCCCGGTACCGACGTTTGCAACAAGATGTACGCGGTCGCCAAGTGCGTCCAACAAAAACGTCCCGACCTCTGGTTCATGCTGTAA

>CcOBP14 [ORF 399 bp]

ATGAAAATTTCAGTTGCTCTGTTTGTGGTTATCTGCATTGTTGGCGTTTATTCGCATCCACATGGAGAGCACGGCCATAAGCTGACTCCGGAGCAGATTGCTCGAATCATGGCAGACGTCGAAGAATGTGCTCGTACCAACGACATTGGTCATGAAGTGTTCGAAGACTTGAAAGCTGGAAAGAATCCTACTCCAAGCAGAAATTTAAGTTGTTTTTCAGCTTGCGTACTCAAGCGTAACGGAGTAATGAATGCCGATGGATCTACCAATCACAAACCTACCGACTCGGATGTTGCCAAGGAATGCAAAGATCTGAGAGGCGACGACGACTGCGAAACTGCGGGTAAAATCGTGTCATGTCTCCACAAAAACAATCTTATCCTAAAAATCTCCGAATAA

>CcOBP15 [ORF 375 bp]

ATGAAACTGTTACTCGCACTTTGTGTAGCAATCTGCTTTGCTGGCGTCTACTGTGATACGGCTCCCAAGCCCGAGGAAGTACAATATGTCAAGGATTGCGCTAGCAAGAATAATATGGATCAAAAAATGATCGATGATCTAAAGATGCAAAAAACATTTTTTGCTAGTCAAGCTGCAATGTGCTTTACACACTGTGTAATGCATCATAATGGAATGCTGGATGATGAAGGAAACATGACTGATATGTTTAAAAAGATACCTGAAGCTGCCGAATGTCAATCAATGACTGGAAATGATAAGTGTGAGACTGCCGCTAAAATTATGGATTGTATGGTAAAAAAAGAAAACGCTGATATGAAGGATTTAAATTTATAA

>CcOBP16 [ORF 345 bp]

ATGAAAGTTCTTCTCGTTCTTGTTTGCTGCTTGGCAGTAGCAATGGCTCAATTCTCTTCGGACAAACAAAGGGCAAGTGCCATGAATGAATGTCAAGAAGAGTTGAAGGTGCCCGACAGCGAAGTTGAAGATCCATCAAAGTTAGGATGCTTGTATGCGTGTATGCATAAAAAAGTCGGCTACACTGATGCTGATGGAACTTACAACTTGAGAAAACTAGCTGGTTCAGCGTACAATCAACGTTTTGAAGAAGCTGCACAAAGAGTAATGAACATGTGTGCGGAACAAGCAAAGGGAGATCCCTGCAAAATGGCGCTATGTTTAGAAACTACCAAGGAATTCTGA

>CcOBP17 [ORF 447 bp]

ATGAAAGTTTATATAGTGATAACTATTTTACTGGTTTGCGCAACACAATTCAAAATAATCGAGTGCGGAAAAAAAATGGATATCGATGGACTGAAGGATATGTTAAAACCAATGAGTAAATCGTGCAAAACGAAAACTGGCGTTTCGGATGAATTAATCGCTGGTACGGCAAATGGAATCTGGCCAAGAGAAAGATCTCTTATGTGCTATTTTAAGTGTTTAGCAGTGATGTTAAAAGCTATGAACAAACAAGGAGAGATAACGCTACGTGAAATCAATAGACAACTTAATATATTAGTAATAGACGAACTTGTGCCACGAATGAAACAAATTTTAGAACAGTGCCTGGCCACAGCAACACCATCCGATGATGCGTGCGAGTACGCTTTTAATCTGATAGTATGTGGCTACAAGGCAGACCCAACGTTGTACTTTCTACCATCATAG

>CcOBP18 [OPF 402 bp]

ATGCAACTCTTCGACGTCGCTTGCATCCTCGGTATCGTTGTTATCATCAATGCATTATCCAACGAGCAACGTTATGACAACTACATAGCGGTGTTGAAAAATTGCTTAAAGGAACTAGGACTGTCCGAAGAGGTTTATGCATACGCATCCGTTATCAACAACACAGACGGTGCATACGACAAAGCAAAATGCGCGGATTTGTGCATGTTCAAAGCACTCAAAATAATGAAACCTGATGGGCATATTGATCTTGAGAAAGCTTTGGAACATTTGTTGTCCGGGGAGCCAGGAGTTCAACGTGACATCATGAAGACTAATATCGAAACGTGCAGTAAGAAAAAAGAAGACAACGATTGCGATACTGCTCATAATATGATGACGTGCGCAGTAGGGACCTACTGA

>CcOBP19 [ORF 465 bp]

ATGCGTCTGATCCACGCGATAATAACTTTAGTTGTAATCGTAAATATATGCAAGATTGCCGAGTCCAAAATGACTATTGAACAAATTAAAAATACCTTGAAACCATACAAATCCAAATGTCTGAGAGTCACAGGTGCGGATCCAGAGCTAGTCGAAGGGACTAAAACTGGGGCTTGGCCTAAAGATCGAACTCTGATGTGTTTTACGAAATGTATTTATAACATGATGAAAGTTATAAAAAATGACGATATTTCACTAAAGGCTGTTTTCTCACAAATAGACTTGATGGTGCCGGATGAATACGTCGCACAAATGAAAGAAATAGTCGAAAAGTGTTTGGAAATGGCTAATTCTAAAGCAACCGATCTTTGCGATAAAACTTGGTTGCTATCAAAATGCTTCTACGAGGCAGACAGTTCGGTACGTATAGAACTTTATTCTGGAAATGTGTATGCTCTGAAATAA

>CcOBP20 [ORF 465 bp]

ATGAAGCGCGCATTTTCCGTTCTTTGCGTCTTTCTTATACTGGGCTATGCCTATTCGATCGATCTTCAATTCGTATCGCAAATGAAGGAGTGTGGAAGCGAAATGGGATTTTCACCTGAGCAAGTGATGGAGATGATGGCGAAAAATGACGGTCAAGTAGGTTGCCTTCGTGCATGTGTGCTGGAAAAACTTGGCGCTTTGCAAAACGGGAATTTGGATAAAAACGTTTTAGCATCACTTTTGGAACAAAATAAAGATACAATACCTAATTACGAACAAATACGTGCAAATCTTGACACGTGTTATGGCGAAGTCACAAGTGGAGGATTGACCGATCAGTGTCAAATAGGAGGAAAATTCAGTACGTGTATGCAGGAACATATGACAGGTATTGCTGGTTTAGCCATGAATGGAGCTTTTCAATTAAGAAATGTAGCACAGAATCTCAATAAGTTTGGTCTTTGA

>CcOBP21 [ORF 432 bp]

ATGAACGTTGTCGCTGCAATCGTTTTCACTTTGTGCTCTGTTGGAGCTTTTGCGGGATTGATCGAAGGAGACGTGTCGCCGCCAAAACCAAACATTACGAAAGAATGTCTGAAAGAGTATGGAATCGATATTGAACAAACCAACGGTGCACCGCTTAGCGATGAAGAAATATACTGCATTCCCGCTTGTGCATATAAAGACTACGGAATAATGAGGCCAGACGGAACGATCGATTCGGATAAAGCGGAGTCTTACTTTGGAGTTAACGATCATGAGGAGAGAAGTATATTCTTCTCGGTTTACGAAGTTTGTCGTGAAGGTAAAACGCATTGTAAATTGGTCCAATGTATGTTCGACAACTTGAAAAATCATTGGAAAAGCTCATCAAAGTCAAAGAAAGTTACGAGGCAACTTTTTTCTCAGCTCGTTTAG

>CcOBP22 [ORF 405 bp]

ATGAAACTCCCCGTTTGCTTGATTTTCTCGGTGTTCTGCTTTTCATCGGCTCACGCTTTATTGACGAAAGAAGCTATAGAGTCGTTGCGAACCCACCAAAAGTATTGCGTTCGAACGAGCGGCGTGTCCGAGGATCACGTGGAAATGGCGCGGTTGGATCGTCAAATCCACGAGGACGAGTATCAAGAAAAGTTTGCCGTTTGTATGCTGAACAAATTCAATATCATGAACACCGATGGATCCATCAACAAAGATGAGATATCGTACGTGTTACTTACGGACAATCCATGGTCGTACCAAACCGCCAAAGACTGCACGGCTCTCGTTGGCTCAAACGTTCGCGAGACCGCGAGGAAAATAACCAATTGTCTTCTACAAACGGACATAATCGTCATAGCGCCATGA

>CcOBP23 [ORF 465 bp]

ATGCGTCTGATCCACGCGATAATAACTTTAGTTGTAATCGTAAATATATGCAAGATTGCCGAGTCCAAAATGACTATTGAACAAATTAAAAATACCTTGAAACCATACAAATCCAAATGTCTGAGAGTCACAGGTGCGGATCCAGAGCTAGTCGAAGGGACTAAAACTGGGGCTTGGCCTAAAGATCGAACTCTGATGTGTTTTACGAAATGTATTTATAACATGATGAAAGTTATAAAAAATGACGATATTTCACTAAAGGCTGTTTTCTCACAAATAGACTTGATGGTGCCGGATGAATACGTCGCACAAATGAAAGAAATAGTCGAAAAGTGTTTGGAAATGGCTAATTCTAAAGCAACCGATCTTTGCGATAAAACTTGGTTGCTATCAAAATGCTTCTACGAGGCAGACAGTTCGGTACGTATAGAACTTTATTCTGGAAATGTGTATGCTCTGAAATAA

>CcOBP24 [ORF 423 bp]

ATGATGAATGTTAGGTGTATTTTCGTGATTCTTGTTATAGCCGGATGCGTTTATGGCGACTTGTCGGAGGATCATAGAGAAGCACGTAAACAAAGACTGGACAAATGTAGAAAAGAAATGGGGATAACCGAAGAAAATCCATTATCCAGACCTCCAAATCTGGATGACCCTAAGGAGAAATGCTTTTATGCTTGCTTAATGAAAGAATCTGGAAAACTAGTGGACGGCAAAATGGTCGCTGAAAAAGTTCTCAGCGCTGAGAAAAAACGTAGGCCTAATTATAACGATGATATTGAGGCAAAATTAACCTACTGTGTGGAAACAGCAAACGAACAATCTGACGAATGCGAGATGGCCGCTACAATGAAGAAATGTACGTTTGAAAAACTTGGTCCTCTTCCTCCTCGGAAACGTCAACAATGA

>CcOBP25 [ORF 444 bp]

ATGATTTTTCAAGTTGTCGAACATACATTGGACCAATTTACAATGCGTTTTACCTTCACGACAAACTTCGTAAACCGAGAAGAATATACTTCTCTCCTCATGATCGTTAACTCCAAAGTAAGACTCCGCTTTATCCGAATCGATCGTTCCGTCTGGCCTCATTATTCCGTAGTCTTTATATGCACAAGCGGGAATGCAGTATATTTCTTCATCGCTAAGCGGTGCACCGTTGGTTTGTTCAATATCGATTCCATACTCTTTCAGACATTCTTTCGTAATGTTTGGTTTTGGCGGCGACACGTCTCCTTCGATCAATCCCGCAAAAGCTCCAACAGAGCACAAAGTGAAAACGATTGCAGCGACAACGTTCATGGCGCGTGGAGTATTAGTTACGAGCACGTACGTACACACTTTGATCGAACATCTTCGTTTTCGACATGTTGA

>CcCSP1 [partial 348 bp]

ATCGTTGTGGCCACGGCCCTCTTGGTACTCGTTGCCGGTGTCGTTAGAGCCGAGGACAAAAAATATGATTCGAAATACGACAATCTTGACGTCGAGGCGATCCTTCAAAACGATGCGGAACGTAACATTTATTACGCTTGCTTCATGGATACTGGACCATGCCCCAATGAGGCTGCCATCTTTTTCAAGGGCCACGCACCAGAGGCTGTAGTTACGTCTTGCAGATATTGCACTCAAAAACAATTAGAGATGTTTGAAAAAATAGTAAGTTGGTTTGTCGATAATAGTCCTCAGGAATGGAACGCATTAATTGAGAAGACTATCAATGATGCCAGAAAACAAGGACTC

>CcCSP2 [partial 312 bp]

GTGATACTTTTCGTGTTTTTGGCCTTTTACGCCGTCGCTGCTGAACAGCTATATTCAGACCAATATGATTACGTCGACGTTAGTAAAATTTTGTCAGACGACGCACTCAGAGAAGAATATTATAACTGTTATATGGGAACGTCACCTTGTCTGACGGCGGACGCTCAGTACTTCAAAGAAATTCTGCCAGAGGCAGCTTTAACTAAATGTGTAAAATGTACCGACAAGCAGAAAGATAATTTCCAAAAAATAGCTACTTGGTTTACAAAAAATCAGCCAGAGAAATGGGATGCTTACACGAAGAAAGCAGTC

>CcCSP3 [ORF 471 bp]

ATGTCGCGTCCAGAAATAAATTTGGAATTTGTAGTTTCAATTACACTATTGGCTCTTTGCATTGCTCGTGCTGCAGATGAAGACAATGTACCACTAAAGAAACCAATAGATATGGCAGAACTTTTTGACAAGAATGCTATGAAAGATCACTATCCTATCGCTTGGACTGAAGTAAATACTAAAACTATCATAGATAATGACCGTCTATTCAAGAAGTATAAAGAGTGTCTGACCAACGAACATCCCGTTAGCTGCCCACGAATGGTGATGGAATTCAAAAAGTTAATACCAGAAATGATCGATACTTTGTGCGCCAAGTGTTTGCCTATTCATATCGAAAAGTTCAAAGAAGCGGTCGAATACATTTGCCACCGCCGCAGAGCTGAGTATGATCAGGTACGACGGGAAAAGGACCCTGATGGTGCCATACAAAAAAAATTTGAAGAGCAGTTTGGCAAAGTCAACTGTTAA

>CcCSP4 [partial 843 bp]

ACATCGTCGCGCCTTCAGTTGCTTCAGGCAAGGTTCCAACAAAAGCAACTACAGGAGAAGGAGCAAAAACTCCTACAGTTGTACGATCAGCAGCAGCAACGAGCGCATCAAGTCGCACAAAGAGGTAGCGCCGGCTCTAATGGCTCCAATCACAGTATCACCGCTAACGCCAATAAAGTGAAACAATTATTCGCCGAAGAGAGACGACAACAGAACGGTGTTAAAGGTATCGACAAGAGTTATCCTCTCGAACCTTTGAAAAGCAAAAAAACATCGCCAATCAATAAAACAACCAACGCCAAATCAACTTCCACCAGTACCATCATCGCCAATATCAATAATAACAATAACAACATGATAAATCGAAAGTCAAATTCAAACGTCGCTACGAAATGTGTGATAAAGGCAGACGCGAACAGCAACGTAAGTAACAGCCGAAGTATCAACGTACAGCAACAGCAGCACGAGAGAGAGAACAACTTTGCCTCGAGCAGTCGACAAGAGAACTTGTCTCGCGAATCTTACGGGAACTCTATTCGCTATAACGAAGCGGCCAACAGCAGCGAATCGATTAGGCAGGTCAACGGACACACTGCAACGAAGAACGGTTATCACTACGAGATAAACATCGATGAGGTGATCGATAACGAAGCACTGCAGCGCAACCGCATGCTAGCGAAATTTCAACCAGGCGACATCGAGAGGAGACGTCGTCAATTGAGTGCCGACATCATCGACGACGACGACGACAACGACGAACGATAAATTGTCTCGTTGTTACGGCGCATTCGTAGCACGCGATACAAGCTCGCTCTAACGAGCTCTGCTCTTTCGTACGTCACG

>CcCSP5 [ORF 465 bp]

ATGTTACGTCGCGGAGTTTACCACGCGTTAGCCATAGCTACAATGTTTCTAGCAGGTGTCGTCGTTGCTCAAGATACCAGCGAAGAGTCCACGGAGATTACTACAGAATTGCCGTTCCAAAAGAACAACAGGACCAACGAATTTTATCCAATTTCGTGGACTAAATACAACTACAAATTTATCGTTGATAACGAGAGATTATTTCGAAAGTATAAACAGTGCTTACTCGTGGACAAAACAACAGGATGTGCTCACGATGTTCTTCAATTGAAAAAAATTATACCCGAAGTGTTGGAATCAATGTGTGCCAAATGCTTACCAGTTCACGTGGAGAGATTCAAAGAAATAGTTGAATACGTTTGCAAAAAGCGAAGGGCCGACTACGATGAGGTACGAAAAGCAAAGGATCCAGCAGGATTACTTCAGAAAAAATTCGAAGATAAATTTGGCAAAGTCAATTGTTGA

>CcCSP6 [partial 492 bp]

ATGGACAAGCGGATGTGCTGGCTGGCACTTTGTTGGCTGCTTGGCGGCTGCCTTGATAAGCCAATAAATAACGAGGCAATAGTCAATGGTTACCCCTGGCCTGAACCTGGTACCTACATGACTCGATGGGACAAAATTGACCTCAACGAACTTTTCAAGAGCAAACGACTTATGAGGCATTACTTTAATTGTCTTGTCAACAAGGGGCCATGTCCACCGGACGGTCGCGAGCTTAAACGCGCTCTTCCCGAAGCTTTGGAAAATGGTTGCGCTAAATGCTCTAAAAGCCAGTTGGAAAGTGCCATCAAAATTATTCGCTATCTCAGAGAATTCGAACCAGTGAAATTTGAAATTCTTGCCAACAAATTCGACCCAAAAGGAATCTATCGAAAACGATATCTGGATCCTACTCCAGATGAGACGAATAACAGTATAACTGACGAAAATTCAGTTGACGAAAATGACCAAAAACTCAAGCGATTAATCAAAAGA

>CcCSP7 [partial 303 bp]

GTCGTTGTCCTGATGCTACTGCTGCTGCTTGCAATCGTCGCTTCTGCTCAAGACGTCAATATCTTACTGCAAAACAAAAATCTCGTTAGTCGAGAAATCGGTTGTGTGTTACAGCGCAACCCTTGTGACGTCATCGGCAAACAAATTAGAGGTTTATTACCAGAAGCTTTGAACAACGGCTGCGGTCGGTGTACTCCTCAGCAAGCGACCAATGCCAAGAAGCTCATTGCCTACATGAAGAAGAATTATCCAAACGAATGGGTTATGATCGCACAGATGTATGGCCGAGCAAAAGCTGTTTAC

>CcCSP8 [ORF 144 bp]

ATGCTCGTGCCTTCAAGAGACTACTACCAGAGTTCGTGTCTACGAGTTGTGGGAGATGTTCAAGAAGACAGAAGCAACTCTCTTGCAAGGTCATCTTCACGTTACAGCAGAAGAAATATAGTGATCTTTGGGACGATTTTTTAA

>CcCSP9 [ORF 375 bp]

ATGAAACAACTTTGCACTTTGGTATTTTGTTGCGTAGCTCTGCTACTGGCTGTAAACGCTGCTGAATATAACAGCAAATACGACAACGTTGACGTTGATAGGATCTTGCAAAACGGACGAGTTCTCACCAATTACATCAAATGTATGCTCGATGAAGGCAATTGCACCCCTGATGGCCGTGAACTCAAAAAAACACTTCCAGATGCACTTGCCACTGGCTGCATTAAATGTAACGAAAAACAGAAGGCTACTGCCGACAAAATCATTAATCATCTAATGAAGAGACGCCCAGCCGATTGGGAAAAATTACTCCGAAAATACGATCCTAAGGGTGAATTCAAAAAGCGATACGAAGCACAAGGAAGGAAAATATAA

>CcCSP10 [partial 351 bp]

ATGGCTACCAAATTGGTATTCGTCCTAGCGATTTGTGCCCTCGCGGCAGTAGTCTGTGCCAAAGAACTCTACTCGGACAAATACGATAACATCAATATCGATGCTATCCTAGCAAATGATAGTGTTAGAAACGAGTATTACAATTGTATGCTTGATTTTGGTCCTTGCGTCACTCCCGATGCTGCATACTTCAAAGGATTATTAGGTGAAATTATAATAACAAACTGCAGAAAATGTACAGATAAACAGAGATACATGTTTAAGCAAGTTTTAAAACATTACACGTTAAAAGAGCCACAAAAGTGGCAAGAATTAGTTTTAAAAGTCCTAAAGGAACTTCCAAAATTGAAG

>CcCSP11 [ORF 396 bp]

ATGCATTCGTACTATTCTTCTCGTTCTACACGGCGAGCGTATACTTTGAATGGATTTTACGAGCGATTGTTTTTGTTGGAAATGTTTTTACGAGTGTGTAATCTGGTATACTTTATTGCAGGCGCTCTTCCCGAAGCTTTGGAAAATGGTTGCGCTAAATGCTCTAAAAGCCAGTTGGAAAGTGCCATCAAAATTATTCGCTATCTCAGAGAATTCGAACCAGTGAAATTTGAAATTCTTGCCAACAAATTCGACCCAAAAGGAATCTATCGAAAACGATATCTGGATCCTACTCCAGATGAGACGAATAACAGTATAACTGACGAAAATTCAGTTGACGAAAATGACCAAAAACTCAAGCGATTAATCAAAAGACACCGTTCAACTATTGCTTAA

>CcOrco [ORF 1428 bp]

ATGATGAAAATGAAGCAAGTCGGCCTGGTGGCCGATCTGATGCCTAACATCAGGATCACGCAAGCCGTTGGTCACTGGCTCTTCAATTATTACAGTGAAGGAATGAGATTTCCTCATAAAATTTACTGTATGGTTACCCTATTCCTAATGCTGTTCCAATTTGGCACAATGGCACTTAATTTGGTGAAGGAGTCCGACGACGTCGATCAATTGACGGCAAATACAATCACGGTGCTGTTCTTCATGCACCCGATCGTGAAGGTAGTCTACCTTGCGGCGCGCGCCAAGATATTCTACAAATGCCTCGGCGTCTGGAACAATCCTAACAGTCATCCTTTATTCGCTGAGAGTAACCAACGTTACCATGCCCTTGCTTTATCTAAAATGAGGAAGCTTTTGTTCTGCGTTTGCGGTGCGGTCACGTTCTCCGTTATCTGTTGGACAGGTATTACTTTCTTCGATGACGCTGTCAGGAAAATCCACGACAAGGAAACCAATGAGACTACCATTATACCACTCCCAAGACTGATGATCCGTTCGGCATATCCATGGAACGCAATGAGTGGCGCAGCACACATTTTCTCGATGATCTATCAGTTCTATTATCTTGTCATTACAATGGGCATCTGCAACATGTTTGACGTACTTTTCTGCTCGTTTCTACTATTCGCCTGCGAGCAGCTTCAACATCTTAAGGCAATCATGAAGCCACTCATGGAGCTCAGCGCAACATTGGACACCGTTGTACCTAACAGCGGAGAGCTCTTCAAAGCTGGAAGCGCAGACCACCTCAGGGAATCGTCGGGCATTCAACCAAGCAGTAACGGTGAGAACGTGCTCGATGTCGACGTTCGCGGTATATACAGCAATCGCCAAGACTTTACCGCGACCTTCAGACCGACAGCGGGTACGACATTCAACGGAGGTGTTGGTCCAAATGGTTTAACGAAGAAACAAGAGATGTTAGTCAGGAGTGCTATCAAATATTGGGTTGAGAGGCATAAGCACGTCGTGAGGCTGGTAACGGTCGTTGGTGACGCTTATGGAGTTGCCCTTCTGCTCCACATGTTAACGACTACTATTACCTTGACTCTACTCGCTTATCAAGCCACCAAAGTGAACGGAGTAAATGTTTATGCGGCAACGACTATCGGTTACTTGCTTTACACCCTGGGTCAGGTGTTTTTGTTCTGCGTGTTTGGAAATCGCCTTATTGAGGAGAGCTCATCTGTTATGGAAGCCGCATATTCTTGTCACTGGTACGATGGTTCAGAGGAGGCAAAAACTTTCGTGCAAATCGTGTGTCAGCAGTGTCAGAAAGCTATGTCTATATCTGGTGCGAAGTTCTTCACGGTATCACTCGATCTATTCGCCTCGGTGTTGGGTGCCGTGGTAACATACTTCATGGTGTTGGTGCAGCTGAAGTAA

>CcOR1 [OPF 1257 bp]

ATGGAGGAAACGTCGAGCTTTTACCAACGAGTACGTCGAGTACAAACGCGCGTCTTACGTTTCGCTGGTTTAATACCGTTGGAAGGTCGTACGCATTATTTTATCGGCACGATAATCATGTCGTGTTACGTAAATTTCGCGTTCGCTGCCGTTAGTAGCGTTTACGTATGGGCTTTCGTCGAAGATTGCATCAACGCGAGGTTTAATCCGGACATCACGTCCGAATTATTTTCGTTCGTTGGTTTTCACTTCAGATTCATGTACATATTTAGCAGGAGAAAGAAACTTACGAAGATGCTCAGATTCGTAGAGGACTTGTGGAAAGGAGTAGAGAGCGAGGAAAAAGTACACGTGCGCAAATTCGTACGCAAAGTAAGTAAATTGAGCTGCTGTTACTCGGGCATCATCCTTACGACGATCACGTTGTACGTCCTATCGAGCCAATTACCACAACTGACGGCTGAATCGTCCAACGAGACGTTACATCGCGTTTTGCCTTATCCGTTTTACTTTGACGTTCAGAGTTCACCAAAGTACGAAGTACTTCTGGCGATCCAGATAATTTGCCTGTTAACCGTCACTCAAACCAGCGTTTGCGTCGACACGTCGATTGCCTTTCTGATAATGATCGCATGCGGACATTTTCGATTGATTCAAGTACGTTTGTCCCTTGTTGCACGCGACATCGAGGATAACGAGGAAGAACGACGTCGCCCAATTGCCGCTAAAGACGAGATTCGCAACAACGAGGAAGGCGTGACGGGTGTCTTCAATGTTCAAACGACCGACGAAGGGATTCGCAAGAGGATTCGAGACTGCGTTGCTTATCACAGCGAGATACTCGAATTTTGCGACGACATTTACGCTCTATCGAGCGAAATATTCATGATTGAGCTAATAAGTACGACCTACAACTTGTCTCTCATTGGCATTCTCTTAGCCGGGAACATGCCCTTAGCCGAGAAATTCAAATTTGCCCCGGTACTTTTGATTCTTACTACTCAACTGTTCGTCTGTCAGTACCCTCCGGATCTTCTTCTCCAAGAGAGCGTTGGAGTAGCCGATGCAGCTTACATGATACCACCGTTTCGAAACGATCGTCTACGCGTTGATCGGCTCTTGCTAACGCTACTTACCAGATCACAAATTCCTTATCAGCTGCTTGCCGGTGGCCAAATCAAATTGTCCATCGAGTCGTTTGGAAATATGATAAGGGGAGCTGTATCGTTCTTTACGGTTCTCAGAAACTTCAATTAG

>CcOR2 [ORF 1227bp]

ATGGGTATATTTTCAAGCTCGGATCAGTCAGGCTTTGACGTAGCTATCGGCCCATGCCGGGCTGTTTTAAACTTTCTTGGCGCTTGGGTAGATCCGGTGGAGCCACAAACGACATTTGCTTTGTTTCGGTTCTTTTTGTCTACATCCACCATGATTATATTCTCGTGTCTCGTACAAACAATCGAAGTTTTCAGAGGCTGGGGCGACTTGAATTACGTGATTGAAATACTAATTATCGACGATATTCCAATATGCGTGGCTGTCATGAAATTCATTGTGTCCTTCTATAACAAAGATATTTTAAAAAGGCTAGTAGTATTGATGGCGAAAGACTGGCAGAGTAAGTATGCGGACGAAGATTGGAAAATAATGTGGGAGACAGCACAATTCAGTCGAAAGCTTTCGCTTGTTTGCATTCTATTGGCTGAAGGAACAATCACGGCGCAGTGTATGGTAGTCCTGATGTTTGATTTGTACAGCAAAGATAAAAAGGAAAGGCCACTCTACATGCATTCTTACTTTCCCTTCAACACGCAAACTAGTCCAAATTACGAGTTTACTTGGTTTGGTCAGTTTATGTGTACCGTTTATGCGGCAAGTGCTTTTTCGTCGGTTGACGCATTCTTTGGTGTTCTCGTGTTACATCTTTGCGGACAGTTGTCGGTATTGAAGAGAAATTTGAAGGTGCTCACACATAATGATAAGGAGAACCTCGATTCCGACGAGTTCACTAGAAGGATTGCGGCACTCGTTGATCGCCACGACCATCTCAATATGTTTGCGAAAACGTTAGAGGATTCGTTCAATTTAATGTTTCTACCGCAAATGATAGGCACGTCTCTTGCTATGTGTTTGCAAGGATACCAGTTGGTAGTGATATCTACTGGTTCCGAAGAAGGCCTGCCTTTATTACAAATCTTTCATATGGTTTATTTCACCACTTGCTTTTCGTTCAGTCTATTCGTGTATTGCTTCGTAGCCGAACGTCTTCAGTTTGAAAGCACGGAAATTGATTATGCAGCCTATCAAAGTGAATGGTACAATTGGGCTCCAAGGGATACGCGCCTTCTTCTTTTATTAATGGGTCGAGCTCGCAAACCATTAGAAATTACAGCTGGAAAATTTTGCGTTTTTTCACTGGGACTTTATTGCAGTATATTAAAAACATGTGGAGGATATGTTTCGATGCTTTTGGCAGTAAGAAGACGTCTAGTGCAAAATCAGTAA

>CcOR3[ORF 1185 bp]

ATGACGCAACGACAACAAGCTACCTTCGACGATTACACGTTTCTCAATCGATGGGGATTGACGTTTCTTGGTATCTGGAGAAACGAGAGTAAAGCCGATCGGTCGTTTCGCAAGGTTCTACATAACCTTCACGTACTCGTTCTTTTTGGGCTGATGACGCTTTTGCTTATTCCTCAATGGCTCGATCTTTACGTTTTTTGGGGAAATATCGACGCCAACGCCGAGACTTTTGTTCTGAACGTTTTCACCATCTGCGCCATGAACAAGCTCTATTACTTCCACTCCGCTCGACGCGTTTTCAAGGATTTAGTATCGACGATGGCGGACAATTGGGAAGAGACGATGAGCGAGACTGGTCCTGACGGTGTCAAACATCGAGATATTCTGTTGGATATGGCAAGTAAAGCTCGCGTTTACACGATGCGATACGGTTTTCTCATGTATTCCACAGCTTCCATGTATTTCGTATCCCCGTTCATGGGAATGCAACCGGACGATCTCAGAGTGAGGAAATATCCGTTCTTCGGATGGTATTACTTTGATAGGTTTTCCGACGTTTATTACGGGCTCTGCTACTTGTCTCAGCTAATGGCAGGTGTATCGGTTGGAACGAGCAACTACTCCACCGACAGTATCTTCCTCGTGGCAATTTATCACTCTTGCGCTCAGTTGCGGATTATTCAACACGACTTGCAGAAGCTCGGCGAGGAAAGTAACGTTTCGCAGGCCAAAATAATCCGTCTCGTCGACAAGCATCAACGAGCGATTCGGACAGCGCGAAAACTCGAGGCAATGTTCAGTGGGTCGAGTATGCAACAACTGCTGGTCAGTTGCATTATAATATGCATCATCGGTTTGAAAATTATCGTTGCTTTGGATCAAGGTGGATTCCAAGTGTTGGTCTACGTTGCGTTCATGTTTGTCGCCCTACTGCAGATTTTCCTCTACTGCCAACCTGGCGACGAGCTCGTATCTCAGAGCGAAGCGGTGGGTTACGCGGTTTATCAGTCCATGTGGTACGCGCTCGACGTACCGGCGAAGAAGAAAATTCAAACGATAATACTGAGATCGCAGCGGCCGCTAAAAATGACGGCTGGCTCCTTTTACGTACTGTCCTTGCCGAATTTCACAAAGATCTTGAAAACGTCCATGTCTTTTCTGTCGCTCTTGAGGGCGATGTACTGA

>CcOR4 [partial 972 bp]

AGGCGAGCGCTGAATCTTGTGATCGAATCAACGAAAAAAGATTGGCTCGATTTGGAAAGACCAAATTGTCAATGGCACGCGGAGATAATGTACAAACATGCTTCGTTAGCGAGGTTCATCACCATTGTTGATCATGGAATTACACTCTTCAGTTACGTGGGTTACGTGTTCTTGCCTTTGATCAATATCGAAGTGCGTACTTATACAAACCTCACCGATTACGGTGATCGCCACCTTATGTTACAAACTTACTACCCTTACAACTATTCAAAAAGCTTTAACTTTGAGATGACTAGCGCATCTCAGTATATCGCTGGATTTTTCATCACCATAAGCTACACTATACCCGAGAATTTTTTCGGTGCTTTGGTATTTCACAGCAGCGCTCAATTCGAAATTCTTGCTGTTCAATTTGAGCGATTGTTCTCGAACGTTTATCAAACTCCTTCCAAGAATCAAGTCTCGAAAAGCGTCGCTAAACGAAATTTTACCTTGAAACTCAAAACTTTGGTCGACAGACACGTACATCTCATGACAATGGTTTCCATAATCGACAATTCATTCAACTTCGTAATACTTGCTCAAATCGTGTGTACTTCGGTGATAATTTGCAGCGCCGGCTACCAAATGATCGAGATGTTTGGAGGTAGATACGAAGAGCCGTCACTTTTTCAATATGCTACGCTAGTGGGAATTATATTTACAATGATGGCGCATACTTTACTAGAGTGTTTTGCTAGCGAAATCCTTACGTCAAAAAGCCAAGATGTATTTGAAAGCTTGTGCAAATCGAAATGGTATGCCGATATGACAAACCGACCGGTACGCGATCTCATTCCAGTTCTCAATATGTCGCAAATTCCACGACAACTTACAGCTGGCAAAGTATTGGTTCTCTCTTTGCCTACATTTTGTACTATTTTGAAAACTACAGCTGGATATATTTCTATGTTACTGGCCGTTACGACAAGA

>CcOR5 [partial 231 bp]

TTGACGGGGCTTTGGCCGTACCAGCACAAGTTGGTGAAATTCTTCGTTCGAATCCTTTTCTCGACTTTGATCGTTATTCCTGGAATACCACACGTTATCAGCGCAATCGTACACAGAGACGACGTGGAATTGTTGATTCAAGGTGTAGGAACGTTTTTTTATTTTATCACCATGGGCTCGAAATATTTTTCGATGCTCATCACGGAAGACAAGTTGAAATTTCTCTACAAA

>CcOR6 [partial 810 bp]

AAGGATCTCATGTACGGAGCAATGAAAAAAGCACGTAGTCGCAGTAATAAGTATTTTTTAGTGATAGGTGCTACAGCTATGCTTTACCTGATGATGCCATTCATGAGAGATAGCCCTTTTCGAGATCGTAAATATCCATTCTTCGGAAGATACTACTTTGATCGCAATTCCGATATCGTCTACTTAGCGTGTTACTTGAGTCAGATGTTGACCGGTTCATTAATTGGCTTGGCCAATTACGTTACGGACACCATGTTTCTCGTGTGCACGTATCACTTTTGCGCGCAGCTGCAGATTCTTCAAAACGACTTGATGGAGCTTGGTTACGATGCTCATATGAGTCGACATAATTATCTAACGAGTCTTATCAAAAGACATCAAGCAGAAATCAAGAACGCTCAAGCATTGCAATCAATGTTCAGCCAATCGAGCTTGCAGCAGGTATTGTTAAGTTGCCTGATGATCTGTATGAACGGTTTCAAATTGATTGTCTCACTGTCTAACCGCGACGTCGAAGTCGTAATGTATCTAGTGTGTCTGGTACTCACACTTCTACAGATACTTTGTTATTGCCAACCGGGCAACGAACTTATTGTTCAGAGTCAGTCGCTCGACGAGGCCATCTATCAATCTGCCTGGATCAATATGGATAAAAATTCAATGAAAAATCTTATCTTTGTGATACAAAGAAGTCAAAGACCACTTGCAGTGACTGCTGGAAAAATTTACGTTCTATCACTAGAGAATTTCATGAGGATAGTGAAAACGTCGATGTCCGGTCTCTCAGTACTCCAAGCGATCTACCGAAGA

>CcOR7 [partial 675 bp]

AACATTATATTAATAGCAGAAAATATCCAACAAGTCATAGGAAGTATGATTCAACCGTTAAATATAATCAACGTTTCGTGGAAAAATTTAACTTTTAGGCTAAAACGCAAAAAAGTGATACATTTTATGAAACTATTTTTTGATGAATTAACTCTACCACAAACTCCTCGAGAAGAAGCCATACAGAAAAAATTTGACAGTGAATCCAGAAACAATACATGGAAGTTGGCTTTAATTTACACAATATCAGTAGTAATGTATGTATACATGCCATTTTTTATATCGGATATCGAAGATAGAGTATTAGCTTATCGCGCATGGTTACCGTATTCGCTAGATAATGTTCATTATTACTACATGACGTATTTACATCAAAGCGTTGCGGTAACTATAGCGGCTCTAGGTAATGGCGCAACGGAAACGTTAGTTAGTGGATTTATGATACAGATTTGTGCACAATTCGAAATACTTGAAGAACGATTTCGACAACTGCCACAAATTCTGAACGAAATGAGGGCAAACAACGAGTTGGAGAGTAACATTTTGTTAACTGAAAAAAGATTGACAACCAGATTAATCAGACACCATTTACGCATTTTCGATATATTTGTTTTGGGCGTATGTACATACACATTGGCATTTTTTAAATCCATAAATAATAATTCTATAAGTATA

>CcOR8 [partial 897 bp]

AACTCGGATGTAGACGAGATGATTTGGCCCAACCGTTACGTACTCACGTGCGTCGGTTTGTGGCCTCACAAACCAAATGACAGCCATTCTGTTACGATATCTTCCACATTGCGAATTATGGTAGTAGCCATCGTGTTATTTGTCAGCGTACTTAATCAGTTGGACATGTTATCCAGTAATTGGCGGGACATCGATGTACTCACGGAGACAGGACTGACGCTAATGGCCAGCGTCCTACTTTTGCTAAAACTTTTGTACGTGTTTAATCGGCGAAGTGACTTTCACGGTGCATACCGTATGATAGGCAGTTTCTGGAGGTTGGCTAAAACCCAGGGGGAACGCAATCAGATTGTGGATCTCGCAAATAACGCTAGATTGTGTACGTTGATTTTTATCTACTGTGGCGTTTTTTACATTTCTTTATTGTCTGTTGGAACTTATTTCGTAACAATGTGGAACCGCGCGCACGCAAATGGCTCCGAATACGTACACAGAACACCGTACTGTCATTGGTCTGGAGTCGATGATTCCGATACGTCGCCAAACTATGAACTTATTCTCGTACTACAATCTATGTCCGCTTTTGTGGCTTTATGTACAGCTGTAGCAATCGATATATCCGTAATGCTTATTTTAATGCACGTAGCTGGCCAGTTCCGGCTAATTAGTTTCAGGTTACAAATAATTGGACGACAAATGTCTCGTGTCGATGAGCTCCCAAGCCATTTAATTGGTGGATTCAACAAGGAGATTAGATCGTGCATAATTCACCAACAAATAATGCTCAGCTTGTTCGAGTATCTGGAGAAATTACTAAGTCCGCTTACGTTTGGCCAATTTCTTATATCAGCGTTGGAAATTTGCTTCGCCGGTTGGGCACTTATAGAAGCTGACACT

>CcOR9 [partial 948 bp]

GTGCTTTTATACGTACAGGGCTATCGGCTGGTGTTTACCGTTGCGGTTTTGGAAGAAACTTTGCAGGACTTGTCAAGTTTGTGGCAAAAGTACATTTCCAATCATCAAGCACGCGGACGGTTCTTGAAAAAAGCCAGCGAAACCCTACTATTTTGCAATATATTCGTATACGCAACTTTGAGTGCCGTTATGAGCTTTTGCTTGCCACCGATGTACAATTTGTTGAACGAGTATTTACATCGAAAAGACGACAATTACACCCACGATTACGAGCAAAGAATATTCTTCTTCAAGTACCCCTTCGAAGTGAACAGTTTTCAAAGGTACTTGTCCGTCATGTCGTGCGAAGGATACTTGCTCGTCGGCGCTGGTTTGATGTGGGTTGCAAGCGACGTTATATTCGCACAGTTGACCACCCACGTTTCCTTGCAACTGCAGGTGTTGCAATACGACATGAAAGTTTTGATCGATCGCAACGTTAGCGACGGTATTTTGAAACGAGGCTTCAACGAATTCGTAAAAAGACATCAACAAATACTGAGCATTTGCAACCAACTCGAGTACGTGTTTAGCCCTGCCATTCTGTCGACCGTATTTCTCTCGGGAATAAACATATGCTTCAACGTGTTTGAAGCACGCGAGACCGTATCGAAGAAAAACTACGCCGATGCCTCGGTACACGTCTTTCTGTTTGTTCTGACGTTTTTGCAAATTCTATTCTACTGCACTTTTGCCGAAGGACTTACCCAGGAGACGATCACGATTGGCAACACCATTTACAATTCAAATTGGCCACGGAATCATCCAGTTCTAGCGGTTTACCTAAAGATGATCATCGTTAAATCTCAGAGTCCGTTTTATTGTACAGCCTATGGATTTTTCCCCATAGGACACGCGCGATTAAGCTCGATACTGAACGCGGCCTACTCTTACTACATGATGCTACAA

>CcOR10[ORF 1263 bp]

ATGGGTTCCGTGAGGGTGAATCCAAAGCCACGAGGGCAGCGAGAAGTTTACGTCAATGCCCACTACGTGCAAGATACCGAGTATGTTGTTCGCGTTGCTAAAACACTTCTATCGCCTGTTGGAATTTGGCCACGGACCGGTGACAACGACCCTACGAGCAATCTTATTTTTACCGTCCGCGTGATTCTAATTTTCTGTTTGATGTTGTTCCTGATGACACCACATTTAATCTGGACATGGTTCGTCGCCGACAACCTACGCAAGCTTATGAAGATCATCGCTGCTCAAGTTTTCAGCTCTTTAGCGGTCTTGAAATTCTGGACACTCATAATCAACAAACGAGATATTCGTCATTGCCTGGAAGTAATGGAAAATGATTATAAAACAGTCGAATCTGAGGATGACCGTCAGATAATGATTAAAAATGCGAAAATAGGTCGTTTCTTCACCGTCGCATATCTCGGTCTTTCTTACGGTGGTGCTTTACCATATCACATAATAATGCCACTTTTAGAACCACGAATTATTCGTTCGGATAACACTACAATGATTCCATTACCGTATCCAAGCGAGTACGTCTTTTTCGTAGTGGAATTCTCGCCACTGTACGAGATAGTGTTCGTCACGCAAATTTTCATCAGCGCACTCATCCTGTCAATCAATACTGGTGTGTACAGCTTGATCGCATGTGTGGTCATGCACTCGTGTTGTCTGTTCGAGGTGACGGGTAATAAAATCGACAAATGGCTTGCCAATTGGACGAGTGATCAACCTCTCAGCAATGCACTTACGAGGAAGCTCAGTAAGATTATCAACTTTCACGTGCAAGCTATACTGTACGCCGAGACTATGGAAAACGCACTTACGATAGTTATGCTTGCTGAAATGGGTGGCTGCACGCTGATTATATGCCTTTTGGAGTACGGGATCTTGCTGGATATGGAGGATGGTGATTACCTTGGCTGTGCGACTTACGCTATGCTAATGACATCCATATTTGTGAATGTGTTCATTCTTTCTTTTGTTGGCGACAAAATTAAGGAGCAGAGCGAGTTAATTGGCTTCTCCGCTTACTCGATCGACTGGCTTGAGCTGCCGAAGGAAGTGATACTGAAGGATCTCAAGTTCATCATGGCGCGAGCTAATCAGCCCACGAGATTAACGGCCGGCAAGCTCTTTGATCTCTCGCTTCAAGGATTCTGCGACGTCGCTAAGACTTCGATGGCTTACTTGAACTTTCTGCGAACCCTTGAGATCACGTAA

>CcOR11 [partial 675 bp]

TTTATTCTTACTGCATACTTCGTTAGAATTACAGGGCCAACCGTGAACTCAACCGATAGGAAAACGTTGCCATTCAGATTCTTTATGGAAGTGTACGAGGAGCCAATGTTTACGTTGTCATCGTTACTGCAGTACTTGGTAACGTACTCGGTAGCATTTGAAATAGCAGGTATTGAAACAGTTGGTCTCTACTTAATAATGATGGCCTGTGGTTATCTGAGACTCTTGAAAAACAGACTTCTAAACATCCAGAATACGACTCAGAAATCGAATAGTAAGAACATATACGATGACGAATGTAGCGAAGTGTTATACTGTGCACAATTTCATCAGAGAATAATGATATTTTGCGAAGGCATAGAAAATGTCACCAATACCGTATTCTTCCTTGCGGTGTTCACAACCATTTACAACATGTCGATTACCGGTCTAAAGATTCTAGAAAATGACGAAAGTATGGTGAAATTCATCATGATATTCTCACTAAATCTCTTTCAATTTTTAACCGTCCAATGGGGTCCTGAATTCCTTCTAATAGAAAGCGAAGCAATTGGCAGGGCCGCATACTTTGCCAGTATGCAGCAGATAAGTTACAAATCGAAAAATAACAAAGTGCTGATGTTAATGATGATGCGAGCACAGCGTCCCTTTCAACTTACTGCCGGGGGTTATATC

>CcOR12 [partial 741 bp]

TATCACATGATTCTCGAGTATCTCGATCTCTTTTTGTTCATTGACAATCTTGAACACGTCATCATGAATCTTACGGAAAACATGGCGTTCTCGCAAATTTTCGTTAGAATGTTGATGTTGCGACTTCACAACGCTCCTTTGGGTGAAATTATCGTCGAAGCTATGAAGGATTTCGATTCGAAGGATTACACCAAGCAAGAGAGGAAGACATTTGTAGCTTATCACGCGAAATCCAAAATTTTTATGAAACTGTTGATGTCGAACACTGCGCTCACGGCTACGTCTTATTACGCGAAGCCACTTCTTGGGCAGATGGGTGAAATCATAGCGTACTCGAATGGCGCAAACGCAAGTTTTATATTGATGCTACCGTATCGTTTTTACACTTTCTACGAGTTAAACGCCAAGTCGTATTTCTGGACGTACGTCTCTCAATTACCATTTGTTTTTGTGAGTGGTTTTGGTCAAAGCGCCGCAGATTGTCTCATGGTCACCCTCGTTTATCACGTATCTGGACAGATGGCTATTTTAGCTATGAGAATTGCTCAACTCGACACCGAAACCAGCGACTGCAGTAAACAACTTAGACGACTCGTTCGATCTCATATCAGACTACTCGCGATGGGTCAAGTGATACAGGACGCATTTAGCGCAACACTCCTCGGACATTTAATAGGTGCTACATCTCTCGTCTGTATCCTTGGTTATCAAATTTTAGTGTGTTTGGCCATTGGAGAACGC

>CcOR13 [partial 555 bp]

CAGGCGACCTTGGCAATACACACTACTGCCGAACTCTATCTGAACAATGGCAAAATATCGGCGATGGTCGACGTCGTCCTCTTTTGCAGTTCGGCATATTTGACACTCGTCAAAACGATTGGTCTTCACGTTCATCGTCGGAAAATTGCCGAAAATTTGGAGTGTTGTCGCGACGATTGGGCGCAAACGACGGATGAGAATTCGTTAAGAATCATGAGAGAGCATTTGAAGGTATACAGATGTCAATTTTTGTTGTACAACTCGTGCGGCTACATTGGTACGATAATGTTCGTGATCCGAACGATCGTTGTTAATTTCCTCGTAAAGCGTCAATTGGATCCCAATGAAGATTTCGAATACGCTCTTGCCTGTCAAACCTCGTTTTTATCGCCACAATTTATAGCTTATTTTTACGCGCCTATTCTGACGGTACAATTCGTCCAATTACTTTATTGCTGCACCAGCGGAGCTTGTACGGACTGTTTTTTTTTTGGCCTCGCCTATCACTTGAGTGGCCAATTCGAAATTCTCAAAGTCGAGTGGCAAACCTTAGGA

>CcOR14 [partial 837 bp]

ATAGAGTTGTATCACAAGACCTTAGAAATAAACACTTATTTGAAAGAAACATCGGCTGAAAAACAAATCACCTACAGATGGCTAAGTTTGCAGAATAAATTGATAAGTAACGTCATCATATCTTACCTCTTTATCGTTGGCATCTACTTTTTATATCCGCTATACCGGGGTGACGCGTTTCCGATAACCCCAGGATTAGTGCCGAAAGTATTTAACAAAATACCCTGGAATATACTCGTGTTCGTATACGAATTTGCCATATGTACCCTTCGACTCATTGTCATAATGTCTTGCGACATTTTCCTCGTTATTTTTATGTGTCAACTTTGTAGCGAGTTACAAATTGTTGGTAACGCAATGATAGAGATAGATGTACACGATAAACGGACGATATTTCGAGCGATCAAGAAACACAAAAGGGTGTTGGATTATGGACGAGTAATATGCGACGAATTCTCTCCGGCAATTGTGATGCAACATCTTGGGATGTCGTTTGCTGCGTGTTTTTCGGCAATTAGCGTACTTTACACAAAAGAGCAGATGCTTTTGTTGAAATTTATATCAATTGGACTGATAATTATCGCTGAAATATTGCTTCTCTGCTTTGTCGGTGAAACTATCACTGGGGAAAGTCTCAAAATAGCCGATTACCTTGAAAACAAATTCGAAGTATTCTTAGATGACGCAACGTCGTTGAAAACAATTTGTTTTATGTTGTTTCGTGCTCAAAGACCGCTAAGTTTGAGAGTAGGCGGTAACACGATAATGAATCTCAACTTGTTTAGACTGACGATGAACAAGATATTCTCCTGCTTCGTTATACTAAAGAGAGCAATG

>CcOR15 [partial 1086 bp]

CCGGAACTTTGGGCTTTGACGATGGTTTTTGATCAATTTCAATTGGTGATCGACAATCTACTGACGAGCAGTCCAACTTTTACATCGTGTTTCAAGCTGTTGTTCATGTGGCGCGAAGCTCATGTTCTAGAACCCGTGGTGGAGTCATCCGCACGCGACTGGATCGTTGACCCGAAAAAACGAAACTACAGGGAGTTGGAGATAATGAGGCGACACGCGTATCGCGCGAGGATCGTTACCATCGTTGATTACTTCATCATCATGTGCTGTTTTGCCTTCTTTGTATACGCACCTCTTTTTGGTCTCAACATTCGCATTATCAACAATATCACCGACTATGCCGAACGTCGACACCTCATGGTCCAGACTTACTATCCTTATGATTATTCCACCTCGCCGCTCTTCGAAATGACGGTGTTTGTCCAGCAGCTGAGCTGCCTCTTTATCGCCATGGCCGTCTCCATTCCTGATAATTACTTTGTCGCTCTTGTGCTTCATACCAGTGCTCAATACGAGATTCTTGGTGCTGACATTGAAAATTTCTTTCCCCACTCGTCTTCTTCTCCGAGCGTCGGAGCAAGCTTTTTCATCGACGAGCAGCTCAACGATCGGATACTGGCTAGCTTCGTCGATAGACACGTACATCTAAACAGGATGGTCTCTCTCATCGAGCAATCCTTTAGTTTCGTCACTGCTGTGCAAGTGTTTTGTATGATTTCCATGGTTTGCTGTTTGGGATTCGAAACGTTAGGGATGCTCGATCCAAATATGACCAGCAGCAAGCCTACACCTTTGCAAATATTAACGATTCTCGCTATGCTCTTCTACATTATGTTACACACTTTGGTTAACTGTCTTGCCAGCGAAACTTTAGCTTTGCGCAGCGAGGCCGTTCATTCGAATATTTATAACAGCCAATGGTATACGATGCCTTGCAAACAGATGCGCTACTTTATTCCAATGATGATCGTGTCCAAGAATCCACAGCAAATCAAAGCTGGCAGGATTTTACCAATGTCAATGGAAACATATTGTAGCATAGTAAAAACTACGGCCGGATACCTTTCCGTACTTATTGCCGTTAGC

>CcOR16 [partial 984 bp]

GACGACATGAACGAAACGATCGAATGTAGCCTCGTCGCTTCGGCGTTTCACATGGCTATGTTACGTTTTATATTGTTTAGTTTACGACGCAAGGATATGCTTTACGTTCTTAACGTGATGCGCGACGATTGGACCGTCAATTATCGCGATCCCGACGACGCTCGACTTCTCGCTGACAAAACTTTGGCTGCGTTTAAACTGTCCAAACTATTTACTCTCAATGTCATATTCGCTGGATCGACTTTTGCAGTAATGCCTGCGCTCGAGCAAGCGTTGTCTTCCGGGGCCGATCACAAAACTGGGGTACTGCCGTTCGGTGGTTATTATTATTTCAATCACACGAGTTCAAACTTGGTCTACGCTATCAGTTACACGTTCAACTCGATGCTCGGAATTATCGGTTGTAGTACGATCGCGGGTGCGACTAGTTTTAGCCTCATCAGTACCATGCACGCGGCCGCAAAGTTCGCACTCGTTCGTCGGCACTTTCAATCCATCACTCATCGCGAGTGGATGCAGCAACCGTCTTTCAAGTTCAATCTCTGCGTTCGAGAACACCAGCAATCCATCAAATTTGCAGAAACGGTCGAGAAGATAATCAACTTGTTAGCGCTCGCTCAGTTCGTCGTTAGCACGGGTCTCGTGTGCTTCGCTGGATTTCAATTGACTACGATGTTGGAAGACAGAGCGCGTTTGACCAAATATAGCTCTTTTCTCAACGCTGCAATCTTGGAGCTATTTGTTTTCAGTTACAGCGGTCAGAGATTGAAAACCGAGAGCGAGGCTGTGGCAGACTTTGCGTACTCGAGCGATTGGCTAGGAGCTTATCGAAAGTCCATTGACATACGAATGGTTATAATTCGAGCAAGTAAAGCTTGCACCATTACAGCGGCAAAGTTCTACGACATGTCTCTCGAGTCATTTTTAAAAGTTCTTAGCTCTTCGTTTTCCTATTTCTCAGTGCTGATTGCCTCGCAAGACAAG

>CcOR17 [partial 267 bp]

GAAGATTACAACTCTGTAATAAGATGGAATCGGTATTTGCTCAACATTGTCGGCTGTTGGCCCGATCACAATTATCAATCAAGTTTTCATTCGAAATGTCGATTTTTCCTATCTGCTTTTCTGATGGTTTACTTTACAACAGCACCACAAACCGCTCATTTGATTCTGATCTGCAGAGATATGGATAGTTTTGTTGAAAACTTAGCAATGGCCAACATTGCTATTACTAATGCACTAATTAAAGTGACGGTGTGGTGGTGGAATAAA

>CcOR18 [partial 315 bp]

ATTATTAGTGCAAGCAATTTTGAAGATTTAACAGGAAGTTGCTTTATGTTGTTATCGATGCTTAATGTTTGTTGCAAAATGACAAATATTTTATACTTTCGTAGCAACATCGTTCAATTGTTGGAGATTTTAAAAAGCGATTATTGCAGAGCAAAAGATGGAGTAGAACGAGATATACTAGAGAAATTTCATTATAAGGCCAGATTTGTTACAGTATGCTATGCATCTCTAACGGAGACTACGTGTATACTCATAACAGTTCGAACATTCTTTGGAAGTATTAACAGAGTACTGCCCTTTAAAGCCTGGATACCG

>CcOR19 [partial 789 bp]

ATGTCGAAAATCGATAAAAACACGAAAATTGTCGAAGTTGAAGAGCGAGAGACGACGCAACATTTCGAGTACTCGATAAAAATGATGCGTTGGCTTCTTCAATTTGGAGGGATGTGGCCTGTCGTATCTACGACTTATCCAAAAATTTTACGTCTACTTTTGACTAGCGTTGTTTGGTTCATACTCTTGTTTTTCACCATACCATCCTGCCTTCACATGTGCATCATTGTCAAAGATTTTAAGATTAGACTACGATTAATTGGTCCATTGAGCTTTGGCTTGATGAATATCTTCAAATTCGTTAGCGTATTAATGCGAGAAAATCAACTTCGCGAGTGTATCGAGTGGATGGCGACGGATTGGCGATCGGTGCGTAGCCAAGTCGAGAAGAGTCTAATGTTTGAAAATGCAAAATCAGCCAAAACTTTTACGATGATTTGCGTTGTCTTCAAGTACGTGAGCGGTATATCTTACAGTGCGCTTTTTAGCTTTCTGAGAGATCCCATTGTCGTCAACAACAAGACCACCAGAATGTTGGCGTATCCAGGCTACTTTATTTTCTTCGATCCATACGATCATTTTAGAAAAATGTACGTATTGCACACGATGGTGAGTATCACCCAGTCGACGATAACGTGTGGCGTGGCGAGCATCGGTATCGTCTGCGTGATGCACGTATGTGGTCAAATTGCCGTAACGTCTTGGATGCTTCGTAAAGTCGATGCCGATGGATTTGACGCTGTGATCTTTGGGCGTATAGTAGAGAAACATCTACGTGCCATCGCATTT

>CcOR20 [partial 255 bp]

TTTGCTGAATCTACTCGTGTGACACGTCGTTGTTTGATCGATCGATTTCAGAGTTTATACTTGTTTGACGCTCTGTACGATAACTCTTGGTACAAACTCAATGCAATTGACATGAAGTATTTTGTGATATTTTTAAATCAAACGCGACGTCCATTGGTCATCACCGGAGGTGGATTTTTTGTATATTCACTCGAAAATTTTCTGTCTGTCGTGAAGTCTGCAATGGCTTATTTATCGGTACTACGTACGGTGCTG

>CcOR21 [partial 216 bp]

GTAAAAAACTTGTTGACTTGCGTCGCTGTTATCGCTGAGTTCTGCACGACTTTTGGTATCATCAACTTTTGCGTGCATAACTTGACCAACATTAACGTACTAACGAGAGGCCTGGGTCTTATGATCAGTTATTCATCGGCATTGCTCAAAATGCTGATATTGACGTTCCACCGAAACGACTTGACCAAGCTCAACCGCACTTTATCGGATCAGTTT

>CcOR22 [partial 486 bp]

AGATTTTGCGCCAAGATCAACAAGTTGGCGCGGACGGTGTTGCTGCTTCAGGTACTCAGCACCGGCTACAACATCTCGCTGATGCTCGTCAAACTCGTCAGCGTGAGTATACACCTTCGTACGCAATGAAATAGAGCTGCGCAGTGTCGGTAATTATTGACGATAACGATTGACCCCTTGGACAAGTCGGACCGTGACAAGTACAAATACGTATCGATACTCATGGTGTACGTCGTGCAACTGTATCTCTGCCAATGGACCCCGGATCATCTGTTCGACGAGAGCCTGGCCGTGTCGACAGCTGCTTACTTTGCCTCGGTATACTGGATGCCGCGACGAATGGGAAAACATCTGCTAACGATTATAGCAAGAGCTCAGCATCCCGTGCAAATCACCGCAGGTGGATTCGTCAATCTCTCGCTTCAAAGCTTTGGTTCCATGCTCACCAGTGCCTTCTCCTTTTTCACTTTACTCCGTAAGTTGAAT

>CcOR23 [partial 528 bp]

ATGGATGTAAAGTTAGCAACGTACACAACGTTCCGAGATCGTATTCAAAATTTAATGCTATGCGGTGGTGTTAGACCATACGAAGGTAAATTTCACTTATGCAGAATCATCGCATTCTACGGGATATTTATGCTCGTTACACTAGTTGGCAAAATTTCACTAATATGCGTGGCAAATATAAAAAACATATTGATGGTCACTCAAAGCGTAAGTCTTATTTTCAGCTTGAGTACGCTTTTCTTAAAAATAATCGTATGCATGGTATATCGCGAGGACATGCTAATGCTGGAGAAAAATTTAGGTGGAAAATTTGAGAAAGAATTGAAAAAAGAAGAAAACCGACCAATACTACTTGAAAGAATAACGGTATACACGAAATTAATGTACATATTGGCGTTGCTTCTCTTCCCATCGTTATTTATACCTACCACCTTACCAATTCTCATGGCAATTAAAGGCAACAATTACATCTATATTTATTTGGGAAATTATCCATTCCCAGTGACAGGAGGGAGTCCACTATACTGG

>CcOR24 [partial 237 bp]

ATTACACGCACTAACGAGCCCGTACACATGTTACAATTTTTCATGGATACAGTGTCGATTATATTTCGCTTATTCTATATTTGTTGGCCAGGGCAAAAAATAGTAGACCACAGTTCTCAATTTAATTATGTCTTGAATAATATTCCATGGTACAATTATCCCGTGAAGTCAAAGAAGTTACTCTTACTTATGTTGCTACGAAGTTCCTCACCATGTTATATTACTGCGGGCAAAATG

>CcOR25 [partial 306 bp]

CTCGATGAGAGTTTCAACGATTGCATCAGATTTGCCATGTTTACCATAGCCCAGATATTTCATATGTTTTGTTATAATTTCCTGGGCCAGAAACTTTTGGATCAAGGTGAAACGTTTTTGAACTACGTTTGTGAATCCGATTGGCATTTAGCGTCCAAAAAAACAAAGTACATCTTTAACTTTATGATATTGAGAAGTTCGAAGCCTAACAAATTGCAAGCCAAAATTTTCCCATTGACGTTGGAAAATTTTACATCTGTTATGAAAACTTCCATGTCTTACTTTACCGTACTCAAATCAACGAGG

>CcOR26 [partial 153 bp]

GATTTCCTCGATAGTCGTTATTTCGTTGTTAATAAATGTTTGCTAAGGTTTATGGGACTTTGGCCTTATCAGCACGTGTTGGCAAAGATCGTCGTTCGCGTTGTATTTACCGTGCTTATCGTCGTCTTCACGATTCCCCAAGTCATTTGCATG

>CcOR27 [partial 1143 bp]

ATGGAAGCCGAAGTAGAATCTTATTATCGCGAGTATAAAGATGGAATTGTTTCGTTGCTCACATTGTCGGGTCTTTGGCCAGAAGATTCTCGTCAGATTAAATACGTACGCGTAGCCTTATTCAGTGTAACTTTGATTTTCGAATTGGTATTTGCTGGCGCCGTCTGCAACTTTGCTATGGTGAACTCTCACAAAATGAGCGCTGTAGTTGCTAGCGTTGCGGTACTCATTAGCTTCATTTTTGTCATCGCCAAGTTGTTAGTAATGTTAATATACAAGGAAGACGTGTTATACCTAAATCGTAACCTGGCATCCTACTTCGAAAGCGACCTCGAGATAACGGAGTATCGCCCGTACTTGTTAGCTTACTACCGTGTGTTTTACCGATTTTTTAAAGCTCACTACTACTACTTCAACGCTATTCTTCTCTTTGGAATGACCGCGCCCATTGTCGCAGCTTTTTACGGTAAATCTGCTAGAGTATATCCGATTCATCCACCATTCCATTACGAGCCAGGTGGAACAAGACATTGGATGATCTTTGTATTCGAAGTGATTTCGGGAGCTTACTCGGTCACTACTACCGTTAGTTATGATTCTCTTTTTGGACTCTACGCGTTTAATCTTGTGGGTGAAATACGTTTATTGACACATCGATTTCGTAACTTGACCCCTGGTAAGAGCTACAGAAACCAACTTAAAGAGTGCGTGGACAAACATGTAATTCTTATGAATGCAAAAAATGTTATGCAACGTTTATTTGGCGCGATGTCGGTGATGTTGGCTATCACTTGTGCTGTTATTATTTGCGCTCAAATCTTTACTCTTACTACGAATAAACAATTGCCCGTATTTAAAGTAGCCTTTTTAATAGATTACATCATTGCAAAATTATTACAAGCTTACATGTACGCTTGGTTTGGAAATTGCATCACTGTAGAAAGCGAAATATGTCTCGATGCAATATACAACGCTGAATGGGCTGGATCGGGTGAATTACGCTTGATGAAGGATATTCTGATTGTACAGTCGCAAAATCCTATGCAATTCGAAACAATGGGTATAATGAAAGTTCGATTGGATATGTTTTTGAAGATTGTCAATACGTCGATCTCTTATTTTTTCCTACTGAAGACCGTCGAG

>CcOR28 [partial 333 bp]

AAGATCTTGGCATTAGTTGAAAAATGTGCAGGTTTTTGGCCTTATCAAAGCAAGTCTATTAACTATCCTATACGATCGCTTCTTTTCTTTTTTCTTATATCGTCGACCATCACATCGGTGGCCGCAATAATAAATAGAGAAGGAGTAGATCCATTGGTCATTATTGAAACCGGACTTTCTATGTTTATAAAGACCGCCGTGATTACGAAATTGATAATGGCCTGTCTTTATCAAACTTACAGACAAGGTTGTTATGAAAAAATCGTTCATCATTTTAAAATAGTTACCGATGAAGATGAATCGAAAATATTGACCAGTTGCTGCGATAATGGA

>CcOR29 [partial 504 bp]

ACCGTCGTTATTCCAGAGGTCATATTTTTCAAAGAAATGATCGGTGAGAGAAACTGGAATAAAGTTGTCGATTGCTTAACCGCTTTAATTGCAACATATGGGGCATTCGTTATAATTGCGAATAGCATATTCCAATTTCATAAGATAAGACAAACTGTCGAACAAATGCAGAGCAACTGGGACAATTTGGACGATAGTAACGAATTGGCAATTTACACGGATTATGCGAAGACGGGAAAACTTTTGATTTTGACGTACTTGATTCTTGCAACAGGCACGTTTTCCTTTTTTTGCTCCATACCGTTCCTTCCGTTTCTGCTGGATATTGTTATTCCGTTAAACGAATCTCGCCCCGTAAATAACTTACTTCAAGTACAATATTACCTAGATACGAGAAAGTATTTCGTGCCAATTTATTTGCACGGCGTACAAGCTGCCATTTCGGTGGTCTATACGATTATAACGTTTGATTTCTATTTCATAATGATCGTACAGCACGCTTGC

>CcOR30 [partial 579 bp]

ACAGTTGGAGCTTGCAGCCTTGCCGCTGGCTTTGTAGTGCACGTTTGTGCTCAACTTGAGATTGTCATGTGTTTACTTGATGATTTTGTTCGGGGAAATGATGCTGGAAGTACTGACAAGATGAAAGAAATAATCGACAAGCATATTCGAGCACTTAGCTTTGCAATGAGAGTTGAGAAAATGCTGAACGTCATTTGCTTTGTTGAGTTTATCGGATGCACACTTAACATTTGCTTTCTTGGATATTACTTTATCACGGAATGGGAAAGTCGAAATACTATTAGTACTATGACCTACTGTATTCTTCTCATATCGTTCACATTTAACATCTTCATCTTTTGTTACATTGGAGAGATTCTCACCGAACAGTGTAAAAAAGTTGGCGAAATATCTTACACGATTGACTGGTACAAACTTCCGGGTAGGAAAGCTTTGGATATAATCATGATAATCGTAATATCTAGTTGTTATCCAGCAAGAATCACCGCTGGAAAGATGGTTTATTTATCGATGAGCAGTTTTTGTAATGTAATCAAAACCGCGGCAACATATCTGAATCTCTTACGAACAGTCATGCTT

>CcOR31 [ORF 1158 bp]

ATGAAGACAGAGTTTAAAAAATACGAGACGTACGTAGGCAACGTAGAAACTATGCTTCGTTATTGTGGTTTGTGGCCTGTAGTTGTCAATCGAACGATTAGTCGAAGTCTTTCTTTCTTAGCGTTTATTACGACATTTAGCACTATGCTATCGGTGCTTAATTTTTGTTATCATCACTCAAACAACATCATTGTCCTTACCAAAGGTGCGGGATTGGCTATTAGTTTATGTACTGCTTGCTTAAAGGTCTGCATTTTCGTCTATCACCAACAAGATTTGTTGTATCTTCATGAAAATCTAACTACGAGGTATCTTATGGACATGAAGGATGTTAACAATAGAGCACGATTGTTGAATCGTGTCTCGTTGTATTCCAAGTTCTTTTGGATTGGCACTGTTGCTGCTTTTGCTACTATAGCCCTTTACGCAAGCATATCGTTCATCGCTTGGGCAAAGTATGGCAAATACGTTCGAGTTTTTCCGGCGATTTATCCTCTAGTCGGCAAACCCACTGGACTAGTACATTGGGCTTTTTACGTATACGAGATGACTACAGGATTGTACTTGAGTTTTGTTACTGTTGCAGTCGATTGTTGTTTTGGCATGTACTCGATGCAAATGTGCGGCCTATTTCGAGTTCTGTCAGATAGATTTCGAAATTTAAAATCTGATCGAGATTACAAGATGAACATCAAAGACTGCATACAGAGGCATCATACGCTTTATACATCGAAACGGAAGCTTGAAAATCTGTTTGGCATTTTAGCTATTTGGTTTGCCGTCACCGCAGCTGTCGTTCTATGTACGTTGATCTTTCAATTCACTCAGACAATCAAAATGAGAACGACATGGTTGCAACTGGGTCTTCTCACTTTGTATTTTTTACTAAAGTGTTTGCAGGCGTTTAGCTTTTCCGTGTATGGCAACGCTATAACCGTAGAAAGCACGTTATGCTTGGATGCTGCGTACAATGCTCATTGGCCAGATTTATACAACGTGAGTCTGAAAAATGATATTCTTATAATCTTAGCTCAAAAACCAATAACTCTCGTGGCAAAAGGTTGCATGTTGATCCAGTTGGAAATGTTTGCCAAGATTATAAATACCTCGGTGTCTTATTTCTTTTTACTACAAACATTGGAAGAAGGATCACGTTAG

>CcOR32 [partial 351 bp]

GTTATCGGCATAGCAGTGCTACTAGTAATGCTAATAAAATTGATAATGCCTCGGATTAAACGACATTACAAGCAGCAAGTTTACGAAAAAATACGAGGACATTTTCGCTTTACAACCGATCTGGCGGAGGCTCGAGTTTTACGCAATTTCTACGATAAAGGCTGGAGGTTTGTCATCGCGTACACGGTCATAAATTTCATCATTTCTGGTCTATTTTATGGAATGCCAATGCTCAAGGCACGTTTCAATCACTTTTTCAACAATATTACCCCGAAAAAGGAATTTCCCACTTCAGTGGATTACCTTGTGGACCAAGACAAGTATTTTTATCTCATATTGATACATATTTAT

>CcOR33 [partial 117 bp]

TCAACGCACCGTCTAGTTGTTTTGACGGCAGGTAAAATGACCAATTTGTCGATATGCGGCTTCTCGAAGATTTTAAAAGCCTCTTTGACGTACTTGAACATGCTTCGGACTGTCACA

>CcOR34 [partial 102 bp]

AGATATTCATCTATTTTTTTGGCAACTTGTATACATTTCTATGTAATGTTTACACAAGGTCAAAAAATAATTGACTCCAGTTTATCTGTATACAATGCTTGG

>CcOR35 [partial 390 bp]

AGACATCATGTGCTCATGAAATCAATGAGTACGTTGGAGAAAATATTCAGTATTCTTGTATTCTGGTTGGCTGGAACTAGCGCCATCATTCAGTGCACTTTTATGTTTCAGATGACAGTGTTGCTGAAAACAAAGAATATTCCAGAATTAATGTATACATTCGCGTACATAATTTTGAAACTCCTTCAAGCTTTCATGTACGCTTGGTATGGAAATCTCATCACCGACGAAAGTCAATCATGCTTGGATGCAATGTATAACGCTGCTTGGGCTGGATCCGGTGACAAACAATTAATGAGTGATGTACTTATTGTACTGTCACAGAGACCATTGGTTTTCATTGCGATAGGATGGTTGAGAGTGAAAATGGAAATGTTTTTAACAGTAATT

>CcOR36 [partial 378 bp]

CACAACGACACCATAAGGTGCTGCGACTTACTTGAAAATTCGTTCAACTTGTGCTTTTTGATGGTCAACTGCATGAGCGTCGTTGGATTAGCCCTTGCTTTGGTTTACATACTCTTTATTTTCGAGGATTTATTCAAATTGATACGGATCGTCGCCTTCGTAATTGGCTTGATCATACATTTGTTGTACCTAAACTGGGTCGGCCAACAGATCATTGACAGCAGCGAGCAAGTTTTCATATCTGCGTACTTTAGCCACTGGTACTTTATATCCAACAAATCCAGAAAACTTATCGCAATCATCTTGATTCGTTCGTTGACACCTTGCATATTGACGGCTGGCAAACTGGCAACGCTGTCCATGGAAAGCTTTGGAATT

>CcOR37 [partial 303 bp]

AGTGGTGACGTCGTCATGCTGATCAAATTCATATTGTTATGTATGCTCTTCTCATGGCAAGGGTATTCCTTTTGTTTTTCTGGTCAGCGACTAATCAACAAGAGTGATACAATTTCGAACAAAATTTATGAGGCATCTTGGTTTAACGCGAATCCCAAAGAAATTCGCGCTATATGTTACGTGATAAACAGAGCGCAATCACCTCTCACACTAACAGTAGGAAAGTTTACCTCTCTGTCGGCGCAAACTTTCACTGCGATGATGAAAACGTCTTTCTCGTACCTATCCGTCCTCAGAGCGAGC

>CcOR38 [partial 309 bp]

TTATCCAAGCAATCGCTATTCAGTGCGGAATTTTTTGCAGTATTTGTATATCTGTGTTGCGCTATGGTACAAAATCATTTTTATACCTGGTATGGCTACAAACTAACACAATTTACTGCGGAAATTCCCTGCACAATTTTTACAATGGATTGGACTATTCTACATATAAAATCAAAAAAGATGTTGTATTTTCTAATGTTATCTGCATCGAACAGTATAACGTTATTCCACAACTTTATCGTGAATTTGTCGCCGGAAATGTTTCTAAAGATGATCAAGCTGTCGTACTCAGCGTTTAATTTGTTGCAA

>CcOR39 [partial 354 bp]

GGATACGAAGAATGTATCTCGAATACGCGCTGGACAATGAAAACCGTGGGTTTATGGCCACTCGATGATGCTGTGATCCTATCGACGTGCAAACTTATTATTGGGATGATAGCAATGGTGTTTACTTTGATCATTCCGCAGACGAGAAAAGTCATCAAAGTGCACGATGATTTTAATTTGACGCTTGAAATCTTGACCATTGCTAACATTGATTTTTACGTTGCTCTGTTGAAACTTATAACGGTCTGGTATTACAAAATAGATTTAAGAAAGCTCTTGCTACAGATACACGCAGATTGGCAGAACACAAGCACGGAAGAACAACGGAAAATGTGGTTGAACGCAAGGATGAGC

>CcOR40 [partial 393 bp]

GACTGCGTGAGAAGACACGTTCTATTAACGAAGTCACATCACTTTCTTGAGAAGGTTTTTGGCTTTCTTGCTATTTGGCTTGCGTTGACTTGTGCTATGGTTCTCTGTGCTCTCATTTTTCAAGCATCACAGATGAAGCATTTGAGTGCCTTAAAAGTATGTTACTTGGTGTGTTATTCGTTTTTAAAATTGGTGCAAGCTTTTTCTTACGCATGGTTTGGAAACATAATAAATGTCGAAAGCGAGGCATGTTTAGAAGCGATGTACGATGCTCATTGGCCAGGCTCTGGTGATACAATTTTTATGAGTGACGTCCTCATCGTTTTATCGCAAAAACCATTAGTTTTCCAAGCAAAAAATGTGATGGTCTTGGGTCTCGATATATTTATGAAG

>CcOR41 [partial 387 bp]

AATTTATTGCACGAGATATGCCTTGTGGAAATATTTGGCGGTACTGCGATTTTATGTTTGACAGGATACTACTTTATACTCGATTTAGAAAAAAATGACGCCATGGGCATACTGACATACTTCGTTATGATAACTTCGCTAATAGCGCTGCTGTTCACCTATTGTTATATCGGCGAATTGGTTACCGACAAGGCGAAAAAAGTGGAAACCAGCTCTTACATGATCGACTGGTACGAGTTACCGCCCAAGAAGGGACTTTGCATTGGCTTGATAATTTCCATCTCGCGTTACCCGACTAAACTCACCGCTGGAAAAATCCTCGAACTATCCATGTACAACTTTGGCGGCATAATGAGATCGGCTGCTAGTTACTTGAACTTGCTACGC

>CcOR42 [partial 225 bp]

AAAAACATCGATGGCTACAACTATGCTGTTGGGCCGTGTCGATTATTTTTGTACTTACTTGGTGCTTGGCCGGATCCTTATCACGACAATACATGGGCATCTAATGTGAGGATTCTGATATCGACGCTGACGATGTTTATATTCGCTACTTTCTCGCAGACGACCAAACTTTTGCTCTCGTTCAACGACTTGAACATGGTGATTGAAATTCTTTCCAATTGCGAT

>CcOR43 [partial 216 bp]

CATTGTTTTGGACTGATCGCTATTGTCAAGTATCGATTGCAAATGCTTAATAAACCAAGGAAAACAATCGACTCTAAAACTGAGGATGCACCGTACAATCGATTAGTCGAAGCCATAAGCTTTCATATAGAAATATTAAAATTTGCAAGCTTGTTGGAACGAACGTTTTCGCGGATGTTTCTCATCTTGATGGGCGTGAGTCTTACGTTCTGCAGT

>CcOR44 [partial 396 bp]

AATATATTCTGCTCAATTCAATCGAACAAGATCAAAATTGTTTTGGACAAAGTCCAACGAGACTTTCAAATCTTTAAAAACGAAGATATCGAAGTGATTCACGAATATTCGACGAAAGCTTACAAAATCAATACATTTTATACGATTTATATGTATGTAGCAGTAGCAGCGTATAGTATTTTACCATTAACGCTACACACAGCCGACACACTATACCCTTTACCTTTCAACAAAACGCGTCTGCAAGGGAAACCCCGATTAACAACATTTTTCAACGAACAACTCGACAATAGTAATTTTTTTATTATATGCCATGGGATGGTAGTTGACACGACAGCGATAGTATTCATTATAGGCTTCGACACGTTATATTTCGCCTTGGCTTATCACGCGTGC

>CcOR45 [partial 492 bp]

GCAGAAACTGTCGAAGATATTTTTAATATGGTATTCTTGGTGCAAATGCTTACATTAACTATTCAAATATGTTTGCAATCGTTTCAATGTATAAATGTGAGTGATCATTTTTTATATTTTTCGAAAAACGAAAACGATGGTCAATTCAAATGAACATAAATTCTAATACAGGCGTTATCAAGCGATCAAGGAGCATCACTTTTGCAAATATGTTCTGTAATCCTGTACATGAGTTGCGTCATACTTTTGTTTTACGTTTATTGCTACGTTGGTGAACAACTTATTGTCCAGACGTCAGAAATTAGAATTGCCGCATACGAGTGCGAATGGTACAATCTTCCAATAACTGAAATAAAAGCATTGTCATTAATCATTCTACGTACGCAAAAACCTCCGAGTATTACGGCTGGAAAATTCTGTCCTTTTATAATGCGTACATTTAGCGAGGTTCTGAAGACGGCCATTAGTTATCTATCAGTTCTACTAACCATC

>CcOR46 [partial 132 bp]

CTCATTGTTCGATTGCAAAGTCCGTTGTACATCAACGCATTGGGAATTATGATCGTTCGATTAGACATGTTTGTCAAGATAGCCCATGCGTCGATTTCGTATTATTTTCTAATGAAAACTATCGAGGAAAAG

>CcOR47 [partial 831 bp]

GCTATTGTTTCAAGTGAAAATCCTGTAATGCTGATGGATGGCTTGAGCGATTTATTTGCTCAAACTATAATACTCATCAAGATATTCATTATGTGGAAAAACAAAAGGCTGATCGATGGTCTGCTAAAAGATATATTGAAAGATTGGAAAATTAGAAAATTACCAAAAGAATGGCAATCAATTGCTCGGACTTGCCTTCTCTTTTGTAGTATCATCATTGCAATGTACGCAATTGCCACGACGGTTTATTTTCCAGATCTTGTATTATCTTACTTTGGACAATCAAAAGAGCATAGAAAATTATTCTTCGCGTCGAAATATCCTTTCGATTATCATCCTTCTCCTATTTACGAAGTTTTAGTTCTTGTACAAATCATACAATGCTTACTTATTGTTTCGGCTGATTTAGTGAGCCAAACTCTCCTTGCGGCATTGATTTTACATACAAGTGCGCATATGGCGCTATTCAAAGATCACCTAAGAAAGTACACGGACAGTATTGTGCTTAGTCGAAAAATAAACAACGGCAAACAAATAGATGATAAACGAGATCGTTTCACGTTCCTCTTAAAACGTATAGTGAAGCAACACATGAAAATTTTAGATATAGTCGATAGAATCGATGCTGTTTACTCTTACGTCTCGTTGTTCCAAATACTCTTTAGTAACGTCATTATCTGCGTCACAGGATACGTCTTGATTACCGCTGTTAATCTGACCAACATGTTGTATCTAATTAAATTTACAATGTTCATTCTGGTCATGTTCTTACAAGCCTTTACATTTTGTTTCGCTGGACAATACTTGCAGAATAAGGCTGAGTTACTCGTG

>CcOR48 [partial 132 bp]

ATGACTACTGATGTTTTTATTAAACATCATCAGATTAGTTTACGAGTGGTTGGAGCTTGGCCTACGGCACCTTTACTACCGGTATTCTACTTCGGAATAGGAATAACATTTTTCTTTCTAATTTTTGAAGTA

>CcOR49 [partial 201 bp]

GTGTTCCAGTACGCCTATTACAGCCAGTGGTACAACCTGTCGATCAATTCGCGCAAGATGATCATGATCATACTGCAGAACAGCATGAAGCCCGTCGCTCTGTCCGCAGCCTCGATATGCGACTTGAATCTGGAGATGTTTGCTTCCGTTGTCAAAACGTCGATGTCTTACGCTACGGTCATGCTCTCGATGCAACAGCAA

>CcOR50 [partial 255 bp]

TTCAGATATTGGTCGGACGTACACGAAGAGCCAGCTTGGAGCATTATCACTTTGTGGCAATGTATTCTCGCTTATTACATACCAGTAGAGATAGCTAGCCTCGAGACCGTTAGTCTTTTTCTTATAATGATGGCTAGTGGATATTTAAGGGTCGTCCAAGAACGATTACTCAACCTCAATGACAAATTCGATTGTACAATAGACGAAAGCCGCAATGCTATTTACGACGAGGTGTTGACGTGTGTTAAATATCAC

>CcOR51 [partial 306 bp]

CAGAACAATATTATAAGCGTTGAGTTCTTGGGATACGCTATGTACCTATCGTGTATATTTGCTCAAGTATTTCTATATTGTTGGTATGGCAATGAATTAAAACTAAAGAATGAACTTATCGTGAAAAACATTTGCGAGTGCAATTGGATAGGTTTGGATATCAAAGATAAAAATGCCTTGTATTTTATTTTGCTGTTTGCCAATAAAGATTGCCGTATGTCTTGGAAAGGACAATGTCCTTTGTCCATCAAAACTTTCGTCTGGATTTTAAAAACATCGTATTCCGCCTTTAGTGTGTTGCAACGT

>CcOR52 [partial 603 bp]

TCGTCCATCGATGCTTTGTTTGCTACTCTTGTATTGCATCTCTGCTCAAGACTCACACTTTTGAAAAATGATATCAAAACATTGCCAATGTGTTTCACCAACAAATGGAATGGTGATTCTTACGATAAACTTCTTTCTCGTATCGTTGACGATCATATATTTCTTGAAAGATACGCTAAAAGTATCGAAGACGCCTTCAATCTTATGTTTCTTGTGCAGATGATAGTCTGTTCAGTGATAATATGCCTTCAAGGTTATGAAATTATTATGATTTCAACTAAAGGTGGTGGCTTCTCACTTTTTGAACTCATTTACATGACGTACTTTATACTGTGTTTTATGTTCAGTTTATTTGTATACTGCTTTGTATGTGAAATTTTACGGAAGGAGTGCATGGAGATCGGAGACGCAGTTTATAGCATTGACTGGTATACCTTTCCCGTGGAAAATGTAACGCCTCTGATGATGATTATTGTGAGAGCAAAGAGACCGTTTCAGATAACTGCTGGAAAATTCGTAGATTTTTGTTTAGAATTATACTGTAGTATATTGAAAACATCGGGTGGCTACTTATCGATGCTACTGGCAGTAAAAGATAGAGTA

>CcOR53 [partial 369 bp]

CAATTGTTCGCTATTCCGTTTACCGTGCTCACGTTGTGCGGAGCATGGTGTCCAGAAAATTGGTCGCACGAGAACAAACGTATTTACAGTGTTTACACGTGCTTCATCATGATCTTGGGTATAATTTTTTTCACCGAAATGATGATCAATATCGTCTTAACGTTTAACTCGGATAATTTCAACATGGAAAACATATTTACCGCAATTGTCGTTGGAGTTGGTATTTACAAGAAAATTAACGTATTACTTTATCGCGCAAACATTATGAATTTTATCAACGAGTACGCGACTAATCAATGGTACAAGCCAAGAAATATCGAAGAAACTGCCATCTACTCGGCAAACTTGTCCGAACGAAGGCGAGTGACC

>CcOR54 [partial 363 bp]

TTTTTCCTCTGTATGGTTGGCTACAATACGCTTATGAACTGGGATAATCGCGAGAGTGCCATGGAAAAGATACTCATGACGACGTTTGCCGGTCTCGCTGCGAACATGACGTTTCTTTTGTACGCCTACTGTTACGCGGGACAATGTCTCATTGACGAGAGTTTATACTTGTTTGACGCTCTGTACGATAACTCTTGGTACAAACTCAATGCAATTGACATGAAGTATTTTGTGATATTTTTAAATCAAACGCGACGTCCATTGGTCATCACCGGAGGTGGATTTTTTGTATATTCACTCGAAAATTTTCTGTCTGTCGTGAAGTCTGCAATGGCTTATTTATCGGTACTACGTACGGTGCTG

>CcOR55 [partial 669 bp]

TATTCCAAACGTTGGATTGTTCACAACAATGCACAAAGATTTCAAGAGATCTTACACATTTGGTCGGAAGTATGTTATTGCGCTATTCTTTATATAGCCGTGATGGGTTTATATTTATGGAGTATTCAACATGCTTGTGGCATGTATAGCATTGTTAGTTACAGATTGAAAAAATTAAGGGGTACTTCACCTACGAAGAGACACGATAGCAAAATGATGCTAGACTTGGTGAACGTCGTAGCTAAACATCAAGCGGCGATCAAATATACCGACGTCGTTAGAGAAACTTTTGGTCGATTATTTTACGCGTTACATCTCGGTTCACTAGCCTGTTTATCGCTAATGTTACTTGCTTTACTTTTGAGCTATCGTACAAGCAGTACAATCTCAATACTACGAACGTTAGCCTCGGGAGCGATGTTCTTCCAGTACATGTTCTATTTAAATTACGGTGGTGAAAAAATTATCGAATCTTCCAATCAAGTTTTCTTGGCTACGTATTACGTACAGTTGGATACGTTGTCAACGGATGCAAGAAAATTTTTACTAATGGTCATAATGAGAAGTTCGCGTACGAACAATCTCACTGGTGGTACAATTACGCTTTCTTTTGAGAATTACACAAAAATCATCAATAGATCTTGGTCGTTCGCTACTGTATTGTTAACC

>CcOR56 [partial 444 bp]

TATGTTGCTGCACCAATCTATGTTTGGAAAGTCAACCAACACAAGTCTAATGGTTCGGATTACGAGAGGAACTTACTATTAGACAGTCGTTACTGGTTTGATCCAAGTCCAACGCCAATTTTTGAGGTAACCTACACCTACCAGGCGATTGCTCTGTATTCTGGAGGTACCAGTGCGGTCGGCTTGGAGGGATCGCTGATGGCAATAATTATGCACATCGTTGGGCAATTCAGACTTATTAGCGTTAGGCTCAAATTTATTGGAAGCGCTTTACTCGATGAAACAATGGCGATAAAGAAATTCGATCCAGGAGCGGAGATCCAGGATTGCGTAAAGCATCACGAACGTATGCTCAGAATAGCCGACGAAATGAACTCCTTGTTTGGGCCGATAATCCTCGGTCAATTGATGCTAGCCAGTGTGGAGATCTGCTTAAACGGATAC

>CcOR57 [partial 216 bp]

CTAATTCATTTTATCGCGACTTTCGGTTCAAACGATTTTAAATTGTTAAACTTTTTTTTCGTGAGTGCAAACATCACGGCAGTTTACAAGTCGTTCCAATTACGAAGGGATCGTAATTCAATAAAGCGTTTCATTAGGCATTACTTCAACAACGAATGGTTAACGTGTACAAATTCAGAAGAGAACAAAATTCACGACGACATCAATTTAAGAGTT

>CcOR58 [partial 129 bp]

AGTTTCCACTGCGAGTGGTTCAACAATTCGACGAGAACCAAGCAACTCGTCGCTATGATGATGATGCGCAGCACGGTGCCGTGTTCTTTCACCGCAAAGGGACTTTGCGTCCTGGACTTGGAGACCTTC

>CcOR59 [partial 657 bp]

GAATTCCTGTTTATCAGCCAATGCTTGGGCATGTTGTTTGCCGCGTTATTCGTGATCGGCTTCGACAGCACTATAATGACCATCATAATGCACCTCATCGGTCAGTTCAGGATGATAAGTCTGAGGTTCAAAGTCATCGGTCGGAAGATTCGCGATGGCGCGAAATTTGATTCAAAATGTAAAAATAAACTGTGGCTAGAGATGGAGTCATGCATACGGCATCATCAAGACATTCTAAATCTGTTTCAGTACGTGGACGAACTAATCAATCCAATTACTTTCGGTCAATTACTCTTGGCGGGGCTGGAAATCTGTCTGAGTGGCTTCGCGATAATCGAAGGTGGCAACGGTGGTACAGACGTCGTCAAATTCAGCTTCTTGCTCGTCTCGATGATCATGCAGCTGATGATTTGTTGCTGGCCCGGCGAACTGGCCATACGCGAGAGTACGAACATCGCTCGTGTGGTGTTCTACGAGATACCTTGGTCGATGCTCACCTGTAGCGAACAACGAAACCTTGCGCTCGTCGTTTGCAGGGCTCAAAAGCAGTGCCAAGTTACTGCCCTCGGCCTCCAAACAATGTGTTTACGAAAGTTTTGCGAGGTATGTAATTCTGCACTTTCGTACATGGCGTTGCTACGGCGTATGAAAGAAAAA

>CcOR60 [partial 882 bp]

TCGATATCCGAGGATTGGAAAACAACGAAAAAGAAATCCGAATTAGTAGTAATGTGGCAGAAAGCTAAAACTAGTCGACGTCTCTCTATAGTCTCCGTGATATTAGGCGAAGGTACAACTTTGGCATACACCGTACGTATGTTCTACGTTCTATTTTATAACGAGGAGCAAGTCAAACCACTCTACATGCACGGAAGTTTCCCCTACGATACTCAAAAAAATCCAAATTTCCAAATAACGTGGGTTCTGCAAATAATAGCGACTCTCATGTCATCGGGAATTTTTTCTGCCGTAGACGCGCTTTTCATAAGTTTTGTATTGCATTTGTGCGGACAATTAACCAACTTGCAAGTGGCATTTAGCGAGATGGGAACGAAAGGACCTATTGCTGGTCCAGCATTTTTTAAATCAATGGAGATCCTTGTTAGGAAACATCAACGAATCAACGAATTTGCCGATATCATCGAATACAGCTTCAACATAATGTTTTTATGTCAAGTATTTCTGTCGACGCTACTGCTTTGTTTACAGGGATATTTATTTGTGATAACAATAACATCTGGAGAGATTGTTATAGTGGAGATGATCTTCATGCTTTATTTCACTATTTGCTTCACCTTTAGTATTTTCGTGTACTGTTACGTGGCAGAACTGTTACGTGAAGAGGTTGCAAAATTAGGAACATCGATTTTTTATTGCAAGTGGTATAATTTACCAGCAAAAGAAGCTAGATTGCTGATTTTATCATTACTTCGTGTCAAGAAACCATTGGAGATTACAGCTGGCAAGCTATGCGTGTTCTCTTTAAATTTATTCTGTGTTATTCTAAAAACCGCTGCTGGTTACATGTCGATGCTTCTCGCAGTTAAGGACAAGTTAACA

>CcOR61 [partial 309 bp]

ATGAAAGAAACAATAGCACCCATACCTTTTCGCATCTTAAAGTGTATCGGATTGTGGCGTCCCTTGACGTGGTCTAGGTGGCTCAAGTCGGGATACACTATATTTTCAACGTTAATGTTCTTGTTACTGATAACAATTACGTTGACAGTTGTTATCGGTGTCTGTCGAATGCCAATGACCGACGATTTATTCGCTGAAAATGTTTTTCTCATGTTTGCTCTCATCAACGCGTGTTTCAAAGCCATTAACGTTTTATCGTCACGTGGTCTGTTCATTGAAATGATGAACATGATTCAGCAGAAACGCTGG

>CcOR62 [partial 237 bp]

TACATGTTCATAGCATGCATATTCTTTGTTTTCACACCAGTGGCGATACCAGCCGTTTTGGATGTCATCTTACCTATAAATGAATCTCGGACAAAGATGATCTGTTATTACGCTGAATATTTCATTGATCAACAAAAATATCTTTATTACTTAGTTCTTCATACATTCGTGGCGGTTGCATTCACTCTTGTCATAATAGCTTCTGTGGACGCTTCGTTCGTTGCTGTTGCATACCAC

>CcOR63 [partial 336 bp]

CCAATTTTTGAGATTTGTTGGATTGGTCAACTAACGTCTGGTTTAATATGCGCAAGTGCCTTTACAGCATTCGATAGTTTTTTTCTCCTATCTATGCTCCATGCATGCGGCCAACTGACCATTTTAGAATACGATTTGAAGAACGCAATTACAATAAAGCACTCATTAAAGCTATCGTTCAAGGAATCACTAGGAATATGTGCAAGAAAGCACGACAAAATTTACAGATTTATACAATGCATAGAACGCATTTCCAATAAAATTTTTTTGGTTGGAATTTTGGGATTCAGTTTAACTTTTTGTCTTCAGGCTTTTCAAGCAGCAATGATCATGACA

>CcOR64 [partial 192 bp]

CTCGTGTACGCCGTGTACGATTGTCAATGGTATAATGCTCCTCCACAAGAAGTTCGAATATTGTCCCTGATACTGTTGAAAACTCAAAATCCATTAACTTTAAGTGGCGCTAAATTATTCGAACTTTCTGCAAATAGCTTCACTACGATCGTCAAAACATCATTGTCATATTTATCAGTTCTGAGAGCAATA

>CcOR65 [partial 204 bp]

CTCATGACAGCAAGAACTTGGGAACAGTTTAGCGATAGAATGTTTCTGATACCAACTGGAATATCCGGAGTGCATAAAATTTCTTTCTTACTTGTTAACAGACAAAAGGTCGTTGATTTGGCAAATTCATTGTTGAATAGATGTTGTTTGCCGAAAAATAAAGAGGAGCTCAATATTCAAGCTAAATACGACGAGACTATCAGG

>CcOR66 [partial 303 bp]

GCAGAGCAAAATGTATTGAGATATTGGATAGGTTTAGTATTGTGCAACGCACTCATGAATATACTCAATCCAATAATTGGTGATAATCCAGATAATAATTTAATAATTCAAAGCTGGATACCCTGCGATAGACGTGTATCGTCTTGCTTTTGGATTATTTACTCGCAACAAGTAGTCAGTTGGATCGCCGCCACTATTACTAACGTAGCTGCAGGCACCATTATTCTCAATTTCATTGAAAGAATTTGCTCGCACATTCGAATATTCCAACACAGATTGACCTCTCTGCCTAATTTAGTGCGA

>CcOR67 [partial 684 bp]

CCTGTCCATGTTGAATATTTTGTGGACCATAAACAATATTACAATTATATAATAATTCACATTCTATGTTACATGATTTCGGTTTTTTTAGTTGTCATACCTTACGATATAGCATACATATTGGAAATACGACACATTACTGCTTTATTCACAATTATTATGCTTCGATTGAAGAAAAGTTCCGCAATGTTAGATTCATTTGAAAATCCGAAATATGTGTCTACAATTGTTGATGATAAAGTGCGCATAGCTATTGCAGAAGTTATTGTATTGCATAACGAAGCCTTTGAAATGATTAATTTGATCGAAGACACAAATAACGTTGGAATGGTTGGAATACTCTTTCAAAACATGATTCTCTTTGGTGCTTGTCCGTTTTTACTCTCAGTTCATTTACACAGACCGATGGAATTCAATAGATACGTTCTAATGTTCATTCTAATAAACTTACACTTTTGGGTTATTTTTACTCATGGACAGTTGATTATTGACTATAGCTCTGCGATATTTGGATCTTGTTATGAATTTCAGTGGTATTATTTATCCCATAAATCATGTAACCTAATACACACAATGATGACACGTAGCGTTAAACCATGCGAAGTGACTGGCGGTAAAATATTTATTTTATCGATGGAATCATATGGTCAGATGGTGAAAACAGGTCTTTCTCTTTTTACAATA

>CcOR68 [partial 108 bp]

CAAAGAGAAATGAGCATTTCGTACCATGGATTCTGTACGCTTACAATAGACATCTTTGTTTGGGTATTGAAAACCTCGTACGGAGCTTACAATTTATTGAAAACAGTC

>CcOR69 [partial 603 bp]

ATAAAATTGTACGAGATAAGACACGAAATAGATTCGGTGATCGAGAGCATCGTGTCCTTTTTCTACAACATTGTCGTTACCATCAAATTCGTCAATGGAATTTTCAACGAAAAAAAAATAAAGGCAACGTTTGAGAAGGTAAGGAACGATTGGGAAAATATTGTCGATCCAATCGAACACGACATTCTAACGCGCTATGCCAATTTGGGAAAACTGCTGAATTTCTTATACATCGGTGCATTTCTTTCAACCGTGGTAGCATTTATGTGTCTTCCTCTCATACCGGTATTCCTAGACGTGATTATACCTTTGAATGAATCAAGACCGAGGAAACCTCTTATAATGGCAGAATTTTTTATCGACGAACAGAAATATTTTCATTCGCTATTGGTACATGCTTTTTTCACGGCCTACTATGGTATTATACCTCTTCTCGGAACTGACACCTTTTACATGAATTGCGTGTATCATGCCTGTGGAATGCTCACCATTTTAGGCCGCCGCATTGAACGTTCGTTTGAATCGAATCACGGAAAGGAATCGCTGGAAAAAGAAAATTATCGACGTATACGAAATTGTATAATTCGCCATCAAGAAATTATT

>CcOR70 [partial 492 bp]

CTTGTCAGCTACTTGAGCACAAACATTAAAATGCTCTTTGCAATGACTATATTCGTTGACTTAGAAGACAAAAAGAACATTGAAAAGTTTGGAATATTTGCTAAAGTGCCACTACTCACGATCATTACGGTATCGTTAGCGACAATAGGCGCATTTGATCAATTCGTATGGATGGTCCAGGCGTTCTCTTACGACACACATCTAGCCACAATGTTGAGTACAAACATATTTTCCAATATTAGCTGTGTTAGTAAGGGTATTTGTCTTACGGTCGCAGCACAGAATTTACAGTCGATAATGTTTGACATTTCAAAAATGTGGGATACTTATCGGCCAAGTGGTGAATGTCGTAATAAGATCGTCAAACGGGCCAAGAATACGGTGACTTTTGTAAAAGGTTACATTTGGATGACAGTTGCGCTTATCTTCAATTTTGGCGGACCACCTCTAAAATATATGCTTTTACAATATATGGAACGTAAATCATCAAAC

>CcOR71 [partial 153 bp]

GCACACTTGGATAGTTTGCTCAATCTTTTGCCGTACATAGGAGTTTACTGCGCTTTCGCGCTACACTTGTGTATGATATTCTGGCCAGGACAACGAATCCTCGATCACGGCGTTATTCTTCGCAACGACGCGTAATTTGACAACGAACACTAT

>CcOR72 [ORF 1179 bp]

ATGGATTTGTACGAAAGCCGTTACTTTATAATCAACAAGAGTTTGTGTTCTATTATAGGACTTTGGCCATACGATTCCATTCTAAAGAGACATACTCGCAAAATATTTCCCGCGTTGTGGGTCTTTGCCTTTATCGTGCCACACGGTATTGGCGCAGTGGTCAACCAAGACGACATCGACAGGTTCATGGAGCACATCTGTCTTATTATTTTTCTTTTTTTAATTTTTGTAAAATACATAACGGCTGTTTTAACCGAGGGCAAGATGATTTTGGTGTTTAACGATATAGTCGACAACATGGAGTACATCACGGATGCCGAAGAGAAAAAAATTCTCGAACACTATTCGGAAAGGGGGAGATACGTCACGTTGGTTTACTTATTTTACATACTGAACGTTGGCGTGTGCTTTACGCTGTTGCCGCTGATGCCGTTACTTTGGAATATGCTGCATCCAGATGCAGAGCCATATCCTCGATTGTTTTTAATGAACGGCGAATTTCTCGTTAATCGAGAAAATCATTATTGGAAGATATACATTTGGGATCTTCTTACCACGTTTATACCGGGCTTTATAGTAATCGGCGTGGATACCACGTTTGCAGCTTGCGTTGAACATTGCGTGGGCGTGTTCGAAGTGAATAGGAAACGTTTGGAAAGCATCGTTCAATCGGACGATCAAACCACGACCGATTCCGTGGACAGCGATGCAGCGTACAGTCGAGTATGCGACATAGTCAGATTACACCAAAGAGCTATAAGATTCACGGACACTCTGGAAAATGCTTATTCGCAATGCTTTTTGATATTAATGCTTGGAAATATGCTTCTCATGCCTTTGTTATGCGTGATGATAATTATGAACAACAATAGGAAAATACACTTGATTCGATACGCCATACTATACACGGGTTGCGCCTTTCATCTGTTCTACATAAGCTTAGCAGGCCAGAGAATTATCGATTACAGCTCGAATCTCTTTTACTACGCCTACAACAACGAATGGTACACGACCTCGTCGAAAACCAAAAACATATTGAGACTCTTTATGATTCGATGTACCGATCCGTGTATGTTGACTGCTGGAAAACTCATGAACATGAGTATGGAAACGTTTTACTCGGTTATGAAAACTGCCGTATCATACATAACGGTTATATCGTCGTTTCGAGCGGGCTAG

>CcOR73 [partial 480 bp]

GAAATGAAGGACGTGTTGAATCGGCACAAATCTGCCGAGACAATGGAAGGATGTTCAAGTACTTTCTATCTGGAAATTTCTAAAGTTATCAGGTTGCACAAACATGCTTTACACTTTGTAGATCTTGTCGAATCGACTTATGCCTCGATGCAGATATTCATCACTGGTCTCACATTGGCGACAATTACATTATCGGAATTCGAGGCAGCGGTAAATAAGACACATCAGGATATAAGATTTCGTTTTATCATCTACGGCGCTGGTGAATTAATTCATATACTATTCCACAATTATCCTGGTCAACGAGTCCAAGATCACAGTCTCATGATTTATCAATCTTACGACTCTGAATGGTATAGAAAAGATGTACCTAATGATTGTAAAAAGCTTATTAATCTGATGATGATAAGAAGTCAGAAACCATGTTATCTGACAGGTGGAGGATTATTCGTTCTTGGTTTAGAAAATTATGCTAATGTA

>CcOR74 [partial 495 bp]

GACAATCAAAAGAACCAACCACATAGTTCGATATTTGATCATAAAAACTGTAATATTTTTCATCGCATTCTGTATGGTCACAAGTGCAGAAATACTTACCGTAAATTCATCTACTTAAATTACAACTATTTTATGAAAACTTATAATTAAATATGTATACTTTTATATTGTGTATAGCAAAAACCAACGTACGCAGGATTGACTCTATATACGTGTATTCTGATGACAGCTTTTTTAAGACTGGGAGTGTCTGCTGAGAGCGCTCAAAATATGATATCAGCGGCCGAAAATGTACAATTTGCATTATATTCTACGAACTGGTATACAGAAAAACATAAGTACATACGATCAAAAATAATTATTATGCAACGTAATAGAAAATTACCAAAAATATACCTGACTATGTTAATGTCTAATTGTAATAGACAATATTTATGGAAGATTGTGAATATTACATTTTCGTATTTCATGACAATACGAACATTGTTAAAAAAG

>CcOR75 [partial 384 bp]

GGAACGTTACCGGTGGAGATCATCTACGAAACAGTGGCAACGGTGGGCCTGGACATACGTTATTACATACTGCTGGCGAAGCGCCGAGAGTTTGCTCGACTGATGGACGCGTGTGACGAACTTTGGGACTACTGCGACGCCAATAATCAACCGTTGGTCGTTCGTTTCGAACGCAAGATGCGCAAGCTGTTCTGGCTTATTTTTGGCAGCATCATCGCCGTCGATGTTCTGTACAGCGCCACTGCGATCTTTCTAACTTACGTGTCCGACCAAGCGAACGGAACGAAAACGTTACCCTTCAGTTGGTACGGAGTTGAAAAAATCGACGAAACGTGGCCAGGCTACAAAGTTGCCTTTGCCGTGCAAGTTCTGGTCATTTATAAC

>CcOR76 [partial 783 bp]

GGAGAAGTCACGATGGATTACATCTACGAAATAGTGTTGGTGTACGGCATGTATATACGTTATTACATCCTACTGTTGAATCGTAACCAATTGAGAAAATTGATAAACGAGTGCGAGTCGTTGTGGAACGAATGCAACGACGCTGAGCATCGACTGATCGTCAAATTCGAGCGCAAAGTACGCAAGCTCTTTTTATTCCTCTTCTATTCGTCCAATTTTGTGAACGTCTTGTACATCGCTGCCGCTGTCAATCTCAATCGGCATGCCGTCACGAGTAACACCACCGATACCCGAGTTCTACCGGCAAAATGGTTCGTGGTCGACAGAGTGGACGCTTGGCCAGGTTACGAAGTGACGTTTTTCTTGCAAGTTTTGCTCATCTTCAACGCGTGCATCGTCTCGTCGATCGCCGACTCTTCCGCGCCGATACTCATGATGTTTAGTAGTGGATACTTCGCTACGTTGACGAAACGATTCGAGGACATGGTTATGACGGAGGATCGCGAACAATTCGATCGTCAGTTCGTTGCTGCTTCCAAGTTTCATCAGAAACTTTTACATTTTAGCAACGGAATTAACAAAGTCGGACAGATGTTGTTTTTATGCCAGCTGTTATGCTCTGGTTACAACATGTCGATCATAGGAATCAAACTATCTGGCACGGATCCACATAGATTCAAGTACCTTCCGAATTTGCTCACTTCCCTAGCCGAGTTGCTGATTTGCCAATACGCTCCGGATTATCTATTGACCGAAAGCGAAGCGGTTTCAACGGCTGCTTAT

>CcOR77 [partial 657 bp]

TCTCAAGAATTTCCAACTGATGCAGTGTATCCTTTTGCTGTAAACAATATTTATATAAAAATCCTTATATATTGCCATCAGTCTATTGTTGGACTTCAGACATCGGCAGCAGTTTTACTTGATTGCTTAGTAGCAGTTTTATTATGGTTCGTATGCGCTAGATTTGAAGCATTAGCAGAATCTATTGGTACTTACAGCCGTTTTGATGGAATAAAAATTCATATTCAACATTATCAAACATTACTACGATACGTAAACGACGTTAAAAAAACCATAGATTTATTTATATTGGCTACAATACTGACATCAGTTGGAGGTGTACTATTTAGTTCCATCCAGTTCGTTGTAGATCAGCCAATAGCTATTAAGGGACAATATGCTATCGTGGCAGTTACTGCAAGCTTGGGACTTTTTATATGTTCTTATGCAGCTGATACACTACTTCAATTGGGGTACCAAATTGGAACAAATATATTTTACTCCAATTGGTACACGATGGAAAAGAAATCAAAACTATGTATAATATATCTAATTGCTCAATCACAGGATCCAATAACTATGAGGGCAAATCGCATTTTCCCAGCACTATCACTACAATTCTTTTCTCAGTTTCTACAAATGTCATTCAAATTTTTTACAAGCATGAGGATTATGGTG

>CcOR78 [partial 588 bp]

TCGGAACTGGTTAATCAATCGGAACACGCTGGAAACAGCGACGAAGCTGCCGATAAAGCGTATTCGTTAATAACGAGAGCGATTGATCTACACTCGGAATGTCTCGAGTAAGTTTCAAATAACCATCGATACGTTTGAATTTATTGTATTTGTCGTTTGAACGGACTCCGTAGATTGGTTAAAATGGTCGAAAAAACTTATACGAGCAATTGGTTCATCATTCTATCGCTCAATACCGGAATGTTTGGAGCTGGTATTCACTTGATATCTTTGAGAATTCACGACTCGATCGACACTTTCCGGAACGTCTGTGCGTATTTTTTGGTTCTAATGCACTTTCACGTGATTTTTTCGCAAGGCCAAAACATCATTGACTCGAGCTCTACGGTGGCCGACATTTGCTACGACTGCAAGTGGTATGGTTTGCCTGGAAAATCGTGCAAATTATTAACTTTAATGATGATACGAACCTCCGTTCCTTGTCGAATAACTGGAGCTAAATTTTTTCCTCTCTCGATCTACACCTTTGGCAAGATGTTGAAAGCTGGTGTATCGTATTATACGGTATTCAAAGCCTAAAATTCGTAT

>CcOR79 [partial 171 bp]

AGCAGCGAGTGGTACACCTGCTCAAAGGAGACCAAACAAATTTTGAAAACGTACTTGCTTGGTTGCTCGCGGCCTACTTTGCTAACAGCAGGAGGACTGCTATTGCTCAACTTTGAGAATTACTTCAGCATAATAAAAACATCGGCATCGTACATAACTGTCTTCGCTTCGTTT

>Cc IR25a [partial 1560 bp]

ATCCTACTCTGGCTGCTCGTTGTCGGTGAAGGAGGACCCGTCACGGCCCAGTACAACGACGTTATCAATAGGCCCGTTAACGTCTTCGTCATCAACGACGCGGGCAATGACGTGGCGAACAAGAGCGTGACCAACTCGCTACGTACCCTCAAAGACAAGAATCCCGACAAGCTTGGCCAAGTATACGTTGTTCAAGTCAACATAAGTGATTCTAAGGAAACTCTCAATGCCATTTGTGATCTTTGGAAAAGTGCAATAACCGACAATCAAGATAACATACCAGATTTCGTGCTTGATACTACGACTTATGGTATTGGCGCTGAGACTGTTAATAAATTCACAGCTTTTCTTGGAATACCGACGCTTTCGGCACAATACGGACAGGAAGGTGATATGCTGGGATGGAGAGAGATTTCAGAGGATCAGAAACAATACCTGGTACAAGTGATGAATCCAGCAGACTTAATGCCGGAAGTTGTACGCGAGCAGTGCTCCCACTTCAACATAAGCAACGCAGCGATCCTATTCGACGAGAGTTTCGTAATGGATCACAAGTACAAGAGTCTACTATTAAACGTGCCAACACGTCACGTAATAGTACCTACAAAACCAGCAGGTACAGCCCTACGCGAACAGATATCACGTCTGAGAGATCTCGACATCGTCAATTTTTTCGTACTGGGTACCGAAGCAACGATATCTGCTGCGCTCCAAGCTGCGAGCAATCTCGGTTTTACCGATCATCGTTACGGCTGGTTCGGCATCAATCTCAACGAGGACTTTACGATTCAATGCCCAGACTGCAAACGCGCGCGTATCCTCGTGTTCAAACCCGAGATGTCGACGAGTCAACAACAACTCAACGAGTTGACCAGCAAAGGCGCACTGCCGAAACCTTGGATTCAGTCGGCATTTTATTACGACCTCACCAAGATCGGCATATTGGCCATGAAAGCGGCAATCGACGCGGGCGAATGGAAGTTGGATCGTCGTCGCTTCATGTTATGCGACGATTACAACGAGAACGTTACAACTCCACTGAGAAATTTGGATTTCAGGAAGAGGCTGAAGCTGGTCACCGGCGGAGGAAGCGTTTTCATGCCGACTTACGCAGGTTTCGGCTGGGGCAACAAGAATGGCGAGAGTCAAGCCAAATTCGAAGCGAATGGCGTTGCTATTCAAATCAGAGACAGTCGCGTAACGAGCGAGGAGATAGTTGAGTCTTGGCAAGCTGGTGTCGACGTTCCCTTGACCATAAAAAACCCGGGAATAGCGGCGAACCAGACAGCGGTGACGAGTTACCGCGTGGTGACGGTGATCAAACCACCGTTCATAATGCACGACAACAAAACAGGAGAGTGGTCGGGTTATTGCATCGATCTACTCAACAAAATACGCGAGCACGTGAAGTTCGAGTACACGATCGAGGAAGTGGAGGACAAGGAGTACGGGAATATGGACGAGCAAGGCAATTGGAACGGCATGGTGAAAGTACTGAAGGACAAGAAAGCCGACATTGCTCTTGGAGCTTTAGCGGTGATGGCCGAACGCGAGAACGTC

>CcIR21a [partial 636 bp]

GGCACACTGGATCACAACGGTTGGGAACGATGGTTCAATGCAAGCAACCTTACGGATAACGATCCCTTGTTGAAAAAACTCTTTCGTAAGATGGAATACGTACCGACTTTGCTAGAAGGAGTTCAAAATGCGAGTCGCGCTTACTTTTGGCCTTACGCTTTTCTTGCCTCGAGGACTGCTCTTGATTATTTAGTCCAAACGGACTTTGCACCCGCCTACTCGACCAAACGTTCCCTCATGCACGTCAGTAACGAATGCTTCGTGCGCTATCAAGTGGTTCAATTATTCCCACCTAATTCTCTTTACACCGACAAAATGAACAATTTCGTAACGCGATCCAACGAGCATGGATTGCTCGATCAACTTGTTACGGACAACGATTGGGAAGTACAACGTATCGCCATGAAGACTGGCAAAAAAGTTACCAAAGGTCTCTCGCAAACTGTACTCATGGAAGACCGAGTGCTCTCCGTCGAAGATACCCAAGGTATGTTTCTCATACTTGGCTCGGGAGTGCTCATGGCTCTACTGGCATTGAGCTTGGAAAAACTCAACGGTTGTATCGTTGCGTTAAGGGCAAGAAATAAATCAGACCGAGTGCAAGATCATGGGGTTGATTTCAATCGACCCAAACTC

>CcIR75q2 [ORF 1908 bp]

ATGCGATTGTTCCCCGGAATTTGGTTGTGGTTAATCTCAATGCGTACGAGCGGATTTATCGTCGACGGATGCGAAAGCGACCAAGCCAAAGAATTGGTCGAACTCTTGATCGAACTCAAAACCAGAGGCCTGTCGACGTTCTCAAGGGTGACAATGTTTGGATGCTCCGACGACAAATCGCAAGTCGCTCGACGACTCTCCAGAGCTCTTGTAGCAAATAGTATGCACGAGCTGGATTCGAATCAGTTTAGCTGGAATTTGCAGAAATCAGTAGCAAGTACACCGGATCACCATCTACTGATAGTTTTGGAAGTCGATTGTCCAAGTTCCGAGCGCTTGCTGCTCGAAGCCAAAAATGCAACGATGTTTGTATCACCGTACAAATGGTTCCTAATCAGTCGTGGCTGCTCGAGTGTCGATTGCGTCCTTTCGAAACTCAGCGAGTTGCGGAGGAACGTTCTACCGGATAGCGAACTGCTGATATGGTTGAAGCAGTCGCAACGTCTCGTATCCGTTTACAAAGTTAGCGCGGAACAAGGCCGCGATTGGCAAATCGAAGAAAGAGCTCGATCGATCGCTTGCGACAATGCCACGTTCAACTTGCTACTCGACGACGACTCGGCAATGATCACCTCGAGAAGACGCAGCGATCTTCGGAGAACTCATCTAAAGTCCAGTCTGGTGATTACGGACTACGACTCGCTCCAGCATCTGACCGACTACCGCAATCGCGAGATCGACACCAGCAGCAAATGCAACTATCCTTGGGCCAAATTTCTCGCGAGCATGCTGAACGCGACGATAAGCTTCAAGTTAGCGCCGACTTGGGGCTACCCAAGGGCCAACGGTACGTGGGACGGTATGACGGGTATGATGCAACGCGGTGAAATAGAGTTTGGCGCGTCGCATTCGTTCGTCACCGGCGAACGTCTCAAAGTCGTCCATTACCTCGCCGGCTTGACAACACCGTCGCACTCGAGGTTTGTATTCCGTCGGCCAGCCTTGTCATCGGTCGCCAATTTGTTCGCGCTTCCGTTTCGTGGCTCCGTCTGGATTGGAGTGTTGGCACTGGCCTTGCTCTTTGGCTTCCTGCTGTATCCCGCAATGGTGCTCGAGTGGCGTCGCCATCGAGACGGTACGATCGAACCGCGCGTCGGCGACGAGACGATCGTCGTCGTTGGCGCGCTGGCCCAACAAGGCTTCTGGTACGAAGCACGAGCTGCAAGCACCAGAGTTCTCGTGCTCGCCGGATTACTTGGTTTTCTCAGTTTACACGCGGCTTACGCGGCCAACATTGTCGCTCTGCTCCAATCGACGAGCAGCTCCATCAAATCGCTGAAAGATCTACTCGCCAGCCCTTTGGATCTTGGCGTCCACGACACCGTGTTCAATCGCTATTATTTCAAAAGTTTGGGTAGCCTCGAACCAAAGATACGTGGCGCGATATCCGAGAAATCCGAATGGTTGGGCTTGGAAGAAGGCGTGAGGCGTTTGCGTCGTGGACACTTTGCCCTGCACGCGGTACTCGGCTGGGCTTACAAGGTCGTACACGAGACTTTCGAAGAAGACGAAAAGTGTGACTTTGAGGAAATTGACTTTTTGAACGTGTTCGAGCCTCATATGGTTGTCGCCAAGTTCAGCCCTTACAAGGAATTATTAAGGGTCAAAGCATTGCGTATACGCGAGACTGGCATGAGAACGCGAGAAATCAACCGATTGTATTCGACGAGACCACGATGCGACGCCGCTGCTAGTTCCAGACGATTCATCAGTGTTGGCTATGCCGAGTGTAAGGGAGCGTTCTATGCTCTCGGTTACGGGCTCGTCGTCGCTTTGCTACTGTTTGCCGCGGAGATCGCACATCGGCGTTTGCGACTCGGTATCCGCAGTAACCGGCTGAAGCGGTAG

>CcIR64a [partial 639 bp]

CGTATAGTTTTTCTAACTATGTTTCTCTGGTTACTTGTGATGTTTCAGTTCTATTCAGCGAGTATAATAGGTTCCTTACTCGCACCGCAACCAAGATTCATCAATACTTTATGGGATTTAGCCGATAGTGACCTAGATTGTGGTTATGAAGATGTTGCGTACATGCGTTTCTCTTTTGCATACAGGAAAGATCCGGATAGAGTTTACCTTCATAAGAAAAAATTTCAACCTGGAAGGAAGAGGAAACTCTGGGATAATCAAAGAGATGGCATGCACGAAGTTAGTAAAGGAGGCTATGCTTTCAGCAGCGAAACGTCTCCAGCTTATCACGTAATTGAGAATACCTTCACGCAAGAACAAATCTGCGAGGTCACAGAATTACCATTATATCAAACACAATATGTGAGCATCATTACCAGAAAATATTCACCATTCAAGAAAATGGTTATTTATGGAATACGTCAAATGGTTGAAAAGGGAATAGTGAATCGTTTGATAAAAATATGGACTCATCGTCAACCGCAGTGTCCAGAGAGTTTTCGTTCTCGACCAGTACCAGTTGCGTTAATGGAATTTGCTCCTGCAATTTGTCTTGTCTTAGCCGGTTTGATTATTGCTATTATTATTATGGCGGGTGAA

>CcIR93a [partial 2595 bp]

ATGATGTTGTCGCTGTTGCTGCCGTTTTTATTGAGACTTGGATGGACCAACGGATATAATGATTTTCCATCGTTGATGACTGCTAACGCTACTATGGCTGTCATAATTGAAAAAGGATTCTTCAAAGGCCAGGATGAGTATAGAAATGCCTTGACAGACATTACTGATATAGTCATCGGTATTATAAAGAAGAATATGAAACAGAGCGGGATCGATATATTTGTGTTTGGAGATACAAATATCAATTTGGGACGAGATTATACGATTTTACTTTCGGTGGCAACTTGCCAGACAACTTGGAACTTGTTCAAGCGGGCGCAGAAGGAAAAGCTTGTACATCTAGCTATAACTGGTCCCGATTGTCCAAGGCTCCCCGAGAGCGACGGTATAAGTTTACCGTTTATTGATCCCGGCGAGGAATTGGCTCAAATCTTCCTCGATCTTCGAATGTCGTCGGCTCTTGCCTGGGCCAAAGTTAATTTCCTTCACGATGACACTTTTGATCGCGACACAATCAGCCGCGTGGTGAAAGCATTGTCGGTAGAGTTGCCGAATAAAAAACTACTACTATCGACGCGTGCCATCTTTTCTGTACAATATGACAAAAGTGACAGCGTTATGAAACAACGCATTCATAATATGCTCGCCGATTTTCATGTCGATCAATTGGGTAGTTGTTTTATGGTCATTATTACAATCGATATGGTATCGTCTATGATGGAAGTTGCAAAGTCGTTAAAAATGGTACACCCAAGAAGTCAATGGCTATACGTCATAAGCGACAGTGCAAGCCGAGACACCAACGTCACGATGTTCCTCGACCTGCTCACCGAGGGCGAAAATGTCGCGTTCATCTACAACACTACTAGCCTTGACCTCGAGTGCAAACTGGGCTTGACTTGTCACATCAAAGAATTCGTGCGCGCATTGGCGAAAAGTTTAGAAAACTCTCTGAAAACTGAGCTTGAACTTTACGATAGCGTTACCGAGGAGGAGTTTGAAGTCGTCAGATTGTCAAAAGCCGAGCGCAAAGACGAGATCATTAAGAGCATAAACAGAGAGCTGTCTGAGCTACGTGCAGGCACAACCAACACCTGTGGCGAGTGCATCAGTTGGAAAATTGCTTCGGCAATTACGTGGGGTGCATCGTTCAGTATCAACAACGAGGCAAAACAACAAGAGCAAAAACAAAAACAAGAACATCATAAAAACAAAACAGAACTCATAAAGAAAAGTGTTGGCGAGCTTGTTGATTCGGGTACCTGGAGTCCAGCACCTGGGATCAAAATGAACGAAATGTTATTTCCTCATATTCAACATGGATTTCGTGGAAAGTCGCTACCTGTATCAACATTTCATAATCCACCGTGGCAGATACTATCATTTTCAAATACTGATAAACCAGAATTCAGAGGCTTGGTTTTCGATATTCTTAATTATTTGAGCTTGAAACTGAATTTCACATATATGGTACAATTGCCATCAGGATACGATATGTTAAGTAAATTCCAACCAAATAACACTACCGTTAAAGGCAAGAAAATCAACAAATTCGATGTAAACGAAGCAGCGATATCGGTAGCACGTAAAGTGCCAGCTGAAGTGATCGAACTTGTGGGAGATGGTCGAGTATTCCTTGCTGCAGTTGCTACTACGGTTAGCGAAAATACGAAAAATGTCAATTTCACTTACATGATCGCGCAGCAGGCGTACGCGTTACTGTCGGCAAAGCCAAAGCCTCTATCACGAGCTCTTCTTTTTATGGCACCTTTCACGTCCGAGACTTGGGCATGTCTATCATCGGCACTTCTCTTGATTGGCCCATTCCTATACGTGATGGTAAAACTAAGTCCAAAACCAATTGAACTTAACGAAGTTGTTGGTCTTTCAACGACGTGGCAATGCTCTTGGTACGTGTATGGAGCACTTCTTCAACAAGGTGGGATGAGTTTGCCGAAAGCGGACAGTGCACGTTTGGTAGTAGGCACTTGGTGGATTGTGGTGATGGTTGTTGTAGCTACGTACTCTGGCAATCTCATCGCTTTTCTTACTTTTCCACGAATGGACGACCCAATTGACACGGTTGACAATCTCATTGCTCGACAAAATCAATTTACTTGGGCTTATCCAAACGGTAGCGCTTTTGAGAATTATCTTATTGCTGCTGCTCAAGACACTGAAAAATATAAAATCCTGTTAGACGGCGCTAGCCAAGAAGATCCAAGCGAACCGAAACGTGTACTTGCAAAGGTAAAGGACAGTAATCACGTTTTGATAGATTGGCGTACGTCCGAGGCATTTCTCATGCGACTAGATCTTATGGATACGGGCGTTTGTAACTTTCACGTTGGCACTGAGGATTTCTTGCATGAAAATATGAGACTCATTATTGCTGATAATAGCCCTTATCTTGAACTCGTAAATGCGGCAATTATAAGAATGCACGAATCAGGCTTGATCAACAAGTGGTCTTTGGACATACTACCTCTAAAAGACAAATGCTTCGTAACGAAGGGGAACCAGGAAGTTACTAATCACAAAGTTGACATGGGCGATATGCAAGGAATATTTTTTGTACTAGCCATTGGATTTACA

>CcIR8a [ORF 2751 bp]

ATGTCGCGCGCTCGACGTATCAACGTACAACTTGTTTTCTTACTCTCGTTTGGACTTGCTCTCGTCGCTTCGCAAGCACCCGTTACCCTACTACTGGTAATCGAACAGCCCGATGCCGAGATCATGGGTAATTTGAACGACGTAGTGTCCGAAGCGGAGAGTCAATTTGGCGCGAATCTCATAAAGATCGACGTTAAACTGGTACAAGTGGATCGCGAGTTCGTCGACGAAAACTACGATAGAGTGTGCGCCCACTTGTACAACGGCATCACGATGATCCTGGACATGACGTGGACCGGTTGGGACAAACTGCGAGACTTGGCTCGCGATTTCAACATCATTTACAAGCGTGCCGACACCACGATCAGCTCCTACGTACAAGCTGTCGACCAGACAATGATGTACAAGAACAGCACGGACGCGGCCCTCATCTTCGAGAACGAAAAGGAGCTCAATCAAACGCTCTATTATCTTATCGGCAACTCGATCATTCGTTTGGTGGTCATCGAATCGTTGACGGCTCGCGAAGTCGACCGTATTAGCAACATGAGACCCTTGCCCTCTTATTACGTAATCTACGCTAGAACTAAACAGATGGAAGAGCTCTTCAAGACGGCGCTCGAAGGTGGTTTGGTGAAGCGCGACATGGTTTGGTACTTGGCGTTCACCGACAACAACCACGCCGATTTCTCTTACTTTCGAGACTCGAACAACCCAAACGTGAGCGTCAACGTGTTCACCATGAAGGAAGAAGTCTGCTGCCATCTCATGTACACCGTGGCACCGTGCAATTGCCCTGCCGACTTTGTGATATTCCATCACTATTTCCGACGACTAGTCCAGCTGATCGTCGAAACGATGAGCGAGTGCCAGGCAGCGAACAGGTTGCAAGAGCCGCAAAGCGGGCAGTGCCAGAACAAAAATGCCACCGAGAACTCGGTTAACGCCACGCTCTCGGAATTCGACAAGAGACTATTGGCCAAAATCGAGAAAAACGATACGTTCGAGTACGTGACGGCAAGGACCTTAGTCACGTACAAAGCGGCTGCAGATTTGAAACTATTGAAGAAAGGACAGCTCGAAACTATTGGTACGTGGTCTCGCGACACCGGCATTCTGCCTCTGCCCAACAAGACTATTCAAGCGGCACGTCGCTATTTCCGTGTCGGCACCGTTGATGCGATCCCGTGGACCTACAAACAATTGGACCCAGACACCAACGAGCCGATCAAGAGACCCGACGGAACCTACGTCTACGAGGGTTATTGCATCGACTTGATCGACAGATTGGCCGAGATGATGGACTTCGACTTTGACCTGGTGATACCGCAGGACGGCGAATTCGGCCAAAAGGTCAACGGGTTTTGGAACGGCTTGGTCGGCGACCTGTCCAAAGGCCAAACCGACATTGCGGTTGCCGCGCTCACGATGACGTCCGAACGAGAGGAGGTCATCGATTTCGTCGCGCCGTATTTCGAGCAATCCGGCATATTAATCGTGATGAGAAAGCCGGTGCGCAAAGCGTCTCTCTTTAAATTCATGACCGTGTTGAGACTGGAAGTGTGGCTGAGTATCGTCGGTGCGCTGACGCTTACGGGAGTGATGATATGGGTGCTCGACAAATACTCGCCGTACAGCGCTCGAAACAACAAGCACATGTATCCTTATCCTTGTCGAGAGTTCACCTTGAAAGAGAGCTTCTGGTTCGCGTTGACTTCCTTCACGCCGCAAGGTGGCGGCGAAGCGCCGAAAGCTTTGTCGAGCCGTACTCTCGTTGCCGCCTATTGGCTGTTCGTCGTGCTAATGTTGGCTACGTTTACGGCCAATTTAGCGGCTTTCCTCACCGTCGAGAGAATGCAGTCACCCGTGCAATCGTTGGAGCAACTGGCGCGCCAATCGCGAATCAACTATTCCGTGTTGGAGAACTCGACGATACACCAGTACTTCAAGAACATGAAGATGGCCGAGGAGAGGCTCTACCAAGTTTGGAAAGAGATCACTCTGAACAGTACGAGCGACCAAGTTGAGTTTCGAGTTTGGGACTATCCGATAAAGGAACAATACGGCCATATATTGCAAGCGATCGCCCAGGTTGGCCCGGTGAAGACGATCGAGGAGGGTTTCCAGAAGGTCGAGGCCAGCGAGAACGCCGAGTTTGCTTTTATCCACGACTCGTCCGAGATCAAGTACAAGGTGACGCAGGACTGCAATCTCACCGAGGTAGGCGAGGTGTTTGCCGAGCAGCCCTATGCCATTGCCGTGCAACAAGGCAGCCATTTGCAAGAGGAAATCAGCCGAAAGATTCTCGATCTCCAGAAAGATCGTTACTTCGAGCAACTCAGCTCCAAGTATTGGAATCAATCGCTCAAGGGGAGCTGTTCGAACGCCGACGACAACGAAGGAATCACCCTCGAAAGTTTAGGCGGTGTTTTCATCGCAACTCTGTTTGGGCTCGCACTCGCGATGATAACGCTTGCCGGTGAAGTGATTTACTATCGACGCCGTAACACACGCCAAGACGACTCGCTGCAACAGTCCAAAAACGGAACCAACAACTCTGGTTCCATAAGCGGTTCCATTCAAGACGTCAAACAAACCAAAGAACTCGATATTCAAAAGCTCGCGGCGAGGTTGCAATTGAAGCCAGCACCGCCCGTTGCCTTTGAACAAAAACCTAGCAGCCTTAATACAACTAAACCACGCGTCTCGCATATATCCGTTTATCCACGACCTTTTCCTTTCAAAGATTAA

>CcIR1 [partial 1242 bp]

GCTTTAGCGGTGATGGCCGAACGCGAGAACGTCATAGATTACACAGTACCTTATTACGATCTCGTCGGCATATCAATTCTACGGAGAAAGCCCAAAATTCAAACGTCACTTTTCAAATTTCTCACCGTCCTCGAGACAGACGTCTGGCTGTGCATCCTCGGAGCTTACTTTTTCACCAGCCTTCTAATGTGGCTCTTCGACAAATTCTCCCCGTACAGCTATCAGAACAATCGCGAGAAGTACAAGAACGACGACGAGAGACGAGAATTCACGCTCAAGGAATGCCTGTGGTTCTGCATGACTTCCCTCACGCCACAGGGTGGGGGAGAAGCTCCTAAAAATTTATCAGGTCGACTTGTTGCTGCGACCTGGTGGTTATTTGGTTTTATCATCATTGCATCGTACACCGCCAATTTGGCTGCTTTCTTGACTGTCTCTAGATTGGACGCACCCATCGAGTCTTTGGAAGATCTTAGCAAACAGTACAAAATTCAATTTGCTCCGGTGTTGAACTCGTCCGAGTATCGTTATTTCGAAAGAATGGCCGCTATTGAGAAACGATTTTACGAGATTTGGAAGGACATGAGCTTGAACGACAGTTTAAGCGACGTGGAACGTTCAAAGTTAGCAGTGTGGGACTATCCAGTGAGCGATAAGTATACCAAGATTTTTCAGACAATGCAAGATACTGGTTTCCCTAAGACTGCGGAAGAGGCTATTGAAAAAGTGAAGAAAGGCGAACCAACGGAATTCGCGTACATCGGTGACGCGACCGACATCAAGTACCTCGCTATGACCGACTGTGATTTCATACAGATTGGCGAAGAATTCTCAAGAAAACCCTATGCTATTGCGGTGCAACAAGGATCACCGCTCAAGGATCAATTTAATAACGCAATTCTACTGATGCTGAATCGTCGTGAATTGGAGAAATTGAAGGACACATGGTGGAACAAGAATAAGGAACGTAAAGCTTGTGGCAGGGACGACGATCAAAGCGACGGCATAAGTATTCAAAACATCGGTGGTGTTTTCATCGTGATTTTCGTGGGTATTGGATTGGCTTGTGTGACTCTTGTCTTTGAATATTACTACTATCGCTACAGACCACAAGCACGTCAGAAACATCAACAGCAAAGCCAACGTAATAACAACGCCAAAAATCCAATATCCAACATCAACTCGATGAAATTTAATCTTAGACCTGCGCCGACCCAACAATTGGAGCCGAATAATCATCGA

>CcIR75q.2 [ORF 2043 bp]

ATGCATCGATACATACGGCAACAATGCGTTTATGTCAGTAATAACATCAATATGAAGCGTTGTACCAACTCTCTTTGGAAATGTCTTCTATTTTCACTGATTCGATTAGAAATCCTTCGAGTTGGCCAAGTCAGTTGTCGTCATATTGACGAATCAATATCGAATCTTGTTGTCGAAGTTTCTACCGTCTCGTTTTTTCCCTCGGCGGCAATAAGTTCTCTACGTTGTAGACATTCAGACGATAATGTCATCTTTTCAAGGATTCTTTCCGAAAATCGTATGCTTCATAATCATCTGAGTTTCGACGATTTTCAAAAAGATGATTTTCCTTTGCACAAGATTGTAATTTTTCTTGATTTCAAGTGCCATGGAGCAAGAGAATTTCTGCTGAAAGCTAATAGTTCGGAGATGTTTTCAGCCCCTTATAAGTGGATCATATTTCAAGATCTCGAACATAGCTCTCCTGACAATTGTACAGATGGATGTGCGTTCAAAGATTTTTATAGTTATGCAATGTATCCAGACAGCAGCGTCGTCATTTTACAAAAATTGTCAAAAGAACGTGTACAAATTGTATCTATATACCGACCAAGTCCAGTACGAGACATGATCGTTGAGAATCTTGGATATTGGAGTAGCACTAATGGCACTAAGTGGCATAATTTGAACATTGCATCACAAAGACGAAAGAACCTTCAAAAAACTCCCTTGAAATCGAGTATTGTGGTCACGAATCCAGATACGTTAAACCACTTGACCGATTATCACGACAAGCACGTAGATACTATCACAAAATGCAATTTTGTGTGGCTCCATCAACTTATCGATGCAATGAACGCAACGGTGACGTACAGTATAGTTAACACCTGGGGATATCGCGATAAAAATGGTTCTTGGACTGGGATGACTGGCCAACTGAGCCGCAAAGAGATCGACATTGGAGGAACATCGATGTTCATCATCGGAGACCGATGGAACGACGTACATTTCATTCCTTTGTCAACACCTACCAGACAAGCGTTCATTTTTCGACAGCCTCCCCTGTCGTTCGTCAGCAACTTGTTCACTCTACCCTTCCGTCCGTCCGTATGGATAGCGATAGGTATCCTTCTGATGATAATTTTTGCAATGCTCTTGCTAGCTACGAAATGGGAGTGGAGAAAGGTATACGCCGACCGAGAATTCAGCGAGAATGAGCCTAAACCAAATTTGAGCGATCAACTGTTGTTAATACTAGGCGTTTGCGCTCAACAAGGATTTGGACGCAGTCCGTATACGGTACCATCTCGAATAGTTCTCTTGATGCTCCTGTTGGCCGTGTTGAATTTGTACGCTTCGTATTCGGCCAACATCGTTGCTCTTTTACAATCTACAACGACGTCGATAACGAGCTTGAAAGATCTACTGGAGAGCCCTATAAAATGCGGAGCCAACGATATCGTTTACAACCGTCATTATTTCAAACTGGAAAAAGATCCGGTGAAAAGAGCTATTATCGACAAGAAAATCGAGCCTAAAGGCAGTAAAGCTAATTGGATGACTGCCGATGAAGGAATCAGTCGAGTGCGACAGGGCTTCTTTGCTTTCTTAATAGAAACCGGTCCTGGGTACAGGATACTTCAGGAAACCTTCGAAGAGGATGAAAAGTGTGGATTTCGGGAAATGTATTTTATCGATCACTTTGATCCAATGTTTGCCATCGTCAAACGTTCGCCTTACAAAGAGCTGATCAGAGTCAATTCCTTAAAAATTTGGGAATCAGGATTAAAATCCAAAGAAATGTCACGTTTGTATACGAAAAGGCCCCCTTGCAATGGCCGCAACAAGTTTGTTAGCGTTGGCTTGAACGAATGTTATTTTGCATTCTACATCATTGGTTACGGAGTACTATTCGCTATTTTAGCGTTCCTCGTCGAAATTCTATCGAAGAAAAGTGGTTCTCTAAGAAAACGTCAACCCGTTGAGTCGACTGCACGGACAAGTTTTGCACAACGCAATTTGCAACAAAACAGTGCGCGCGAATCACCGTTCTCCGCTTCGTAA

>CcIR76b [partial 210 bp]

TTTCCGTCGCATATTACGGTCACAACGTATTCTGATATGCCCTACTCAAGATATAGAAAATTAGATAATGGCACCTTTGTCGGAGAAGGATTTGCTTTTGAATTGTTAGCATTATTGATGAAAAAATTTAAGTTTACCTACACAATCATACCACCAGCCAAAGATATTATTGGAGACGAAAGTAGCGGCATGATACAACAATTATACAAT

>CcIR75p [partial 282 bp]

AGCGAGAATGAGCCTAAACCAAATTTGAGCGATCAACTGTTGTTAATACTAGGCGTTTGCGCTCAACAAGGATTTGGACGCAGTCCGTATACGGTACCATCTCGAATAGTTCTCTTGATGCTCCTGTTGGCCGTGTTGAATTTGTACGCTTCGTATTCGGCCAACATCGTTGCTCTTTTACAATCTACAACGACGTCGATAACGAGCTTGAAAGATCTACTGGAGAGCCCTATAAAATGCGGAGCCAACGATATCGTTTACAACCGTCATTATTTCAAAGTA

>CcSNMP1 [ORF 4092 bp]

ATGAACGGAAGACACAGGCGAACCTATAGTCTTAGTACTACGGGTAATGAACTGGTAAATCAAGTAAGTCCAATAGCTAAAGATCCATATCGCACGTTTGCTTTAAATGGAGCACGCGACATTGCAAGGACTTCCACACCTTTGCCCAAGAGCTCTACTTGGAAGAAGGATATTCAACCAAACGGAGACTTGGGATTATTTACACATTCTAGCACTCCGTTAATCAAGAAACAGTCACCCACACGAAGTGAGAACACGCATACATTGGTGTCACAACAACGCAGTGAAGAGGCACTGTTTGGTCCAAAAGGTTCGCCATGGGGACCTGGCAATAGTCCCAAGATAAGGGAAAGACCCGTAGGTGTTAAAACTGTGCAAAATGTAGCTGGTCCTCTATTAGCATCTACCAGATACAATATTGATCCTAAGTTATATACAGATGTAAGTTCACCAGGTCTGACAACACGTCTTACCAAATATGCAGCTGAAGCTAGTAATAAATTAACTCATCAAACTCAATATGGAGCAGGACAGTTTCCAAAAGTTAATCTCAATGCAACTCCAGCTCCCTTGTTAAGTCCTAAAGTGGCAAGGGCAAGGATTCCAGTAACCGTTAGAGTTGCACCTCCAGAAATAATCAACTATTCACCCGTGGATACACACCATGATAGATCCTCCAGCTCTTCTTCTCAGGAACTCAAAGAATCCCTGTCTTTTCCTAGTGTCAATAAGTCAATGAAGAAGCTTCCGCTCAAGCGACACACTTCTAGAGAGGATATCATGTTGGATTTAGCAAAAAAACAACGATTTGAAAGGATATACGATGGTGAATTAGAAAATTTGGAGGAAATGATGCAGAAACGAACTAGAGAAGAGTCAACAGCGTCGGATGAAGATGAATCTCCTCAATCGATACACGAAAGACCGTCAAAGAGGCCGAAAGCATCATCTTGCCACGATGTCATTAATTCACTCAGTTCCAGTATGAGCGTGTATACTGGCATTAAAAGGAAAGCAATGGATACATCTCGTTGTAATACTCCAAATATTGAGAAACATTTCAAACCATCGTCTACCTCTCCCCCAAGATTTTCATCTTTAAGTCTGTCATCTTCTGGAGAATTGGCGAGATCGAAAGAACAGCTAAACGTAAAAGTTCCCGTAATTACTACGATCAAAGGAGTATCACCTGAAAAAGCCAAGGAGACGAGTCCAGAAGTGACAAAACCCGACGGTCTTTCCCCAGTAAAATCGAATTCGCAATCACCTCATAATAATTTAAATTTACCAACTCCGAAACTTCGTAAAGAGATCCCCATCGCAGCAGATATTAAACCACCAGCTAAACTCACAGAGAAGCTATTTATGAGAGCAGAGCCTCAAGATAATGAAAAAATAAAATCGTTGATAGAGGAGCAAGGTAAAATAGAGCCAAAATTTACGACAGAAGACAAGGAAGAGATTAAAAAAAAAGACATCGTTAACATGAGGCAAAATAGCATGCGTTTGAGGCTTCAGAATATGTTTGATGCTATTTCTGGAAAATCAAGCAAAATCGATCCAGACGTCGTGATTCGAGCGGAGGAGGTGAATCCTGCCGCTACTTCATCCTACGCTTTGCTCAGTTCTCAAACTTCAACGATAACCGTTAATACCTCCCCAATCGCAACAACGATAGTGCCAATCTTGAAAACCGACACAGAGACTAAGTCGCCCACGAACAAGCATGTTACCTTCAATCTCCCATCCTCGCAGTCGTCGCTTAGCAGCAACACTAGCACAGTTTCCAGTGCTGAAGTAACCAAGCCAACCGAAACGAAAATTGTGACTTCGGTTCCAAACTTCAATTTCAGTTCGAGTCCAAGATCTGCGCAAACTTTCAACGTTGGTAGCATTGCTTCAACAACTGCATCGACCACAACCACCGTATCGCCGTCGACAACGACTAGCAATTTCTCCTTCCCAGCTAGCAGTAGCGCAACGTCTATCGCCACGTCGAAGATCGAAAGCAACCTACCAACTACTACCTTGTCTTTGAGCAGTCCTACTTCGCTCTTTACTGCTAAGCCATCGTCTACGAGTATCGGCACGACTTTGAGTTCTACAGGATTCTCATTTGGTTCGGTCACGACTAAGGCAGCTACGTCGATGTCAACTGCCGCGTCATCCGCAGCGCCAGTAACGTCATTCGCCATTACGAGTCCCGTTGCCAAAACCACGGAGAATAATTTAGGCGGTATTACCTCGCCATCGACTTTCGGAAATGCTTTGGCAAAGAGTCCGATAAGTATATCTCTCGCACAAACGACAACCTCGTTACCGAGCTTCGTAGTTGATAAAAAAGAGCCAATGTTCTCCTTTGGCGGAGTATCATCGACCCCCAGCATTAACTCGGCAACTACAACCAACCAATCGTCCTCGACGTTCAGCCCAACAACCACGACAACTTCGATGTTCTCAGCTGGAAGTAACAATACTCCATCACCCGCGTACAACGCTACCTCGACCGTCACCACCTCGACACCCGCTACTGCACCTATATTTAATTTTGGAGGCTCCAGTAAACCACCTGGTACATTCTCGTTTGGCGCAGCGACGTCTCCGGCTGTCACTACCGCTGCAACTACAGTAACGCCTACGTTTGGAAGTGGTACTGTTACGGCGATTGGCTTTGGCACCTCTGCTGCCAGTAATCCCGCTGCTACGACCTCAGCACCGGCGTTCGGTTCGTCAACGGCGTCGCTTTTTGGTGCGCAAACAACTAGTGCACCCAATCTCTTCAGTGGCACGGGGTCTACGACCACCGTGAGTCCGATGTTCAAGCCGCCGAGCTCCACGGCTACCTTTGGAGTTCCAGAGACGACTACCACAACGTCGGCCACTTCCTCGATATTTGGAGCGCCATCGAGCACTTCGAGCTTCGGCGCCACGCCATCAACGACGTCGACGCCCAGTTTGTTCGGTAGTGCAGTGGCATTGGCGCCTAGCACGAGCTTGTTCGGCAACCCTTCGGTAACGGCAACAATGACGCCGTCGGCATTCAAAACTACCCAGCATGTCAGCATTTTTTCCGGCGCGTCTATCAGTGCGACTGGCAGTGCGACTGGCGCAACGTCGATCTTCGGAGCGTCAATTGCAACTGCGCATAGTGGATTGTTCAGCGGATCCAACGCCAGCGCCAATCAGACGTCAGCGGCAAATCCAGCTGACACCGGCGGCGTCTTTGGCCAATCGAAATCACCAACGTCCATAACCTTCGGGAGATCGACGTCAGCCTTTGGCTCGGCCACTGCACCGTCGAGCTTCCCAGCACTCAACGCGGCGGGTCCATCGACTTTACAGGTCGACGCGGCGTCGTCGCCAACTGGCAGCTTACCAGCCTTCGGATCGATCAAATCCACGACGCAGTTCGGCGCGTCAACCACCACTCAACCGTCGACCACTAGTTCTGGAACGTCGATATTCGGGCCAACCGCTACTGGCGGGACCTTCGGAGCGAACCCTACACCGGGTTTCGGAGCCAACGTCAACGCTACCCCAACGTTTGGATCAACGACTTCGACGAACAACGCTTTCGGTATGTCGCCGACAAGTTCCAATGTTCCCACCTTTGGAGCTTCCAGCCCTTCCGGCTTCGGTTCCACTGCCAATACGCCGTCGCCTTTCGGTGCACCACCGACGTCGACTACAACGAGTCCCTCGCTGTTTGGCAACGCGGCCGTCGCGGGTACATCTACAGCTGGACCTGGCGCAAACAGTACTTTCAGTTTTGGAGGCAGTCAGCCAGCTCAATCGGCCGCTCCGCAACCTACCAGCGGATCGTCGTTCTCTTTTGGATCGGCGGCTGGTAGTACTGGCGGACCGGGCATTTTTCAGTTTGGTGCCGCCAACAACGCTAATAAACCAGCCGGTTTCAATTTCAACGCACCGACAGCAGCGCCGCAAATCAATTTCACCGGCAACGCAGCAGCAACACCGACGTTCAACGCTCCAGCACCAAACTTCCAACAACCCGGGACGAACATGTTTAGCATAGGTTCAGGGTCGTCGACGCCTCGCTCGCGAGGTGGCCGCGCCAGGCGGCAAAGATGA

>CcGR1 [partial 933 bp]

AATTTTTATGTTTTTTTTTTCTCTCTTTTTAAACCTTCATGACTTCCGTTGATTTATGACTTAACGGAATATGAGTTATGCTCTTATTTTTATTGCTTGATTTATATTTGACGGGACTTGAGTTCTAATTTTATTGTTTTGACGCAGTTAATATATTTGTTTCGATGCATTCTCATGTTACTAGTTTAGTTGGATTTTGCAATATTAGGTGTTACTAACATTGAAGGACATCATATCATACAATTGTTAAATGTGTTTTTTTTTAGAATAACACCACTTTACTTCATTTATATTTTTACCAGTTATATTTTAACTCAATATAATGAAGTTTTCGAGTTTTCACATCTTAAGCTAATTGCCCTAGTTCCAGTTGTTGTTCTATAAATATTTTATCTTGTTTACGCAGGTGTTAATTGTTCCCTATTTTGCCTCCTCCATTTTCTCAAGTTTTTATATTTCTGTTTCCTCATTTTTTTGTTTATTTTTTACGTACAATTTTTCATATTGTTTTCATCGATATTATATTTACTTATATAAAAAATAATAATCAAATGATATTTAATTTTAAATCAACTAATCCTTCCCTATATATTGGAGTATTTTTATATTATTTTTGACAAAATCACCACTAACTTTGTTGTATCGCATATACAATTATTTTCTAGCATTTAAGTTTGTAATCTATTTTTGTAAGTAATATCAGTTCTTATCTTTATCGATATTGAATGTAACATAAAGCTTTAATCGATACAATCGCTTTTGATACTTTTAAATACTCATTTTCTTTTACGATTACCATTACTACTTGTTTCATGTTTCATCCAGTAGATATTCCTTATCCATAATCATTTTTACACTTACTAAATTTTATTTTTTCTTTATATGTTTATCAAATTTCTTTTATTTTACTTGGATCGTGTAGTCTTTACTTTC

>CcGR2 [partial 522 bp]

ATTAAATTGGTCATTTTTATTTATATTTTCCTTTGGTTAGTAGTGACAGCAACTTTACAGTATTTTGATGTTTATAATATTATAATTTATACTGTGTTAAATTTGAACGAATGGCTTCGAATGACGGTAGTTATACAGTATTTTATCATTGTCGAGTTAGTAAAACTGATGTTTGTCAGGCTTAATGAAAATTTGCTTGGTCTATTGAGGCCGATCGATCCACTTTGCGAAGTCGCTCAGGGTTTGGAATGGGAAAAACAATTTTCACAATTGTGGAAATTCCATGCCGATTTAAGCAAATTATCTCGCGACATATCGAGCTTTTATTCTCTGAGTATGTTGTTGATTATCGTGGGAAAATTTATCAACTCGATCTTAATCACCTATAATATGGTTAAACCAATATTTCTTGGAGGCCAA TATTTGACTGAATATCAATACGTCAATAGATCTATGTGCATGATCGTATATCTAATTCCTATCGTATTGTTAACAACGTCAGTCTCCGATACACTCAACGAG

>CcGR3 [partial 414 bp]

GCACTACTTGTCTTTGTTGTTGTCGTTGCTCATCCTGCCAATGCACGACCTTACCAATATATACCGGAGATACCTGGTTGGATACCTGTGTATATAAGACAAGGTGATCAACCTTTATCGGAAATTCATCCGGCGTTAGCAGAAGCTTTCCACGAGGATATTCAATCAATTCGTTTGGACGAGTCAGCAAGTAAAAATCTCTTAGCTGACGGGAACGAAGTTTCGGCAGAATTGAAACCAGTGAAGGAGAACGCTCAATCAATTGGTGCCAATCCCGAAGAGAATGACTTCCCGCAAAAAACTAAAGTATTCGAAGAGGATTCCGTTGTGATGAAGATACCCAAGCCATTGGCGTTCGATAAAGCCGAACTAAAGAGACTTACCAAGGAACTCAAGCATTATAAGAAGAATGAA

>CcGR4 [partial 192 bp]

AAAATAGTGCAAGACTGTTTGGAAATCCCTTTGAACGACAAACGCATGGAAAAACAGCTTATTAATTTTTCTCAATGCTTATTACATCAAAATATCCAATTTACCGTCTTTGGTTTATATTCGTTAGATATAACTTTGTCGAAAGTGCTGTTGTCTTGTACCGCAACGTACTCAATTATACTTTTGCAATTT

>CcGR5 [partial 162 bp]

AAACAGCTTATTAATTTTTCTCAATGCTTATTACATCAAAATATCCAATTTACCGTCTTTGGTTTATATTCGTTAGATATAACTTTGTCGAAAGTGCTGTTGTCTTGTACCGCAACGTACTCAATTATACTTTTGCAATTTTTCAATACGACAACTCGAAAA

>CcGR6 [partial 1491 bp]

ATGTACGAAGCAAGAAAAAAGGTTTTTTGGTTTGCTATAGTCTCTCGATTATCAACTTTGATACTGCAAGTGATTTTCAATGTACTATGTCCAGATCACGATGCAGACGCCTTTCGTACACCTGCAGATCCAACTGAAAAACATTCACGTTTAGACAATATCGTAACATTTTTATTAGAAGGACTGACGAGATGGGATGCACAATATTTTATTCATATAGCGAAATATGGTTACACATATGAAAATACTCTTGCCTTTTTTCCACTTTTTCCATTATCGATGAAATATATGGCCAGAGTGTTTAGAATACAACCACCAATACTTAATTATAGTAACGTCATTGTGATATGCGGCGTTGTAATTAATTTTGTATGTTTCGTCAAAGCAGTCTTAGTTTTTTACGATCTCAGCCTAGTAGTATTTAAAAACATTAAAGTAGCTTATCGAGCTGCCATATTTTTTTGTGTAAATCCAGCAAGTATATTCTTCACTGCTCTTTACACGGAATCGTTGTTTGCTTACCTGTCGTTTTATAGTATGCTAGAAAGCATCAGCAATAATCCTTGCGTATTTTTGCCATTGAGTCTTTCTAGTTTAGTAAGATCGAATGGACTAGTCAATCTTGGTTTTCCTATTTATTTTTGGTTGAGAAATTTATTGATAACAGTTTTGCCAAACTATGTGTTGGAAAATAGGCATTTTCACGGCAATTCAAAATCATTGCTCTTCAACTTTCGACACGTTTTCATAAGTTTATCCCAAATTATTTTTGTAATAGTTTTGTCCCTGCTACCATTTGGTTATTCACAAGCTTACAATTATACGAAATTTTGTAAACCGGAATTAAACGATTCGTTGCTTCCGTATCACGTACAAGAATATGCAATAGGCAATAATATGTCGTTGCCAGGAGAACATGATTTTTCATGGTGCAATTCCAAATTACCCATTGCATATTCGCACATACAACATAAGTATTGGAACGTAGGTTTTCTAAAGTACTACCAATTCAAGCAGATTCCTAACTTCATTCTTGCTGTACCTGTAATATACTTGATGTTAAAGTGCTGCATCGAATTTTTTAACGAACACAAATCAAAATTTTTCACTTTGGAATTTTTCACGGGTAAATCCAGAGCGTCAGACAATATAAAACAATACCCATTAGAAATGTTCGTTTTTGTAGTACACGCTTTATTTTTGACGATCTTTTGTATTTTCTTTGTACATATTCAAGTGAGTACACGGCTTCTATGCTCAGCTAGTCCCGTGTTATATTGGTATTGTGCCTTAGCAACTTTGCGAAAAACTAAAACGTCAAAAAAACTTAAAGAGATTGAATATGAAAGTTCGGAAAATTTATACTCTCGATGGAAAGTATTTTTTATTACTCAACAGCACTATCCTTATCAAGAAAAATTAATCCTTGGATATTTTCTAGGATACTTTGCGATAGGCTGTTTCATGTATGTTAATTTTTTGCCTTGGACT

>CcGR7 [partial 783 bp]

ACGGCTGGGATAACGAGTCTACTATTTTTGAAACTTGCGCGACAGTGGCCGACTTTTGCTGTCTCTTGGGAAAATATGGAACGTGAACTCGCTGCTCGTCACAATCCACAGAAGCAGAATTCTCTCAACCTCGCGCTCAAGTTCAAGATACTCAGTATCGTCGTCATGGTGTTTGCACTCGTGGAGCATACATTATCAATTCTCGCCGGATACTTCAGTGCACTGGAGTGCGCAAATATTCGTGGCGACGAAAACATCTGGGCCACTTATTTCATGTTACAATTCCCAGGGATGTTTACCCATCATAACTATGCATTCTGGAAAGGATTTATCGTTCAATTTATCAACTTCCTAAGTACATTCTCTTGGAATTTCATGGACCTTTTTCTCATTCTTGTTAGCGTCGCTCTCGCCGAACAATTCAGACAGCTCAACCACAGACTCTACTCAATTAGGGGCAAGACAATGCCTGACTGGTGGTGGGCCGAGGCACGGATCGACTTCAATCGATTAGCCACCATGACACGACGCGTCGACTCTCAAATCTCCGACATAGTACTTCTTTCTTTCTCCACAAACCTCTACTTCATCTGCATTCAGCTGCTCAACTCGTTCAAACCGATGCCGAATGCAATTCAAACAATTTACTTCTGCTTCTCCTTTGGTTTTCTACTGTCAAGAACAGCGGCGGTCTCGCTGTACGCCGCCACCGTGCACGATGAATCACTTTTACCCGCTCCAATATTGTACAGCGTTTGCACCGAGAGTTACTCCAAAGAAATT

>CcGR8 [partial 570 bp]

AACAGGTCCAGGCCATTAACGTTGAAAGTATTTGAAGTGTATTTATCTCTTGTGCAGACGAGTAACGTGATGCTCTTTGCTTGGATGATACTAAATGTTGGTTGTAGATTTCAAGTTATGAATCAAGGAATTCAAAGTAGAATGTCTAAAACCAACATCAATAATTATACGGTATCGGCTAATTATATGTTTCTAAGAACCTCGGCACAAGCCCATTCAGAACTCTGTAAAATAGCGAAAAGGGCAATCGAACCATTTGTTATAAGCATAATCAATTGCGTCATCATGGCATTCACCATCACTACTTCCATCGTATACGTTATTTTTAGCGAATTGAAAAACACGTTAAGCGTCAACCATGCTTTGTATTATTTCACATTAATAATGACGTCTATTTTATTAACGATTCTAATTGTGGGTAGTTGTAATTGGACCACGAGGAAGGCTGCTGAAACTATGAAAATACTCCATAAAATCATGTTGGCAAATATTAGTACAGATAATAAACATCTTGATGAAACGGCTCGAACGTTTTGCATGCAAATAATTCATCATAATCTACATTTCACC

>CcGR9 [partial 819 bp]

GTATTCGCTTGTGACCTTACGATGAATTGCATCGTTTTTGACGACGCTCACGAAGCTATACTTGTAACGATCATTTTCGACATCCCGTTACTGACGAATCCTATGGTTGAATTGAATTTCGGTATAGCAATCTCAATCTTGGGAAAGAGATTTGAAAGGCTGAATGCACTCCTTCAAAGTATCACGGATACGCCAATGTCGTCAGCTCATCCAAACGACGCAAATAAGTACGAAAATATCCTAAAGTGCAATCAAAAGAAAGTTGCCGTTCGGCCAACTTTCTATCATCAAAATAGAAATAACTTGGAACTACTACTAAAAGCAACAAGACAACTCCATTTGGATTTGTGTGGAATCTCTCGAAGAGTCAACGATACCTGTAGTAAACAAATGTCGATGCAAATGGCAGCTACATTTTTACTTCTAACGGGATTCAGTTATAGCTTTTATCTTGTTTACAACGAACCTTACATACCACTTGAACATAAAGTCCAACATTACGTTTCTTTAGCGGTATGGATTATAATCAGTATTTTCCGAATGATTTACGTGGTTCGCATATCTGTCAACGTTACCACAGAAGCGCAGAAAACCAGTCAAATAGCACACGAAATACAAGTTCCTAGATCAAAGAGCAAATTAATCGACGAAATCCACCAACTGTCTTTGCAAATAATGCAACATCCGTTGTACTTCACTGCTTCTGGACTAATCGTGCTGGACTTTGGCTACGTTCGAGGGTTCGTTGGATCAGTTACAACGTACTTAATGATTTTGATACAAAATCAACCCGACATGATTAAAGCTGCCAATACTCTA

>CcGR10 [partial 153 bp]

ACGTGTTTTCAGCTGAACCAATTTTCCATTTACTTGCTGCAAGACAACGTCAGCTTTACGGTATTCGATCTTTTCTCGCTGGATAGCTCTCTACTCGTCAAGATTGCAGGTTCCATCACTACGTACATGGTTATACTATTACAATTTAAAAGT

>CcGR11 [partial 618 bp]

AAGTATCAGATTCTAAATAGAACGTTGACAAATATGCTGGGGGAAGCACCTCGTCACAAATTTTTGATGAACCATCTGATAAACGAGCAAACTAAATTACGTCTCCGTTCGATTAGTAATACTGTCAAAGAGGCAACGACCTTAATCAAATTAACGAAGGAGGTTCATCTGCAATTGCTCAAATTGTGCGAGCAAGTGAACAATACTTACGGACTGCAGATACTATTGTCGATCGTTGTTGCATTTGGCGTTATAACGGGAAATATTTACGAATCGTACTCACTGCTGCAGGATGGTTACGTCAAAAGACATTTGGCTTTTAAATCGATGACCAATTCGATAGTCTGGACCCTTTACTACGCCATCAAGATTTCGAATTTCATCGAAGCTTGCAGCCGATGCAAACAACACGCCGTGGAAACGGGAGACCTGTTAAACAAGTTTTACGACGATCCATTTGCAGACGAGCAGGTTCAAAGTGAAATAAGAGACTTCAACGTGCAACTGATACAGAGACCGGTACAATTTACGGCAGCCGGGTGCATTGCCCTTGATACGCGTCTACTGCAAATCATGGTGTCTTCGATCACAACTTACTTGATGATTTTAATTCAACTT

>CcGR12 [partial 480 bp]

ATAGTAGCAGTTGGTACGTACTTATACTCCAAGTGTTTTGGATATTTTTTCACGTCTACAGGTTGTTACTTTTCGTTCAGCCGTGTTATATGGTTTCCGTTAAGGTATACATTGTAATATCGTTTATAACGTACCACTGTAACTATACATTTATAATTTATCGTTGAAAACTAAAAGGCACGAACCACAGGAGCTTTGGTAAGCCAAGCACTAGCAACCAATTGGAATCCCGAAACACAAAAGCAATTGGAAATATTTTCAATGCAATTGTTACAGAGACCTATTGAATTCACCGCTTGTGGACTTTTTTATCTAGATCGAGGTTTAATGACATCGATAGCTGGTTCGGTCACCACGTACTTGGTGATACTTGTACAATTTCAAAATGCCGACGATACCAAAGGTACCAAACATTTGCTCCAAAATGCATCGGAATTGTTGAGGAATGTTTCCAGTTTCAAAAACATAAGCACAATCAAG

>CcGR13 [partial 381 bp]

TCATGGGTGTTGAAATTATCACAGGATATATCCGATTTCTATTCTCACTTGATATTACTTTGTATTCTTTATGTGTTTGTAACATCTGTACTCTTTGGTTACTACATCTTGACACCAATTGTCAAAGGAAGGTATCCAGTATCGCTAAATGATTCTGTCCATTGTGTTTTCTATTTATTACTCTTAGCAGTTTCACTAAAAATGCTGACAAATATAGCCACGGCGACTGTCAAAGAGCACAGACAATTGAGAGATATTTTGAATGAGAGTTTCGACAAAATTAACGATGATAGCGTTATTAAAAAAGTGACAAATTTTACAATTTATTTGTCGCAAAAGGATATCGTATTTAGTGTGTACAATTTATTTTCTCTAGACGAC

>CcGR14 [partial 369 bp]

GTGACTTTCAACATTTTGTTCTTCATTACGGGTACAAAATATTTCTATGAATACTTGAATATGGTTAAGAGACGTTACGCATTTTCAATGGACGATGATTCTTTATCGATTTTAATTTTAACAATAAGACAGTTTTTTAATATCGTTTGTACATTCGACGCTATGATTTTGAAAATCATCTATAGCAAACAAGTGACTTTATCAATGGAAAACCTTGCGATCCAAGATGAGACGCTGGCAAGTTTTGGATATCAATTCAAATACAAACCGGCCGCAAACCTGAGCGTTGTAGTAGTAACTACAATTTTAATTATTTCTTATAGTACCGTGAATTTAGAGTTTCTTGTACTCGTCCAAGTGCCTGTTCCA

>CcGR15 [partial 156 bp]

CAGTTACTTCAGTTTTCGATTTACCTGTTACACAACAACGTATCTTTCAGTATTTTCGAT

CTATTTTCTTTGGATGGCACTCTCCTCGTAATGATTGCAGGTTCAGTGGCAACGTACTTG

GTGATACTATTGCAGTTTCAGCCTCAGAGTACTACC

>CcGR16 [partial 279 bp]

AATGCCTTTATATTGCAATATTGCATTGTGTTAAAATTGCTGTGTCGCAAGTTTAACATTATCAATGAGAATTTGCTGCGAATGTCGCGAATAATCGATGCCAATGACTTGCTGCTGCACAACAAAATATTAGAACAATGGGATTTGTATTCGAAGCTAAGTGACATGTGCCAGGCTATATCAAAGTTCTATTCTTTCTGTACATTAATGACCGTTGCGTATCGATTTTTAACATTGACGACAACCGCTTATTTTTTAGTCAAACCTATCTTTGTCAAA

>CcGR17 [partial 798 bp]

TATATTTTTTTACTAGCTCAATTTCCTCATATATATTCGAATAATTTGGTATTATTTATATCTAAAATGTACGCGATGTACATAACGATAGTTGTGTTTCTAGTCGACATGCAATATATGACTTACGTTATGATATTGAAAATTTGTTTCAAAAATATCAATAACTATTTACTGAAACTGAAAATGATCAACGACCAGAAATCAGTTCATACTGATGAAAACGTAAGTTGTTTGGGAAATAGCGTCAAACTACAGTTCGTAAAATTGCGAAAATTACAACTTAGACATCATAACGTAAGCAACGCAGTCAAACAGTTAAACAAAGTGTTTGCTTTACATGTCATAGCTACAGTTCTTATGACATTTGTGGAGGTGACATTTGGTCTATATTTTTTTATATTGCACAGTCAAGGTAGAAAAGGTATTGATTTGGAAAAGCAAATATGGTACAATTATTTTATAACGTCTGTAACTTACTACTCGCTCAAAATTGTGATAATAGTATGGGTATGCCAAAAGGCTACAAATGAATCTGCGAAAACCGGAATCATTGTGCATGATGTTATTCTAAACAACGATAACGATCAATTTATTGCAGAGGTAAAATATATTTCTATTCACTGATATTTAAATCATTGTTCACAAGTAATGAATTCGTCATTCTTTTTATCGTAGCTTAATCTATTTTCGCTACAACTACTACAATGCAAAAATGAATTTACCTCCAAATGTATCACTATGGATGCAAAACTTCTTACCGCTGTATGTTACGAAATCTTATTGATATTCTTTCATTTT
