# Supplementary material for: Transcriptome and Expression Patterns of Chemosensory Genes in Antennae of the Parasitoid Wasp Chouioia cunea
Source: PLoS One. 2016 Feb 3;11(2):e0148159. doi: 10.1371/journal.pone.0148159 (PMC4739689; doi:10.1371/journal.pone.0148159)
Supplement: S4 Table — (DOCX) [file pone.0148159.s009.docx]

S4 Table. List of OR genes in *C.cunea* antennae

| Gene | Unigene | Length of Unigene  (bp) | ORF  (bp) | BLASTx annotation | Score | E-value | % Identify | RPKM value | |
| --- | --- | --- | --- | --- | --- | --- | --- | --- | --- |
|  |  |  |  |  |  |  |  | Male | Female |
| Orco | Unigene11320_All | 1636 | 1428 | gi\|283436213\|ref\|NP_001164465.1\| odorant receptor 1 [Nasonia vitripennis] | 834.7 | 0 | 93 | 9.2183 | 30.2326 |
| OR1 | CL546.Contig1_All | 1887 | 1260 | gi\|299522910\|ref\|NP_001177592.1\| odorant receptor 236 [Nasonia vitripennis] | 633.3 | 1.00E-179 | 86 | 2.0843 | 10.6553 |
| OR2 | Unigene13145_All | 1387 | 1227 | gi\|299523221\|ref\|NP_001177470.1\| odorant receptor 14 [Nasonia vitripennis] | 506.9 | 1.00E-141 | 79 | 1.9361 | 8.612 |
| OR3 | Unigene10017_All | 1751 | 1236 | gi\|299522736\|ref\|NP_001177510.1\| odorant receptor 78 [Nasonia vitripennis] | 545.4 | 3.00E-153 | 67 | 1.1928 | 7.2218 |
| OR4 | Unigene23316_All | 992 | ---- | gi\|299523264\|ref\|NP_001177486.1\| odorant receptor 35 [Nasonia vitripennis] | 311.6 | 3.00E-83 | 67 | 0.3281 | 5.8263 |
| OR5 | Unigene19906_All | 248 | ---- | gi\|299522836\|ref\|NP_001177556.1\| odorant receptor 170 [Nasonia vitripennis] | 57.4 | 2.00E-07 | 58 | 0.5469 | 5.6498 |
| OR6 | CL3151.Contig1_All | 888 | ---- | gi\|299522738\|ref\|NP_001177511.1\| odorant receptor 79 [Nasonia vitripennis] | 349.7 | 1.00E-94 | 79 | 1.6495 | 5.4042 |
| OR7 | CL971.Contig2_All | 755 | ---- | gi\|283135146\|ref\|NP_001164396.1\| odorant receptor 80 [Nasonia vitripennis] | 290 | 7.00E-77 | 75 | 1.473 | 5.3355 |
| OR8 | Unigene17447_All | 1034 | ---- | gi\|340725033\|ref\|XP_003400879.1\| odorant receptor 13a-like [Bombus terrestris] | 151 | 9.00E-35 | 51 | 0.0525 | 5.3187 |
| OR9 | Unigene7290_All | 1162 | ---- | gi\|299522963\|ref\|NP_001177618.1\| odorant receptor 285 [Nasonia vitripennis] | 341.7 | 4.00E-92 | 71 | 0.5369 | 4.9137 |
| OR10 | Unigene10603_All | 1336 | 1266 | gi\|299523100\|ref\|NP_001177429.1\| odorant receptor 2 [Nasonia vitripennis] | 657.9 | 0 | 88 | 1.9491 | 4.6408 |
| OR11 | Unigene14300_All | 1143 | ---- | gi\|299522902\|ref\|NP_001177588.1\| odorant receptor 229 [Nasonia vitripennis] | 319.7 | 1.00E-85 | 64 | 1.5188 | 4.4437 |
| OR12 | Unigene3722_All | 741 | ---- | gi\|299782524\|ref\|NP_001177710.1\| odorant receptor 289 [Nasonia vitripennis] | 384.8 | 2.00E-105 | 90 | 0.4393 | 4.2545 |
| OR13 | Unigene17625_All | 559 | ---- | gi\|299522720\|ref\|NP_001177502.1\| odorant receptor 61 [Nasonia vitripennis] | 235.3 | 1.00E-60 | 73 | 0.2911 | 3.7598 |
| OR14 | Unigene10391_All | 991 | ---- | gi\|299522971\|ref\|NP_001177622.1\| odorant receptor 294 [Nasonia vitripennis] | 156.8 | 2.00E-36 | 56 | 0.5748 | 3.7468 |
| OR15 | Unigene10273_All | 1132 | ---- | gi\|299523266\|ref\|NP_001177487.1\| odorant receptor 36 [Nasonia vitripennis] | 379.4 | 1.00E-103 | 71 | 0.7668 | 3.6514 |
| OR16 | Unigene9066_All | 1009 | ---- | gi\|299782522\|ref\|NP_001177711.1\| odorant receptor 296 [Nasonia vitripennis] | 357.1 | 8.00E-97 | 74 | 1.5323 | 3.5063 |
| OR17 | Unigene5579_All | 464 | ---- | gi\|299523229\|ref\|NP_001177472.1\| odorant receptor 16 [Nasonia vitripennis] | 63.2 | 4.00E-09 | 62 | 1.0522 | 3.4727 |
| OR18 | Unigene27324_All | 318 | ---- | gi\|283135140\|ref\|NP_001164395.1\| odorant receptor 82 [Nasonia vitripennis] | 168.3 | 9.00E-41 | 89 | 0.3412 | 3.4147 |
| OR19 | Unigene10394_All | 1323 | ---- | gi\|299523212\|ref\|NP_001177467.1\| odorant receptor 10 [Nasonia vitripennis] | 235 | 7.00E-60 | 64 | 0.6151 | 3.3361 |
| OR20 | CL3740.Contig2_All | 316 | ---- | gi\|299528645\|ref\|NP_001177643.1\| odorant receptor 288 [Nasonia vitripennis] | 59.7 | 4.00E-08 | 56 | 0.2575 | 3.3255 |
| OR21 | CL4622.Contig2_All | 243 | ---- | gi\|299522928\|ref\|NP_001177601.1\| odorant receptor 256 [Nasonia vitripennis] | 63.9 | 2.00E-09 | 62 | 0.1116 | 3.3155 |
| OR22 | Unigene24785_All | 642 | ---- | gi\|299522904\|ref\|NP_001177589.1\| odorant receptor 230 [Nasonia vitripennis] | 114 | 5.00E-24 | 58 | 0.6337 | 3.2737 |
| OR23 | Unigene28282_All | 678 | ---- | gi\|299522934\|ref\|NP_001177604.1\| odorant receptor 261 [Nasonia vitripennis] | 121.7 | 2.00E-26 | 58 | 0 | 3.2549 |
| OR24 | Unigene28115_All | 238 | ---- | gi\|299522807\|ref\|NP_001177541.1\| odorant receptor 137 [Nasonia vitripennis] | 70.1 | 3.00E-11 | 59 | 0.3419 | 3.2379 |
| OR25 | Unigene20547_All | 314 | ---- | gi\|299782513\|ref\|NP_001177706.1\| odorant receptor 198 [Nasonia vitripennis] | 142.1 | 7.00E-33 | 79 | 0.0864 | 3.2351 |
| OR26 | Unigene28417_All | 223 | ---- | gi\|299522815\|ref\|NP_001177545.1\| odorant receptor 143 [Nasonia vitripennis] | 58.5 | 1.00E-07 | 71 | 0.2433 | 3.1416 |
| OR27 | Unigene4509_All | 1159 | ---- | gi\|283436097\|ref\|NP_001164418.1\| odorant receptor 246 [Nasonia vitripennis] | 313.5 | 1.00E-83 | 62 | 1.1468 | 2.9921 |
| OR28 | Unigene3459_All | 450 | ---- | gi\|299522831\|ref\|NP_001177554.1\| odorant receptor 166 [Nasonia vitripennis] | 57 | 3.00E-07 | 45 | 0.8439 | 2.958 |
| OR29 | Unigene20868_All | 513 | ---- | gi\|299522874\|ref\|NP_001177573.1\| odorant receptor 201 [Nasonia vitripennis] | 102.1 | 1.00E-20 | 51 | 0.1057 | 2.9361 |
| OR30 | Unigene10731_All | 682 | ---- | gi\|299523107\|ref\|NP_001177431.1\| odorant receptor 5 [Nasonia vitripennis] | 291.6 | 2.00E-77 | 80 | 1.7897 | 2.9276 |
| OR31 | Unigene10134_All | 1237 | 1167 | gi\|299782517\|ref\|NP_001177708.1\| odorant receptor 241 [Nasonia vitripennis] | 358.2 | 5.00E-97 | 66 | 2.105 | 2.8601 |
| OR32 | Unigene19955_All | 371 | ---- | gi\|299522882\|ref\|NP_001177577.1\| odorant receptor 207 [Nasonia vitripennis] | 59.3 | 6.00E-08 | 55 | 0.7311 | 2.8325 |
| OR33 | Unigene21167_All | 433 | ---- | gi\|299523119\|ref\|NP_001177435.1\| odorant receptor 9 [Nasonia vitripennis] | 54.3 | 2.00E-06 | 82 | 0.5638 | 2.8314 |
| OR34 | CL3725.Contig1_All | 835 | ---- | gi\|299522813\|ref\|NP_001177544.1\| odorant receptor 142 [Nasonia vitripennis] | 33.9 | 1.00E-07 | 66 | 3.9631 | 2.8107 |
| OR35 | Unigene19908_All | 455 | ---- | gi\|299522932\|ref\|NP_001177603.1\| odorant receptor 260 [Nasonia vitripennis] | 156.4 | 3.00E-37 | 75 | 0.1192 | 2.7715 |
| OR36 | Unigene3936_All | 382 | ---- | gi\|299522844\|ref\|NP_001177558.1\| odorant receptor 175 [Nasonia vitripennis] | 169.1 | 5.00E-41 | 81 | 0.6391 | 2.7509 |
| OR37 | Unigene2177_All | 560 | ---- | gi\|299522732\|ref\|NP_001177508.1\| odorant receptor 68 [Nasonia vitripennis] | 123.6 | 4.00E-27 | 79 | 0.7265 | 2.6272 |
| OR38 | Unigene29337_All | 318 | ---- | gi\|299522775\|ref\|NP_001177528.1\| odorant receptor 114 [Nasonia vitripennis] | 127.9 | 1.00E-28 | 75 | 0.4265 | 2.5335 |
| OR39 | CL4313.Contig1_All | 498 | ---- | gi\|299523244\|ref\|NP_001177477.1\| odorant receptor 22 [Nasonia vitripennis] | 116.7 | 4.00E-25 | 66 | 0.1089 | 2.5322 |
| OR40 | Unigene19418_All | 394 | ---- | gi\|283436099\|ref\|NP_001164419.1\| odorant receptor 249 [Nasonia vitripennis] | 173.3 | 3.00E-42 | 81 | 0 | 2.4893 |
| OR41 | Unigene28353_All | 388 | ---- | gi\|299523104\|ref\|NP_001177430.1\| odorant receptor 3 [Nasonia vitripennis] | 202.2 | 6.00E-51 | 88 | 0.0699 | 2.4376 |
| OR42 | Unigene13499_All | 289 | ---- | gi\|299523217\|ref\|NP_001177469.1\| odorant receptor 13 [Nasonia vitripennis] | 105.1 | 1.00E-21 | 80 | 0.7508 | 2.4241 |
| OR43 | Unigene22398_All | 221 | ---- | gi\|299522809\|ref\|NP_001177542.1\| odorant receptor 139 [Nasonia vitripennis] | 63.5 | 3.00E-09 | 67 | 0 | 2.3775 |
| OR44 | Unigene27870_All | 398 | ---- | gi\|299522860\|ref\|NP_001177567.1\| odorant receptor 191 [Nasonia vitripennis] | 151 | 1.00E-35 | 74 | 0 | 2.3763 |
| OR45 | Unigene8010_All | 498 | ---- | gi\|307171286\|gb\|EFN63211.1\| Putative odorant receptor 13a [Camponotus floridanus] | 120.9 | 2.00E-26 | 57 | 0.817 | 2.3212 |
| OR46 | Unigene30655_All | 292 | ---- | gi\|283436101\|ref\|NP_001164417.1\| odorant receptor 243 [Nasonia vitripennis] | 53.9 | 2.00E-06 | 77 | 0.0929 | 2.2793 |
| OR47 | Unigene27949_All | 840 | ---- | gi\|299522730\|ref\|NP_001177506.1\| odorant receptor 66 [Nasonia vitripennis] | 260.8 | 5.00E-68 | 69 | 0.5167 | 2.2101 |
| OR48 | Unigene29150_All | 431 | ---- | gi\|299522722\|ref\|NP_001177503.1\| odorant receptor 62 [Nasonia vitripennis] | 61.2 | 1.00E-08 | 70 | 0.0629 | 2.0318 |
| OR49 | Unigene31045_All | 269 | ---- | gi\|283945514\|ref\|NP_001164659.1\| odorant receptor 301 [Nasonia vitripennis] | 71.2 | 1.00E-11 | 72 | 0.1008 | 1.9533 |
| OR50 | Unigene33224_All | 256 | ---- | gi\|299522892\|ref\|NP_001177583.1\| odorant receptor 222 [Nasonia vitripennis] | 72 | 9.00E-12 | 62 | 0.3179 | 1.9156 |
| OR51 | Unigene15272_All | 315 | ---- | gi\|299522793\|ref\|NP_001177534.1\| odorant receptor 125 [Nasonia vitripennis] | 138.3 | 1.00E-31 | 79 | 1.7222 | 1.8904 |
| OR52 | Unigene3380_All | 779 | ---- | gi\|299523231\|ref\|NP_001177473.1\| odorant receptor 17 [Nasonia vitripennis] | 237.3 | 6.00E-61 | 74 | 1.2187 | 1.8886 |
| OR53 | Unigene30182_All | 378 | ---- | gi\|299522754\|ref\|NP_001177518.1\| odorant receptor 99 [Nasonia vitripennis] | 141.4 | 1.00E-32 | 67 | 0 | 1.8534 |
| OR54 | CL3740.Contig1_All | 438 | ---- | gi\|307207959\|gb\|EFN85518.1\| Putative odorant receptor 22c [Harpegnathos saltator] | 85.5 | 8.00E-16 | 55 | 0.3096 | 1.8394 |
| OR55 | Unigene7526_All | 1166 | ---- | gi\|299782511\|ref\|NP_001177705.1\| odorant receptor 190 [Nasonia vitripennis] | 157.9 | 9.00E-37 | 57 | 2.7915 | 1.8325 |
| OR56 | Unigene16911_All | 472 | ---- | gi\|283945550\|ref\|NP_001164670.1\| odorant receptor 76 [Nasonia vitripennis] | 97.8 | 1.00E-19 | 56 | 0 | 1.7811 |
| OR57 | Unigene32162_All | 219 | ---- | gi\|299522757\|ref\|NP_001177520.1\| odorant receptor 101 [Nasonia vitripennis] | 84.3 | 1.00E-15 | 75 | 0 | 1.7594 |
| OR58 | Unigene33617_All | 227 | ---- | gi\|299522823\|ref\|NP_001177550.1\| odorant receptor 157 [Nasonia vitripennis] | 67.4 | 2.00E-10 | 86 | 0 | 1.6974 |
| OR59 | Unigene11662_All | 730 | ---- | gi\|283945552\|ref\|NP_001164671.1\| odorant receptor 77 [Nasonia vitripennis] | 184.9 | 3.00E-45 | 63 | 1.0404 | 1.6315 |
| OR60 | Unigene12141_All | 979 | ---- | gi\|299523236\|ref\|NP_001177474.1\| odorant receptor 19 [Nasonia vitripennis] | 379 | 1.00E-103 | 77 | 2.1057 | 1.5028 |
| OR61 | Unigene33085_All | 380 | ---- | gi\|283436197\|ref\|NP_001164458.1\| odorant receptor 98 [Nasonia vitripennis] | 130.6 | 2.00E-29 | 76 | 0 | 1.4749 |
| OR62 | Unigene34410_All | 240 | ---- | gi\|283436095\|ref\|NP_001164416.1\| odorant receptor 168 [Nasonia vitripennis] | 114 | 2.00E-24 | 82 | 0.226 | 1.4595 |
| OR63 | Unigene5208_All | 345 | ---- | gi\|299523238\|ref\|NP_001177476.1\| odorant receptor 21 [Nasonia vitripennis] | 89.4 | 5.00E-17 | 59 | 0.7862 | 1.3199 |
| OR64 | CL236.Contig11_All | 1731 | ---- | gi\|299522811\|ref\|NP_001177543.1\| odorant receptor 140 [Nasonia vitripennis] | 67.8 | 3.00E-59 | 71 | 0 | 1.3141 |
| OR65 | Unigene34498_All | 287 | ---- | gi\|299522728\|ref\|NP_001177507.1\| odorant receptor 67 [Nasonia vitripennis] | 83.6 | 3.00E-15 | 75 | 0.2835 | 1.2205 |
| OR66 | Unigene34754_All | 205 | ---- | gi\|299522740\|ref\|NP_001177513.1\| odorant receptor 87 [Nasonia vitripennis] | 80.5 | 2.00E-14 | 74 | 0.1323 | 1.1961 |
| OR67 | Unigene12391_All | 309 | ---- | gi\|299522777\|ref\|NP_001177529.1\| odorant receptor 115 [Nasonia vitripennis] | 97.1 | 2.00E-19 | 65 | 1.3167 | 1.1336 |
| OR68 | Unigene480_All | 958 | ---- | gi\|299522827\|ref\|NP_001177552.1\| odorant receptor 161 [Nasonia vitripennis] | 155.6 | 3.00E-36 | 57 | 2.1518 | 1.0238 |
| OR69 | Unigene16517_All | 312 | ---- | gi\|299522779\|ref\|NP_001177530.1\| odorant receptor 117 [Nasonia vitripennis] | 54.3 | 2.00E-06 | 81 | 1.1302 | 1.0104 |
| OR70 | Unigene11872_All | 652 | ---- | gi\|299522878\|ref\|NP_001177575.1\| odorant receptor 203 [Nasonia vitripennis] | 247.3 | 4.00E-64 | 77 | 2.2564 | 0.8736 |
| OR71 | Unigene17298_All | 560 | ---- | gi\|299522936\|ref\|NP_001177605.1\| odorant receptor 264 [Nasonia vitripennis] | 91.3 | 2.00E-17 | 52 | 1.2796 | 0.867 |
| OR72 | CL4220.Contig1_All | 336 | ---- | gi\|299522821\|ref\|NP_001177549.1\| odorant receptor 151 [Nasonia vitripennis] | 29.3 | 2.00E-07 | 49 | 1.0495 | 0.834 |
| OR73 | CL236.Contig6_All | 3926 | 1179 | gi\|283135164\|ref\|NP_001164404.1\| odorant receptor 141 [Nasonia vitripennis] | 45.4 | 1.00E-43 | 60 | 0.2625 | 0.687 |
| OR74 | CL662.Contig6_All | 984 | ---- | gi\|299522866\|ref\|NP_001177569.1\| odorant receptor 193 [Nasonia vitripennis] | 196.1 | 2.00E-48 | 75 | 1.0199 | 0.6764 |
| OR75 | Unigene740_All | 878 | ---- | gi\|299522712\|ref\|NP_001177498.1\| odorant receptor 53 [Nasonia vitripennis] | 30 | 1.00E-19 | 67 | 2.039 | 0.5585 |
| OR76 | CL2749.Contig2_All | 698 | ---- | gi\|299522908\|ref\|NP_001177591.1\| odorant receptor 233 [Nasonia vitripennis] | 85.5 | 2.00E-15 | 58 | 0.1166 | 0.4517 |
| OR77 | CL117.Contig2_All | 1104 | ---- | gi\|299522906\|ref\|NP_001177590.1\| odorant receptor 232 [Nasonia vitripennis] | 219.9 | 1.00E-55 | 63 | 0.1228 | 0.3807 |
| OR78 | Unigene14563_All | 828 | ---- | gi\|299523273\|ref\|NP_001177491.1\| odorant receptor 44 [Nasonia vitripennis] | 129.8 | 1.00E-28 | 56 | 1.8345 | 0.1269 |
| OR79 | CL2647.Contig5_All | 995 | ---- | gi\|299782507\|ref\|NP_001177703.1\| odorant receptor 160 [Nasonia vitripennis] | 102.8 | 2.00E-20 | 50 | 0 | 0.012 |
