# Supplementary material for: Transcriptome and Expression Patterns of Chemosensory Genes in Antennae of the Parasitoid Wasp Chouioia cunea
Source: PLoS One. 2016 Feb 3;11(2):e0148159. doi: 10.1371/journal.pone.0148159 (PMC4739689; doi:10.1371/journal.pone.0148159)
Supplement: S5 Table — (DOCX) [file pone.0148159.s010.docx]

S5 Table. List of GR genes in *C.cunea* antennae

| Gene | Unigene | Length of Unigene  (bp) | ORF  (bp) | BLASTx annotation | Score | E-value | % Identify | RPKM value | |
| --- | --- | --- | --- | --- | --- | --- | --- | --- | --- |
|  |  |  |  |  |  |  |  | Male | Female |
| GR1 | CL1580.Contig1_All | 3602 | ---- | gi\|163716794\|gb\|ABY40621.1\| gustatory receptor [Tribolium castaneum] | 57.4 | 6.00E-06 | 47 | 9.0515 | 84.87 |
| GR2 | Unigene2804_All | 772 | ---- | gi\|299523195\|ref\|NP_001177460.1\| gustatory receptor 43 [Nasonia vitripennis] | 81.6 | 4.00E-14 | 53 | 2.2486 | 8.17 |
| GR3 | Unigene13958_All | 865 | ---- | gi\|357616042\|gb\|EHJ69979.1\| putative gustatory receptor candidate 59 [Danaus plexippus] | 62 | 4.00E-08 | 55 | 7.714 | 7.84 |
| GR4 | Unigene19548_All | 332 | ---- | gi\|283135122\|ref\|NP_001164388.1\| gustatory receptor 47 [Nasonia vitripennis] | 60.1 | 3.00E-08 | 70 | 0 | 4.64 |
| GR5 | Unigene6905_EA | 267 | ---- | gi\|299523136\|ref\|NM_001190512.1\| gustatory receptor 15 [Nasonia vitripennis] | 52.8 | 6.00E-06 | 54 | 1.0168 | 4.05 |
| GR6 | Unigene7399_All | 1788 | ---- | gi\|163716754\|gb\|ABY40601.1\| gustatory receptor [Tribolium castaneum] | 341.7 | 7.00E-92 | 57 | 2.7306 | 3.72 |
| GR7 | Unigene10905_All | 785 | ---- | gi\|345483933\|ref\|XP_001603636.2\| putative gustatory receptor 64 [Nasonia vitripennis] | 462.2 | 1.00E-128 | 93 | 0.622 | 3.53 |
| GR8 | Unigene20714_All | 572 | ---- | gi\|299523084\|ref\|NP_001177424.1\| gustatory receptor 4 [Nasonia vitripennis] | 167.2 | 4.00E-40 | 66 | 0.0474 | 3.31 |
| GR9 | Unigene10420_All | 827 | ---- | gi\|299523199\|ref\|NP_001177462.1\| gustatory receptor 49 [Nasonia vitripennis] | 361.7 | 2.00E-98 | 80 | 1.0168 | 3.05 |
| GR10 | Unigene5980_All | 475 | ---- | gi\|299523175\|ref\|NP_001177454.1\| gustatory receptor 37 [Nasonia vitripennis] | 68.9 | 8.00E-11 | 80 | 1.085 | 2.95 |
| GR11 | Unigene1949_All | 730 | ---- | gi\|283436203\|ref\|NP_001164461.1\| gustatory receptor 58 [Nasonia vitripennis] | 169.1 | 1.00E-40 | 64 | 1.3005 | 2.83 |
| GR12 | Unigene3057_All | 1161 | ---- | gi\|283135112\|ref\|NP_001164386.1\| gustatory receptor 3 [Nasonia vitripennis] | 181.8 | 5.00E-44 | 73 | 0.8644 | 2.69 |
| GR13 | Unigene28196_All | 393 | ---- | gi\|299523162\|ref\|NP_001177449.1\| gustatory receptor 30 [Nasonia vitripennis] | 102.1 | 8.00E-21 | 62 | 0.138 | 2.58 |
| GR14 | Unigene29601_All | 372 | ---- | gi\|284172475\|ref\|NP_001164387.2\| gustatory receptor 5 [Nasonia vitripennis] | 110.5 | 2.00E-23 | 68 | 0.2187 | 2.17 |
| GR15 | Unigene29342_All | 679 | ---- | gi\|299523170\|ref\|NP_001177452.1\| gustatory receptor 34 [Nasonia vitripennis] | 64.7 | 4.00E-09 | 75 | 0.3995 | 2.01 |
| GR16 | Unigene18441_All | 334 | ---- | gi\|299523179\|ref\|NP_001177455.1\| gustatory receptor 38 [Nasonia vitripennis] | 61.6 | 1.00E-08 | 56 | 0.0812 | 1.78 |
| GR17 | Unigene550_All | 1113 | ---- | gi\|299523094\|ref\|NP_001177427.1\| gustatory receptor 8 [Nasonia vitripennis] | 267.3 | 1.00E-69 | 67 | 1.6085 | 1.70 |
