# Supplementary material for: Transcriptome and Expression Patterns of Chemosensory Genes in Antennae of the Parasitoid Wasp Chouioia cunea
Source: PLoS One. 2016 Feb 3;11(2):e0148159. doi: 10.1371/journal.pone.0148159 (PMC4739689; doi:10.1371/journal.pone.0148159)
Supplement: S6 Table — (DOCX) [file pone.0148159.s011.docx]

S6 Table. List of CSP genes in *C.cunea* antennae

| Gene | Unigene | Length of Unigene  (bp) | ORF  (bp) | BLASTx annotation | Score | E-value | % Identify | RPKM value | |
| --- | --- | --- | --- | --- | --- | --- | --- | --- | --- |
|  |  |  |  |  |  |  |  | Male | Female |
| CSP1 | Unigene13968_All | 644 | ---- | gi\|56805549\|dbj\|BAD83397.1\| chemosensory protein [Camponotus japonicus] | 109 | 1.00E-22 | 66 | 895.9453 | 495.5143 |
| CSP2 | CL3301.Contig1_All | 2558 | ---- | gi\|31442896\|gb\|AAP55719.1\| chemosensory protein CSP-1 [Polistes dominulus] | 114.8 | 2.00E-23 | 78 | 42.8921 | 166.1192 |
| CSP3 | Unigene10292_All | 1371 | 426 | gi\|91983607\|gb\|ABE68832.1\| putative chemosensory protein 1 [Sclerodermus guani] | 123.6 | 2.00E-26 | 69 | 2.0378 | 112.7765 |
| CSP4 | Unigene8884_All | 1567 | ---- | gi\|385199910\|gb\|AFI45003.1\| chemosensory protein [Dendroctonus ponderosae] | 144.8 | 1.00E-32 | 76 | 4.0159 | 27.9425 |
| CSP5 | Unigene5325_All | 842 | 492 | gi\|91983607\|gb\|ABE68832.1\| putative chemosensory protein 1 [Sclerodermus guani] | 129 | 2.00E-28 | 72 | 18.5554 | 24.0874 |
| CSP6 | Unigene11018_All | 663 | ---- | gi\|21898673\|gb\|AAM77025.1\| chemosensory protein [Rhyparobia maderae] | 117.9 | 3.00E-25 | 76 | 5.7276 | 6.4985 |
| CSP7 | Unigene12280_All | 1074 | ---- | gi\|340396204\|gb\|AEK32392.1\| chemosensory protein 1 [Culex quinquefasciatus] | 92 | 5.00E-17 | 68 | 0.5809 | 5.1206 |
| CSP8 | Unigene12726_All | 716 | 531 | gi\|112032244\|gb\|ABH88204.1\| chemosensory protein 11 [Bombyx mori] | 85.9 | 1.00E-15 | 59 | 1.6669 | 1.7612 |
| CSP9 | CL4431.Contig1_All | 832 | 405 | gi\|270011118\|gb\|EFA07566.1\| chemosensory protein 12 [Tribolium castaneum] | 166.8 | 1.00E-39 | 82 | 3.4231 | 0.9683 |
| CSP10 | Unigene3640_All | 898 | ---- | gi\|205326625\|gb\|ACI03402.1\| chemosensory protein [Apis cerana cerana] | 110.2 | 1.00E-22 | 68 | 3.8059 | 0.8972 |
| CSP11 | Unigene14702_All | 668 | 423 | gi\|443908529\|gb\|AGD80088.1\| chemosensory protein 8 [Apolygus lucorum] | 62.8 | 1.00E-08 | 71 | 1.2994 | 0.3671 |
