# Supplementary material for: Transcriptome and Expression Patterns of Chemosensory Genes in Antennae of the Parasitoid Wasp Chouioia cunea
Source: PLoS One. 2016 Feb 3;11(2):e0148159. doi: 10.1371/journal.pone.0148159 (PMC4739689; doi:10.1371/journal.pone.0148159)
Supplement: S7 Table — (DOCX) [file pone.0148159.s012.docx]

S7 Table. List of IR and SNMP genes in *C.cunea* antennae

| Gene | Unigene | Length of Unigene | ORF  (bp) | BLASTx annotation | Score | E-value | % Identify | RPKM value | |
| --- | --- | --- | --- | --- | --- | --- | --- | --- | --- |
|  |  |  |  |  |  |  |  | Male | Male |
| IR8a | Unigene7533_All | 3173 | 2751 | gi\|471180437\|gb\|AGI05169.1\| ionotropic receptor 8a [Dendroctonus ponderosae] | 777.7 | 0 | 66 | 10.9578 | 33.0084 |
| IR75q2 | Unigene4531_All | 2288 | 1908 | gi\|379070082\|gb\|AFC91752.1\| putative ionotropic receptor IR75q2 [Cydia pomonella] | 329.3 | 5.00E-88 | 53 | 0.6639 | 6.8894 |
| IR64a | Unigene11160_All | 743 | ---- | gi\|7295466\|gb\|AAF50781.1\| ionotropic receptor 64a [Drosophila melanogaster] | 120.2 | 1.00E-25 | 48 | 2.738 | 6.0345 |
| IR93a | Unigene7278_All | 2799 | ---- | gi\|440217690\|gb\|AAF55817.3\| ionotropic receptor 93a [Drosophila melanogaster] | 497.7 | 1.00E-138 | 53 | 0.6008 | 4.4427 |
| IR1 | Unigene12975_All | 1771 | ---- | gi\|399163347\|gb\|AFP33229.1\| ionotropic receptor [Locusta migratoria] | 612.8 | 1.00E-173 | 89 | 1.7154 | 4.2327 |
| IR76b | Unigene21355_All | 283 | ---- | gi\|316994957\|gb\|ADU79033.1\| ionotropic receptor 76b [Drosophila melanogaster] | 56.6 | 4.00E-07 | 60 | 0.3834 | 3.9608 |
| IR21a | CL2191.Contig2_All | 691 | ---- | gi\|313505768\|gb\|ADR64678.1\| putative chemosensory ionotropic receptor IR21a [Spodoptera littoralis] | 153.7 | 7.00E-36 | 63 | 0.4318 | 3.2443 |
| IR25a | CL1937.Contig2_All | 1816 | ---- | gi\|401063649\|gb\|AFP89966.1\| ionotropic receptor 25a [Musca domestica] | 363.6 | 2.00E-98 | 60 | 0.4033 | 3.0284 |
| IR75q.2 | CL1137.Contig1_All | 3212 | 2043 | gi\|313505782\|gb\|ADR64685.1\| putative chemosensory ionotropic receptor IR75q.2 [Spodoptera littoralis] | 380.6 | 3.00E-103 | 57 | 1.1147 | 1.5159 |
| IR75p | CL1137.Contig2_All | 341 | ---- | gi\|379070088\|gb\|AFC91755.1\| putative ionotropic receptor IR75p, partial [Cydia pomonella] | 87 | 2.00E-16 | 71 | 0.3182 | 0 |
| SNMP1 | CL194.Contig2_All | 10416 | 1050 | gi\|156537374\|ref\|XP_001606675.1\| PREDICTED: sensory neuron membrane protein 1 [Nasonia vitripennis] | 468.4 | 3.00E-129 | 60 | 0.3906 | 3.7699 |
