# Supplementary material for: Transcriptome and Expression Patterns of Chemosensory Genes in Antennae of the Parasitoid Wasp Chouioia cunea
Source: PLoS One. 2016 Feb 3;11(2):e0148159. doi: 10.1371/journal.pone.0148159 (PMC4739689; doi:10.1371/journal.pone.0148159)
Supplement: S8 Table — (DOCX) [file pone.0148159.s013.docx]

S8 Table. The protein names and sequences of OBPs that were used in phylogentic tree analysis.

| **GI Number** | **Name** | **Protein Sequence** |
| --- | --- | --- |
| 333102335 | MmOBP8 | CYLFCLFKDINIMNQKGEFDPNLAAQEVQDNLREAARKYIFMCYDLVKPNMTSDGCKNALEMVQCFKEKAPEMYEMLGLFHPPSNEPLKMT |
| 126508768 | MmOBP1 | MKVAIIFLAIIAVALAATTKTYTSKFDDVDVDGILGSDRLLRNYVNCLLDRGPCTKEGVTLKEILPDALATSCESCTEKQKTKSEKVIRHLVNNKKELWDELAVKYDPNNEYRKKYEDQ |
| 148641513 | MmOBP1 | RVILNYIFLGPLLQTFIVSAKLPDWVPAEIIDMAQGEKGRCMSEHGTTEDMINMVNEGNIPNDPKLTCYMFCLFESFSIIDEDGVLEYGMLTEMFPDDIKAKAESVLSGCAEQPGADNCEKVYKIATCVQSKSPDM |
| 148641512 | MmOBP4 | CFTLTAAGILFTVLITVNNASSNSNMEELVKKSMEETFKACKDKLTPENFALLNKDPHADNQEIKCFKACGMNHAGIMADGKIQIEKMEEKLNSLLGEDKKDFSKIIIGRAKPCVEEANKGENECDVAAGFEACVQKTINTK |
| 148641511 | MmOBP1 | KNIIIFTTAITIFTFINFSQTEARMTMTQIRNAMKPLGKTCLGKTGLSKEVQAGQHNGEFPEDEALMCYHSCLLKLAKISDKSGNINLDTVHKQIDLMMPEDLIARAKAVTTDCFGEIKSTEICRMSFEFVKCYFIKGPEI |
| 126508766 | MmOBP6 | KNTLFFTLAAAFLLGYNIPHVESRMSMAQTINTMKPLGKTCAAKTGLSKEMQDGQHEGQFPEEEALMCYHTCLLKMAKVADKTGKLNIDAMVKQIDMLMPEDLVDKAKTACSGCADEVTATEGCRPSWEFMKCWYGRAPEL |
| 119888042 | MmOBP5 | MKNFVVIVILALYFTATTESLQEIMNTFQKARLEVRAPCLHLLSNETLTTLKTRRHLDNPEIRCFKACLMERQGYLKDNKIFIDEYEKLIDVNLKRIKELNMKFARACVNEAEKSENKCELAHNYNRCILHQTRKH |
| 119888038 | MmOBP3 | GSVLAIVACALVVGVLGDDDMKEKHKEIFKKCAEETGVTKEDLHNHKRGEEPETKIKCFHACIAKADGAMVDGKLNKDKVIEKIPADLPDRERIIEAVTKCSEQTAADECETAHLVFKCLRENKALP |
| 119888036 | MmOBP2 | KSIIFLGVLLTVLISNKAEAKSVQKRECPFKKPFEANAPKCMDKISEENMGRMMQGNMDNDEIRCFVGCVFENAGFVKDNKVQMDKVREAVDNFVDDYKYSKEVGDQVYGVVSDCAPQAEKGANNCEVSSNLLICFKTNNKFT |
| 346426891 | MmOBP10 | MAKFLLSSVGVLVLIAYVQSGPVPEEFKDVQPTIRAACVKESGLTNEELVNKAALGEFTDDPQLKCYLKCIFDQFRLVSKRGINFDAMLALSPPSMKENAIKMVKECRDTKGKEGDLCDLSFEVTKCLYNSNPET |
| 301341842 | NvOBP | MKIVVLCLVVLSAVACVSAGYREYQNACLDENGLTKEEFYAMKRNQDPRSGCVTACIMKKNGSMKHGIIDARGIKRRMRTLLAPFISKDKLYEKIDYCVDEAENHVGVCEKAYVLQKCLRTPRANN |
| 355390029 | NvOBP78 | MKTIVFTLCMMTVAVTCSPRPGGRGGGSMFSRESVKKCMAEMDIKREDIKTLKQNNDPKLSCLNACAMTKEEIMDEAGNIDADKLIKATLEIVQKKKPDINVEELETAMLSCIEKAKEVEDKCMKAKTLVVCSHEYWKAN |
| 355389901 | NvOBP14 | MKAFLCTFSIVLAAAMSVNGDMPGELKPAFQECHNELLGTPHEEPTGPPNMDDPKVKCIHACVAKKIGHMVDGKIVAEKEIESAKQHMPNADNSLTDKITECANKANEQSDECEVSAAFHKCIVEKVGPP |
| 355389887 | NvOBP7 | MKAFLCTFSIVLAAAMSVNGDMPGELKPAFQECHNELLGTPQEEPTGPPNMDDPKVKCIHACVAKKIGHMVDGKIVAEKEIESAKQHMPNADNSLTDKITECANKANEQSDECEVSAAFHKCIVEKVGPP |
| 355390139 | NvOBP3b | MKSLLLCFLVVILGVTKVKSNEIPQEIQAMVVGVRDKCHRETGVDIEHVDRTVEGYFHPSELLGCYFSCIFNHFNLLDNDGHLDWVKVVNVIPPSFKDHADEMIAACKTTTGKDPCDSAVNIVQCFQKTNPAV |
| 355390137 | NvOBP90 | KNLALLLLTLCVVSCLLINGARAGVSREQMEKMANGFRNTCVGKTGADMSLVEGIRVGNFVEDPTSMCYTKCIMGLMKTFTKQGNIDVEMLVKQINVMASPDIAGSMVTNARKCHAETSASDPCELAWLFTKCIYAADPAV |
| 355390133 | NvOBP88 | MKLLIFVISFFIVAAHSQPRSMDWKGCMEEIGVSKDDVKSTEWGDPKSRCVLACTFKKVGVINDGKVVFDVAFDITKGEAQDSSHDKYIEEKVNSCIEKAHQETNECDVSYVFMECMKTNNNTA |
| 355390131 | NvOBP87 | MKFLIFVISLFTVVARSRQSLADIEACASQYGVENVTRIPDNDRPFKQRDPDYECLRACLWRKQGIMKNGKFDLDKAFNYFKKTTRFPLTVFKEKLSVCVEKGNQEKNECGVTRVYVDCMNGSPKAR |
| 355390129 | NvOBP86 | MKSYSVILLAICFAAIYSSSALISIEDKAACLKKNGLNNTEKWDLTAQFDYRLEKPFTCYVACVINAIKKPEETVYGKLSEVIERGHVIPASLKKDMENRLDSCYRYNGEGDDCKLLYCVKILQSPLIKLS |
| 355390127 | NvOBP85 | MRSVLLIFCLSSVAVRVSAHVSPVADSFKACLAESGMTRDDFIKALQSSDDSKAQCIAACTMEKEKFMSDDKINVDAIIAKMEDVSQEIGKVQITDLVMNCAAEAKDKSGKCGVAHSVVRCIHEELRKE |
| 355390125 | NvOBP84 | RSILITSILIILISQYKLVKCKKMNLDELRDMLRPMSKSCKSKTGVSDEMVAATHQGIFPREKPLMCYFKCLSVMLKVMNKQGEIKPKDVERQIDLLVIPELAPTLKKIGTDCYNKVAPTNDACAYAFEIVMCGYQTDPKY |
| 355390123 | NvOBP83 | RLTLQLITLVSLVAIFKTTESKMTMDQIKNTLKPFKNSCIKKISPDVAMVEATKSGQFPEDATLMCFLKCVLSMMKVMKNGEILLPSIMQQIDIMMPDEYVETMKEICTNCYEMSLKVDDACEKAYVFVKCYYNTNSEL |
| 355390121 | NvOBP82 | MKRVMALVGAFLLVSAVQCDDMPFWNEKVECAQSMGISPDQMTSMLTSNDAQMNCVHACVLEKIGGMVDGKLSLDSLMESLEKLKAEVKDYDATKAGIHQCFDQASGDRCESAGKFAMCMQEHMQG |
| 355390119 | NvOBP81 | MKVIVLLVTVLTITIHVSCQTDEEVHKIKEKCFDLSDIPVEDRVVYNPENPKLKCFNACTYTGVGMMKDGKIVPEKYIERLQDSLKNEKKSDVEAFMKHMEDCAVMANKLSDECEVAYSMIKCL |
| 355390033 | NvOBP80 | MGGFVTVLYFLSIIICVYSLNWSEAKKHVQECLDEYQITREDVAKLKKEESPDYNCYIACIMKKRGSLVDGKIDEEKMLEILKQLHVLNSERTEDKFRICATEANKQSNECLVAGDMIGCLYFKSN |
| 355390031 | NvOBP79 | MKLFFVTLCVLFAAVYGATKSDSKSEKIFHECLEENDIKESDFKNLEGKKDPKMRCLMACILEKEGALKDGEIDGDVIKKDIIAEFTEVDAQKISDAIDTCVDGANDLSDICEKTSFIGECLKVELDKL |
| 355390027 | NvOBP77 | MKIVVLCLVVLSAVACVSAGYREYQNACLDENGLTKEEFYAMKRNQDPRSGCVTACIMKKNGSMKHGIIDARGIKRRMRTLLAPFISKDKLYEKIDYCVDEAENHVGVCEKAYVLQKCLRTPRANN |
| 355390025 | NvOBP76 | QGSLCALVVLSLVCLVRAGPPDWISAEILEMVQSDKGRCMAEHGTTEALIDDVNKGNLPNDKAITCYMYCLFEAFSLVDEEANIEVEMLVGFLPEHMQAVANELIDVCAKLDGADVCDKMYVMAKCVMEKRPDL |
| 355390023 | NvOBP75 | RVLLVVVSVCFVGSYADYADDIRKLQEETKRIEEYRRPCLKEVGLYADPANGITSQPASSPTIGQIFCLWACLYRKNGSIRPDGSVDEAAVRSKNPELEGPLDVIISKCENQAGENTCKLAGCLAKAHFNLLE |
| 355390017 | NvOBP72 | MLFFTVVLLFSSVCTATKEEEEFKSELAECKNLVGVTEDYVRDVFKSGLKGADEKFKCFIACLIQDSYKFNDGGVFDAERTIANDRGPAGLLRDYTNKALKACSNIKGYSECDAIFKVYKCMVENVEKL |
| 355390015 | NvOBP71 | VAIVACVLTICSIFAGSKADLTEDQRKILQPLKDECFQETGLDAVTLEKFKKEALQKFKTTGEVSNDEKVNCFSACMFKKIGFMSEEGKFEEDTVRALMSENFPPETLDKAIENCKNEVGKDHCETAAKLIVCFMNNKAGM |
| 355390013 | NvOBP70 | ISGEEISLLVIFTICWGINLKCKHAGEIQLHLQDKEAAEKCSKDIGITLETVYATMKNELKDADEKLKCFAACVFKEKEMLKDDGPINVAKAIEDLPDEIKDDVRDAMIKTIEKCSQKKEANECETVFHAVQCATLDMSKL |
| 355390011 | NvOBP69 | MKLFAVVLVFFALGSSSVALDEEERGVLRQIRNVCVVESGLSPYELGFIYRAIRPAKKLAQASRCVIQKISELQSENETVKHIADRGKAALANAPISNIADNVLGSCQNLLGQNGCIQVLELAAKIIDNLRSR |
| 355390009 | NvOBP68 | LFAFAVFAFTNVLNPMYFHTFYETTFFLSCVESIFKLTESERSCAFQTSFLRELGLINKDNSFNVNDLLKQRKSGIPESKIHDAVKTCDVESLDSLEKTSKAVKCLM |
| 355390007 | NvOBP67 | MKTSALLLVAFGIFAFTELSTASLDKWFEECVKSYGHTEESVSKLPDLEKSCVIHICFMRDVGLINEDNSLNVNYLLERRKSHVPESKIYDAVRTCNAESIDTLAKTCEAVKCLM |
| 355390005 | NvOBP66 | SVLVVFAAICIAGVLSDPKGDIDACVAESKVDTKLFEDMMHTPDFKATREMDCFAACMFKKDGVLDADGNVDASKLPNVDVSKVCGALRGKDACETAGKIIGCFAEKGVMD |
| 355390003 | NvOBP65 | SILFIFAIVCVVGVFSDDDKKDLTREQILECVAESGVDETKVEDIKLGNQGLETTREIDCFAACVFKKQGIMNEAGVITPDKPMDNEAAKQCVATTGADACDTAGKVLKCFISNNLVS |
| 355390001 | NvOBP64 | MKKFTLIFVSCYLVFSSMHRVMCVTQCFFNELNLVDQRGFPERSAVIGIMTQNIQDPELRDFVEESVIECYHYINNNNSGRQEKCQFSQSLLSCLAEKGSER |
| 355389999 | NvOBP63 | GTFLPSIDDTLHEYETNCARVSGATHSAIEIARNTKMLANTARLNAFAMCMLQQFNVMDSNGIVNPDVMSYSIISNVPNATAGISQQCISKRGIDAVNTARMIMNCYLRANQMV |
| 355389997 | NvOBP62 | IYVICVVVFFLAPAVFASFSPLIEDDFHAYEADCGASDESIEAARRARQLPQSPQMNAFALCMMQKYKVMAADGSVNPDVRSYGIITDGPDNTWRVSEHCRTLNGNSAGETARMIMNCYLDNNQLV |
| 355389995 | NvOBP61 | IYVICAVLLFAPAALGLFSNGIWDVLHANEAKCQLNSGASDASIEDARRARKLSESPEMNAFAKCMLGIYNVMRPDGSINPDFQSYTVPTDVPNNTWRISQKCITLGGTDSGDTARKIFNCYTENNQLV |
| 355389993 | NvOBP60 | IYVLCAVLFFTPTVFGIYSSAIWDALLHANEEPCGRSAGLSEESIESSRRARYLPESPEMNVFAFCVIRVLNIMSKDGKVNPDIGSYLVPTNTPDITKVISEKCRTHVGVDAGDTARTILNCYLQADQLV |
| 355389991 | NvOBP59 | FYALCVILLCSSAAFALLEARVRDYLYEYQRDCMIESGADTSLVAAADRARIIPNDGLLDTFAICMLKKYNILHKDGSVNQDHDSYTIFSDNPDVYRISERCKAKIGKDAGETARKIMNCFAEDGDSL |
| 355389989 | NvOBP58 | FFISCVLVIFCSSSAIGLLSHEAILSLQRDQDDCVRESGVTRSTVEQAHLDRVIHNDENMAKFAACMLKKFNVMSDDGKINEDVYSYHLISDNPAMFETAEKCKKRTGSDVDETASKIMTCFLNSDVFV |
| 355389987 | NvOBP57 | LTFNFRTNTDILFQASTGNIVNDTLNRKFLLLVKTCANKKLHMSDYGSINEDVNSCHLIFDNSSMLETVEKCKNRRETASRDMTCFLKSHVLIIDPY |
| 355389985 | NvOBP56 | LFVFCVFALCLTAANALFGPKLKEKLLEREDACLRETGNTLLSIDHVRRTKTLPEDGSLDKFALCLLKKHRIVNDDDTVNKDKHRYYLILDDGRKKEYAEDCVLSSGGSNNGEIARHLLSCLLKTDIFF |
| 355389983 | NvOBP55 | NSLVVIVLICFSQIHAIPLTKKKEKFVPAEDSKEQCMIKFGLDPDFVDYLIGLHRPQIEINAYIGSKHSCIHACMVKLDQNLNPYDYVVDRVSADTKEEYERLIKLVNKCNEKDSGNGCVLLDCVRRNKELRDFVY |
| 355389981 | NvOBP54 | LCWIILIALCIFGINARPNSEPDNDGGFEPLALQCLRELKKDPTLSAKNCDEIESSLTDDERNCILACMFRRNDPDKKSLYEYLKSQLSTIDNRIQVYRDELLEKLNSCKALVGEGNDCGVMKCIELFKPPFAHWY |
| 355389979 | NvOBP53 | KILLFLLIFCVVGIYTQKHNDSAEKNKHAMDIEDCLNQHSNITKKGLSVKDIILKSIAPYDLGCITSCLKKKELKNGVTLNSYVIQNAYLPSTKFPDWYEKKNEDYQYVVIANRCINEAKEDECKLFMCLKAWELPFAHIL |
| 355389943 | NvOBP35 | MKLFFALFVLSFALLHSATGAKDSLVECLQENGLKMVDLDFMRKIKPNTDMPRNKLIEDKLACAFACSFNRDNSWKDENVFTFMTDVIKKDYRIPVGLKKQMLDTLKSCNAEAKGDDCTLLQCIKVTRYPFMDFV |
| 355389937 | NvOBP32 | MKSYVLAFAICFAVIDLSFALGKEDQEKCLRKNGLNNSTDVELMKAFVRSDGKNEFHLEREFSCVVACVIDERRTEDNVNTSTYQLLTDLISEAHNKIPDEQWRDMKTTLDKCHQQDEGDDCKLLYCVKILRDPFKELI |
| 355389935 | NvOBP31 | MKSYILPIAICFAVIDMIFIRVLQCSFLPLEKMNKRHAYIVTGYTIVTDVELLRELMLRNDSKDEKAIDLENKFTCAVACFSDAKINVSREEIKSDLMNTLDTCHQKDEGDNCNLLECVKVLIPPFKALL |
| 355389933 | NvOBP30 | KLHALLVLCFATASANIRLTDQQLKEYVQVCLAKTRLSQGFYQSGDEAQKILTEEQKSCFLACMFKRTGIIDHDGSVNLKLGDEELPRTPAIEACITTAKEDICKLAICLHKTGKFSITSV |
| 355389931 | NvOBP29 | FVALALCVIAVNGEVTESSSTEFPSAVDIYKMKIFKYSMECLFERKLDLSKFALQKDVKKAVEDLHKDEKACFAGCVFKKLGAMNDDGTFNEDKLFMGATAETLPIFKQTHDAAVKHCTDKVGKDELCKFAACIVIQAPAYASSL |
| 355389929 | NvOBP28 | MKIFVIVALCAVAVYAEENEVLKQYERDCMTENGIDPTVQDPKNLTLEDGNCYYACYFKKFGIIKEDGSYDVAAIKEKYSKPNSVEAVQKKLDEITQTYCQDKVGNHCNLAACLSKISKEQWKI |
| 355389927 | NvOBP27 | VWGETSATVEPPTKLKTCANVTRITVAVNLLDKECMKTSSSSAILLNGDENNVEVKDIEMNVYALCLLQKSSIMNEQGKINLNFDIFKIVKNLYKRTDQRGFGLAFIIKSLEKCRQTDGPDQFSTATKIMKCLLDNQKTV |
| 355389925 | NvOBP26 | TFAIVLTLCIVGAYASTLKDDQKAKLREYKESCITETSADKAVIDSIIKGGPINRDEKLDCFSACMLKKIGIMRPDGSIDVESARAKAATTNVDVAKANEVIDKCKDLKGKDTCETGGAVFGCFITNKDFP |
| 355389923 | NvOBP25 | MKKIVFIISTFCFVMIQGIRKELNPKKINLTEFTEAMQSCGTMLGFDREFKLHLFGSQEDYNKTLCLSFCALRKLKFYVIEDEIKKELAHVRNARLKEEVYKALDVCKHLLDDPCKLFDCFFDYAKVVDAEN |
| 355389921 | NvOBP24 | NAAIIILAFCLAGALARGIIDKNESGVEVNDACLLEYGINPDQVYNDGSDGSEAVTALTDEQIYCVAACIYKDYGIMRPNGTIDTEKADSYFGEDDSRERDIFFAVYNACSEGRVGCKLVQCMFSELKNHWGSS |
| 355389919 | NvOBP23 | MKTVIVFFFILVGILAETTTNVDSRDDDMTTCLVEYGLDPGPNNPTEDQKNCYFACMFKTIGYMKKDGSFNLDLILSDAYRSEKRVESKRKLDNIVSMCKQRAGNDICKLAGCYQEHRN |
| 355389917 | NvOBP22 | TSPAVVLALCFVNVFGNSITELKNGSSRVKVNVRQCLTDYHIDPAVLELDIDRNDDLYSKLSEEKKGCVTACVYRGFNWLRPDGSLDIDLLCEGETPEESEAERKRYTKIVAECRAEVGKDDCKFFNCLNLKDL |
| 355389915 | NvOBP21 | TLFFVVLGLVAVSAAVPVEHSFPQFENGTPKMKEQVNTCLRNYKIDAAVLELGDEKNFEKTDKLTKLEWGCVRACVYKGANFMRADGSLDIEVLTDGDEPEDKKKFESVVGICRAEAGKD  DCKFFQCMDEKDDS |
| 355389913 | NvOBP20 | ELIVIVGFLVAAMPSPAAFQIDSSKRMNQTVTECLTGYNIDPAVLDINTEDVHLMMDELSGEQRGCVTACVYKGFDWLKDDGSLDIDALTMDEDPEDTAEFIKDIVDCRNKVGTEACKFFHCLDTKGT |
| 355389911 | NvOBP19 | RILLLSIFGSIIAFSHQQENPELSDAHWQEDLQSCLDQTGLDLSIFGVSRIDEVTEQHLKKLTKVPADKRGCLVACVFQKQGMISKEGVLQNNPPHPDPTTKFETTFEDAIAVCRAEENFCKLGNCLFGIYFNYEI |
| 355389909 | NvOBP18 | SFAVIFAFCFVGAIAALTEEQKAKLKEYKYACITETGVSEDVIESVKKGEQVTFDEKLNCFSACMLKKVGIMNADGTVNEEVARAKVPQDLPKDKVDQVINTCKAEVGKDSCETGGKVLACLMKTKAVS |
| 355389907 | NvOBP17 | FFTVATFAMCIIGTFAAFTMTEEQAKDLQDKLDCIKETGADIATLLNIKNGIPTLYDDKVNCFAACMLEKFNIMKPDGSMDETVARLRASKSMSQEKVDRVLSSCKSEVGKDKCETGGKILECLMKNDAVP |
| 355389905 | NvOBP16 | RVILLSSILLGSIAISRQNPVTYTDKDNNVIIPPCLAETGLNLSVLGVAKIEDVRDSSFYNLKTLTEDKRGCFVACVYKKLGIITEENVLINDRVIPPGVAVPKKKLATAFEDATEACRAQKDLCKLGNCLYEIYFF |
| 355389903 | NvOBP15 | RILLLGFFCSIFALSHQEIPEPSYTHWQEHLQSCLDQTGLDLSIFGVSRIDDVTEQNLKKLAEVTADKRGCLVACVFQKQGMISKEGVLQANPPRPDPTTKLETTFEEAIAACRSEKNFCKLGNCLFEIYFKYK |
| 355389899 | NvOBP13 | MRLFAFANVLGIVLLIHNSATKTNVERFWDYVDVLKDCAKENGISIESYAYASKKNNTDGIYEKSKCVEACMFKSHKIMRPDGTIDMEKAIEHLLTGNPGEKRDLMKKNIESCEIPNGDNECEVAHTMVKCALGYD |
| 355389897 | NvOBP12 | MRLLTALLLIGIVAVVNAKSGSTAFTITQNDRNVFRNCMTKIGIPDDEMVAVLDNHEKDADEKVKCYNGCLYKAFKVIKDDGTVDTEAAIKFFKVEDMESDKNIIVKCSNESNSNKEKNDCDTAQTMESCYYKLKKEQ |
| 355389895 | NvOBP11 | MSRHLIIALALFSAVFMVKSLSPEERVARDKCLKENGFSREPDFIGIDAVDMRSKCYAACALRGYGIMKEDSSIDINKILEHISDTKNKDIDVKKSLIIPCAEKKGETDCDTGYLITNCVALAVRKL |
| 355389893 | NvOBP10 | MDRHLIIALTLFSVVFMVQSLSQEDIDARNKCLKEHGFTIEPKYVSAYKTIDIRAKCYASCLMRETGVVKEDGSIDLNKVLEKISDSENKTLDEVVKKSFIPCTEKKGDNDCDTGHQILTCIVATISIL |
| 355389891 | NvOBP9 | KAVVIVLAVCLAGVFAEDPIKDINKEYIKGCLIENGFDPQQYPTGLRNAKVPEKQEQNRNCYYSCMMKKMNLMKADGSLNEDALRQKFNMNLDTLGKALSTCKDQVKDDKCKLAACLMANRGA |
| 355389889 | NvOBP8 | MKAFLCVLGVIIAAASASCGMPEEMKQAFKECHTELGMPDEKPHGPPNPDDPKIKCFHACIMKKAGKMVDGKLDADKEIEFAKKRMPNADDSMIEKITECVKTANEQSDECEVAGAMHKC  IMEKVGSP |
| 355389885 | NvOBP6 | MKFLTSVLSCFVIHAMLVRCAPFHETLDDDPDLNDSIDLCAAEVGLLVEETRKSFNMPIEAPGNCVVACVWKKIGLMELDGKIVKEEMISSLHPTLKQMPNITPIHEDDFYHCVDEANDYEGGCIVVSEYFKCIIRDLFNQ |
| 355389883 | NvOBP5 | SSAISQFGKLKDAPIPSRQYYLRHRRNRERKKGAGALRIGIEFQRDSLCYALCIAFLAADIDPTAEIEIDRSGSRAMRWIVDEDFPRERHRPTANCVTRTHNAITSCLGSFPLVRCT |
| 355389881 | NvOBP4 | KAVAIILVVCLVQGLQALNKSETPGLNDQMKECLTQNDLDADLYTELWKDHPKLNAPQKKVNCFLACLYKKVGALSADGAIVLPEGLIEERIINWSPELREKCKKQAGDDVCELAGCLDK  PNGFLSATV |
| 355389879 | NvOBP3a | MKSLLLCFLVVILGVTKVKSNEIPQEIQAMVVGVRDKCHRETGVDIEHVDRTVEGYFHPSELLGCYFSCIFNHFNLLDNDGHLDWVKVVNVIPPSFKDHADEMIAACKTTTGKDPCDSAVNIVQCFQKTNPAK |
| 355389877 | NvOBP2 | GQSLLLLALGIFLPHCLAGTRPSFVSDKMIATAASVVNACQMQTGVATADIESVRNGQWPDTMELKCYMYCLWEQFGLIDEKRELSLNGMLTFFQRIPAYRVEVEKAINECKALATGDTCEYAYTFNKCYAERSPRT |
| 355389875 | NvOBP1 | MMKNLTLCFLVVVLGVIKVNGNEIPHEIRHMVVGVRDKCHRETGVDIEHVDRTVEGYFHPSELLGCYFSCIFNHFDLLDKDGHLDWDKLVPRIPESFKEHADEMIAACRSTTGKDPCDSALNIVQCFQKTNPSK |
|  |  |  |
|  | CcOBP1 | TVIVSFLFCIATVLAELTEEQKEMIKPYKDACLAELKLDEAIIEQSKKEYLEQGKTEFSDQLNCFSACMFKKVGIMTEEGKFDEDMARALASGQFPEDEINKAINACKNEVGKDICETAGILFECFLKQRISV |
|  | CcOBP3 | VICILILSVHIINEAHAGASREQMEKISEGFRKTCIGKTGADLAIVQEIRNGNFIVDPLAKCYTKCIMGLMKTLTKQGQIDAEMMIKQINIMVSPDIAGHMIAGVRKCHVEVSADEPCELAWLFTKCVHDENPEL |
|  | CcOBP4 | LFVICCYCNFAAYSRNITFTNDELDENIQMCLAKTRLSQAFFKSGDENLKYLTEEQKSCFLACMFKKAGIITHDGSVRSVTEEMDIATANAIDRCQSLAEGNLCKLAWCLYKTDKFSIPVI |
|  | CcOBP5 | MHSFTVIVVFCALFTKNALADSYNVELTEEDREIFKKCIKEGGLTKEELNAAVRNFDKNADRKVKCFRGCLLRSHKVIKDDNTIDGNAAVNYYHVEDVEPLKKLILKCSASTTGNDYCDV  AQSVESCF |
|  | CcOBP6 | GETSAEDETNITLCVNGTRIDLVVNLLDKECIEKAENS |
|  | CcOBP7 | MKFVIFSFFALFLAVRDIHGNDESIQNLKECLNENNIKVDIEKLKEHDDPKIRCILACVMEKEGILENGDIQYDLLKEDLLEDAEQLGEKKISEIVDYCGNLAKELTDVCDKTNLIGMCL  EEELEK |
|  | CcOBP8 | ALLLLLFGLTNAKPGTRPSFVSDKMIATAASVVNACQTQTGVTTADIELVRNGQWPDSMELKCYMYCLWEQFGLIDEKRELSLNGMLTFFQRIPAYRNEVQQAISECKALGKYFATGDSCEYAYTFNKCYADRSPRT |
|  | CcOBP9 | SFVIVFALCIAGAFAGLTDQQIVKLREYKIGCLAETGVSEDVVNKLKVGEAVVFDEKLNCFSACILKKVGIMRPDGSIDEQVARDKLPKDWPQDKVDHVVNACKVQVGKDSCETGGKVLGCLAKTRAIA |
|  | CcOBP10 | KTFAIVLAVCLAVVYADDPLKDIPKDLIKTCLTENGFDAAQYPNGLRNVKVPDNQEKNRDCYYACMMKKMNLMKPDGMLMEDNLKSKFNLNLETLQKALNTCKAQVKGNDSCKLAACLMANRG |
|  | CcOBP11 | TLLSMFLLFVSVSTTLASFSADIEISLYEYEDDCSKESGLGAADIEIIRKNHAVPDNEIASQWAKCMMQKHNVIKSDGTFNMDVKTIAIPADTDTVIRAFPICKNQIGKDMAGTARLMMNCYLSNSDGL |
|  | CcOBP12 | MKNVVVCFIVIVFGAININAGEIPKEIAHMVADVREKCHRETGVDIEHVDRTAEGYFHPTETLGCYFSCIFGHFDLLDHNGHIDFDKIIPKIPESFKDHGMEMITACRHLTGKNPCDMAFNVVQCFQKTNPEK |
|  | CcOBP13 | YCALVLLLCMQAILLVHAGPPDWISPEILEMVQSDKARCMGEHGTTEALIEEVNQGHLTDDRAITCYMYCLFEAFSLVDEDGELEVEMLVGFLPEHMQGVANELIDACAKEPGTDVCNKMYAVAKCVQQKRPDL |
|  | CcOBP14 | ISVALFVVICIVGVYSHPHGEHGHKLTPEQIARIMADVEECARTNDIGHEVFEDLKAGKNPTPSRNLSCFSACVLKRNGVMNADGSTNHKPTDSDVAKECKDLRGDDDCETAGKIVSCLHKNNLI |
|  | CcOBP15 | LLLALCVAICFAGVYCDTAPKPEEVQYVKDCASKNNMDQKMIDDLKMQKTFFASQAAMCFTHCVMHHNGMLDDEGNMTDMFKKIPEAAECQSMTGNDKCETAAKIMDCMVKKENAD |
|  | CcOBP16 | MKVLLVLVCCLAVAMAQFSSDKQRASAMNECQEELKVPDSEVEDPSKLGCLYACMHKKVGYTDADGTYNLRKLAGSAYNQRFEEAAQRVMNMCAEQAKGDPCKMALCLE |
|  | CcOBP17 | IVITILLVCATQFKIIECGKKMDIDGLKDMLKPMSKSCKTKTGVSDELIAGTANGIWPRERSLMCYFKCLAVMLKAMNKQGEITLREINRQLNILVIDELVPRMKQILEQCLATATPSDDACEYAFNLIVCGYKADPTL |
|  | CcOBP18 | MQLFDVACILGIVVIINALSNEQRYDNYIAVLKNCLKELGLSEEVYAYASVINNTDGAYDKAKCADLCMFKALKIMKPDGHIDLEKALEHLLSGEPGVQRDIMKTNIETCSKKKEDNDCDTAHNMMTCAVG |
|  | CcOBP19 | KSLVQIAPFVLILLSFETVNAKMTMDQIKNTLKPFKTSCLKKTGVDIDLVDGTKSGHFPEERSLMCFTKCVMQMMKVAKNGEILINAMMQQVDLMMPDEYVDEMKSIITNCGPEANTKDDGCESAFTFAKCFYQSNSDI |
|  | CcOBP20 | MKRAFSVLCVFLILGYAYSIDLQFVSQMKECGSEMGFSPEQVMEMMAKNDGQVGCLRACVLEKLGALQNGNLDKNVLASLLEQNKDTIPNYEQIRANLDTCYGEVTSGGLTDQCQIGGKF  STCMQEHMTG |
|  | CcOBP21 | AAIVFTLCSVGAFAGLIEGDVSPPKPNITKECLKEYGIDIEQTNGAPLSDEEIYCIPACAYKDYGIMRPDGTIDSDKAESYFGVNDHEERSIFFSVYEVCREGKTHCKLVQCMFDNLKNHWKSS |
|  | CcOBP22 | LPVCLIFSVFCFSSAHALLTKEAIESLRTHQKYCVRTSGVSEDHVEMARLDRQIHEDEYQEKFAVCMLNKFNIMNTDGSINKDEISYVLLTDNPWSYQTAKDCTALVGSNVRETARKITNCLLQTDIIV |
|  | CcOBP23 | CFAIVLALCVVGAYAATLSDEQKAKLKGFKEACITESGVNADLVNSIIKGGEIKRDKNLDCFSACMLKKIGIMRDDGTIDVETTRAKARTTSVDVAKADKIIDKCKELVGKDACETGGNVFGCFILGKDFP |
|  | CcOBP24 | CIFVILVIAGCVYGDLSEDHREARKQRLDKCRKEMGITEENPLSRPPNLDDPKEKCFYACLMKESGKLVDGKMVAEKVLSAEKKRRPNYNDDIEAKLTYCVETANEQSDECEMAATMKKC  TFEKLGPL |
|  | CcOBP25 | MKLLLLTCSYLIICMKMSTSAPNHERVDDVDLNDSIDQCAAQIGLLIEETRRSFTMPIEAPGNCVMACVWDKIGLMDIDGKIIKEEMITSIHPTLELLPNITRVTEDDFYQCVDEANRFGDPCTVISEYFKCLIKDLF |
