# Supplementary material for: Transcriptome and Expression Patterns of Chemosensory Genes in Antennae of the Parasitoid Wasp Chouioia cunea
Source: PLoS One. 2016 Feb 3;11(2):e0148159. doi: 10.1371/journal.pone.0148159 (PMC4739689; doi:10.1371/journal.pone.0148159)
Supplement: S9 Table — (DOCX) [file pone.0148159.s014.docx]

S9 Table. The protein names and sequences of ORs that were used in phylogentic tree analysis.

| **GI Number** | **Name** | **Protein Sequence** |
| --- | --- | --- |
| 645012607 | NvOR1 | MMKMKQQGLVADLLPNIRVMQGVGHFMFNYYSEGKKFPHRIYCIVTLLMLLMQYGMMAVNLMMESDDVDDLTANTITMLFFLHPIVKMIYFPVRSKIFYKTLAIWNNPNSHPLFAESNARFHALAITKMRRLLFCVAGATIFSVISWTGITFVDESVKRIVDPETNETTIIPIPRLMIRTFYPFNAMSGAGHVFALIYQFYYLIISMAISNSLDVLFCSWLLFACEQLQHLKAIMKPLMELSATLDTVVPNSGELFKAGSADHLRDSQGVQPSGNGDNVLDVDLRGIYSNRQDFTATFRPTAGTTFNGGVGPNGLTKKQEMLVRSAIKYWVERHKHVVRLVTSVGDAYGVALLLHMLTTTITLTLLAYQATKVNGVNVYAATVIGYLLYTLGQVFLFCIFGNRLIEESSSVMEAAYSCHWYDGSEEAKTFVQIVCQQCQKAMSISGAKFFTVSLDLFASVLGAVVTYFMVLVQLK |
| 299523100 | NvOR2 | MTSKVSPKLEKAPLAYVNEQYLADTEYVVRVAKTLLMPIGIWPRYGDNSTLSNAIIYIRVCLIFCLMLFLLTPHFIWTWFKAEDLRKLMKIIAAQVFSSLAVLKFWTLILNKQDIRYCLEIMENDYRVVESEEDRQIMLKNAKIGRFFTTAYLGLSYGGALPYHIIMPLLQPRVLRSDNTTMIPLPYPSEYVFFIVEDSPLYEIVFVTQILISSIILSTNTGVYSLIACVVMHCCCLFEVTSNRAEKLLRGMKYDKSKISPELGKKLSELIDFHVKAIQYAETMENALNIVMLSEMGGCTIIICFLEYGILQDLEDREYLGMVTYIMLMTSIFVNVFILSYVGDKVKEQSEAIGFSAYSMQWVDLPNEFIMKDLKFVMARANQPTRLTAGKLFDLSLQGFCDVAKTSMAYLNFLRTLEIT |
| 299523104 | NvOR3 | MAEMKRMEDVFAYYDERMKKPGPSCSNEKFEEDVKYATALNRRIANAIGIWPIFTSTGARLGFDICVKTLKNAAVYILLSFLLVPGILHIVVEEGKLKAKILKTGPMILNTMALLKYSVMLFRKSQIQECLKQLESDWRKAGNDELRALMRRNTAVGHRLSRVCVATFYVGGIFYRLIKTLLTPIRYTKDGLMIKPLPSPLYKGLFRFNTSASPVYETIFATQMMSGFVVHSTTVTTCSYAVLLATHACGQLDIVVYLLKRLIEDDGDNGRLTRVGNEAVDRKLRVIVQLHLKVLRFISSVEDLMNQICLVEILGGSTILCLTSFYFIVDLQSNDALGLFTYMVMITSLIALLFTYCYVGEIVSDKAKKVGAKTYMINWYDLPPKKGLCIGLIISVAHSPVQLTAGKMLELSMYNFGCIMKSTAGYLNLLRTITD |
| 299523107 | NvOR5 | MLGEKSHYAVQLNRLFLTPIGVWPIGRDAPLVQRLLKRLAIIGCYLLMSYLLVPTALHTFLEEPDPAIKLKLIGPMSFHLMAIGKYVSLVGRTEEISACFEHVEEDWKMYSDKNAKPELEMMKRNAKIGRFLIYLCAAFMYGGGFFYHMIMPLSVGRLVTKQVRAERYLQLVAENASTDNIDVEPVRVLSYPIYGLLAKLDYVTLLLVQFVAGFVLYTITIASCSLAAVFANHVCGQLEIVMSLLRDFVHDNEDNPRIYALADDAATVERSRSDKFAEIVQRHLRALNFASRVEKNLNAICFVEFIGCTLNICFLEYYFITEWENQNTVSTMTYCILLISFIFNIFIFCYIGELLTEQSKKIGEVTYAINWYTLSGKRAVDLIMIIMIASCYPARITAGKMVNLSLGSFCNVIKTSATYLNLLRTMML |
| 299523110 | NvOR6 | METQSTKRIENDRGFNYAVKLTRLLMISCGIWPAKFSTSFQKCLRPILIIICFFIMFFQLIPFCLFMFLIIKDMRIRLKLLGPLGFSLTSLFKYVVVVIKNREIAKCVQIMVDDWHQLNSTEDRKAMLINAKTGRVLTMVCMFLMYGGGMPYVTIVPLTKGVTMVGNVSYRHLAYPSYYIFFNPHVRPIYDVIFATHCICGFTRYTITCAVYSIVIICVMHICSRIAITSSMLQRLADDSDGRLLGTAVKHHLDILKFATKLENIFKEIFLAEVLGSTYQICLLGYYFITEYEQRAGIATATYLFLFMSFVFNIFILCYIGQILTEQCESIATTAYTSKWYQLSGREARSIILIVHWNRRRVVLTAGKMLTLSLESFSSIVKAAGGYLNILRTAVANSN |
| 299523113 | NvOR7 | MIESVYKLKMNEDNKQNLERNQGFDYAVQLTRLLLMPCGIWPAKFSSRVQRFLRPFLIVACCFVMLFLLVPVCLFMFLIVRDVRIRIKLLGPLGFSLMSLFKYAVVIIRSREIEKCIQNMLDDWQQVASDEDRDTMFENARTGRVLTMVCMFLMYGGGMPYVTIVPLAKGATMVGNVSYRALAYPSYFIFFNPYIRPVYDVVFLTQCLCGFTRYTITCGVYSVVIICVMHICSRITVTSSMLQRLADNYDNKLMGTVVKHHLKFLNFAAKLDNIFREIFLVEVMGSTGVICLLGYYFITEYEQRESIATITYFFLLMSFVFNIFILCYIGQVLTEKCESIAKAAYTTKWYQLTGKEARSIVFIVSCNHRPVELTAGKLLKLSLNSFSSIIKAAAGYLNILRTAIVNSS |
| 299523116 | NvOR8 | MYSAMDNHDIKDLINAEDFEYAIQILRWLFQPMGIWPLKSAAYPSFLRPISIVISFWSAAFLIIPGILSVIRVQNDFALRLRLIGPVSFCLVTSFKYFSFLVKNRQFYAYLINVALDWREMKKNNHNRIIMLRKTQISRFFMTSCSICMYLSGMSYNILLPLTKAPTQVGNVTFKNLPYIGYYIFFDQYADPYYYVVFVMQCMSSFFCYSTCCGVCCISIQSVLHISGRCDITSIMIKNLNGNCNEKALKAVVEFQLQSLKFAREIEKLLNQMFLVEFVGSTFNICLLVYYFMGDFKENDTVGTMTYVLLFISFTFNIFIFCYLGEHLTEQCASAGAAVYTMDWFRFSAKKSRDLFLIVLFCQRPVVITAGKMVNFSLLSFASLMKASAAYLNMLYKMG |
| 299523119 | NvOR9 | MVKICPDMNSEDVSAIKNAQGYAYAVQLTRWLLLPLGLWPTKSIIYQKILRPVAILLCLFIMLFVIIPLCLFIFLVVKDLGIRLKLIGPLGFGLMSLFKYVVVIVKQRDVASCFLGMAVDWQELSSLSDRKVMLRNAKTGRLLTIICVIFMIFGGMPYITVLPLTKGPIMRGNVSLRPLAYPSYFVFFNPQIRPIWDYVFVTHCMCGLVRYSVTCGVYSIAILCIMHICSQITITSSMLDRLVENFDNMLLGKIVTQHLRFLKFASKLEDLFNQICLVEVLGSTCIICFLGYYLITEYEQREPIATVTYFLLLCSFVFNIFILCYIGEILTEQCESIGTTAYMIRWYHLSGKEARNVVLIIASTQRPVVMTAGKMVNLSLQSFTNVIKASASYLNMLRTVTANAN |
| 299523212 | NvOR10 | MSKQNLISNLIELKSIRNAQDFEYAVQITRWLLQPLGIWPMKSSTFFSSILRSLSIATCTFLLGFLLVPCCLHMFLIEKDLGVRLKMIGPLSFCLMNIFKYYAVLIKDGQISSCIVDMAGDWHRLEGSEQRGYMLENAKTARVFTTICALFMYGGGLPYSTILPLTRDAIIVGNDSYRHLAYPSYFIFFNPHIRPIYDLVFFAHCLCGFVMYSVTCGVCSIAILCIMHICSQCSITSATLRSLTSDVDEKTFGKIVTQHLRSLKFASKLEKILNDMCLVELIGCTFNICMLGYYFITEFEQSETVGTITYSLLLISLTFNIFIFCYIGDLLTEQCENIGEVAYMINWYQFSGKDARNIILIVASTQRPVVLTAGKMVTLSIRSFCNVIKASVTYLNMLRTLTASES |
| 299523215 | NvOR12 | MADKKGYEEAIEATRAVLRAFGVWPNRHKISENWLSRSHFLAPAFLIICFINIPQTLKIIKVWRNLNEVLDILVTANIPSFVALIKLLCVRYNKKVIGLLLVSMENDWKSLKTLVETRIMWKNGKLGSLITLVIYTLTCGSYVAYVIMITYINVGGSKQEDVITLNESKKLRPLYMRSYFIYDVQKTPVYEIIWIFQFVSMGVATFTFMAVDSLFAVLMMHLCGQLINLQERLKNFTNMLGQTKTRNFSYQLSTIVSRHEQLNRFAKAIENAFNTMFLVQMLLSGMVLCLQGYQIVIILTGRDTVQIIELLFMVYYTLCFAFSLFVYCYIAEILRIESMEIGNAAYHCDWYDLSAFERRLFILTIIRSKTPFEITAGKFAAFSLEFYCSILKTSGGYLSVLLAVQDRLAA |
| 299523217 | NvOR13 | MTVEIAEDSMERIVALSDDQNVDGYNHAIGPCRFFLRLLGTWPDPYGNVDSWTTSARCLVITATMFLFATISQTVKMALSYKDLNLVTEILTNCNIPTTIATIKIASIWYYRWVLRDLVRQIIEDWEMSHDRHESAIMWRSAKISRIFSIGCMFMTEGTLLTQCVVGLFRPISYAFKTDLNQSIEWPLYMKGSFPYDVQSSPNYELSILGQLLSNVFASTSFSSADSFFIVLMFHLIGQLSILKLTILDLPSKIENSDDRSKFIDRFAFVHMRHNRLWRFSMAIEESFNTMFLIQMIPCIFALCTQGYQLIMIMDADNVSLMELIFMIYFLVLFLFTIFTYCYVTEILRCKSLELSYAVYDCDWTILPAKEARILLLILVRTQHPFEITAGKFASFSLPFYCRILKTSAGYLSMLLAVKKRSEQVASKVVL |
| 299523221 | NvOR14 | MSKKSGFDVAVGPSRAFLCFVGVWPNPEGSETTFETIQCIIVTLTMIIFANIAQTVKVFMVWGNLNSVIEILTTADMPIFVALMKFLVAWYNRKVLKGLVILMMEDWSRSYSSSNLDSMWRTARFSRKLSAVCIGLAQGTITAQFIMVVVFDVNNKGEAERTLYMISYFPYDTQVSPNYEITWLGQCFSNIFAAGAFSAVDAFFAVLVLHLCCQLSILRKELVMLADHHKKQGDNSEEFSRKLARIVEKHEYFNSFAKTIEDSFNTMFLSQMIASSLALCLQGYQLVMIITNTEGKLPVFQLIHMIYFTCCFSFSLFVYCYVAEELRFESTELDYAAYDSDWYNLPPKDTKLLLLLMHRSRKPLEITAGKFCAFSLRLYCSILKTSGGYLSMLLAVKDRLVVEAD |
| 299523226 | NvOR15 | MAEKEQGFQTAFSVTRFVMRFQGIWPGVDKPRTGFSRFQFIPAALMMVFFINAVQTMELTRVGGDLNMIIDILTFADIPIFIALVKHVGIAYNNKVLYKLLYLISEDWKEVTKESEKKVMWQKARLSRIFTMIEVSLGLGRLFIHTIRMTYAMLHPTSFDPTGKLIRPSYMRGYFIYDSQSTPIYEITWGCQFVATAFGGCAFASADALFVALVFHLCGQLTNLQTEFREVGKNTSGKKLEFVRSLARIIKKHRRICHMADTVEYCFNKIYLVQVSSSSVIFCLHGYSLVTILFDQDDVVVELIVMTFFTLGFIYSMFVYCYVAECLSTESLALSSAIFDNTWYDLPPKHAKLLLLPLQRTGKPLIVTAGKFVVFSLNLFSNIIKTSAGYLSMLLALREKL |
| 299523229 | NvOR16 | MDDKEGFEVAVKASRTILRVLGIWPNHHERTESWLSRSYFIMPTFILVYFTSFPQTMEIIKVWGDLNSVLELLTTFDIPNLISLIKILSVWYNKKVLGLLIMAMENDWKSLKTVFELRVMWKNVKLGRLITLAIYLLTYSTVATYVVMAVYITANAYKQEFILTPDNSTKLRPQYMRAHFAYDVQKSPVYEIVWIFQCIAMHLAGLSFMAIDSLFSILVLHLCGQLINLQERLKNVTENLTKRHNLSYQLSRIVMRHEQLDRFAKAIENAFNTMFLAQILLSGVVLCLQGYQIVIILTSRDTVQVTELLFMIYFILCIAFSLFIYCYIAEILRTESTEIGNAAYECNWYDLPACETRLFILTMIRSKTPFEITAGKFTAFSLQLYCSILKTSGGYLSMLLAVKERLAL |
| 299523231 | NvOR17 | MSDKEGFDVAIQTSRTILRFLGVWPDPKRKESWIYSGHFLIPAIVMFYFVNIPQTMMVTKVWGDLNAVLEVLTTSDIPIGIALFKMLGIWYNRDVLGQLVVSMSEDWKSVKSPEERDVMWRNARLSRLLSVTIIGLAEGTIVAQFAMVIYFNVLEARQYSLTKDNVTARFRPLYMSAQFFYDAQKSPNYEIHWLFQCSSTIFAASAFSSVDAFFAVLMLHLCGQLNNLREKLKKLPKQISDKGGGSFVEKLSEIVTRHDHLDRFGNAIEDAFNVMFLVQMVASSMVLCLQGYQLVMITTAGDGIPLFELIFMIYFTCCFTFSLFVYCYVAEVLRTESMEVGNAAYESNWYDLPSCETKLLMLVIIRAKKPFKITAGKFAAFSLGLYCSILRSSGGYLSMLLAMKDRLAS |
| 299523236 | NvOR19 | MTTKEEGFDVAIGITRFVMRTHGIWPGFSVSKAGIMRYAYLPAALMLLLFVIIPQTVQVIFVSRDLNAVLNVLTLGNVPVGIALAKLLGVSYKQNVLHQLILSVCEDWKHTTKESELVVMRLNARKSRMFSIICIVLSEGTAMAYSARMFYAAFSTHTKAQATGIDDCEKPLFFIGKFPFDPQSYPNYQITWTLQIIATFLAAGAFSSVDALFVTLVLHLCGQLTNLQAAFSEIGEENAEKGTMFVSKLSKLIERHRKINVFADIIEYSFNMMFLVQVLSSTLLLCLQGYLFMIILSGQDGLLVEMIFISYFTICFTFSIFVYCYVAELLQEKSLQLGYAIFYSKWYNLPAKKARLLIISIVRCKRPLEISAGKFCIFSLNLFCNIVRTSAGYMSVLLAVKDKIT |
| 299523240 | NvOR20 | MARKEEGFDVAVGFSRFFMRLHGIWPGDTSSKFTWARFAFVPPAVIILMFINIPQTVQIFFVGGDLNAILDILTLANVPLGIALAKILGVSYNHNILRQLIVSVSGDWKHTTKKSELQVMWRNARISRTFSILFIGLAEVTVLANTARMFYILYSTRSEAESSGIKNYKKPLYYTGKFPYDAQSSPNFEITWVMQILATILAAGSFMAVDALFVTLVLHLCAQLTNLQTAFRKIGEDKHEKEVDFMSKLSKLMKRHRKINEFADIIEYSFNMMFLFQVMSSTFLLCLQGYLFVILISSQKVILVELIFMVYFIICSSCSIFVYCYVAEILREESLQLGNAIFYSKWYNLPANKARLLIIAILRVQKPLELSAGKFCIFSLNLFCNIVKTSAGYISVLLAVRDKIVQP |
| 299523238 | NvOR21 | MKIRKSGYDECVGFTRLIMTIIGTWPGAEYSQHWYARYMFSIPLFFSMFFMIIPQTRMLLHVKDDLNYIIEILTTADVMIIVACLKLIGVWYNKKDLRYLLNEIEKDWTITEKEEQHVGNAMWENVKLGKFIMNGYAVLTYGTVVLYAAGMLLLMNSQKIEDFDNENITQSRLMFVRSKFPFETQGSPTFEIIWFLQFLAAVMSIAAFTTFDGFFIFSILHVCAQLVNLQCNFRNLISRCRLTKRTFVQHMRDLVERHIHLQRFTQIIENNFNKVFLMQMIGYSVTLCLQGYQLVISLTENSEQNFITIAFILVYTTANILSLFVYCYVAEKLRKESTEIFYAVCAMPWHEVKPEESKMIVNIMYAAKHPFEITAGKFAVLSFSYFVKVLKTAMGYLSMLLAMKSSHKM |
| 299523244 | NvOR22 | MMANNNKLGFDESVGVTRWTMNVIGLWTLDERRDLQTRFRSLLPAFLILFFIVIPQTRKATLAHDDLNLMLEILTTADIIEGICLLKIFGLWYNKADLKKLVIQISEDWTHTNNDEQGIMWSNARLSKFVCLFCISSSSGSVLTHAIVFLVTNVGANETRSLFLISQFPFNTQHSPVYEIVCFCQFAGALLSTFIFSSFDGFFVFSILHFSSQLSNLNIRIRSLTEKTSGDKCQFVESLKSVVKHHQHLISYTDIIEYNFNKIFLVQIFATSIVLCLQGYQFVMIISESGTKLLTSLIFILVFTTGNVLSLFMYCYIAEIIRNESQRLLRAVYEMKWYTLPAKDSCLLLIVMCRLKMPVEITVGKFAPFSLEYFASVVKTSVGYLSVLLAVRNKIND |
| 299523242 | NvOR23 | MEVKTLVKSDTQISISNNLNGLSGFDHSVKVTRVISRMCGVWPGFEEKKSFTERFFFIVPGMVTFFSITLPQLRRVMIHRKDLSTVLELMTTGIVMELISILKLLAIRYNQSGLRWLLRRMVDDWKIYDKGQYYKIMWVYARHTNTIVTICIALTTGNIAAQIIRQYAIYIIERHYSSANETVIKPTILKSDFYFNEQIEGIYELVVAAQILGGFSVAFSFTAFDGFFVCSIMHVSGQIHKLQMQIEDLVQCYERREGAFSEVLGPIVHRHRDLRGYAAVIEENFNKIFLVQMLVTSVFLCLQGFEFAMVVAEGGTEMVPHLIFIVCFVASNLVSIFTYCFVAEQLRTQSNQLFRSIFQIRWYDLTPKDSRLLIIIMVQTKKPIEITVGKFVPFSLDYFCSVLKTSAGYLSVLLSMKDRL |
| 299523246 | NvOR24 | MEESPGFLHAFGICRTCLTMSGLWSDTHFKKSKKFVISVLYAANVFVILTFMNVAQTVKLFLIWGDFDEMSQIISTSDFSVGMLVVKMFVFRSYRKALALLIEFVEKDWLDLKTISEEETMEQNAHTANKIYLTCFFLGNSAVNSYTLLRLGQEMSFLPGPPDKRQPLFDAYFPYDDKRSPAYEITWLMQYAGIALANLAFTGMYCLFVGLMLHLCGQFANLRIKLIEAVSRKEGESEKKSDGAKTFRERLAFIVERHNSLNKYAQVIEKIYHWIFFVEILSSTIQMCSQWFMLVTVISNTQGGLPYLQIGFLLIFTAHSGFHLFACCYAAERLQNESLSIFEAAYSCEWYNLSPQDAKMLLFIMQRTKTPLRVTAGKLCVFGLELFAKILKTAGGYLSILLAMRDRLVIDEEPI |
| 299523248 | NvOR25 | MDGKRGFDHAFSLCRINLGTVGLWPNSKNGKGHQEVASLIFFIISLFTIIVFVNLAQTVKLIMIWGDLNHMIDNISTANLPIAVVVFKMLTFRRYKKTLTRLLGIAMDDWCTKKTSREAENMSKNARTARKMSLVCVVLGFGSVNGQLAVRISQELDILPGQTEKRLPMLSSYIPYEYQTSPAYEITWFMQYLGAVLATLVYSGVYCVFVGLVLHLRGQVANLRFMFESVDDPEEDKGKNFRRRLRSLVERHESLNRFAEDIENIFTLMFLAEILSCTIQICLQVFLLVTLMSNDNGGVPILQILFMMVYAMHVGTHVFICCYVADKLRDESLSICDLAYNYEWYRLPARDARLLLFIMLRAERPLEVTAGKFCAFSLRLYAQILKTSGGYLSMLLAVKDRSTNF |
| 299523251 | NvOR26 | MDKKRGFDHTFGMCSINLGIVGLWPNSKNTKFQEFRSNVSFVFAIFSVSVFISMSQTAKLIMIWGDLYQMIENISTANLPITVTVFKMLIFRSHKKVLGELLALAIGDWCTKKTEEETANMCANARLAHRISMICVFLAGGTVSIHAVLRTCQELDIMPGPPEKRLPLFSSSYVPYDYKSSPIYQVTWLMQLTGTSCATLVFSGVYCAFVGMVLHLRGQVANLRLKLENICEIREKGEGLVEARRDFRKKLGFIVERHLVLNRFAADLETVFTLMFLAELLSCTIQICLQVFLLVTLLSNIKHGFPILELFFLMVYIMHVGTHVFICCFVADKLREESLLICNSVYNYQWYKLSAQDAKMLIFVMHRGDRPLAMTAGKFCAFSLQLYAQILKTSGGYLSMLLALKDQS |
| 299523255 | NvOR27 | METKAVAMTDSRAQVSNYFPDSSGFHKSINITRTISRVCGIWPELEEKKSIAARYYFIVPTIVIFFTMTVPQVRRAVLHRKDLSAVLELMTTGIVMELIALLKLLGIRLNESGLRWLLRRMIDDWKTSNSKERNIMQEYSNLTRFIMTLCITLTIGNVVAQTTKQFAIYFMERYQSMANETVIKPTFLKSDFYFNEQPEGIYEAVVAAQILGGFYVAFAFTACDGFFVFSILHVSGQICNLQLQIEGLVQNHEQRRCSFIKVLAPIVVRHRDLRGYAAVIEENFNKIFLVQMIATSIFLCLQGFEFAMVITKSGSEMVPYLMFILCFVASNLVSIFTYCYVAERLREQSENLFRAIFEIRWYDLAPNDSKLLIIIMTQTKTPIEITVGKFVAFSLGYFCSVLKTSAGYLSMLLAVQDRL |
| 299523261 | NvOR28 | MDGEKGFLYAFGMCKKSLTLIGLWPKSKSSNYAEAVVVLRFTLTTLLIVSFVNIVQTIKLLAVWGDLDAMTDIISTANLPIAVAVFKMMVFYKHRKAFEPLLSFVEADWKSYKTDSDMTNMWSNAQTTRRISMICVILGAGTVNGHLFIRLGQEAKILPGKDGATRLSFVDSYFPYDYSPTPIYEITWAIQYIGAALATCAYSGIYCLFVALMLHLCGQFSNLRKKLRRVVTNEDDKRKFVEKLAEIVKRHENLNNFARVIEKIFNLMFLAEILGCTIQFCMQGFFLLTLSSKEGMGLPILHILFMVIYVLHIGTHLFICCYVSEKLQDESVSIVRAAYNCEWYNLSAKDAMLLVMIMNRAKKPLRITAGKFCAFSLSLYAQIFKTSGGYLSMLLAVRDRIT |
| 299523259 | NvOR29 | MDDKKGTSFIHAFGLCRINLTVLGIWPTLRSSKRDETAALFRLVLSLTIIILFINTVQTIKLFIMWGDLDAMTDIISTANLPIGLMVFKTFVFLYHKEALVPLLSFVQTDWSNFKTVSEAANMWSNALAARKISLLCVVIGWVTVNCHLAIRIGQELRFMSGKNGLTRLPFFDSYFPYDYTPSPVYEITFVIQYIATMLATFGYSGLYSLFVALMLHLCGQFANLRDRLYTVTQKKAGVTFQQRLGYIVMRHQCLYNFAQVVEKMFNLMFLAEILGCTIQFCMQGFFLLTLSSKEGMGLPILHIMFMVVYVAHIGTHLFICCYVAEKLQDESVSIAKAAYECQWYHLSPKDVMLLIMIINRAKDPIEMTAGKFCTFSLSLYAQIFKNSGGYLSMLLAMRDKIT |
| 299507620 | NvOR31 | MDDKDGFEYAFGVCRKELIIFGMWPKPNDTMDHKVFAIFRLVLCIALNFIFINLVQTIQLFIMWGDLFAMTDIISKASLPIGLVLFKTLVFIYYREALLPLLAYASSDWKKPKSSLEAANMWSNARTARQLSITCLFIGLSAVNYHMAVRICQELRIIPGKTKVERELYFNAYFPYNYTESPAYELTFAMQYFATVLATFSYSGLYGLFVGLMLHLCGQFANLRVKMDKVAKQADSAKFRQNLTAIIIRHQFLFRFSQIIEKIFNVIFLGEILGCTIQFCLQGFFLCTLSTEDVGLLVMYIFFMVFFIGHIGSHLFICCYVSERLQDESVSIANAAYKCQWYHLPAKDVMLLVMVINRAKDPIQITAGKFCVFSLSLLAQIFKTSGGYLSMLLAVRDKIT |
| 299523264 | NvOR35 | MSDKIEDAQKKLETREQRLRDFKWALGLNRLSLRLMGVWPGDDEAEGLGRLAILLRVPFMIAAMFFCLFLPQMGALALVIHELPLVIDNLMTSCAAFTCCIKLYFVWRSKQVLRPVIQSVSADWLRPKLDWEREAMIREASRARIFTVSGYAVLAGCYTGFAFAPLFGFDIRMISNITDYGEKHLLVQSYFPYDYSKSPNYEITQVSQLIAGFFIGMSVSVPDNYFGALLFHASAQFEILGANLENLVRQDDKALRSRQFNRRFGIFVDRHVHLMTMVTAVEYSFSFVIMAQIFCMSIMVCSLGFQILGMIEGTTADKPSLLQVLTLLGTLFTLMMHTLVDCFACETLELRSAGIFENVYNSRWYTVPKQSVAKDVIPMMVVSKNPRKLTAGKIFTLSLATYCSILKSTAMLIAVNRR |
| 299523266 | NvOR36 | MERSQKNQLQDFDWALGLNRFSLRLMGIWPADQDESSKSLLTVSRIPLMILVLLCGLFLPQMWALALVIEQLPLAIDNLMTSCPAFTSCIKLFFIWRSKTILQPVIDSALQDYLRPKSKSEETAMQREALRGRLVTIADYSIMASCYVGFIFMPMLGFNVRIINNLTDCDTQRVLLVQSYFPYDYARSPAFELTHLLQLAASFFVGMAISIPDDYFCALLFHASAQFEILGLQIESLPIDGSKSGRLLSGFIERHVHLNRMVSAVERSFEFVIAAQIFCMSIMVCCLGFQVLRMLDSAAEKPTPVQILTLGGTLFTMLLHTFVDCFASENLAARSSELFFKIYSSRWYSLSWSKMRCLVPMMLVAKTPRQIRAGKILSMSLATYCSIIKSTAGYISMLIAVSGR |
| 299523269 | NvOR37 | MESLEKYRSQEFDWALGINRVSLRLLGIWPADQDESSKSLLTVSRIPLMVLVIFGGLFLPQMWALALIIEQLPLAIDNLMTSCPAFTSCIKLFFIWRSKTILQPVIESVLQDYLRPKSEWEELTMRREASKGRLITIADYSLMTICCVGFIILPTLGFHVRIVNNVTDYASYGNRALLVQSYYPYDYYESPAFELTNLVQLTAAFFVGMTVAIPDDYFCALMFHVSGQFEILGLQIENLMGKDDAKEGVDWSLLGSFVERHVHLNRMVATLEKSFEFLIAAQILLVTVMVCCMGVQVLRTLNGAGEKPSPFQILTLSGTVFYLLLHTFVDCFVSESLTSRSSEIFFKIYSCRWCALPWNKVRCLLPMMLAAKTPRQIRAGRIMPLSLATYCSIVKSTTGYISMLVAVSGR |
| 299528647 | NvOR38 | MKSNNAHESFFAYLNWAIGLNRLSLRLMGIWPDDSVAETKLFTTILRIPLIISVMMLCIVVPQMYALILVRNNLLLIIDNFMTSFPTLIGCAKFYFLWRSKEVLRPVVCSVTEDWLRPKSDLECQKMRDAAVVARLFTVGGYSLITGSLMGFIIAPLCGLNIRVEQNITDYGRQPLLVQSYYPYDYSQSPNFEITHSSQIVAACFVAMSLAVPDNYFGALVFHISGQFQLLGLNFEHFIKQNEKIVGIMAVRDFNKSLGVYVDRHVHLIRMVAIVEKSFNFIILIQIFCLCVMACCLGVRILSAIGNPNDKTAVIQIINLGATLISLMIFAFSNCYASETLASRSAEIFQQVYSSDWYKIPKRSTCCYLIMIMIMSKNPQMLSAGKILYLSLSTFCIILKSIAGYLSVLIAQSN |
| 645002394 | NvOR41 | MHRMRTRVKRLKVSRSSKTKGGFSYEFRIYKIITWPAGLWPLERDNIFNVLRFLLAASSQMFIVVAALVEIYRKCGNVADVLDYYALSIAFWLSFVRLVLVRIHLAKIHKICYNARKTWARIKDPDLVKIMISHAKTGKRFYYLQMSIAFVIVTLYVFNPLILRRYDAANLPMQTVCTFNNADVLKHTAVYFIETLSFVYLAVGFISIDLLFLGIAMHLCGQLKILQKEFSEIVGKSTSQADCIRYVISLSRRFQRVVELTDDIRKTFSEILLVNFVVNLFLITSQSVTLLLALKINNYFLAVKCSQTFPILLIEMFLYCYVGELLRHAFDDIPRAIYSSRWYLLPPKIRRGYLLHVMAQASKTFDLTAGKMIRMNMCTFIQLVRSIVSFFSLLLLMFDK |
| 299523271 | NvOR43 | MKCFDSYDKSNFKTDPQHAINFFKKLGRFWTIWPVSANASRFTKVYHECSLWFIIINLFFASLTLWMSVCVYHKYPILMAKNLSQLMIISDSFTHLVLYRINRSELQVLVQEVFDFMKNSKQNEKYIMRKHYNRFLGHYTILIVLYVIASLAFFCGAFILGKKFPMDASYPFSTDSILVSSIIFTHQTFSIVQNSVLIMIDLLVITLFWYAGARIKILGYKFKIVDSNEKLKNCIKEHQKIIQYVASIVKAVRFILYKTISIVAIIIISAGLQLLYYDAKVVISQFSLIIIVACFRIITYSSTIEEMNQLNEDLRWTVYKSSWFCITSEMKQCLQIFIHRCQIPLVTIDGQLLNIMSLAFFAKLIYSTVSHLTTLRAIIERS |
| 299523273 | NvOR44 | MAFKISPEKAFTFTKLSVFFTAIWPPNCNDSSFKIKLANIFWIYSIISAMCLLIPMLASVLVYKDNPMIVSKSICLSCAVIQVIAKAIVCRHHQKKLQFLVKELTHFLKKAKKEERQLIEKYINRRAIFHMTFTLCCFGSSFFVICGPIFLPFSLPADAVYPFSVNSSPIWEIIYVHQASVGIQASSGMCVDNLVAYILWYTGVRFESLYYKFKHIKDSKEMLQCIKEHQYLLRYGTTVADTFRYVIFTTVLSVTAGLAFAGIYLFSPQPIFVKGQFVVVSISVVVNLYVTALPSNNLISMCHKVGDVVYESLWVGDSPSIMKHWIFIIQRCQKPVVIAIPGLIKELSLQFYSSVLCSTFSYFSALHVIMTKE |
| 299523275 | NvOR45 | MKFLIRPKFFFKTLRLIGDMIAIWPKHIGAKKTMIMFHEVKWWLSFTNATGLLIPLVLGVYYFRNDSITMTKTLSELTALCEVFINLIQCRLQEKKFQVILYEIENFIENSNEQEESLLQDYLNRYKTLQLFVGSSFISTAILFSCIPIFTSQLLPADAWYPFSVEYFPIRVFLYITQVLAIFQTGFGICVDLTVATMLWYSAVQIELLEKNVHKAVSKAELRECARRHQEIIEFTDNIKKGIKFIILKTNATMIIVVICGAFQLIHHEPLEVLLRFTLMVLAGCLRLYVSAKPADDLKENSEQLARTAFQTALMQKSTSNSKIGLMLAFRCQKPIVLSVTAVIRAYTLQYYASFLSRTVTYFVNLRAVLDD |
| 299523277 | NvOR46 | MRKMKCTFTFSIISVYETLKYFGLLTSIWPIFSKNRYLWVFMKAVYYFILVNYLCVFIPLILMTLFNINTSATVTITAVEQMIIVEAVYNLLYTRFYSAQFKSAVKEIEEFFKNSSPKERYILDRYATTRTFFNIYIAINYFVAIMSFNFGQLFLKDRPYPLNLWYPFTIKSQVIVVIIYIHQVIVITHTLILIVFDLIVQIFLWTLAARFELLQADFKKTASEMDLKCNIQKHQYLIRTTEAVIDFTKYMILKVFLAVTILVISSTLQILHRGPSTIIVQFFFIMKIASMRAFAYCWAGHSLAEKTGGLARSIYNSYWINQTQRMKTNVLIVMQRCQKPTVVKISGIISSLSFRFCVNYFYMIYSAFMTLRAVLEV |
| 299523279 | NvOR47 | MSSWRMGSIKDIMMNVFMMKVIGVALAIWPLKSAGKRWYYAFLQEMVYRFFHVNFWLLVIPSLWSIYKKRHNLASVLTSVTQLTIVFEILAIMVLSRRQAARLKTLLTMAYDYVSVADDKVYPVVYKYVRKAQIIFGIITIAYALILLTYLVQAFIENKPPIYAYYPFDIKSPVVWICVYGNQLLCTFYAAVVIIMDAMMMFMIFVTSIRLELLQNDFKKVKDYPDLVKCIRTHQDIIWYIKEVYCIKKYMVLKMLISIAIYIMCEGLQLFALNLSWGMRFQVSLLFGIGLFRVYIYAACSQDLISSGLDLGYFVYSSLWYNQSHSVMVAKAFVICRCQKSLGIRVCGITDDLNMKFLANFLYRVFSYTMTLRAIIKTLR |
| 299522708 | NvOR48 | MMINKKVTDKLVTIVSTLLNIFLHIDYLFHSFKILKFFSRLFALYPLNSDCTKLEILYDNFVWLFIHFHFWVAAAATLVAIYKARSDLSIWLIAFSELIIIIEIIVAMILYRLQRSRLKILMHIFEDFVKDPDDSKIQLIRRNAKEHIKVFSILALLFIIVVIMYVYRALSTRPYQLLLSGYYPCTSDSLIIWLVVFFHQCILVIYSPSTFASDSIVTVLIFAAIIKLQKIRPRFRNIENYAQLVGCINEHQHIIWFVQEINYVIRLFVFKSIFCLAALQLGVGVILFMPNISVFTRIQFLLLFTVTIFRIYIYSYCAEILTKSGLDLGFAVYSSRWYDQRRKMVLAKSIIICRCQKPLLIAINGIIPALGMRYLARFLYLTFSYIITLQAMTRT |
| 299522706 | NvOR50 | MFKKIKPNFTVQKNFNILCNCMKILGTWPVHRRHNKIFTCLNHSLWWFYVVNHMMLLLPTMQTFYNTTKDIISASYSLIEITGIVESMVILITFKLQGSRIQLLLQIIKNQIVVKKPKPALNNRNVHASVFAIIAVLYVIVVYMYIHKPATLINKGFIMTTCYAFPTEDIRTKIAIYCNQLIALMHTSVVLVTDGVAVLFIYTCAIKLKTLEIRLKKAPDWTKLKYDIAEHQTILLVIEETNSLAGVLVVKTVICFMCYSISAGVQIINQHVVTAQMLHQFIIIAIVYLRIYICAETAEMLLTVNGDMLFTVYSIAFSTPNIVKVKSLIIMRCQKVPKIYVNGLMAALNRAYLRSISYATFSYFMTIRAIVSK |
| 299522710 | NvOR51 | MRIHLNAKIAYQYLRLSATLMATWPLSDATKKYRNVFYNMLWWLYLTNHLIILYLTLNTIITHNKNHLTVFYTWLEISFMTENIIVLVSYKLQETKWKQLLYTSKMTINRTEDNIRLENSDLYPKVFATLFIFFIVIIISYVNKAETYERGLIMTTRYPFEIKSIGLKLFLNLSQFITLLHASSILITDAIVVLLLYTCTIRLKIVEQKFRACKYYRHLKLHIYEHQKTLLLIEDTNLLVSKTVLKSIASFMSYSIGGGLVLYNKNTSPLQLVQICLVICVIYLRVYVCAEIAEKMISANESIGFTIYFTKWYEESAKDINAKNIIIQRCQKLPRIYINGFMQSLNRNYVRMITYATFSYFMTIRKIINKTANCVDC |
| 299522712 | NvOR53 | MQNKFKLRDRTNKFTFMFKSYSGVHLGFLCVNFYMKCLGIYPLPSTVSKFWTRVYNLLWCFYLSNHLLIIFPTFYAFGSTTQDIAVATFSLMEGLCMIECIVLLIHFKYQRSDFKILLSLVHHELNKKKRIITLDNGNVYIIAFVLIAIMYVLIIFNYIQRPETVRYHKLLTTARYPFSTRAATIKIILSCHQIVVLLHMTIILTSDGLAVLLTLICTVRLKNLETKISNEKRGKLPKRI  REHQQILLQVEETNLIVRIIVIKTVFCFMVFSISTGLQIFHKFEIIQIFIVMIVFLRFYVSAESADNMATCANNLGIAVYSTAWYEEKTKIRIAKTIIIQRCQKSPRIFITGFMSELNRKYFLVVAYATYSYFTMIRTLISKNK |
| 299522714 | NvOR56 | MSVYISPNRSFRVLRFLGTHLRIWPDDNKKWNFKTDVFFWFCVINYVLLLLPLFNALYLNRKNVVAASNTWIEVSGYAEVLAAFIYSKYKRVQLYVLLCEAEKYLLFKKVTIIKKYANTYAKIFLLVIVFYLFTVFVYWSIEKPITGYEHLITTAVYPFNIRSHPIKGLIYCNQTFNLVYSSILPVFDGISVLLIFNCTHRLKILEHKFKLAKTSSDLSECVREHDDVSRTIKETNSIVRFLVFKTVCSFTSNVIPGGLQILNNVALSQSICQVCIILLVYSRIVLCAECAGNMTDAGEDLLFTVYSTLWYNEEPKIVSMKIFIIQKCQNIPAIHIKGIMSGLGRKYLLTIMYSTFSYLTTLRTVTSDEKS |
| 299528533 | NvOR58 | MTIQSVLRRKVDVLLKAIALNKMCVSPKMILLVIKFAAMYLAIWPLDSSGKHWNTAFDCLWWFYVVNNVLVIIPTLLAFYSSRRDIIAAMFSWLEILALLEALIILANFRYYRSRMQPILKEAVDYIGSANSRRQLCLEKRASIITTTFGVIVALYIAGIIIYIYRPAVTEWDGMLTTAYYPASMRSPFADVFIYITQLTALLHNGVLIVSDAFTVLLLYVCTVRLEVLQKNILRVADYDELKLWIREHERVLRLVTDTNMVVRINISKTVISFVGYSVGAGLQIISPTVTIVSFQRFALVIAMNAMRLFFSATFADDLVNSSNSLINTIYSTIWYKDNRDMKIGKIIIMLRCQKLLRISVGGIMPVLGKPYLTKILYTSVSYFMTFRAITGN |
| 299522716 | NvOR59 | MSRNLYHDAKIKCKMNVITYLLKCLNFLRFMGKIYAVWPLKTDDNIRWRFVYECLWWFYFLNYLVAASFTLNTCGHASDDITIASFSWLEFVSMVESIIILINYKCYHVTLQLLLTEVEDYLTLADEKKQWVLKEKASIFAVMMCIITFLYFVLVVLYFTNPAITAWETFLTTSYYPPAIRSPVMDVFLFSNQLIVMCHTSVIVNLDAMVVLLIYICSVRLKVLAADLESVNDDEELKQRIREHQHILCLAKKTNIAVRLVVSKTVICFISYTVGAGLQLVNPTATVASLQRFGIVLLINYVRLIMNATSADELLTVSRNVGLSIYSTDWYGESKIVTSSKFIVMLRCQKLVRIHVDGVMPALTLTFITGIISTSISYYTTLRAVTRQN |
| 299522718 | NvOR60 | MSRVLQSDSSSREGKHVWSKDAKFALMLNKFIVWPLGLWPLECDDAFSRFRNFYAVVSQVWMIGTQATAAYLGCGDVADTVDFVMMTACALMALSKIVTIRLHMSKVHTVFVSALDDWLAVDVDKSRDVLIPFAKTGRFVFYLQMVSAYMSNTLIIIGALPFLIPPAANGTWANVSETLQSRQLPMRTGCMFAGYRDEIYGSLYVYESVMIMITAHGNVGCDVLFFILAMHLCGQIELLKTDVLKIGEDEKVPGEWKNKIVECVHRHIRLLGMAKALNKVVSGVLVIQLLLNAGLNLMLGIRMLIEIKRGSIFNAVRPMIGFNVLMLQLYLLSYASDRLSSQAESILDAVYDSYWYKLPAKLRRDLYFVTMRANKPIYFMAGHFYAMNIENFMNILKASFSYFSILRIMFQA |
| 299522720 | NvOR61 | MGGKSEVDEAFAVYRAFLWAIGVWPLEEKSFSQILRYIVAAVVQVTFLLHTFTEILLNNGKVSDMVDVFFFSSAAFLTFAKHTYLHLHKDAIRENLRCYLDDWSNTKDEHFLRIMREHVKIYKYQFHIYNLCGYVGTTLFMCRSILINILAKRQLGPGESYNYQFICQTSYLSQDTLAKYYPIIMAIQYIQCMYCCTSGACTDCFFFGLVFHLCAQFEILKIKWERLGTKDFGVTAVHDRVKVNALIARHKELVKLGENLESGFNNTILVQLMISIVLICMSGCSILVAIMRNDHVTMLISTNSISFMVTETLIYGYASDYLVTQSESIVQAVYSSSWYDMDSSVKKDIVFVMMRAKIPLHITAGKFFCVTRNTIVQLLKTSVSYLSVLRLTLEMSHQEGQL |
| 299522722 | NvOR62 | MCANIFIGHHQFGLRVVGSWPGKSQLPGFYFAIGIMLFFLIFEILNITEVYHDLEELMDNLVSTIGVVLGLFKFITVRVKRRKLKTVINKIFDDWKTDSQFVSEMMVKNCTRSQLVSKFVIFLYNSMNFTYFLRTVISHIFDEVQDRKFLAQVTFPIVDGRQTPLYEIIIFFQFITASVCFNSQALVEGLLATLVLHACSKVDVVRREILNFSTICKTDKNDKKDILKTLRKLSEEHFKFIEFSEDIQDIFSYVSFFHIFFLTLIQVVSGYMFIDGLERGTKPVNLIHYAILTTSFLVSAGYYCIAGEYLTSQSEIIFNELYNCYWYEFPSSYKKAICFMLLKARKPVKLTVGKFSTLSLIYLTSIMKTSFSYLSLVRAVR |
| 299522724 | NvOR64 | MSDKIVMRHVRVALQVIGLWPGYTSSVGFVIAITWLLTCLTFQLWHAAVVFSKLDALMGNLGATMAVATATLKLIAFHVKGRNVKIVIKEILNDWAYENRSSNCEVMVQNTKRAKYLTKWITGAYNATVITYLVNAIIAYCSGITEQRLYVLPSKFPSFCKQSPVFEIVCFFQFSAALISTNVQVLVEGMLTVLVLHAGTKVFLLQKEIQKLSVICQSKTNNKEVISKSTIALINKHLNFIKFVKEVKDIYYFISFVHVFTFTFLHVIVGYMFIDTLERGDRSIKLFLYGLFTTRALASTTIYCIAGEYLMNQSMRIFDELYNSAWYEFDVPNIKAITFMIMKARNATSLTPASFGQLSLFYLTSVIRTSFSILSLTRATR |
| 299522726 | NvOR65 | MSILSRHVKIGLYAIDAWPGVSSSGLFFLVMAYMTFSLIFQILNTTEMITQLDLLMNNLQTTMPVILVVLKLSVFRVKCRSARLIIADMLSDWKCINETKERKVMMKNAKIAFYLSSTIAICYNGLILSYLLKAILAYETENIYDRKYVMQATFPINAKSSPVFEMLCLFQFTVSVFAANGHAILEGLLTTSVLHANTKAFGVCQEITKFAKSCEANKSRKNIVEAKRRLIKRHLYFINFAEKIQETYAYISFFHLFLMTLINCIVGYMFINLTINKDNISALLLCIAYMFTALSAVGSYCIAGEYLMSQGSLIFEKLYDCPWYKFKPVDTKTFIIMLMKSRHSVTITAGNFGDLSLVYFTNIIKTSVSYLSLVRAATN |
| 299522730 | NvOR66 | MIPIFNKPLECCLKVAGFWPYDFNMLGPVAITSMLVTTLPFQCWNAFALTENLVVLMDSLSDIFTEVLIYIKIFILWNHRREIRDLLEEIGKDWSIKSIPTEWENIADYCRIICNIDVIVYASASILYYPDLLMSYFGKPVNERHMLFQSYYPFDYRRSPIYEVINIVYFFQGILMIIADSVSKTLFISMIFHVSSQIYELRNNLEQYSRHSNDGYENKNFKRLKLVVQQHLKILSLVRRIDHIYSYVALFQIVFSSIIICVTGFVIITAMESANIMLLVKFMTFIIAMLAQVSYFCFAGQYLLNKGESIVEMINSSFWYNSQCKDVKVLIFVLTNAQKPLTVSGANIFNLSAETFTMIVKTSASYLSVLRAMYTQ |
| 299522728 | NvOR67 | MILLINKPLEYSLKLSGFWPFEFNIIGSLALISTLVTTLPFQCWQAFNFTNDFVLLMDSLSDILAEVLIFLKLFAMWKSKSCITIILREIFDEWSTEKIPDEWKTLAYYSRMFCNIDTLVYFSAAASYYPDLLMSYFGKPIENRKMLFQSCYPFNYLGSPTYELINLMQMIQAVAMMAADSLSKTLLVALILHVIANIDLLKNEIRIYSTNIANTCNHTNNKKSTVDLKQVISQHRKILYLVQSIDNAYSYVSLFQIVFSTIIICVTGFVIVTAMESANIILLFKFILYIIVMLSQAFTFCIAGQYLRNEGESIIHEIYDCLWYYTEPKEIKSLIFVLKSAQIPLTLGGGKLFELSTNSFTMIVKTSVSYLSVLRAVCV |
| 299522732 | NvOR68 | MKIPIIGIPLEYTLKLAGLWPDQSNILGSIVMGSALVTMIPFQVWDTINVSDNLVMVMDNLSNILSEVLLYTNFIVLLLNKSYLDDLLREIADDYKNNIVTEKWLKLDQNSRRFCNYDYGMYLGACCLFYLQFALMYTQMPSEDRIMLLKAYYPFDYKSSPVFEIMCFIQVIQGLLMCSIQALSESLLIALVSHVSGHIDLMNKQINVVSKSYDGQNSLTLKLVIKSHLKVLNLVNKIESVYTYVSLTQVCLSTFIICVTGFVVLTMNSANEIVVMIKYIMLYFTLLWQSFSFCFAGQHLLNKSDMIPYQVYDALWYKAEATEMKAILFIIKRAQTPLSLSAGKFIALSAQTFTLIIKTSFSYLSVLKASYA |
| 299522734 | NvOR69 | MKIPMVYWPLEYTLRINGLWPGENNILGSIVTASGMVLILPFQVWDAIKTIDNPILLMDSLSDIMTEIALYAKLIIMWFNRRYVVDVLKEISNDCNQNDVSQNWTLLNYNARRFCKYDYSWYISATLLYYIQLVTMYIEVPVDGREMLLKSYYPFDYKSSPTYEIMLFLQIILAMSMAIANAMTESLFIVLILHACSYVDLLLDEIKIFSDNCNKKVLNITDSNNMRFYVHVILKRHIQLLESVKKIENIYSNVSLVQMFFSVITICVTGFVMITALESKDIVLLIKFATFIWFLLWQIFSFCFAGQYLLNKGETITGAMYDSDWYNIESNDVKAISFIIKKTQRPLSVTAGKYIPLSVTSFAAIVKTSFSYLSVLRASYVE |
| 299782496 | NvOR71 | MYDEIFIRPHKISLKLIGAWPGYAKLTGFFLVIGSSSVLLFFALWNTIEVFGNLELLVDNLVNVIGIIVGFFKLTTLRVKRRNLIFMVDTMFEDWQTSKKTIEELNAMKDHFERSKWLCKSIIMLYNSLILTFLLKPVRSYMNDSIEGRQYLAPVSFPKFIDAKQSPIYEIVIIGEIGTAFFCINSHALVEGLLASTVLHASAKIAAVRQEIIRFSKVCRSQNSNKRLIISATRRLVQVHLSCNEFSETIVDIFAVISFFSILLMTLAQVFSGYMFIFNIENGGETVQTLHYGFLTIVFLVSSGYFCIAGEHLANQSELLTMEIYNCFWSEFRIPEQKAIRFILAQSQRPVRLTLGKFDELNLVYLTKIIKTSFS |
| 299528641 | NvOR72 | MDDEIFIRPYQISLKLVGAWPGCAKLSGFFFVTGWSSILLFFALWNTTEVYENLDFLVDNLVNVIAVVVGLLKLTTLRVKRRTLMTILNKMLEDWQTMKMIEEFKAMTDNFERSKWICKSIVMLYNSLILTFLLKPAISYMNDSVEHREYLAPVSFPKFMDAKQSPMYEIITAGEIVTTFLCLNSHALIEGLLASSVLHACSKVDAVRQEIVKFSDVCRTQSGDKMLKLTAIRRLVNVHVNCDEFSENVENIFTVISFFHISLLTLMQVLSGYMFILNLEEGGEILQTLHHGLIIIVILVSCGYYCIAGEYLTNQNELLNVEIYNCFWTEFPVPQQKAIKFILAKSQRPVRLTFGKFDQLNLLCLTKIIKTSFSYLSLVRQVH |
| 283945550 | NvOR76 | MSTKKIASSIDSFLWPNRYTLEFLGFWPPEPGTSSISKYFAAFRIVFSILAIGFLFVPEIMMVVVFWGDITVLTGVGCVSTTLAQLNFKMLYVLARRRRFCRAYRKTRELWSMTDHESELRKGLEKLAGQAKKYSIAFFFTCFCNNISFTTLSVVVWLNYNAQENKSLLERRLPFDVWFGFDLQRTPNFELVFVGQSISAIFCCFGIVGLDTAMMALILHVCGHFRVIGARLRAIGQGMHNDVQSKNSVEYLHTSPKLAIWQCIQYHQQMIKFAEEVRSLLSPIIFVQLLTSGLEICLSGYAVIVNSDAGNYGDLVKCTGYFLSVFIQLIIWCWPGQILIQDSSEIGRIVLHDLPWWDMATEQQRQFVFVIFRTQKECQITALGFQVMSMSKLTDVFNTAGSYLALLRRVYEKETEE |
| 283945552 | NvOR77 | MAGRGSSVRIDEYLWPNRYLLELFGTWPTDYDGRTLASQLFVNFRVCFVFVAITGVLVPEILMIIVYWGDLDVLTGVGCIATPVSLILFKVAYMIIRRNRFHGVYSNLRRLWLAIDDAEEFEPLEELARLAKRVTIGFFLSCFSNNVSFTTAAVIDWVNYDETRNDSTPRHLPFDVWFSFDVERSPNFEIAFGCQVISSLYCCTGIVGIDATMMTFILHICGHFRTIAAKWRAIGSKILDNEKYSKSGQVMPVKKDINQILRQHSEMLRIAEEVRRLLAPIIFMQLLTSGLGICLSVYAVTMNGSKGADLFKFIVFFVSIFVGLIIWCWPGQLLMQDSAALGDVVCYELPWHLLGVAEQRNLAFFIMRAQKECQITALGFQVLSMNKFTEIFNSAGSYFALLRTIHEKQLEAQ |
| 645019199 | NvOR78 | MARSFASFDEYTFLNRWGLTFLGIWKSDAEARGGPLRRFLHRLHVTILFTLLMLLLLPQWMDMYVLWGNIDANAETFVLNVFTITALLKLWCFLSARQIFEVPENARAATEARVYFLKFEIKQVIDTMKENWRRTMSGDEPGRKTHREILLDMAGKARDYTKRYGLLMYSTATMYFVSPFVGMQRDNVRIRKYPFFGWYYFDRFSNLYYGICYASQVIIGIVVGTSNYAMDSIFLVAIYHTCARLQMLQHDLKKIGEDRENRSPEEIVQLIRLHQREIRDAKRLTKIFNGSSLQQLLVSCVIICIIGFKLIIALNDGGFEFLVYVAFMFVALLQIFLYCRPGDELIVQSTAVGYAAYQSHWTSLEAESIRKIMFMILRSQTSLKMTAGNFYVLSLPNFTMILRMSMSFLSLLRAMYRKSDGFG |
| 299522738 | NvOR79 | MRIGARRASRMESTTEASGIMREYDDCIFLNRLGLTMVGIWPLEHNASRLRIVLRRIHLGAIYVLMLSVVIPQWFDIYCLWGNIDANTETFMSNVFMIAVMIKISNFLNSMRLFEDVLRTMRLNWLDVMRLSSGELEKKEIMQGLSMKARSRGRVYGLVVVMTGAMYGLMPLIGSNKVASLRDRSYPFFGRYLFDRNSDTVYRLCYLSQLMSGSVTAVANFATDAIFLFCVYHFCAQLRILQTDLLKLGGPRFDSREALVQLIRRHQKEIRNVRALQSLFSISSLQQLFLSCLMICLNGFKLIVSLCNREVDILMYIVCLPVTLFQILFYCQPGNELIVQSQSLDEAIQQSHWVNLDRLSKRQLFFMIQRSQKPLAITAGKIYVLSLENFMRIVKTAMSALSVLQAMYRKTGS |
| 283135146 | NvOR80 | MHCSYSFFLVTAFAMWRPRSWDDSKILTALYTLYSILSFTVYYTFLISQILDIVLLAENIQQITENMIQLINVVNVSQKSLCFFLKRKKIIRFMDYFFEDMTLPQSPREKEIQKSFDDESKGNSQKLFVLYSVSVVMYVYMPFFISKREDRVLPYRAWRPYSLDNVNYYYLAYLHQSWSVTIAATGNAATETLVSGFMIQICAQFEILEHRFMQLPKILKEMRENGESESTVLATERSIIIKLIHHHWRIFEMTELFNDIFVFVILSQFVTSITVLCVSTYNLALCKSVNNDFVTIFMYLLCMLLQIFMYTWYGNEITLRSCDLGNRIFLSEWRSLNPPTVKNLLIIAQRTMKPIILSSGYVITLSNVAFTSIVKTSYSVFNVLNV |
| 283135138 | NvOR81 | MHILSLTFTFFKIYGFWRPLSWKSPTLGFLYDVYTFVMFMIVFTFALSQLMSIILTVQTVDEFTSSSFILLSIVSACFKASNLLLKRKSLVRLLNVLISTTCKYQDDDEKMIQDMFDKKARRNTVWYMALIQSSVFMITLQSIFINIPQKTLPFPAWLPYNYSNTRLYAISYTHQVIGNAASATLHAANDALISGIMLQICAQLEILKHRILKLPTIVLKMNSGKEAPMNTVASKESELLGNIIKHHNCIFQFSKDINDTFSMALFAQFFIAALVICSSVYELSKIVLLSSDFVALLSYLSCMLVQIFLYCWYGTAVTMKSWSVGDTIFATDWSPLSMGLKKSLLIVMIRAKKPIELKTGKIFTLSILTFAKIIKASYSAFNFMQQA |
| 283135140 | NvOR82 | MRVLPITFGILTVCGFWRPISLESSIPKQMYNCYSIFMCFLIYTFTLSHLIDIVISAADFESLTGSCFMLLSMMNVCCKMTNILYFRKNIVELLQILASDHCTAKDVVERDIEKKFHKRARSVTLCYWILTETTCMLITLRTFFGSSKQILPFKAWIPYEITGLAVYWTTFFHQTIAHVAAANLQIANETLICGLMIQACSQLEILKYRLKKIPDESKIDKFPLQSTVNNAQNTNKKDTKTLLVNCIDHHRRIIEFSEKLNSTFNVILFVQFAISSLVLCSSVYLLSKMKLVSVHFMSLSLYLSCMLYQIFLFCWYGNEVILQSLDLGNAVYHMDWTILSTEDKKKLLIVILLVRKPIQFTSSFLVSLSIESYCKILKTSYSVFNLLQRTSI |
| 283135151 | NvOR85 | MRSLTFTFKVLSLCGIWLPLHWQSHRRLRLFYKIFSISTVVLTNIFILLQGLLLALSEFDWQFLAEILFTLLTAFSVSFKATNFLMRRDKIICLADMLLKSWCIPRNAVEIEMESRINEFLRVFTIYFNALAQLSLACLLIMPLVQDPDKRELPFRMWLPYDIRNQWNYWSTYVIEVGPMIVGILLNVTTDVVVSGFVLQACIQLDMLKHRLNKLPNIVKVAKRKRLASEEVVRSFERKTLHQAARHHDYIIKYAKVVTETFDVVIVEQFFAGALIFSVIIYVLTIGKVPILQKLMSVGYLICMLGELFAYCWFGNEITLKSLEFSDDIYKIDWMALSDSSNKKLIFIMMRATQPIIMSYGHLVILNIESFKSILKITYTAFNILKESTSTT |
| 299522742 | NvOR86 | MLELPYKLLILTGIWMPEDWTHKHQKLGWLIFSIISIGLVFMQFSSLVIFLMISKSCAQFFERVFLIPAGVSSLQKIYIFITHRKELIDLGKMLLKDYCIPRNFEELSIQHRYEELIRVLTLVCFVLVNITMMNLLVLPLVTNGENRTLPMNVWLPYPVDSDASYWLTYTHQTLGTLLLGTGAVGSTLMINGFMHQVCCQFEILSSRFQKLPQIIKRLQLLKKPNHLIYEYEKKSMKQYVQHHLYIFRVADTINDIFKSVIFQQFCISSIVVSASIFQLSTRPDKDMEFIMVFCYLICVLVEFLIYSWFGNELMLESLHFQTSVYQIDWTALSIGSGKDLVFIMMRASKPVIMYCGHFIILSLESYLGILKASYSVFNILRRSSN |
| 299522740 | NvOR87 | MHILYLPFKLLTLTGIWMPEDWTQKQQKLIWVLYSMVSIGLVFMQLSSQIGYLMQSKTWAQVNERLFFIPTGISSVHKIFIFIVHRKDLISLGNMLLKEYCIPRNAEELSIQERYNEIIRVLTLACAFLVNVTMMNLVTLPLVTSGDNRTLPMRVWLPYKVDSDMSYWLSYAHQTVGIVFVGTGAVGSTLMINGFMYQVCCQFEILSSRFQNLPLIIEKFQSLKKPNQLIYRYEKRVMRQNIRHHLYIFRFAEALNKIFKSVIFQQFCLSSIVVSVSIYQLSTRPEKDLEFIMVFFYLVCVLVEFLVYSWFGNELMLESLNFQQTIYEIDWTSLSTRSSRDLVLIMMRASKPIIMYCGHFIVLSLESYIGILKVSYSVFNILRMSEE |
| 299522744 | NvOR88 | MHTILQLPFKLMTLTGIWMPKEFTSQYEKQGWTLYSIASITLMAIQSLTLSITLILSENSEQFFETLFIVPTGLQNLQKIYVVVAHRKKLMDLEKMFSNDYCIPRNVEELLIQRKYDENIRILTLSCIILMNLTVANLIASPLFDAYFTTMNTRTLPMRIWLPYKMDLNIIFWLTFIQQSVGVIFVGYCIISTTLMINGFMYHVCCQFRILSCRFKKLPQVIDYFRSLKKPYNVIYQYERRAIKQNVQHHLCIFRIAENINDTFKSVIFQQFCISSIVVSASIFQLSTRQEIDMEFFMVLFYLICVLVDFYIYSWFGNQLMLESLNFQRSIYEIDWTTLSTNAGKDLVFIMMRASKPILMYCGHFVVLSLESYVGILKVSYSVLNLFRRSK |
| 299522746 | NvOR89 | MEIIEQLEKMRILQVPFKVLTWSGVWMPEDWTQNQRKLKYNLFSFVCIGLMTIQSCSLTVYLMMSKTWSQFVETLFLIPPGLSNLQKIFVIMLHRKKVIDLVNMFENGHCIPRTADEWSIQQRYDATIRVVTLVCFVLVNVTMVNMVTTPLFLKADERILPMKVWLPYSIETDFFYWLSYMHQTLGVTLVGSGIIGSTLLINGFVYQVCCQFEILSSRLEKLPQIIRNLRSLKKSDHLVHQYELKLIKQIVQHHLYLFSIAETVNEIFKSVIFQQFCVSSIVVSASIFQLSTKPDTKTEFIMVLFYSICLLVELFIYCWFGNKLMFESLNFHQAVYDADWTVLSNESGKDLMFIMMRASKPIIMYCGHFIVLSLETFLSILKVSYSVFNVLRRSHG |
| 283135153 | NvOR92 | MQSLKVSFTILTYCGIWQPIYWTSGWHRTSFNFCRVVFRPLPYLLASAQLARIALVDMSFEELTEVIFILLSIVNICCKSVSILMRRADLIKLTKMLGIVSASPQDSDEFNIQHQYHQFIRYVTLSSLVLVEITAITFLIPPFFQPENNRTLPFKIWLPYDYSMDKLFWITYFPESITIILASLISVSSNTLIFGFLIEACGQFELLNHRFMTMPLYIEDFAKGEKITTYEVCKLEKQLLSRNIRHHTFIFEFVDIFKKTFSSAIIGQYIVSSLVISTSVYQLSTNTTMDVVFFTNLLYLMCMLLEFFLYCWFGNELTVKSEDFGRKVFRTNWLALSTKSNKDIFVAMLRSSKPIIVSTGFFAVLSLESFMKIIKLSFSAFNVLRTASDYQ |
| 299522748 | NvOR93 | MHVLPESFMMFTCAGVWQPVHWSACDSRFLLYKLYTLFSIVLVYTLTISELMGAILLTQSLEDFTDISFLLISTISVCCKIASIIARRDRVIHLTEMLLEVQCIPKNVRELEITRKFDKIARFTALSCIVLAEATVVVMSTGPLFQKAENRTLPFKSWLPYDSTTTPCTFWLSYVHQTAAIVLCATVNVANDSLICGFMTHSCSQLELLNRRLLELPRAVKLKMKKLPRRLMCNVEAMIVSRHVKHHVHIFKFAENINVIFTPVILVQFCMSSIVLSLSVYQLAVRSANGIQFITMVMYLTCMLVQFFMYCWFGNEVTLKSVEFGQAIYNIEWTSLQVQTSKDLMIMMIRAKRPIIMSSGALVTLSIKSFTSILKASYSTFNVLQRSSHN |
| 299522752 | NvOR94 | MHVLPEAFNLATYIGLWEPTHLESSIARCFYKFYTCLSFALIILTMITQILAMLFFTKTLDEFAETAYMLLSAINASVKGVVILLRRKHVIDLAEMLLKKECVPINATEKRVCSYFNKISRYTVLSCIVLAEGTISALALLPVVFEQGELVLPLRAWYPYNAGSGLGYWLSYLHQAMALTLIAAYDVANDTIITGFMVQACAQLELMTCRFHRFSWRGSNAVMRNGARHQLRLFEKRMVAQSVRHHLLIFRFTEIINSIFAPVILVQFCLSSGVLCITVYQMSASKSNGLKVIVLSLYLVSMLVEFFLYCWFGNEVTLKSLGFNIAVCEMDWTAMHVQTLKELLIIMVRSTSPIFLSCGPLIKLSLESFTNILKISYSAFNVLKQFD |
| 299782498 | NvOR96 | MLSIHFQVLTISGVWCPNHSSSVQRIFYKCYSFIVVVLMYSLALSQLARIIFVKQSFNEFNDTFFISLSTNFACFKAASNLVNQKQIVSLVNMFKHNCCLAHNDSERSIQKQYNDSCSKIIISLLILVETSAFFVVVAPLCGTMDNQDLPYQVLLPYDLSNKLFFWLTFVHHSFGAVLFTAISITNDAVITGFMMHVCGQLIILQHRFALLSRSLANEVSKKGRITDFDMMLERHWLRQIVYHQNHISNIAKKICSTFNEIVICQFFISGLEICVSVYQLSVRNNNTVELCTYAIYLMVMLGQFFVYCYFGNEITLQSKITHRAIFDIDWTSFSLSLKKDLTLIMLYSSKPIAMSCGPFAHLTLESFTNILKTSYSIFSVLKTAT |
| 283436197 | NvOR98 | MNNQQMNEDLAPIPFRILKFCGWWRPLNMSTWRRAVYSCFTVIMLTLLVTITLTVLIGVTQMSATDDLFADNVFLMFALINSVFKATNVLLSRRRFIKMLEIVQDTRWRDLRNDEEIEIQDRYRKTIRKISVYFTTAVFVAIILRVVAPLLDLSDEIKLPVDAYCPCDIRHSSCYWTLYWHQALGTGVATLTHAAKDCLISALLLQTCAQLEILKNRLLSIADTCVVAGNKTGAADRVEKLEQKLIGDCVRDHESIFEFAKILNDSLNVMLFGQIAVTIPNLCLSIYLLSTQKIASMDFMMTTQFFSAVVIELFFFCWYGNEVTLNSLDVENAISEMDWTLLSTRSKKDLLMMMVRTSRPILFRVGPIMNMNIDSFLSIMKTSYSAFSVLQSTGD |
| 299522754 | NvOR99 | MKFQDSIEYQLLPIPFMVLTLCGTWCPENWSKKRKRIYKCVTTVLVSLGIILLVEMLVFIIVKSGKDNIDLENIFATICIAVGLYKKINILYHRPKLMNFISNYTKNEWNKPKNFEEATIHLNILSETRYISYAYAAFILVSIIFRSITPILESGTFIILPLDACYPYNADNFIAFSLTYLHQIISGVTLTCMHIGTDTLFVGLLLQMNYQLHILKNRLRQLGNSKTYKNNTQTIKDRELFIKSKISQRVREHESIFRFGYDLQKTFKPILMAQMVVVVPSVIINVYFLSIYTDRLNLKYFMTFFFALVSLMQIYMFCWYGNEILLSSSDVGDALYESNWFALDQSTKKIMLTMITRSSKIFLISAVAIIPLDIDTFIKIMKTSYSAFNLLQRTTAQ |
| 299522759 | NvOR100 | MHEKLIAIQKANVEYELLPFQFLLLTIWGIWHPKDWPVRLKNISNIIFIVVFCLDIIICFEMSIYLVLSIGTNDFKLVNIFFTSATITGIYKAIKTMQIRESFRTILLNYFNYEHLCSLNTKERMIRESNQAQIRKVTVIYSASMAGIFALNAIAPALSQPDSTMQLPVDAWYPYSIQKSLNYWLTYFHQIILGSSLICVHIGTDTLFVGLLLKLVCQINILRYRLQSLTSLCSKNFEHFNAMGRKFIYRYIHHQNEIYEFSKVLNNKFQAVLLIQVITSIPNLCINVYTLSKYSGIINMDYISIFFNTTSSLIQLFITCWYGNEVLLSSLQIKKSIYEMDWTKLDVPTKKLLIVIMARSLRPIAFSVAHVIPMNIESFIKIIKISNSAFNVLQQT |
| 299522757 | NvOR101 | MHLKFVGILRSNIEYELLPFQFVVLTVWNIWCPKDWPRRLKNTSIILFIVILILNFIMCTEMLIYFILSIGTEQFKLTNLFFVSASITGVYKSLKIMKNRKIIRCFVRNYFNHQWIKLLDDEENEIHEKINTRIRHITVTYFISMISIILMKDLGPIAESGLAIQLPADGWYPYDIENSVLFGITYVHQVILGSFVICAHVGIDTLFVGLLLKLLGQINILKHRLQILGNSLDHKMISLNKFESFQTVQKHLILECIHHHKRIYRFGEDLNKIFQEMLLILVVSSLPNICINIYALSSNLKNINMDYIATFFSTTSAFIQFFIACWFGNEVSLNSVEVRNAVYAMDWNKLDTPTQKLLIVVMARSLKPIEFSVGYIIPMNVDSFLKIIKASYTAFNLLQQTSSS |
| 299522761 | NvOR102 | MHSTLAAIVKNNIEYKILPFQFFLLTFLGIWCPSNWSLKSKTAHNVYFTFIFFLDFLICIEMFIHFVSSFGTDNFKLINFFLVSANITAVYKSIRLMQNREVLRYFIISYFDYEWTKSHDSVEHEINSKIDLRIRRVTVIYSASMIGIVLLKAMSPIAESNGISLPVDAWYPYSIEKSRWFWITYLHQVILGSSAVGAHIGIDTLFVGLLLKTSGQIHLLNYRLRNLMLLKECNFAKLKEYSEKNVVLRCIYHHKRIYRFGGDLNDKFQEILFILVVSSLPNICINVYSLSSYKGNINVQYIATIFSTTSALLQFFIACWYGNETTFDSLQVINAVYEMDWTNFHVSTKRLLIFIMLRASKPLKFSVAYIIPMNLDSFIKIIKASYTAFNLLQQTTN |
| 299522763 | NvOR103 | MDLSQCLEYRALPMQFYIFTLSGVWCPSNWTSLLKLSYNMYTTTIAISGILFWASMFVNLIITKNESEYFYENVFAISTLTYAMYKEFFVLKKRKEIQQMLKLSFDDEWYRPFDNREIQIIDHYAHETRWVTQVYAIGIIAGLATKAIMPMLNSNSAWVLPIEAWYPYNTSNLKNYLFAYTQQLMGGIPLICLHISVDSLFVGLILQMCIQLKLLQYRLQKTFSTDIDLQEEKNIERNIKISDVIIANYAFKHQCIFRLGNYLNQEFRGILAGQVMITIPNICINVYLLSQHRGGITLHLVDSFLCFTTCLMQIFLYCWYGNKIILLSIDVANTAYTTNWLSLNISSKKKLLTIMVRATRSIQFAAGTFIMNIDSFIEIIKTSYSAYRVLQKTS |
| 283135159 | NvOR105 | MIIRKTLEHQVLPIPFHILTLWGIWCPEHVQPRLRRFYFAFTCIVIISEILLTTEVFINLIIIIRNKRFELDVFFILTSLMNGLYKALNILLTRKRIAKLITIGFEDRWRFPRDDSEKKILQNYKFESWRIHLIYAGACLAGVTIKLVGPMMKQNADIEFPAPAWYPYDTNKPVYFWLAYVQQMFVGGATISMHIGADTMLSGLMLQSCIQLKLLKHRFKHFFQHYEQVKGRLHLSSTKRKVEIALMKQYICDHQFVYSYANKINRNFSGWLIAVLIVVVPNICINVYLLSFSKIGLNVDFITSLGLFSISLFQIYLPCWYGNEVMLHSSEIANSIYDMDWVRLSPTARKTLIIVMIRSSKPIQIRAGYFVSMNLRSFLSIMKTSYSALSVLQQTT |
| 299522765 | NvOR106 | MTTTTIIERTGSADVYGIENRLFSISFNVIKLSGFWRPTTFRKPFDYLYEMYTLFCLVGILMLIATIIVDNVVTEKSIRSLIENLYLILTVSNGISKLCNIYHRRDRVISMLQRSSEDRWSVHRDEEEARIVEESIESESYIIRFCIYLVTINNVSNALNPILNPDPEHDLMVDAYSPCDRSKSALCFWTAYLYQVFGYVSTSLVHVGCDCLVFNFVDRLCAHLKILEHRILQLPDLVEANACDEIRYLKSCIEDHHSICEGIKELNDTFYETIFIQFVTSISVLCTNIYLLSMQDLFSAEFIAVFVYLCCAFVQNFFYCWYGYKVSVNTLHISDAIFNMNWCILKRESKKILSYVMMKTSQKVFLFNSAVVTLTPESFVNILKVSYSAFNILQQTK |
| 299522767 | NvOR107 | MEVMDTIKSTDIMPLPFFYLKLSGAWKPSSWPSYLRLIYDSYTILMTFFIMKVIIVTEILYVIFAEENQSKVLKDNVYIICTFINGWFKMFNLICRRKNIANLVKGCIAKQWNPPRDNYESSVLAATKQTSRKITLAHASVVGSCVVSTLLNSVLSSPPFLPVDAWYPCNITLPICFWTSFVHQSIGYTVTAIVHVANDNIVVGFMMQICAQLNVLNRRLLLVHVEVEKAARQQKDQSQITSLETTLVNDCIVNYRDILKFAEQLSETFIETIFIQFCAGLSVICTSVYVLTTLNIFSFEFFGMFLYLWCMLGQMFLYCWFGNEVVLNSSKLFHSIYNMDWIKLQSQTQTKLLFMMLVASSPIQLFRGAIIRVNLDAFINILKFSYSAFNILH |
| 645038719 | NvOR110 | MDDDILCVRSLQINSVSSKIRKMKEMNPAKSVDVLSTSFLYFKLIGAWRPLNLPKWLRVIYDLFTISMVILMYEMLIVTEILAIIFAEENRLKVFQDIVHITITHVSGCFKMLFVINRRQSIMLLVNGCVAKQWYPPRNELEATILTKHNNLSRRITLTYATLVGASLLAAVLNPILYSTRVLTIATWYPCNISLPICYWSSYAHQTMGILAMAIAHVATDSLIVGFTIKICTQLNVLNQRLLSINFQLENTSARCQKSQEQSLALEAILVNECIVNYKDILRFADLLSRTFIEIVFIQFCVGLTVICSTVYLLAKLSIFSYDFFGLFLYLGCMLMQMFLFCWYGNEVVLDSTKLFHTIYNINWIELQIQTQSKLLLMMLVASSPIQLFRGAIIKVNLDAFINILKFSYSAFNLLQKSS |
| 299522773 | NvOR111 | MDEIIDPIKRTDVLPISFLYLKLVGAWKPLDLPKCLRLIYDLFTIFMVIFICKLLIISDILCVVFAEENRFAVFKGIVHVTITHLSGWIKMLHVLSRRRSIMLLVNGCVAKQWNPPRDRHEASILTSFDNSSRRTTIAYTIQVSAAVSMLVLSPVFSSTWFLPIDNWYPCNISSPICFWPSYVHQSMGIVAIAVAHVATDTLIVGFMIQICTQLNILNHRLLSIHIKLEDTARRQKNQEQISAVETLLVNECISHYIDILKFADLLSKTFVEVVFIQFCVGFSVICSIVYLLAILSILSFDFFGTLFYLGGMLSQMFIYCWYGNEVVLNSTKLFHTIYNMNWVAFQIKTQKKLLLMMLVALSPIQLFEGAIIKVNLDAFINVLKFSYSAFNILQKSS |
| 299522771 | NvOR113 | MMSEIFPTTYFFLKISGFWRPYSLKLPLYLCYQIYTAFSFATVLSLIILLVLYCAFAHDKFLELLLENMYLVISFSNCISKTSNIILRKKNVEKLLQWIREKRWLAERDLEESCIVAHSKLMEKAIPQFCTLLVCANGIGNLMNPIIRANPDKKLVIEAYPICDRSRPVCFWLTYLHQCFGFVIVNVIHVACDCLIYNFIDRTCAHLKILGHRLQKLPVLVKGIRHQGIDTVEFEKSYVIDCIKNHQGIFIFIRELNDTFCETVFFQFLSSILVLCTNIFLLSKQELFSPEFIAVFSYFCCVLAQNFFYCWYGYKLSVNSLAFVDAIAKINWVELDMKTKKMLVYMMLITSNKVELFNNAVVNLSPASFINIVKISYSAFTVLQRTSHKEMKI |
| 299522775 | NvOR114 | MLRTEELLANSRANERRNERVGIEDQVFPATFLLLKAAGVWTPTTLKLRSQYMCYRIYSAFCFISVLALVVTVSIENVVSSNASILESWYMLVIFSHGLLKIKNLQWRRVKVIHLLKECIMNERWSIARNQDERAIINESKRAEKFITHLWLSLLLVNGLGNALNPLIHENPNNSLIFECYSPCDRSLPSCFWTAYAYQLFGYAISSTVHVGCDCLIFNFIERINAHTMIFIDRLQKLPSRVVEGKNEGCLDASRHEARLLKECIQDHRRIYESVEELNNTFYEVVTIQFLTTISIVCTNIYFLSKQELFSADFIGVLVFLVCVLTQNFIFCWYGYKLSESSSYIVNAIFNMDWLVLNKRSKGLLLFAMMSASNEIKIFHNALVNLSPETFLQFVKMSYSAFNLLQQSN |
| 299522777 | NvOR115 | MHQAIKIANGRIDADGIPNVEGLEKRVFPRTFLLLIVGGMWAPTTIKSRALFACYQLYTVFCFVSVCMLIITILIDNVLSDDKTMESLVEYAYMLIVFSNGLVRIINLVSRRDKILRLLQGNIMLDRWQSLRDDEELAIIAESKVSEKLVLKIWGSLILMNGISNAVNPIIHENPDNTLMFECYSPCDRSVSYCFWMTYSYQLFGYVIMSVAHLGVDCLIYNIIDQINSHYKIFLNRLLKLPARVREKARDDVAAALRYENNYIKECVADHHSIYKAAGELNDIFNELVFIQFISCISLLCTNIYFLSKQELFSPPFIAVFAFLCCALTQNFFFCLFGNKLSQTGSEIAGAIFGMDWQELQKETRRKLLFIMLLTSKGIALFNNAVVNLSPETFLKLVKVSYSAFNLLNQSTHK |
| 299522779 | NvOR117 | MDLLPVHFRTFQFFGLWYNDPCSYRLIKLVHRSLIVLLIVHLSLFQMIALFSAKRNVDEYTNTLFLALTYFVHIYKTLVFMAKNRSVNEMLDEFRSDICRTRGPEEEHILAKHVQRANWAYSGRMILTLFAGSIRVVLPILIGFSTGKLELLPFDTYFFNVKHLVQYALVYVLQTLAIITVIVTDVCLDSTPCACMILACAQLEICRHRIKHDNMVLYENSEDGPGRGFNEEMALKEYVKHYVLIQEAVHRIQSVFIAIVLPIFSSALLTLCTSIFQLAQKNHTTGEYCFIILYLCCLLVQTFSLCWFGNELQSKGEIVTSAVYETDWTVLKPCLKKSYRYLMFMGQNKFIISFHGQCTLTLQTFIWMIKTSYGAFNLLKQVADT |
| 299522781 | NvOR118 | MDLLPMHFRTFRFFGLWYDDPRSYGLAKLVHRSLVVILIVHMSLFQMIALFSVKHSVDEYTNTLFIALTYFVNIYKILAFMAKNRSVNEMLDKFRLDVCRTRDAEEERILAQYLHTANWTYSARMILTLCTGVIQIVVPFLIGYFTGKVGLLPFDTFFFNVEDLAQYALVYALQAVAIVTVVITDVTLDSTPCACMILACAQLEICRYRIKHDNNVIANEVTGDGEMNSECKPGKELREKMALKKYVKHYVLMREIVDRIQSVFISIVLPIFCSALLTLCFSTFQLAQKNQTTGEYCFIITYLCCLLVQIFCLCWFGNELQFKGEIVSNAVYETDWTVMKPRIKKSYWYLMFMGQNKFIISFHGQCTLTLQTFIWMIKASYGAFNLLNQVADTKY |
| 299522786 | NvOR119 | MDLLPMHFRTFQFFGLWYSDWRSYRFFKLVHRSLELLLIVHVCLLQIIALFSVKHSVEEYTNSLFIGLTHFANIYKTVVFMVKNQSINEMLDKFRLDICRARGREEEQILAKYLHKANWTYSARMILTLFCGSISIVVPILVGIFTGKLELLPLDTYFFNVNDLKQWTLAYVLQSLTVITVVVTDVCLDSTPCAFMILACAQLEICRHRIKHDNMASHEIGKDVPRKGYKEEMALKEYVKHYVLIREVVYQIQSVFISIILPIFCSALLTLCTSIFELAQVIVFLFNYHTTGEYCFIMSYLCCLLVQIFCLCWFGNELQLKGEIVSNAIYETDWTVMKPCTKRDYWYLMFMGQNKFIISFHGQCTLTLQTFIWMIKTSYGAFNLLTQVADT |
| 645018833 | NvOR122 | MDVLPLNFRTLWLCGIWHEENEKLTVPRIAYRFLVICLMFYFTFTLSAVVFVENSNVSELTEAIFLAVTYITLCLKIVNFAFRRAEMIEILHDFRHPYCKAEHSEESEILKGYSKQARKMYIYLMAFVMSDVAYFWSTFAFKVSKNIMELPYHTYQFYNMSSKAILFSTAALQATSVLYSVSINISFDTMTAGLLILTTGQLELNAHRLSKLGEHNVDSMNGYIAHNVLINGTVDKIESFIKTVVIPFLFFSLLSICASVFQLSEYSVFSLEFLGLFSFAICILLQVLVYCWFGNELMLKSEAVTDAIYRSDWTMLSPQNRKSLQVMMICNKDGRTVSFGGQCSLTLETFVWILKTSYATISLLNRVSA |
| 299522788 | NvOR124 | MDILPLNFRILRYCGIWYELPEHLWLVKIVYKIFVVVVIFSFTLSELIELALTYDDLQNLTECLFLTLTFLALCFKMINFMCRQESLKALLNTFRDEICQPKTLEEKDIIEKNRSMLRLFCISYFSLGILSGSTLVFVPFASFKSSKIELPIKTYQPYDVEDFVLFSLTYFHQILSMYLGVLINVSLDMLVCGFIFLTCGQLDLCYYRIVSSNMYTMNNNIRHHAVTKDIVKKMQSFSIVVVVPLFIFSLITLCTSLFLMPEKEIMSFEFITLFIYLTCMLTQIFLYCWFGNELQLKSKTISDAVYHSNWTRLTPKLRRNLLFTMFISQNGLMISFHGQCSLSINTYVSILKTSYAAFNLLRKTSNTLGV |
| 299522793 | NvOR125 | MDMLPSFRVLAYLGIWIEEGSSFVFLRRLCGLFLSNTIFYFTLTEVIELYLLRNNIEELVDVMFLTVTFAMLCLKILNFNFRHKGLLNLLTDFRMDVCKARSPEEENILNKYTTKILNIFQNILVLSQATGIFFCVLPFITLEPADYEIPYKTYQFYDDTTAMGFTITCVIQFIALIFGIFINVSMDTMIYGFIILSTGQFELISYRINKSSKENDRALLKQCIMHHNCMNNLVKKTTNLFMTVIAPLFFFSLLTLCASIFQMSQNDIISLEFLGFAMYLSCMLCQVFLYCWYGNELKLKSADLVNEVFGSDWTVLEYTEKKTLYLLMLSAQRPCDISWRGQCTLSLETFVWIMKTSYTAFNLLQRASDK |
| 299522795 | NvOR126 | MDVLPLNFRSLQYCGIWYEFPEHLWLIKTVYRTFIVVVIFSFTLSELIELALTYDDLQNLTECLFLALTFLALCFKMINFMCRQESLKALLNTFRDEICQPKTLEEKGILAKYQNILKKVFIFYMSLGLMSGSSLLIVPLVSMENSRISTLPMKAYQPYDVEDTILLNITYFYQVFSTWIGIIINVSLDLMVCGFIILICGQLDLCCYRILCTKTKMFHDNNVRHHAVIAEVVRRVKSFFIVVIVPLFIFSLITLCTSLFQMPEKEVLSLEFFSLFMYLSCMLFQIFIYCWFGNELQLKSKTIVDAVYQSDWTDLTPKLRRHLLFTMFISQNGLTISFHGQCSLSINTYVSILKTSYGVFNLLQKTSNI |
| 299522797 | NvOR128 | MDVLHLNFRVLQYCGIWYEYPENLWLVKMVYKTLIVVMLFCFTLSELTELVLNRNNVHDLTECLFLSLTFLTCCFKMINFLCRQEGLNRILNAYRADVFQPKTTEEKQMTTQYQNLISKFFMVYLIMALLSGICLSLIPIISSASNETQFPAKSYQPYNTQDSTLYLITYFHQILSIFFGIFINVSMDMLVCGFIILACCQLDLCCYRISLNKKDTSTNDHVVHHVLIGHAVNRVQSFFIVIIVLLFIFNLIVLCTSLFQIPQKNIMTLEFFTLFVYLVGILFQVYVYCWFGNQLQLKSKTISDAIYESNWPDLTPCKRKDYIFSMFMSQNGFTISFHGQCSLSIKTYVWIVKTSYAVYNLLQNTST |
| 299522799 | NvOR129 | MEIYDSRYFVHAKRFQELLGIWPYQSRLKNNCSWVILSFLFIAMIIPQIVGLSVHAGKDSKRTLECTFGTCYMLAIYMKLLVACADKDKAKFIFEYTARNFKKINDNDERKILIEYSERGRLIGVVYTIFVLAALGVFVVVPLAPGILDVILPLENGTRSKFFILNGEFLVDKTEYFIEIYAFDSICCIVTVLIICATDPLYAAILEHCLAIFAIVKLRLRKYRVRKGLKCVSADEAEYEAIIRAVQLHREIIAFIETIQNNCSLYFAFEMGVTLISFTVNFVLAVLKTPDLFDRLRLAMVLFAQAVHLFYITWPGQKLIDHGEDLFKETYFNDWYKSSVKCQKALRFMSLRCSKPCKLSGAGVYVLNFAYTLLILKTSASYITVVAQFEYKIVA |
| 299782532 | NvOR130 | MEIYDSRYFVFNKRFQMALGIWPYQSRVKNSITYAGLVLVMIIMLIPQFIRLNTYLGKDIEKTMENIFIFFYVFGIFVKLFTAHFAEDKLKILYESTAKNFETYTDAVEAEIMKRYSERGRLLTFVFLLYMISAVAVSVVLPMCPIVLDSTDPLDQPRPRMFILNGEYIVDKYEYYFQIYTLDIISVFLMICILCATDPMYAAIVEHCLGLFSICKYRLRNFNKSCGLQMVEHAEAERYGGDYAYAALVRAILLHKDIIKFTEIIQTSYSLYFLLEMGATIGILTSSSVVVVMKLKQPLELLRWSLFLFGVILHIFFLTWPGQKLIDFSSDIFQEAYLNDWYKSSLKCQNLLKFMSLRCSRPCELSGGGLYIMNFINFATILKTSASYITVFSSV |
| 299522801 | NvOR132 | MEIYDSRYFVYNKRFQTALGVWPYQSRVKNAIICGFLLLVMIALVVPQIIRLKMYIGKDKDKSMENVFGLFYIFAIYVKLFTAVYAEDRLKILYESTARNFQIYTDKMEKKILHENSERGRLITLVFIMYMMTALIVFILLPMYPIMTDVIVPLDHPRARMFILNGDYLVDRDEYYFQIYVFESTSAALTVFILCSTDPMYAAIVEHCLGLFCICKYRLNNFNKPRRTEIIEKANAESQVDEYAYTALVEAIQLHKNILKYTKIIQTSYSLYFLLEMGATMGLLTSTSIIIVMKLYRPLDCIRYFLVLIGLLLHIFFLSWPGQKLINVSGDIFQDTYHNDWYESSLRCQRLLRFMSLNCSKPCQLSGGGLYVMNFVNFARILKTSASYITVFSSF |
| 299522803 | NvOR133 | MEIYDSRYFIHNKRFQKALGVWPYQSRAKNIVVCGLLLLLMLGMLLPQIVRLKKYAGKDSDKMMENIFILFYIFGIYIKLFTAVYAENRLKVLYESTAKNFQIYTGEAERRILYEYSERGRLLTLAFIVYMLPAVTVYVMLPMCPIIMDAAKPLDHPRYRMFILNGDYLVDEYDYYFYIYAFDSMAAIVTVAIMCATDPMYAAIVEHCLGLFSICKLRLKNFNKPNGTKAIEKTYYYSETCGDEYAYAALVKVVQLHKDIFKYTEIMQASYSLYFLLEMGVTMGVVVCNSVIIVMKLSQPLELVRWSLVLIGGLLHIFFLTWPGQKLINFSGDIFQDTYLNDWYESSLRCQKLLKFMSLRCLKPCELSGGGLYVMNFINFATILKTSASYITVFSSF |
| 299782491 | NvOR134 | MEIYDSKYFIHNKRFQMALGVWPYQNRVKNLSICGVLLLVMFGMLIPQLLRLRTYLGKDIDKSMENIFILLYTFGIYIKLFTAHIAENKMKILYESTAKNFETYTDEAEKKIMKQYSERGRLITLAFLIYMVLALILFVMLPLYPIIMDATIPLDLPRPRISVLNGDYLVDENDYYFQIYVFDSIACTLTVFIMCSTDPMYAAIVEHCLGLFSICKYRLKNFNKSCGMRMVERADAERYGGDYAYAALVRAILLHKEILKYTQIIQTSYSLYFLLEMGVTVGILTATSVIIVMKLERPLDCLRYFLVFIGLLVHIFYLTWPGQKLIDFSGDIFQDTYLNDWYKSSLKCQNLLRFMSLRCSRPCELSGCGLYVMNLINFAAILKSSASYITVFSSV |
| 299522805 | NvOR135 | MELFDSRYFIINNTCMKLLGIWPYSSHVKNYLRRCGLGLFLLSCYLPQFIPLYMYFGEDMDQMIQNIGVILYVFGTSVKLITGVTAKDRMKIVYEKTARDFQTIVDKEERNILFEYSERGRTLSITFIIYMWIALAIYVGLPMGPLVLDYFIPLQNGSRERGFVWKGEYLVDPDKYYLTIYAVELFSSVLSVTILSSVGPMYQAIVEHCLGLFVIVKFRLQICTRGGKKAEEESYRLIVKIIRLHNDIIEFTRIIEASYTSYFFIEMDITISLVTLISVNLISRLDYLFDSIRHIFILLGVMIHMFYLTWPSQKMINHSTDLFHDTYSNEWYNCSIRCQNLLKFVALRCVEPSQLTARGLYVMNFENYASLVKTSASYITVLLSFR |
| 299522807 | NvOR137 | MDIFDRRYFVLNKALLRSTGLWPYEDRRKKLYIRTFVNLILGICVIFPQIVRIYNYFGVNMNMVLEHAAVLMYITSIYLKFLTSVYYEEKLRVVYDNIAKNWQVIKDENEINILIQYSENGRLLTIGYTMYIIAAFCSYVFLPIVPVLLDVFNPLNQTRSRFYILGGEYFIINNVEDYGKVYAFDCLAVIVTVWLISAVDSMYAASIEHCLGLFAIVKLRLQTCTRSICDGQKDECYKMIVRLIRMHKDIINFTDILESSYSSSFLILVGINVIFLSFECIIVLTRFGQAMEMMRYSMIMVGIVVHLFYISWPGQKLIDFSLGLFQDAYLNEWYTCPTRAQKLLGLMTLRCSKPCQLTAGGMYVMNFSNFAKIVKTSMSYMTVLASFR |
| 645009713 | NvOR139 | MEIFDQHYFSVNKALLKSTGLWPYESRRRKFCIRTFINLILGVFVIFPQLVRIYNYFGVNMDMVLEHAAILLFILTTYLKFLTSVYYEEKLKVVYDNIAKNWQAIKDENEVNILSQYSESGWFLTISYIMYIVIAASAYSLLPMAPVLLDMIDPLNETRPRLYILGGEYFIVDNVEDYGKVYAFELVPAAVTVWLICAVDSMYAASIEHCLGLLAIVKLRLQMCTQPSCDSRKDVSYRLIVQLIRLHKDIINFTDILESSYSSSFLILVGVNVLFLSFECIIVLTRFGQTMELIRYSMIMVGIVVHLFYLSWPGQKLTDLSIGLFQDAYLNEWYTCSTRAQKLLNLMILRCSKPCQLTAGGIYVMNFSNFAKIVKTSMSYMTVFASFR |
| 299522811 | NvOR140 | MEIYDSRYFIINKTLMTKMGLWPYQHPLKKFLVRTFLVVFIFVSSMPQLYGLKKNFGVHMDKIIEHLALLMYIYGIKLKLVTSILSEKKLKKVYENIMENWQQIKDVHERAILVEYSERGRTLTIGYIMYMTSALLFFIILPITPMVLNVIKPLNESRPWDFIMHGEFPVNDMHAHYGEIYLFDSLACIATVLVFCTVDSMYATCIEHCIGLFAIVKSRLDLSTKFVNRQGALGIKRDDKVYDLIVKTIKLHKKIINFTHILESSYSTSFLILMGMNMLYCSLVSVLLIIKSDALMERIRYGTILLGLLIHLFYISWPGQKIIDLSTGLFEDAYSNEWYETSIRSQNLLKFMRLRCLTPCQLTAGGIYVMNFANFASIIKTSTSYITVFASFT |
| 283135164 | NvOR141 | MDIYDTYVLNINKKLLSFVGIWPYEEKKKNKFTRVFYLITMFCIIVPQMIGFYQHFGVDIDELLENTGTIFFTLSIYTKLFTSIIFENKLKILYDSVAKNWKNITEKHEREILVKYSERGRMLTLGYITYNFAAVIVYTTMPLMPFLLDIILPLNESRPSMFILNGQFYVDKHEHYKKLYAFDCLCIFVIVPAALAVDTMYVACTEHCLGLFAIIKYRLAMSDKFISTRDIYLTEEKDSSYRWMIHTIRMHIDILKFANILDKSYSSSFVILMLINTVYVSVLCVLVLISLDKPLNLIRYYMLLVAICIHLFYLSWPGQKLIDHSEGLFRDAYNNQWYEGSAKSKTLLKILTLRCVEPCLITAGGLVTMNFATYLTIMKKSVSFITVFSSFR |
| 299522813 | NvOR142 | MTMDFYNSRYFSINRRMMTIMGLWPYQDFKTKLFIRTFLAIVLGIALIPQIISIVKYTNEDSDKVIQGIATLLYVTGITLKILTTITSEKKIEIVYRNIVDNWKLLDDENEIRTMTEYSEFGRLLTIGYVAYMFFALGLFVTMPMLPMMIDVISPINGSRPRIFILDGEYIADKNENYGKVYIFESLTCIMSVFVFSTVDSTYAVCVEQCVGLMAVVRLRLKLATAKAARMKYKSDSDEHDIPYQLVSSSAKLHIKAISFARILDSSYSVNFLLSMGSNVMILSVGSVVILINLGRPMEFIRYSMIFIGLMIHMFYLSWPGQKVIDSSQGILYDAYNNEWYECSKKTKTLLKFMMLRCIEPCQLTAGGLYVMNIANFGSLAKTSMSYITVFASFR |
| 645004222 | NvOR143 | MDFLDSRYFILNKKMLHILGIWPYQKRLERYAIRSVYFFFMGVSFVPQILCVKKYFKVDSDKFIRGVTTLLYLSGVSLKLTIAILMNGKIQIVYSKVADNWKMFTDKDEIKTLLEYSEVGRMLTLGYVVYMVLAVIVFITMPYLPVVIDIVFPINGTRPRLFVLDGEYIVDKYENYNKIYIFESVCSVVSVPIFCTIDSTYAVCVQQCVALLAIVKLRLKVATKYTKNYLRDHKYNDASQQLIIKSADLHNKVIEFAQILETSYSMVFLLLMGMNCLILSVGTLVILVNLNNPLELSRYIMIFIGLMMHMFYVSYPGQQLIDRSSAIFNDAYNNEWYECSIKSQRLLAFMMLRCTKPCELTAGGIYTMNLENFGSLVKTSISYIAVFASFT |
| 645000979 | NvOR145 | MDKLKQSTVDIDTINNIFGNTYFKINKELQELVGLWPYQKGFSVRVVQTIMLFVLSFIMIPHLNGIRVWCGKDLGICSENIAATIYLSGCFLKYLVVLLCKRDISKVYEKIAINWLTINDPNERVILDKFSSLGKLKSIGYTVYVSAAGIGFSQFALLPFAFDYFSPLQNGSRPKIRIVRAEFFVDPIEYYWHIYATYCIVTFVSAFTIISIDTSYTAVVHQNLGIFNIVKYRLSLAKKAVGTSKDLAYEQIISAVRLHQDSLGFNNLIEVTYRVCFLLLILVCISFLTFGAITILENSDNWIDIVRLGSIEVGAVIHLFYLSWPGQLVVSESEELYYYTYNNEWYNLSAESKTLLHFMMLRCINPCCLTAAGLYVMNFENYGAIIKSTVSYITVLSSFRE |
| 299522817 | NvOR146 | MNGFETSSLNSKIINEVFDNTYFKINKILQELIGIWPYQKRFDALIKQFIVILILSMVAMPHINGIRVWCGKDLGLCAENLAGVTYASGVCTKYFVVTRSKDQMTKVYEKITSNWLTITDPDERVILNKFALLGKYKSIGYVGYITVAAICFSQLGLLPILIDVVLPLQNGTREKLRVVKAEFGVDPYDYYWHIYGAYCAISVVSSGVLMAIDTSYTAVVHQNLAIFNIVKYRLTQAKRAVNTVKDVAYEQIISAIRLHQDSLEFNNLIEHTYDVSFLILILICVTFLTFGAITIMEESDSYLDMFRLSLLECGVCIHLFYLSWPGQLVVTESEDLYYYTYNNEWYNLSEKSKTLLKFMMLRCMKPCCLTAAGLYVMNFENYGAIIKSTVSYITVVASFRED |
| 299522819 | NvOR147 | MTNEISQEKLNRVFDSHYFYLNKKLQIVSGLWPYQSRKRKFIHKLTMLCFLGTALVFLLNGLRHWCGVDIDVCGENLVGLIYVISVLSKLFITSLYEEKFKIIYTRLAINWLELTDPQEHNILISFARQAKIKTVVYFVYMAAAGVGFCQIPMIPVFLDFINPLNETRPKILFVKAEFIFDPYKYFYQLYAFFIGCAASAVFIVCSIDTTFTAVVHQIIGVVSIIKYRLNCATVSFNPNKDVSYKLIVHAIQLHKEVLQFSDLIEKSYNIFFLVLTGLTVVFLSTGAIVMLVRVGAMLDLIRLVLVLIGAILHFLFLTWPGQNLIDHTSDLFAAIYATEWYNVSERSKKLLSIIMLRSLKPCVFTAGGLYVMNLENFGSIMKTAVSYMAVVSSFR |
| 299782500 | NvOR149 | MNKEEVDEAFNDSLLKINKELNIFNGLWPHRPDGDKLFRRIIVLTVLISVTLPHVLGMFIQCGRNMALCGENICGFCYCSGVIAKFIVPIVSKEKFITLYEKIALNWKEITDPYEQSILEEFSKLGRLKSWLYFVYCAVAGFAFCQMTALPALMDIILPLNESRPKILVTKAEYPFDPFEYYYELYFLYCTAAVVSVSVLASTDSTYSVIIHQSLGIFGIVNHRLQKAAKHKNQEESYRVMVSAIELHKSALEFLELIESTYQSAFLIFIFVTVAFLSFGSLIIVEHSEEIIDLIRMTLIEFGAMIHIFFISWPGQLVIDHSENLFLSTYTTKWYNMSKKGKMLLLFMMMRCLKPSFLTAGGFYIMNFENYGSIVKTTLSYVTVALSFH |
| 299522821 | NvOR151 | MDEREIDLLYDNYYFKLNKKLQIITGLWPYKSRKYKLGIRAVVYATLSLVMIPLCNGFRTWCGVNLDICGENLVGICYTMLIFLKYWVTTHSEERLKNVYRLVAKNWMEITDPHEHEILVDYAKQGRLKTIGYTAYVVVAGIGFCQIPMISVILDIIIPLNVSRTKILFKGGEFILDPYQHFYKLYVYFVITSFVIMTIIIAIDTNYTIIIHQILGLLTIVKHRLQRLAIPMNLKKDNSYHAIIKAIHLHNDALQFVDLIESSYSCLFLVFIGFTIIIISISTSIMMAQIGKLLNMIRVAMFVLGASLHFLYINWTGQQMIDHSKELYLNVYSNEWYNLTKEAKTLLKIVMLRCLKPSKFTAGGLYTLNLESFGTIMESALTYVAIMSSFR |
| 299782505 | NvOR153 | MNKKEVDKAFNDSWLQINKELNVFNGLWPNRPNGDKIFRRFIVLTVLITVTLPHVLGIVMQCGSNMALCGENVCGFCYCSGVIAKFIVPIVSKKKFVTLYEKIALNWKDITDPYEQSILEEFSKLGRLKTWFYFVYCIVAGFAFCQMTALPALMDIIQPLNESRPKILVTKAEYPFNPFDYYYELYFIYCTAAVVSVSVLVSTDSTYSVIIHQSLGIFSIVNHRLQKAAQHKNPDESYRVMVSAIKLHKSALQFLELIESTYQSVFLMFIFVTVAFLSFGSLIIVEHSEEIIDLIRMTMMELGAMIHIFFISWPGQLVIDHSENLFLSTYTTKWYNMSMKGKILLQFMMMRCFKPSFLTAGGFYIMNFENYGSIVKTTLSYVTVALSFH |
| 283135167 | NvOR154 | MDIYDSRYYKTNIFYLKLLGLWPFDDFLNKRVRRILIIIAVVSLIIPQVIRLFEEWGRDIDIVIEVIGSLIYFSGCQIKYLSFLRVEAKMKYLYNKIAEHWKSLSSKDEIKTLEEYGEIGRGLTLGYIIPINIILVIYISLPLLPLLLDVIDPQNETRPKQFPYFAEYFIDDQKYYFELTIHGWIVCILSVQIYGTFDTTYTQCVQHACGLFGIVEQRLRKATKLASSNAFSTQEEKDEKVYDKVIDAILLHKEAIQFVNLIEDCYSFSYFFVVTLNTAVVSLAAVDTMLNLENGNTKQMVRIGALYIGFSFHLLYNMSPGQRVIDSSTNIQNAAFHCDWFNASSKTKTLIRIIMLRSLTPCQFTAGKLIVLHLESFAFVFKNSISYVTVVGSMR |
| 299782503 | NvOR156 | MSESKTLKIFESDYYRTYKNSVKLIGLWPHENIHKKRITRFFITALLTTFMILQGIRLYEELGNDIDIVLELIGSIAYFSGCICKYLTTIKAQAALQFLYEQIQGHWDTITNKRERQILEQSASESQFLSKFYMGASYVALVVYTASPVLVPIILDIALPLNESRRKTFPYFIEYFIDTEFYYYQLMVHGTICFTISVLVYISIDTMYAACCQHLCGLFDIVEHRLKEAVKTNSNRINLEPDRTDILMHKLLNEAITLHQDSIEFAVLIENTYALCYLLVLGLNLAVIVLAAVDIVINLDDTNQIIRLSILYIAFSFHLFFNSVPGQKIHDKSVNVMNSAYFSEWYNLPLNARKLIQLIIHRSLNPCQFTAGGLFVLNIENFGSIMKSSMSYITVLASIR |
| 299522823 | NvOR157 | MDIFNSRFYRTNCFFLKLLGLWPLGDVSNNRIKRVTVVSLVVSLIIPMVIKLVQEWGNDIDIVIEVIGSLIYLSGSQLKYISCASVQSQIKFLYTEIERHWNTLTNEEEKKILKQYARDGYNLSFGYLMLLNVILVGYLLVPFTPMLLDLIDPLNETRPKAFPYFAEYFIDNQKYYFELTVHGWIICILSVQIYGTFDATYTQLVQHSCALFAIVEYRLGQATKMVASDEDSSHKDTDKVAYNMMVGAINYHKQAIQFVGLIEKCYSLLSFLIIILNTAVVSLAAVVTMLHIEKGNQKQAIRIGMLYVAFSFHLLYNSYPGQKVIDSSTRIQEAAFHCEWFNTSSKTKQLIKIIMLRSMVPCTLTAKTLVVLDLESFAFVFKKSISYITVIGSMR |
| 299522825 | NvOR158 | MSESKRLEIFESDYYRMYTNALRLIGLWPFECTYRQRIIRFFIIILLITFTILQGIRLYEEFGQNLDIVLELIGSITFFIGCILKYIVTIQTQSMFQFLYEQIQSHWEIVTNRERRILEQSANDSQFFTKLYMGAAYGALIVYVSTPIIVPNVLDVVIPLNESRAKTFPYFIEYFIDTEVYYYQLMAHGTLCFTISALVYVSIDTMYATCSQHLCSLFDVVEYRLEKASKTDSKMNVNLDLNGNDKNIYKLLNEAIVLHQDSLEFALFIENTYASCFLPVLGLYLTSIVIVAVDIVINLGDMNQIIRLSILYFAFSFHVFFNCVPGQKIHDKSVSIMNSAYFSEWYNLPLEAKKLIQIIIHRSSIPCKFTAGGIVVLNVENFIVIMKSTLSYITLLSSIR |
| 299523072 | NvOR159 | MSVRKDMHVFESQYYRIYKNSVKIIGLWPYENIQIKRVIRISIILLLISLVILQAIRLYEELGRDLDIVLELIASLSYFAGCLSKYITTIRAQSAFRFLYDLIAGHWQIITDIKEREILEESTRQSQTLCLSYMVAAYSALVVYSTMPAIAPAVLDIVIPLNESRKKTFPYYAEYFIDDEAYYYQLMGHGTIVFTVSVMVYVSIDTMYACCAQHLCGLFSIVEYRLQEALRTDDKLHLEPPERDKLTHKKLHEAIILHKDSIEFAFLIENTYALCFLLVMGLNLTVIVFTAVVIIINLGDMKQMIRLTLLFGAFSFHLFFNCVPGQKVHDKSISIMNSAYFSEWYNVSLKSRKLIKFVMHRSLNPCQFTAGGLFVMNMENFGSIMKSSMSYVTVIASIR |
| 299782507 | NvOR160 | MSVYSSDYWKMPVLAQKFMGVWPFNNRQYDKCMRVFVYVALYSLIVPIGIRLVEELGVNTAIAIENLVGQMYLNAAVIKFSMTILFKEKHKQIYELIARDWKMTSDKEELEIMEKHAAIGRTISLAYGICCCSTAGAFLMIPTLLPLLDYVAPLDNSSRPVVLPYYAEYYIDQRKYYLPLMLKALVAGMISMTVFITYDMAFAMCVQHVCSLFDIINLRLQRASQLGSQLGSQLGRSARTSASYDSGVFRLIQKAIELHQIVIENVSSLENAYNLNWFFILLLNTTAVGGALLVVLLKLGHPEDLVRYGMFFAAIFIHKYFIFLPGQKIINYSLEVFEYSYSCEWYNLSAECKVLIKIMMLRSIRPLNLTGGKMFLLCMETYSAMLKAGMSYFTVFASTQSF |
| 299522827 | NvOR161 | MELFESNYWKLTVFLQKLIGLYYFQSLWKNIVAWIYVYTFTLSFIIAIGVRLYQEIGIDINIVTENLVAEMYLIIVFAKLTTSVVYMKDLKRLYKSIANDWRVMSDEKELKVMHEYTDIGRKSVQLYSGYMIIGIAIFLSLPISAPLWDYIVPLENATRPNALPYYAEYGVDQEKYYFPLMGQAVFGGIGTGMLLVTFDLGFILTVQHVVALFALVCYRLDQAANLSLSVERGKIDFIKADRSAYEYTVKAINLHQTVLGYVDLVENCYNAGWLVVLFMNMLLCGGGLAVLLMKTDRPEELLRYFTVLFAGFIHFYYIFLPGQKIINSSLEVFDKCYASRWYNLSEKSKSLIKIMMIRSLRRCELTGGKMFILCMDTYCNMMKTGLSVFTVLR |
| 299522829 | NvOR162 | MFDSKINTQDDFNLDIFETTDYKLYKDGMKLIGLWPFESSTKKTLKRAFVLISMISVLISIQIRFVEELNQNIDIVLQSAGSEILSIGCIAKFVTTLRAEDSFRVLFIQIAKQWASITDETECKILADNVKLCHPLCTFYRVMAVFALSSYACLPSFGPVIMNILLPLNETRQKRIPAPAEYFVDEEKYFYILFSHGMILYMLVCVLYVTIDSMYSCIVHHTVGLVGIVTYRLQNIIDLDITSSPKNHTNNLEIRRRLRRAITLHKESIEFAENIEATYSLCFIIVMFVNLFSMVFTAACGIRTLHYDKVESFRWLMLYGSIIFHLFFNSNPGQNLFDKTSEIINTLYFTAWYDSGISTSNKRIIQIMMIRCLRPCQLTAGGLLVLNMFNFGAIVKTSFSYITMLLSVG |
| 283135180 | NvOR163 | MFDSKINTQDDFNLDIFETTDYKLYKDGMKLIGLWPFESSTKKNIKRAFVLISMISVLIFIQIRFVEELNQNIDIALQSAGTEILSIGCIAKFVTTLRAEDSFRVLFIQIAKQWASITDETECKILADNVKLCHPLCTFYRVMAAFVLSSYACLPSFGPVIMNILLPLNETRQKRIPAPAEYFVDEEKYFYILFSHGMILYMLVSLLYVTIDSMYSCIVHHTVGLVGIVTYRLQNIIDLNITSSPKNHTNNLEIRRRLRRAITLHKESIEFAENIEATYSLCFIIVIFVNLFSMVFTAACGIRTLHYNKVESFRWLMLYGSIIFHLFFNSNPGQNLFDKTSEIINTLYFTAWYDSGISTSNKRIIQITMIRCLRPCQLTAGGLLVLNMFNFGVIVKTSFSYITMLLSVG |
| 299522831 | NvOR166 | MLSFKIQAKVNNGDVLGVGYWKLNKLMLKSVGLWPYQKSSTKMCIRTFIFIAIYSMMIPQIIRTFEEWGKNSEIVIENITGFLYFQVVITKYVTSCIAESNLQYLYVRITEDWNHFRDEGEQKVLSHFASHGRFLTIGYSVYLYTAGIAFTTLPCLIPAVLDLIIPLNDSRQKVLCFYGEYFIDQRVYYYELLLHTFVCVMCTIMLFTTIDAAYACCIEHVIGLFNIVDYRLNQAFNLVKDKYDTKSEVMRSEIHKCVLRSIEVHNHSIEIVELIQTTYTTCFFFTTGISLICLSLGTVDMMLSVNNYINFARVFFAWCGIVIYFFYISMPGQRIIDASSDIFNSVYFSGWYDFPLKTQRLLKFMMMRCSVPCQFTAGPLLVLNLENCGVILKTAMSYCTFVFAIS |
| 299522833 | NvOR167 | MEEHEILNNEYFKVNRFLLKLTGLWPYQKRHVKLIIRILYICAIHSMMIPQVIRTVEEWGKDFEIVLENIVGFIYLQCVLAKYIITFTAEPQLVFLYKKMAFDWTRYIEAEEQLSLQRAASNGQLMTIIYSVYVNFAGVGFATLPGTLPTILNIIAPLNESRPTKVLCFYAEYFIDQEEYYYQLLFQTFIGVMSTVFINATVDTLYVICAHHSDGLFNIVSYRFQKAFNKSQERYQVKSRNLVAAKNLDEEIHEYVLTAINIHNESIEFINLIQSTYTLYFFIQMSLTIISLSLATVVAMMNLHDIINLIRIFFIWCGIILNLAYISIAGQQIIDTSLQIFDSAYFCGWYNHPLKTQRLLKFIMLRCSRQCQITAGPMLVINLESCSNILKSSLSYCTFMIAVS |
| 283436095 | NvOR168 | MEHDVKKYFKYKRGIVFMLSASGVWPNYTSHPAAVRLFLNICSALASGCMFYCIVNFCLNYATNINAFTSCLGLMIGFFSTFIKVIILPMQKEDLQSLNEGVSASYERNLRIVKFRHHLLAHFPMFSRFFYLYSYSVGMSVLLLTIMPLLALRQGKYVRMYPQLVPFSYEPGGSLHWSIYAFEVFCGFYLWSVTSGVDSVFGLYALHMVGELRLLNVRFQMLKSSNNYAKDLKSCVDSHIMLMESRHKLQRIFGFLAIWLAITCAIALCALVFQALQAKHATIIRIIYLCGHCFLKLLQAYFYAWYGNIIAIESDACQSAIYESQWPGSGDKRFMNDVLVVLSQTPMIFKAKQWMPLRLDMFSKVVHTSVSYFFLLRTLDES |
| 299522836 | NvOR170 | MELKVERKAKSGLREEDIFNNKYFILNKKLLALVGLWPYQDARLKRVVRILLVLCIYSMMIPQMMKGIEECREKNPNPEIILENISGFFYFQGVTAKFLTAILTEDKLKYVYEEVMKDWKRFTDKNEIAILCKFAHVGRVLTVVWSIYAAMSCLLFVTLPAVIPMILNIILTRNETFKKSLCIYCEYYIDQDKYFFYIFLHHIIAGIATIFLTIGIDTSYVNCVQHVLALFNVSSYRLKVAFDTIHHSKKNDYNLKTLENNVHSYVVSSIRLHQRSIKFVDTIQSAYNIVFFIVCALLLFGISIITVDLVWNVHNPINLIRIACLWMGTIMYMFYSNWPGQKLIDSSNELFDAIYTCGWFEFPMKTKILIRFMLLRSIDPCRLTAGPLLQMNFESCSLILRSAMSYFTVLVTTG |
| 299522840 | NvOR173 | MDIFDGRYYKTSKWFLEFLGLWPFQSNRRRYVTCFIFVFMTATVVFPQVLLLIELKTSNFNILIENSLSIIFGFACLLKYGVTFASRSRLQTLLTQIASDWQRLTDKAEIDILSQYGEEGRYLVLFYTVYVFLAWVTCNFVPFIPPLLDILLPLENGTHDLVYPFYADYVFFKQTDYHYESCLHVFFVYFGTTSLFAGMDTIYVATVKHSCGLFAITWLETMARTGKSNRSNYSIKPNSVVHHDMVEAIVMHNETIRFVELLEDSFSLCFLMVQCMIVAGLATLCFYMMRIYDKTFNMCQFSTFTVGLVIHLLYLHWVGQKIIDSSDKVFYSTYYSDWYLISRNERQLTKIILARSLYPCQLTAGKISVLSMETFGALMKTSMSYCTVLLSVS |
| 299522844 | NvOR175 | MDIFDGAYYKSCKWFLSTLGLWPYQTDNRKRISAVIFFVVNISLAIPTLWLKSFTVTFENTVSVMFAIGCCAKYVVTYTSSQRVRNKADELVRLFKQIASDWQRITDTTELSILTKYSEKGKFLITFYQVYVWFGWTVYTLMPFIPYFLDKVSPLNESRPLLMPFYADYIVFDQADYHYTSCFHIAFVYISSALLFCGVDATFVMSVQHTCGLFAIICHRLEGEKIKKESEYAQNVMKTLSEEELREVVIFHNNCITCSGLLEDSFNLSFLILNSMSVLGLALSGVYFIYIYEDYYKFVRIMAFFVGLIIHLLYLNWVGQKIIDSSEDVFLAAYCSKWYVISTSARAFIKIIMVRALEPCRLTAGGLSTLCMESFGILIKTSVSYFTVFLSVA |
| 299522846 | NvOR177 | MDLFDGQYFKLNKIFLTICGLWPYQSKLRRRITFAMLASSTLLFIFTLVAGILSQSKFDFVNTEETFIFIFYCSAGLLKCTILYNQQNKIKKLYERIATDWKQLTDTSERDILRSFLLEGRKLNFIIMISCSSAFIIYSCVDLLPRILKEKSEYHRPHSFPYYFRPMVINEKLYDLQVAVHITVIVFYAGFAYMSAIATYISSVKHVCALYEIARYRLQNAVSYDKSNNSLLELVEDTSIVPKLVKVIDMHAQALRGIQIIEKVFSADFFVLEASSLTALATDVYELKYCRANVRTFIRAKLLTPIVIIYLFFVNCSGEQVIQACNDMRITAYYIDWYRTSSRARVFVLMIMRRTLNPKYLTAGTIVMIISIENFAAIITTAWSIGTILLTT |
| 299522848 | NvOR179 | MDSFDSQYFEINKRVLTICGLWPYQSKLGKRITFAMLASNTFLFNFTLIAGILTHSPGEFVNTAETLVGLFFCSTGLLKCAILYNQQNKIKKLYERIATDLKKLTDNSERGILRSFLLEGRRQNFLTMVYGVSAFIVCSCVEFLPRIFNEESEYHRPHSFPYYYRSMVIHEKFYDLQVAVHGTVIMLYSGLTYMSAIATYISSVKHVCALYEIARYRLQNAIIYDKHNYPLQELTDDTSTIPKLIKVINMHKRALRVTKKIEKVFSADFFVMEASCLIALASGIFELNYFRGNVRAIIRPLLVMPIVTIYLFFVNQSGQQVIQACNDIHTTAYNNDWYKASARVRIFVFMIMQRTLKPENLTAGSILILSIENFATILRAAWSFGTIMLTTLKHSPSRNEDA |
| 299782509 | NvOR180 | MDLFYNQYFNINKHVSMICGLWPFQSQFGRRISYMIFAMSTFSMIFSLTAGIISQLNPDLLNILETCVALFFCVCGFLVCTILYNQKNQIKRLYERIAADWENLTDDLERDVLRTFLLEGRKLIFITLVYSFPAFSLFACITFLPRMFSEESTKLCLHSFPYYLESMVIDKNLCNLQVSLHYSVALGYVGLSFLSVGATYICSVKHVCALYEIARLRLENATVRYGNYDPLGELTDETSIIHNLIEAIDMHKNALRGIQIIEQVFSTGFFIIQIFGLSLLAILICELKYHEGEITEMIRFMLVLSVFVIYLFFMNWSGEQVIQSCDDIQKTAYDIDWHRISSRTRIFVLMIMQRTLKPVHLTAGNMMILSIQNFGTILKSAWSFGTILLTTQKSV |
| 299522850 | NvOR181 | MDLFDSQYFKINKLALTVYGLWPYQSEIGRIINHVIFVVTSFSMIFAMAAGIQSQVNAELKNILETVVALVFCGAGLVKCTILYNQRNQIKKLYERIAADWEKLTDTSERDILRAFLLEGRHSIVITIVYAVPAFCLFICVEFLPRIFSKESAKHRLHSFPYYYKSMVISENTYDLQVCVHLMVVIIYVGFSYLCASATYISSVKHVCALYAIACQRLRNAIVYRKNSTPLKELIEDTSVIPNLIKVIEMHKEALRGIQIIEQVFSAGFFVFEISALTTIAILIFDLNYHQGNPFQMMRVLLILSVFVLYLFFMNWCGEQTIQSCNNVNAQAYNIEWYGISLKARVFVLMILRRTLKPIHLTAGTIMILSMENFGTILKTAWSFGMILLTTQTSARNKDPNFFGY |
| 299522852 | NvOR182 | MDLFDSQYFKINKRVLMICGLWPYQSILGRRIAFAMLANSIFLFVFTLIAGVISQSQLDIINTEDTIIITFFCLLGLLKCTMFYNQQNKIKNLYECIATDWNKLTDSSEHNILRSFLLDGRKINFITMVFCSSAFMIYSCIDFLLRIFNKESEYQRQHSFPYYFKPMVIYEKLYDWQVALHVTVIVIYSGLAYLSAIVTYISSVKHVCALYEIARHRLQNAIIACDKINHPLQKCIEDISLIPKLIKVIEMHEQAVRGIRIIKKVFGADFFVLTVFCISALTIGTFELNFCRADIHSFIRVLLLMPIIMIYLFYVNYSGEQVIQACDDMYTTAYNIDWYKTSSKTRIFVLMIMRRTLKSEYLTAGTMIMILSIKNFATIIKTAWSFGTLLLTTQKHRKNEDANFVAENTLI |
| 299522854 | NvOR183 | MTLINSHYFKLNKLLLTFCGLWPYQTKLKRRINYTTFAIITLSMIFSLAGGIQSELNTGFMNISESIIALLFFSTGFLKCTIFYNQRNQLKILYEQTAHDLKKMTHHLERDILQAFLLEARNFNVVSLVYSIPIYIVFAIATYLPQVFGFTNESAKYELHFFLYYKPMIIHESVQDLQVLIHATISTIYVGSAYLCVSATYISSVKHVCALFEIARYRLKNVIVDHSNNNRRGLMKNASVISNLIKVIDIHEKALRGVQRIDNVFNASLFILEVTALSAVTILIFHLNYHQGNFRQMTRYSTILSAFVSYLFFCNWFGEQVIQSCNDIRETAYNVNWYNMSLRARMFVLMIMQRTLKPVHLTAGTVVILSMENFSAFLKTAWSFGTLLLTTQKPSPKENSIFFEY |
| 299522856 | NvOR187 | MKLNGYDKFYYTLHKYVLTCIGLWPYQSRMSKRFFFITYGISSCSLIIALIAGLSEKWSTDPVIILENMLGIIFLTSSTAESSILYMHESKIIEFYDKIKTDWKKLTNKKEIEILQMHTRKGQFVSTAYILYGIPAFAIFGFVTFLPPILDPPSRVEYSHIFPYYFYCMIINEDFRYYQIVLHCMVSFSYASVSYLAVNCTFAKCVNHVCGIYAIICYRLQNAIEPTVVRGPFNKLKNSKLIRFNLIDVIAKHREVIHGVDMIEQIFSTGFLVIEIAGFSGIALVIADILYNQKNAYQLFRIMVVTAIFLVYIFYINWMGEQIIQVSDDVRLTAYFIDWFTLSIEAQEIIHMIVWRSCKTNKLTAGSFVALSLENFLSMLKTSWSVATVLLSAHRSQKNAHFTGYGITNSFTNSSST |
| 299522858 | NvOR188 | MNIFDFPQRHLLTCLGLWPYQSKFTQRIFFTCAILSFFSLFVAMAAGLGEEWSTELVIIYETIVALFVIFGGLAKCIVLFCRKHQMKSLYDQIRKDWQELTNEKEAAILQSFMLKGKAQIILYVVCAIPGYFIFVALTYVPIISSEDASKDYSHTFPYYTDLLILSKRFRVYQVFIHAGLGIFCGGITYVAFMAMYITCVRHVCALYAIVRYRLENMVKSQDKLMDKLNDDEEVIPGLLEIICTHKRAIKRVRLINRIFSRTFFMVEICLLICLALLIFDVKYNQHNVRLVIRMLMIALMFIVHVFCMNYCGEQVIQFSTDVQYAAYFMEWYMISSRAQKILIMILCRSSNPDYLTAGNMALSLKNFASIVRTSWSMATVLLTTQKVNRPSYSIS |
| 299522862 | NvOR189 | MEEPDLDIPYMKLQKLLMNCCGLWPYNSRLINRLIYSFFLLILISTIVPLGLGLIEEANNDIVTYFESLVSVVTMFGGVAQITMLRTIRNHLVKHLYKKITADWQTLKDAKEIKILSAFSFKGRSLTFLYMLITMSSYVIYLMLIYIPLANDKATSWDYSKIFPYYSKHWIISERVRHLQVTLHGCFGIFYGGVAYVVGMALYICCCKHVCGMYAIVGYRLKRLIISCEVTSSGKLRDDVLVYNLYAIMDQHKEAIKGVHLLARLFSHSFFLIQLCLLVCLSFLIFGVQYNIYSHKGMVRMFPASIIFVAHVFFMNYGGEQIIYYSSKIHTTTHFMQWYLLSVKSRRILLMLIRRSCKPEQLNAGTMTLSLVNFTSIIKASWSMGTVLVSAHRKH |
| 299782511 | NvOR190 | MPMEEPDSSIPYVKIQKFLMNCCGIWPYNSRLVNCLIYSFFVVFSFTTMTPLSLGLNEEANNDIVTYFETLVAVVAIFGGFAQITMLGIRNHLLKCLYRKISTDWRTLKDARETEILSAFSIEGRSLTFLFMLITISSYVFYLLLTYIPLINDEVASSDYSEIFPYYSNDWIISDRMRHLQVILHGCFGIFYGGAAYIIVMALYICSFKHICGMYAIVGYRLKRLVTSCKLTSSGELRDDDAVSKLYAIIDQHEEAIKGVRLLVRLFSRFFFIIELFLLICLAFLIFVLQYDIRSSKVIVRIFLASIILVTHVFFMNYGGEQIIHYSSKIHTTTHFMQWYLLSVKCRRILLMVIQRSCQSEQLSAGIMTLSLENFTSIIRASWSMGTVLMSAHRKE |
| 299522860 | NvOR191 | MDIFQSSYYIRCNRYLSFCGHWPYQSLRNRIRNFVLLMFLMSTILIPQIIKFWQLRHNIHVFVAALPSMLYYCAFLFKNSFSMLQSKEIKKVLEKIKSDFQRYKDEDLKILHKYSGQANKINTFYTVYMFMAVGGYSMLPLTLHVMDIALPKNESRLPTKPRLINYNIEAFDENIFFIIIHGVIVDTAVIVFIIGFETLCFSFSYHVCALFVIVTNKIRDSIDERITSKHSEVDQDIFYRNFVKIVIMHKDALDFVDTVETALSVLNLFAIGFAMMPLTITGFEFILSKGNVGEMARWSLFAFGEIVHLFYYNWPGQKIRDHSLCVYQSCYAIEWYKEEIPDKCKKLLNLMMLRGQKPCSLTAGKVYILGLENFAAVMKVSMSYFTVLSSVM |
| 299522864 | NvOR192 | MDILKSSYYIRCNKYLSFYGHWPYQNVIVKIRNQIVIMLLIMSIFLPQFMKMIEIRHYFHYFILSLPSLLYYTQFIAKNVFAFVGRKQIKNVLDKIQQDFQVYKGEDLAVLHEYSKKAQKFNKFYTVYMFMVVGAYSMLPFTLYMLDTFVPLNYSRLPYKPRLVKYCITTFDDNILFIIIHGGIADMMAIVFVIGFDTLFLSFAYHICALFVIVTHKIRDAVNDEIDSQNSTINECSNLREDISYRNFVKTITLHKYVLTFIDTIETAFSPLNVISIALAMVPLTITGFEVVMNKGNPGEMLRYAMYAIAEMIHLFYYNWPGQKIRDHSMLIYEACYATNWYRKDFSVRSKKIMNLMMIRSQKPSYLTAGKIYVLGLENFAAVMRVSMSYFTVLSSVT |
| 299522866 | NvOR193 | MEDNVLDGPYYVYCKNYLSSFGTWPLQSYKKKVLLRTLMYLGCSSALIPHVTKAYELRNHLEYFFLCIPSIIFYVQVLTKISCMILNEDKCKELIKQIKSDFQSYTGDNLRILNEYAEQARKVNHVYIYYFMGTVVVYNTSAFVPLLLDLLVPLNETRPRPVLRLMKYNIQRIENNFFVTTLHGFVLNILGMMLIMGFDTLLLNCSQHACALFQIVITELKDTIDKHKIEATSDTAKDTNSRDVFYQEVVKVIIKHKHAIEFVDLVESIYAMANLLVIGITLGSITLAEFETVQHKDNHEIAFRYAIFTSGELLHILFHNYPGQRIKDHSLMVYQSCYNCEWYREGITDECKKLLSFMMLRSQKPSCLTGGGLYVLGLENYATILKASLSYFTFLSSV |
| 299522868 | NvOR194 | MDIINGPDFVYSRICLRPFGLWPFQDPKSKLISRVITLMAVSTVLIPHIMKTYEFRNDFHILLMCIPSLLYYAHYITKFLYIAFREEKVFRNVLERIKDDFVTFRGESLNHLTNYSEEARKFNTFYMMYLCSTVVIYNVTAFIPHMLDFVFPLENATRPRHAARLVKYNIHQIDNNFYFVLIHGMIFDVVAIAIIIGFDALFINCAQHACALFKIVVVELRKSTKLDEKMSNSASDLVTLQCRQDIFYAKLVRTIIAHKHAIEFTDNLESTYALVNFLMIGIAVATITLTEFETIVHVNEVDIMCRFAFFSGGELISMLYQNWPGQRIKDHSLRVHASCFECEWYREDVSYKSKRLLMFMMLKSEVPSALTAGKLFILDLQNYVKIFKASLSYFAFLSSVAKVSSN |
| 299522870 | NvOR195 | MKMKDNVLDGPYYVYSKNNLSPFGMWPLQSYKKKVLLRTLIYLGCNSVFIPHVCKAYEVRNNFQYFFICIPSVIFYIHVMLKMACMILNEDKCKELFKQIKNDFEIYTGESLRILNKYAEQARKVNRVYVYYFMGTIVAYNTLAFMPLFLDFLVPLNETRPRIITKHMKYNIKRIENNYFVTTLHGYVFNILGMMVVVGFDSLLLSYTQHACALFQVVRNELKDTIDKHEIEVTSHAAKDANSRDVFYQEVVKVIIKHKHAIEFADLVESTYAVTNLLVIGITLGFITLVEFETVQHKDNRALGIRFAIITIVELLHILFHNYPGQRIKDHSLRVHQSCYDCEWYREGITDECKKLLSFMMLRSQKPSCLTGGGLYVLGMENYATILKASLSYFTFLSSV |
| 299522872 | NvOR196 | MAYAEEPRENILESSTFLYSKSSLRVFGLWPYQEPKQRLICRTSTAVLIGSLLIPTICIVLEQWRNFDYVVLGLPSFLYYVEFVTKYMYLAANQKKLEQIFGHIKNDFDTRKDRKLEILKDYATETRLFNHIYAAYLIIVVVMFNLSFYQPHFLDLIMPLNESRPRPIPRLARYYVTSLDESFNFVVLHGLVIDWYSMIFFLGHDTLLVNCAQHACALFKIVINDIQDCLVVQKNDKADDEDQFYRRISNTIDLHKWALEYTAMVDKMYMYVNLCIIGVSLLAITLSQYQTAIHLDNTDLVIRYSFFSIGELVHILYFNWPGQRIRDHSLSIYQACYNCEWYRDDISYRCKKLLKLMMARSQLPSNLSAGKLYVLGYENFAQVLKASLSFFTVLLSVN |
| 299782513 | NvOR198 | MDKERNMDRMNFYYIYSRISMLSLGLWPYQSWSSMMTLRSLWIIQHISIMLPELIKIYENRGHFNLLIESLPPFTYNIVMAIKFTNGVLNQRKLKSILEKIKYDWNKFTDKKEIEMLCYYSHRGKSLNTVYIGLVAVVLLSYMLLPMLPAVLDLINPLNESRPKSPLYMVEFYIDQDKYFYSVLTHAYITSLAGVLPLFATDLLFSNCAHHACGIIKILGRRIENILSEEPALKRSYKVDDEKKAIACVIEHQNIIKYCESINSLYTTSFFLILSISIGLMSVTGFVTLIKMNEEFKDCIRFAMFTFAQIFHQFCYYFLGQSVLNHEEKLKDYVSNFNWYKASPKTKFIIKFMIMRTLKPTKIRAMIFPLTLENFTSLMKTTMSYFTVIKSTR |
| 299522874 | NvOR201 | MDKERNMKFYFVYTRLSMLCLGIWPYQSWSSMLTLRSLWVIQHISILLPEGIKIYKNRKNLNSIIDGLPPFIYNVVIAIKFINGIINQHKIKSILEKIKNDWNQLSEKKEIEMLRDYSDTGKAFNTVYLSLVTVILLSYMLIPMLPAALDLVNPLNESRPTSPLYLVELYIDQDKYFYSVLTHAYITSLAGILPLFAIDSLFSSCAHHACGMIEILGGRLENIINEEASIKEIDNNEEEKNAIACVIEHRGVIKYCESINSLYSTSFFFVLSFAIVMMSVTGFVAVIKMGEEFKDSIRFAMFTFAQIFHMFCYYFLGEIVLHHEEKLKDYASNLNWYKASPKTKYIIKFMIMRALKPTTMRAIIFPLTLENFTTLMKTTMSYFTVIKSTR |
| 299522876 | NvOR202 | MEQHYSIRTYFKLNRVFMVSSGVWPYQPLHVARIIRLLWITQHISIMTPEIIKLIEVRGMADLLLECIPSVFYNIVIAVLYGTTIHHQRKIKELIEKIQKNWITISKKSEVEILTRYSNMGIRIGWLYIGALYFTLFIFCLFPLSPIVMDYVNPLNVSRQRLPLYRVQFFVDDKKYYWTILMHAYTTTMIGIIPLLTVDLFLANCTQHICGMMLILGKRLEKTMETTKLVVNKLDDNIYKDIRKCTILHTEILDFIEDINYIFSTAFGILLAVLTFLTSFTGIVVLIKWGDWNEVIRFGMFTMAELFHAFCYSYHSQDVIDHNNQIHKSIMNSGWYKSSMRTRVLVQMMFLRSNKPCLINCIIFPLSMENFTTILKTMFSYFTVVKSCRF |
| 299522878 | NvOR203 | MDGKQCLQTYFIVNKVFLFSCGTWPYQHTIFAKTFRYFWITQQIVIMAAKSIKLYEIKNDTDLVIEAVASFFYNISITIKFVNQVINEHKVKIILEKIQDDWKSLEDDSEIKILSYYARLGKLFNFMYIGAVYSALISYMVLPLTPIILDFIVPLNESRPKQPLIMAEFFIDQDKYFYPLMIHAYLSVLYGIIPLLGTDTLYMNCVHHSCGMLKILGNRIRNILNSSSRELSNKIKYEKMVKCIIQHQNIIEFCNNINETYSTSFLIVLCFSITLMSFSGVATVIKLGDNFNDVIRFGFFSVAQIFHLLCYNYMGQNVLNYGEELRAQIYNTNWYEASLKTQRLVKFMMAKNMHPIILRANIVPLCLPNFTRVIKTSMSYFTVLQSTR |
| 299522880 | NvOR204 | MEKENDCIRAYYKLNKFSMTLLGHWPYQSENSVKIVTFLWIFQHLSILLPELIKFVEIRNNVDYVILAFSPIIYNIVVGIKFVNGSLNRHKIKITLDTIQSDWKSLRTEEEARILANYSSFGKLCTVGWAWICTTTIICYLLFPFTPFVLDLIRPLNETRPRQLIYMVEFFIDEDKYFYEIQIHSYATTLIGFIPLISIDTFYAASVQHACGMFAILGHRLRRINGAMSKSKKRSDEDAYREIVSCAIQHDKILQYCDNLNDTYTDSFFYIMGCNMISLSFCGVLLILMWGRIYDMLRNGIFTFAQIFHLFYYSFQGQVLSDRSLMISDCVYDSEWYTASLRTRKIMTMVSMRSLKPFLLTAKVYVMSLPNFTLVIKTSMSYFTVLKSSR |
| 299522882 | NvOR207 | MIFSFKDKFFPYYAWQTVTVLEKAAGWWPFQNRTKNMTLRLMHFGNLLLIIIMCSVRLFQEYKQKKLYIVVENTVILVMIICTKMKVIMFFINEKQRKIFYENVLIHWKDTNDEEEMMILKQYAKLGLKTIINYAISVNILFQGVPVLSDVINYLNDPNITYLKKELPIYIEVYIDQEKYFYQLYVVLFFMTCAALLLAFSHELTFFQSVQHINAMFKIIEIRIIRLSKIVKRTECGLDTFKEADRKIFVCISRAVDLHNAVLNNIKFINSSFGATYLVVLLFNCLIFGASLFLIFNNSDQKIHLIRYGLVFLGLSVHFFIIFWPGQKIMDGSESLFNVCCSCDWYKLSKRSKNLLRIMMLKSVMQCQITASGMFVLSFETYVKLFKTGLSFVAVFS |
| 299782515 | NvOR216 | MTISETDLAVFDGPHYSLNKKLLIMFGLWPTLSRTRKVICLIFFTMIDLSLYASFADGINYYRKQKKWMYVIEDTISIIYLSVTWIKYVTSYIFESRIKLIYEQIAADWKSLIDEEEIQILNNYSAFARLLTVLYVFYAIIATTLFYVLPFLTIVIDRIKPLENGTRFRAQPYHQHYFDLIDNEKYYYHMYIGHGYVVSIIVTVAVMAIDTMYAANIQHACGLFAIVRHRLSKIGILNGEREYEFRVVDDKKVYESIRAVCEMHKNSIKIVELIWDSFSISFLIFMGCSLVGMGMLMFNYIFNMIHPIEKVVGTGLLLGIAILLFYMNWIAQQLTNSSDEIFIAVYSNRWYNLSIKGQKLIYSLLQSNANSVTLRAGGIAEMNLQQFAAILKTAMSYATVMMSMNG |
| 299522884 | NvOR217 | MGIPDPENPLAIFYTDYYKYNRKLLEICGLWPELSRPRKIIMMILFALLMTSLIIPMGAGAIHYFHKGRIMYVVEDLIGLLYLTVASSKYFTYSVFEGRILRLYHQVGEDWRTTTDEEERKILQEYSEFARLLSIIYFVYAAIGAFYFNMSPYLPLGLDRWMPLESNESRVRIRTYHPYYFDLIDAEKYYYECYFFHGNSVVYVSTMVGLSVDSMTAFNVQHICALFHIVGHRLRKIGSTLEINAKGEKIARVDDITVVRQIKHVCGMHRTSIDSVELLQSSFGMNWLVFLIGTVTGIALLMFDLIFSMKHPLEKMTGLVIFIGIQILIFYINWIAQKLTDSTEQIFLAACETCWYNLSVKGQKLVYFMMQKNIIPLTLTAGGIAELNFQQFASVSKTSMSYAMVILQMNDE |
| 299522886 | NvOR218 | MPVETKDQRKSYDFLSISSSHLTFMRLSSFLPLKGKSFFHPLSLLLQLWDHFIVIGYNVMWQGYGIRMIQRGDVEVDFICEDIITVGFTIRYLLLCAKREKLCCLVESCEKLWDLLKDGEVIFVRQFARKGYYFRNFILINAMLMAALYSVTAPFVRLPPIEANGTERKILPFRFFMDIQKEPAYSIVFAFQSILLQFIDLMIVSTETVSLYLIMMACGYLRSVRNRLLSFKGNDDNTSEKGEAALKFVIDCAHFHQQIMIFCEDIERMTRTLFFFACFCPIYNVSITGIVLFNNDEDKYKFLPLLCYNFFQFFLCQWAPEHLAVESEDIALAAYSASLRPQAPSHREKINRILYFMMMRAQKPVQLTAGGFVDLSIETFGAMTKSAFSFFMVLRKFRS |
| 299522890 | NvOR219 | MSTKAEDSHTFQSISASHLNYLRLCSLLPWQGKGFSHPFSLLFQLWNHLAIFGFNAMWHGYGIRMLQRGDVEIDLICEEITVLDITARYFLLLIKREKLGRHIETCRKLWSYLKAGEDMFVSQFERKGYYLRNFVMINSVMVTTAFIITATFVRLPPLEANGTERRMLPARFFMDVQEDPAYSIVFASQSILLLSVDVMIGSTQTVSLYPIMMACGYLRSVRNRLLSLEGSDNGTDAKGEATFKFVVDCAHFHQQIIIFCEDIERMTRMLFFFACFCPIYNVSIAGIVILNSNEDKIKFVLLLVYNFFLFFLCQWAPEHLTVESRAIAEAAYFASLQPLASSYREKINWILYFMVVRAQKPIQLTAGGFAPLSIQTFGAMTKSAFSFFMVLRNFRT |
| 299522888 | NvOR221 | MRGAMSAEPKDLRESFTFLSISSSHLIFMRMFSYLPLKGKSFSHPLSRLLQLWNHFAVFGFNAMWQGYGIRMIQRGDVEVDFLCEDIITIGFTIRYIVMRIKREQLCRLVESCEKLWDLLEDGEAVFVRKFERKGYYFRTFILCNALLMAGSYSIAAPFVRLPPLEANGTERKILPFRFFMDVQEEPAYSIVFVLQSIALQFLDFMMVMTETISLYLIMMACGYLRSVRNRLLNLKGSDSDPSEKGEAALKAVVGCAHFHQQIMIYCEDLSKMTETLFLISCFCPIYNVSVTCLVILNTEEDNLKFVPLMLYNFFQFFLCQWAPEHLTVESDNIAEAAYFASLQPQAPSHREKINRILYYMMMRAQKPVQLTAGGFAPLSIKTFGAMTKNAFSFFMVLRNFKN |
| 299522892 | NvOR222 | MTTKTKDGYTFLSISSSHLIFLRLSSFLPLKDKSFSHPLSILLQLWDHFVVMVYNSMWTGYGIRMILRGEMEIDFICENVVVMGFTVWYIVIQMKRQQFCSLVKFCEKLWSYLEVGEEVVVRQFERKGHYFRNFMLFNLLLMCTLFITTAHFIKLPPLEANGTERKILPFRFFMDVQEEPAYSAMYTLQFFVCYFVVFMIASAETVSLYLIMMACGYLRSVRNRLLSLEGNDDDTGEKGEAAFKLVVGCAYFHQQILIFCKDIERMTRTLFLFACFCPIYNASITGIVLLNNDEDKFKFILNLFYNFFQFFLCQWAPEYLSEESEVIAEAAYFASLQPLASSHRQKINRILFFMMMRAQKPVQLTAGGFVKLSIETFGAMSKNAFSFFMVLQNFRS |
| 299522896 | NvOR224 | MPGAMSAETKNTFLSISSSHLIFLRLASFLPLRSKSFSHPLSLLLQLWDHWSVLAGNMMWSGYGIRMTLRGEMEIDFICEDIIMVGFTMRYILLATKRKKLCHLVESCEKLWDYLEIGEDALVRQFERRGYYYRNFMMLNLLLMCTLYIVTAHFATLPPLEANGTERRMLPFKFFMDVQEEPAYSIAFVSQSVVTYFICFMFVSTETVPLYLILMACGYLRSVRDRLLSIEGSDDDTSERGEVAFKFVAGCAHFHQQIMIFCEDIKHTMRTIFLFACFCPIYNLSITGIKLLENDEDKFKFIVILVYNFFQFFLCQWAPEYLIEESEDIAAAAYSASLQPQALSHREKINGILYFMMMRAQKPMQLTAGGFVRLSVETFGAMTKNAFSFFMVLRNFSS |
| 299522894 | NvOR225 | MSTEMEDPHESYTFLSISSSHLIFMQLSSFLPLKNKRFTHPLSLLLQLWGHFVVFASNVFWTGYGIHMVMHGEVEVDFICEEIVVLDFTARYILLIVNREQLCCLVKSCGRLCSYLEAGEDIFVRQFERKVYYFRNFVIINSLLVSTVFDVTAYFTRLPALEANGTERRMLPARFFMDVQEQPAYVVTFVMQVILDYYLDFLIASTGAAPFYLIMMACGYLRSLRNRLLNFKGGDYDTSEQGEAALDTVIGCAHFHQQMMIFCKNIERMTQTLFLFACFCPIYNVSITGIAILNSDEDILKFTPLLVYNFIQFFICQWASEYLAEESEAIAEAAYFASLQPQVPSHRERINRILYFMMMRAQKPVQLTAGGLVNLSIQTFGAMTKSAFSFFMVLRD |
| 299522900 | NvOR226 | MSAKKKVQKEGDTFLSLSWSHILFLRVASFLPLKGKSFSHPLSLLLQLWDHINVIAFTSLWQGYGYRMIKRGEMEIDFICENIITIGFTLRYIILCLNRELLCHLVESCEKLWDLLEDGETVFVRQFERKGYNFRNFFFGNLMFMATLYTITAAFVKLPPMEPNGTETRMLPFRFFMDVQENPGYAAAFVFQDVVVFYTDVIFASAETVPLYLVLMACGYLRAVRNRLLKIEGNDNDSSEKGEAALKVVVGCAHFHQQIMIYCEEIGQMTKTLFLVSCFAPIYNVSIAGIKLLENDEDKFKFIVILVYNYFQFFICQWAPEYLTEESESIAVAAYSASLRPQAPSHRQKINGILYFMIMRAQKPVQLTAGGFVNLSIQTFGAMTKSAFSFFTVLRNFSG |
| 299522898 | NvOR227 | MPTGMDSLTTTFQSISSSHLMFLQLSLFLPLESKSFSQRLISNLLQLWNHLMVIVYNVSYAGYGIGMALRRDIEIDYICEQIVVETFSARYILLCFKRAQLRRLIESCKRLWGYLEVGEDIVVRQFERKGHFFRHFLILSSLMAVTSYVVTAHFLRLPPLEANGTERKMLPFRFFMDVQEGPAFNAMYALQIINSYYLVFMFASVETVSLYLIMMACGYLRSLQDRLLSLITEMNEDDLSKNGEATFNVVMGCAHFHQKIMIFCKDVDQMTRTLFLFACFCPIYNMSITGIKLLESDEDKFKYASLLFVNLFQFFSCQWAPEFLIIESEAIATAAYFASLQPFAPSHREKINRILYFMMMRAQKPIQLTAGGFIKLSIETFGAMVKSAFSFFAVLRSFRT |
| 299522902 | NvOR229 | MLIEKKIESFTFLSISSSHLTFLRMAAFLPLDNRSFHHPYSRLLQLYGHICIFIYNTMWTGYGYRMISRREFEIDYICEQMVVEGVCLRYIVLCAKREQLCALVESCKRLWSYLRSGEDVIVRQFERKAYFFRNFMLINSILVVMLFIGTACFVRLPPLEVIGTERKVLPFRFYVDVQEDPMFSAVYALQAVVCTTISFVIASIETVSLYLIMMACGYLRSLRNRLLSLAENEDDAILAGETSFRLVVGCAHFHQQIMIFCEEVDRMTRTLFLFACFCTIYNMSITGIKLLENDENKFKFGAILSLNLFQFFTCQWAPEFLIIESEAIGKAAYFASLQPMASSHRERINRILYFIMMRAQKPIQLTAGGFIKLSIQTFGAMVKSAFSFFAVLRSFSST |
| 299522904 | NvOR230 | MEREPIKYEDISRLYYRLFRTMGILPSSSSRRTTLLRVYFHVTIVLYYSMSMFDGLRMLGHNDIEIEYVFEEVVIHGICARFLILSCRREELAELLLSCEKLWRMLKPGEDRVVKSYEKIARYLAHYITWTTLVAIFFYIVAARIVKLPPAEVNGTERRMLPFRFYVDVQRQPWYDIVTVLEIVVVLNIAMIVSTIETTGPFLITMACGYLRSIRNRLLAIADEAEGRGEISRLSTIRVVSCVKFHQKIMRFCQDIEKLTSSVLLVQVVCTAYNISLVGFRILKNDPNAVKFVPLLLLNLLQLFTAQWIPEHLLSESKAIANAAYSASLLHPEYEPRANRALLFVMLRANRPVQITAGGYMKLSLETFKRMLTSALSFFTVLRSINDGAGDEGE |
| 299522906 | NvOR232 | MRLHEINSFERVPASGTIRKFVEFRETDGSLRIFSPPHRGFTFGEPRLQLSLLQDASHLLRVYWPTQQCSGVVEVDSIFVMVMAVSTIMRYYILVYHRFDFRDTMDACRVIWNDCTPNEHQIVRWFERKTWMLFKLLAGSGMFINVFCSIGSIVVRLPPDEPNGTERRLLPYRWFIEDREYHWLGYELIFGLQVLITHHLTVIAATVDTAGPLLMMISCGFFKALQERFFAAAARNEMILCKDKLEFKQTIVSCSKFHQSVLVLCKKIEVMTRMIFMVQLICLGYNISLIGLKLTGTDPERFQYIPNLVLCLCQLFITQWASDYLLEQSEEVATAAYFATLMSLDARIGGLLLTMVIRAQKPVQMTAGGVIKLSIERFGSLITNAISFFMVLRNFTTQV |
| 299522908 | NvOR233 | MRVHEINSFERALASDEIRKVAEFQESDGGLRIFPSTHRGVTFGKPRLQLSQLPDASHLLHVHRTPHQRHGAVELDSVFVTVMATSTIVRYYILVYHRFEFRDAIDACRDIWEDCTPSEHQIVRWFERKSWILFKLLAGSGLLINIFCSIGSIVVRLPPDEPNGTERRMLPYKWFIEDREYYWMGYELIFGLQVLILHHLTVLTATVDTAGPLLMLLSCGFLKALQERFFAAAVSNEKFFIEDKLSYQPLLTSCSKFHQNVLNLCRKIEVIMRMIFMVQLMCLGYNISLIGLKLAGNDPERFQYIPNLVLCLCQLFITQWAADYLLEQSEGVATAAYFTTLMSLDPRIGGLLLTVITRAQKPVQITAGGVINLSVERFGNLITNAISFFMVLRSFTA |
| 299522910 | NvOR236 | MEETSAFYRRIRRIQTRVLRLAGLVPFENRTLIFAGTILMSIYVNFAFTAVSSVYIWAFFEDCLNKRFNPDITSELFSFVGFHFRFMYIFSRRRKLGEMLGYAESLWERVRSEEKVHVRLFVRKVSKLSVCYSGIILTTITLYVLSSQLPQLTAAATNETVHRVLPYPFYVDVQSSPRYEILLGAQIVCLLTVTQTSVCVDTAIAFLIMIACGHFRLIQVRLGVIARHIEENEDKRKSQRSVGKNGEVIEAEAEMDEEDFERTDDRVRERVKELVMHHQEILSFCDDIKNLSSEIFMIELISTTYNLSLIGILLAGNMPLAEKFKFAPVLFILTTQLFVCQYPPDLLIQESEAVANAAYFVPPFRRDRRRIDRILLSLLTRSQTPYQLRAGGQIPLSIESFGNMIRGAVSFFTVLRSFN |
| 299782517 | NvOR241 | MELELLRYEAYTHNVIWFLKSAGLWPEAHPVPRKILSMVTLCSTFVVMVTVSNFSFQNVGNVMVLTRGMSLAVSFSSAFSKVALFLLHHDDLVYLNKHLTGGFMRDMKEPENRPDLLNNVKTFNRFMFTHAISVAIAMSMYSIGPLLALRKHGKYIRAFPAIYPFAYESGGLVHWILYALEVSGAASLWTVTVGVDCVFGLYALQVCGELRILAKKFRELRATENYREKLHDCIQRHHVLINAKNKLDNIFGLISIWLAISGALVLCSLIFQVTELLKTNNSYLRAAHLCAYLLPKFLQIFTYAWYGNLIAEESGACLDAMYGSHWTDSCDKNFKSDILIVLAQEPLALVAMGCMVIQLDMFTKIVKTSVSYFFLLRTLNEENE |
| 644995932 | NvOR242 | MIMEKEVEKYKKYKSNLKFMIVSNGVWPDYEKHPYCVRKFLNFCSISSISMTNYCMMLFVIATTTDVRSFTSFFGLLLGGFGNLFKVCALTMNQKELHALNEGISASFERNLRVPENRPHLLANFPMFSKFFNFLSYSTLGTIGFLTVIPLLHLRHGTYSRMWPILLPFSYEPGGTIHWIIFVFELVVSFFAWITTCGVDCLFGLYSLHIVGEMRLLSSRFQKLEWSENYRKDIRSCVKSHLLLLKTLSQMQEAFGDLAVWFAFNSAASLCTLVFQFSQLTVMNPARVLYLLCHTCIKLVQAYSYSWYGNIITVESEVCLNAAYNSHWPNHGDKHFMRDVLIILLQRPMVFKAKSFIALRLDLFARIANTTLSYFFLLQTLDEKV |
| 283436101 | NvOR243 | MELELLRYRAYTHNVIWFLKSAGLWPEGHPVSRKIRSMVTLFSTFVVMVTVSNFSFQNVSNVMVLTRGMSLAVSFSSAFSKVALFLLNYEDLVYLNEHLTGIFERDMKKPEYRPDLLKNVKTFHRFMYTHVASLTFTLIMYVIGPLLALRKHGKYVRVFPAIYPFAYEPGGLVHWILYILEVLGATCLWSVTSGVDCVFGVYALQVCGELRILAKKFKELGAIENYREKLNDCIRRHHVLIKAKNKLDNIFGLISIWLAISGALVLCSLIFQITELIKAKSSYLRVVHLSVYLLPKFLQIFSYAWYGNLIAEESTGCLEAMYDSHWTDSLDKNFKSDILIVLVQEPLTLIAMGCMVIQLDMFTKIVKTSVSYFFLLRTLNEK |
| 299522914 | NvOR245 | MDQKRFKYKAYERNVIWLLKSAGLWPEAHPVPRKILSLVTLFTSFVVMVTATNYSFQNVGNVRMLTKGMSLAVSFSSVFSKIAFFILHQEDLLYLNKHLTGGFMRDMKRPENGPALLSNVKTFNRFLYMHAVSVAIAMIMYSITPLLVLRKHGKYIRTFPSIYPFAYELGGLVHWIIYAVEVSAAATLVTVSAGVDNLFGFYALQMCGELRMLAHRFRDLRAGNNYKDNLKDCIERHQVLINAKNKLEDIFGLITIWLAISGSLVLCSLIFQVSELIKNHVSYLRIAHVCAYLLPKFLQIFLYAWCGNLIAEESKICLYAMYDSHWPDSHNTNSKRDILIVMSQEPLSVVAMGCMVIQLDMFAKIVKTSVSYFFLLRTLSAENE |
| 283436097 | NvOR246 | MEHDVKKYFKYKRGIVFMLSASGVWPNYTSHPAAVRLFLNICSALASGCMFYCIVNFCLNYATNINAFTSCLGLMIGFFSTFIKVIILPMQKEDLQSLNEGVSASYERNLRIVKFRHHLLAHFPMFSRFFYLYSYSVGMSVLLLTIMPLLALRQGKYVRMYPQLVPFSYEPGGSLHWSIYAFEVFCGFYLWSVTSGVDSVFGLYALHMVGELRLLNVRFQMLKSSNNYAKDLKSCVDSHIMLMESRHKLQRIFGFLAIWLAITCAIALCALVFQALQAKHATIIRIIYLCGHCFLKLLQAYFYAWYGNIIAIESDACQSAIYESQWPGSGDKRFMNDVLVVLSQTPMIFKAKQWMPLRLDMFSKVVHTSVSYFFLLRTLDES |
| 299522916 | NvOR247 | MELELAKYKSYARHVITRLIFAGIWPESNKTIKTILYFISFTSTLTVSVTSINFGIQNANNVILLTKGIGLASAFSSVFSKALLLPLHQEDIIFLKNRLTTKFMSDMETIEYRADLLSSVHVFSAFFNMHEAMVAFAMFMYCFVPLYVLFKHGTYLRTYPCLYPFSYTPGGLVHWLIYALEVAGAISVWTITVGADCGFLMYALELCGEFKILARKFTELKAGDGYKRNLKECIERHHLIIEAKNRLEDSYGLIVIWLALSGAFLLCSLIFQITELYDNHGSYVRIAHLCSHLVAKNLQIFMYAWYGNLIADESKAFLNAMYDSHWPEACDKNFKNDILIVLTQEPLVVVAKGCMYVQLDMFTKIVKTSMSYFFLIQTLAN |
| 299522918 | NvOR248 | MMDNEVASYVKYSSYLKRLTAFIGLWPDYQKQMPAISLLLSIQAAFSSFTTFCFIAYSCYLDSADIGAFTSYIGGLVGYLTTVMKIFVLGIQQKNLKKLNNGISASFEANLKVPENRQYLLAHLPMSLRFFYTYAITTGSSLALLVLIPLLLLRHGVYVRMLPLTLPFSYKPGGMVHWMFYLYEILCGWNLWTVAVGTDNLFSLYCLHIVGELKLLSSRFRNLKSSKNYRKDMKDCIQSHMLLMKTFLKLQKVFGFVVMWFAITCALCLCSLVFQAVEMDKVSVMRVFYLFNHSFVKLLQAYLYTWCGNIITVESEICLNAAYEAHWSDSGDKRFMKDILTVVLQRPMVFKANKFMELRMELFLKIVNTSVSYFFLLRTLDDDS |
| 283436099 | NvOR249 | MESKVARYAKYKRDIKCLIVASGIWPHYEKHPHVLRKLLSFCSAFCSGSTFYCIVAFCFKYATNINIFTSCLGLMIGFFTTFIKIVILSMRQEDLQSLNEGVSKSFENNLKLPENQPHLLYHFPSFSRFFYLYAYVVGISFVFLASTPLSIMLRYGKYVRMYPQLMPFAYEPGGSVHWAVFGFEMFTGFYLWSVTIGVDSIFGLYALHMVGQLRLLGSRFQNLKSSSNYDKELGECVRSHIQLMKSRHKLQRVFGFLAIWLAVTCAIALCSQVFQALHMRNTTPVRALYLFGHWFIKIVQAYSYSWYGNIIAVESDLCLNSMYYSHWPGSGDKRFMADVLIILSQKPLVFKAKQLMELRLDMFLKIVHTSLSYFFLLRTLDENPKAGT |
| 299522920 | NvOR250 | MELELAKYKSYARHVIFRLIFAGLWPESNPKIKRMLSFVTFTSTLTVMVTAINFGIHNASNVILLTKGIGLASGFSSVFSKALMLPLHQEDIVFLKKRLTSKFMSDMETIEYRADLLSSVHVFSAFVNMHEAMMAFAMSMYCFVPLYVLFKHGTYMRTYPCLYPFSYTPGGLAHWLIYALEVAGAISVWTITIGADCGFVMYALELCGEFKILARKFTELKAGDDYKKKLKECIERHHLIIEAKNRLEDAYGIIAIWLALSGAFLLCSLIFQITELYDNNGSYVRIAHLCSHLLAKYLQIFMYAWYGNLIADESKAFLDAMYGSHWTEACDKRFKNDILIVLTQEPLIVVAKGCMYVQLDMFTKIVKTAMSYFFLIQTLAS |
| 299522922 | NvOR251 | MERDIQTYKVCSENVTLCLIFSGVWSATHPVLKKIAFFVTFFSTFSIMAHTLNFSLHNAQNVRILVRGLAAASSFLSISSKAFLFLQHQNDLNYLKDYLTEKFMSDMKNPENLPDLLSNMRMFAVFVTMYKTTIAFIMSMYCIVPLFSFLKYGKYLRVYPCLYPFSFVPGGVVHWLLYGWESTGALSAWAISVGTDCAFGMYAIQICGEQRVLARKLKDLRVGSNYTRELRDCMERHHLIITAKNTFESLYGLISIWLAISGAIVLCSLIFQVTEYLENRGGYVRAIIFFAHFSGKMMQVFMYAWYGNLINEESLAFPRAIYSSHWTDCCDTRFKNDILIVLAQRPLIVTALGCMNVQLDMFAKIVKTSISYFFLLQTLKAKTEEK |
| 644995937 | NvOR252 | MEEDIQTYKVCLQNVVICLIFSGVWPATRPLLKRIAFFVTFFSTFSIMAHTLNFSLHNAQNVRILVRGLAAASSFLSISSKVFLFFQHQDDLVYLNDYLSKKFMSDMQNPENLPDLLSNVRTFAVFVRMYKTTAAFIASMYSVVPIIAFLKYGKYLRVYPCLYPFSYAPGGVVHWLLYGWESAGALSAWAITVGTDCIFGMYAIQICGEQRILARKLKDLRVGSNYKKQLRDCMERHHFIITVKNKFEDLYGLISIWLAISGAIVLCSLIFQVTEYLENDGGHVRAIIFFAHFSSKMMQVFMYAWYGNLINEESLAFPRAIYSSHWTDCCDTRFKNDILIVLAQRPLIVTALGCMNVQLDMFAKIVQSSISYFFLLQTLKAKGEEK |
| 283436209 | NvOR253 | MELEMAKYNSYSNTIIWSLICSGLWPKGHYVLKKILSCISFLSITTIMTTAINFSFQNARNVQLMTKGMGTAVSFSSVFSKIVMVLYHQNDFIYLKKHLTTRFKRDLEQTENRQDLLFNVHIFTKFVNTHEASMAFAMFMYCIGPILALYRHGKYVRTFPCLYPFHYEPGDVVHWVIYGLEVTGATVIWFITIGVDCGFCMYALELCGEFKVLGRKFRELRVADDYKEKLRDCIERHHLIINAKNRLEDAFGIMAIWLALSGAFLLCSLIFQITEILENHGSYLKIAHLCSHLLAKYLQIFMYAWYGNLIADESQSFLYSMYSSHWIDACDKRFKSDILIVLVQEPLMLVAKGCMNIQLDMFLKIVKTAMSYFFLLQTISSEE |
| 299522926 | NvOR255 | MEVELKKYKRYYRDIKLLLVVSGIWPNFYPILDRVVSIVAAISTLLLTMALLNFCAHHVANIMILTKSMGIAISFFSSFLKICIFLSHHDDLVYLNDYLTSSHTSDLSNPDDRSHLLEKFSSFSKFFYTLTIAVALTFVLNTIAPFFALKRGKYLHIYPVIFPFDYEPGGSVYWSLISLELTAGFFVWSVTSGVDSVFGLYALQMCGELRVLAKRFEELRATGDYRMRMRECMDRHHLLMRSRDILEKVFGFLAIWLAVTSALVQCSLVFQAKVEFKTLSPFKIGFFFFYILMKLVQAFTYAWYGNLIAEESALCLNAMYNAHWPGSGDVRFMNDVLIVLSQKPLIFKAKSCMSLHMDVFTKIMNTAVSYFFLLQTLDEGSVRHL |
| 299522928 | NvOR256 | METELRKYERYSRDLKCLLVLSGIWPDFHPIIQPLLGCFAAFVCFVTVIAFLNFSIHHITNVVVLTKSFGLVISFFSSFLKICVFLWHHDDLVYLKAALTDRFNTDNLNKSFRRFTLAKVNVFANLFYILTIAVGLTTGMAVVLLIISLRHGKYVMLYPSIFPFSYEPGSRVYWILLMVELFANLFVWAVTSGVDSVFGWYTLQICGEFRVLAHKFQNLKSSENYRDDLKECVERHYVLMKTRDVLQDVFGFLTILLALTSAIVQCMLVFQAIQVFKNLSLGMMVFLIAYITLKVVQAFIYAWYGQLIAEESEVCLGAIYNARWAGSGDTRFMSDVVIVLSQKPLIFRANGCMSLKMDIFIKILNTSVSYFFLLQTLDEGSEHHQH |
| 299522930 | NvOR257 | METELRKYERYSRDLKWLLVLSGVWPDFHPVIQPMLGCFAVLVCSLTAIAVLNFSIHHITNFVVMTKSCSIAIGLCLSTLKLCACLWHHDDLVYLNTSLAASFNADNQNKSFRRFTLAKVNVFANLFYILTIAVGLVIVMGLVFMILSLLHGKYVLVWPSIFPFSYEPGGWVYWILLTVQLLANFFAWTVPSGVDSVFGWYTLQICGEFRVLAHKFQNLKISENYQDDLKECLERHYALMKSGEVLQDVFGFLAILVGLSSAIIQCMLIFQAIQVFQQLSFGMMILIFAFITLKHVQVFIYAWYGQLIADESEDCIEAMYNAQWAGSGNIRFMRDVLVVLSQKPMIFRAKGCMLLKMDMFIKVLNTSVSYFFLLQTLDEGLQN |
| 299782520 | NvOR258 | METELRKYARYSRALKCLLVLSGVWPDFHPVIQPMLGCFAVFVCSLTAMATLNFSIHHITNFVVMTKSLTIAIGLCLSTIKIVVCLRHHDGLVYLNSSLTASFDADNQNKSFRRFTLAKVNIFANLFYTLTIAVGLATGMGVVFLILALLHGKYVMVWPSIFPFSYEPGGRVYWILLLVELSANIFAWAVPSGVDSVFGWYTMQICGEFRVLAHKFQNLKTSENYQDDLKECLERHYALMKSGEVLQDVFGFLAIMVAVTSAVIQCMLIFQAIQVFQQLSFGMMILIFAFITLKHVQAFIYAWYGQLIADESEDCIEAMYCAQWAGSGDIRFMSDVLMVLSQKPMVFRAKGCMSLKMDMFIKVLNTSVSYYFLLQTLDEGLQS |
| 299522932 | NvOR260 | MELEVRKYENYSRDIKRLLIVSGIWPNFYPVLQRFVAVLAICCTAMTFMGAFNFCLEHVSNVVVLTRGMGLLFTLLSTGMKICVFLHHQKDLIHLNQHLSARFLDDLQNKAYQSHVLARLPAFSELFYSLTYTIGSTAFLTTILIPLLALRHGKYIQVCPSIFPFEYAPGGLVYWLLQLTEALAAFFVWAVTSGVDSAFGLYTLQMCGELRILGSKFESLRVSDKYREELRECIERHHLLMKARDSMEKTFGLLAIWLAVSSAVIQCTLVFQAMEVAKSMNPLRIGFFFLYIVLKLLQAFMYAWYGNLIAEESAMCLNAIYNARWAGCGNSRFMTDVLIILSQKPLVFTAKGCVSLKMEIFSKIVNTSVSYFFLLRTLDEGSQN |
| 299522934 | NvOR261 | MEAKLAKYARYRNVVRRLLLLSGIWPHLEDTCRLYRVLTFSATFVIAALGAKVFAYCIDNIAHVSLFAKGMSNAFSFYTSVLCYLVYRKDLVMLNDCLGRRFEDELKREDRRPLLLQSISVYTRFMCIVAGLTATALVFYTLVPLVFIFKYKKLTQIYQGRYPFAVEPGGRVYWCVCFVESISVVFVWNVVCSVDNAFGLHSFRMCGLLRSLADRFAKLQPDDPGYIVELRDCVRTHQLVLRAKEALQRVYGLVVLWTYVTSAIIMCSILYQADQAKKHMTVTRVIFFTSYITLKLLQSFTYAYYGSLVSQESEKCQNAIYTSNWPGSGDLRLMKDVLIIQSQRPIVLRANGFFIVSMEMFEKIVNTTISYFFLLQAVEEK |
| 283945544 | NvOR262 | MMMELELLRYKAYTQNVIWFLKLAGLWPESHPVPKKILSTITLSSILVIVLTVSNFSFHNLGNIMVFTSGMCMAASSTSAFSKVALFLLHREDVVYLNKHLSGGFMRDMDEPDNRPDLLSNVKTFERFMVTHVISVAIAMFTYSVRPLLVLRKHGKYIRSFPAVYPFAYEPGGLVHWILYAVEVSGTASLWTVTIGVDCVFGVYALQVCGELRILSRKFRELRADDNYKEKLKDCIRRHHVLINAKNKLENIYGLISILLITSTTLVLCSLVFQVSELMKTNSYLRTAHLCVYLIPKFLQIFTYAWYGNLIAEESGACLDAMYGSHWTDSCDKNFKNDILIVLAQEPLALVAMGCMVLQLDLFAKTVKTAVSYFFLLRTMNEGSE |
| 283945546 | NvOR263 | MMELELLRYKAYTQNVIWFLKLVGLWPESDPLPKKILSTITLSSILVVVVTVSNFSFHNLSNIVVFTSGMCMAASSTSAFSKIAMFLLHREDVVYLNKYLSGGFMRDMREPNNRPDLLNNVKTFDRLMVTHVICVAIALFTYSIRPLLVLRKHGKYIRSFPAIYPFAYEPGGLVHWILYAVEVSGTASLWTVTIGVDCIFGVYALQVCGELRVLSRKFRELRASDNYKEKLRDCIQRHHVLINAKNKLDNIYGLISILLITSATLVLCSLVFQVSELIKTNSYLRVAHLCVYLIPKFLQIFTYAWYGNLIAEESGACLDAMYGSHWTDACDKNFKNDILIVLAQEPFALVAMGCMVLQLDLFAKTVKTAVSYFFLLQTLNEKNE |
| 299522936 | NvOR264 | MKTKDESLQPNIFLQHYLNINSKMLRYMGLVVRTKGNKTDSKSKILERLPTYATNLISIIDAFFQMRWIMDLWQRDNDLVMQITTSGISNIVCICKGFRLAYCREDIQTLFEKLATIWDQTCVPEDIRDTIVKKAQSTLVFCRCYIVMMLGLGICFALPPMKNFLIQYFARKEMNHTYDYSERVFLVRYPFEINSSSIYFSVLFEEQWVLFCSALYWVCCDTLFAQLTTHTSLHFEILQYDIEAVVNRENDEDRLKQSMIDFVKRHRELLRICHMIEKLFSPVIFTTMLLTSINICVNVFELREMISEAKLGDALLHGFHLVNIFFQLLVYCIFAERLTQQAGTIANATYNCKWTEKNNKLRIYLQILIMKSQKPFHCTAYGFFPIDHKTITIIVNRALSFYMMLETTN |
| 283436195 | NvOR265 | MKTEDKSAPLTPDFEDYTKINSLFLRCMGMGIGTDGNKKDRRSQIIERVPTALINVLCLLDSVFQVQWVSELWKTDKKLVLQILTNALSNIVCLCKGFQLAYSREDLQTLFEDLAMIWRKRIPHHEIRDEILRGAQKTLVFCRCYISMILVLGLCFGLPPLKYFILQFTDRNANRTYDYTERIFLVRYPFDVNNLTAYNFIFMEELWVLYSAAIHWMCCDTLFVQLTSHTSLQLKLLHYDIEASGNTEDERQFKENVMDIIKRHQELLRICDLIEDVFSPVLFVIMLLTAMTMCVNLFELREMLLEAQYVGAILHSFHLINVIFQLLIYCVYAETLTEQAGSIAEAIYNCKWTENSHEVRTNLRMCIMKSQKPFYCTAYGFFPIDHRRITYIFKTAMSYYMMLHQTTS |
| 299522938 | NvOR266 | MKTEDSPSVTPAFEDYTKLNSLLFRCMGMGIGTDGNKRDKRSQIIERVPTVLINIICLLDFVYQMQWINDIWKTDKKFVLQILTNALSNFVCLCKGFRLVYNREDLQTLFEDMAVIWRRRMPRHEIRNEISREAQKTLVFCRFYVIMILFLSLSFCLPPLKYFILQFTDRNANRTYDYTERIFFVRYPFEVNNLKVYNFLFIQELWVLYAAALHWMCCDTLFVQLTSHTSLQFKILHYDTETSDNTKDERQSRKNIVDIIRRHQELLRICDAIEDVFSPIIFIIMLLSAITMCVNLFELQEMFLEAQYAGIALQSFHFMSVFFQLLVYCDYAETLTEQAGSIAEAVYNSKWTENGHVLRMNLQMCIMKSQKPFYCTAYGFFPIDHQRITTILKTAMSYYMMLYQTTS |
| 299522940 | NvOR267 | MSQPTEDDLEYYFAFNLKLLALVGFKCSMDKKEKGLGFVNKLPSYIMCIQGTILSLFEVYLLRDIYKDEDKTIVMQVLSQGVENTLNVCKGFFLAYSIERMENVLQEIKFLWNTYRPSPDNRKIILAEAQQTYSYCKIYFCVLASCCTSYFLCYLPALFKLAQQYRDREANNYTYDFSQRLLLLKYPFDIPSIPIYFLVELQEGFYLFYAAALFFVSGDTLFAQTVTHICLQFKILKFDIDAMFNPENTGEKDHLNLVTFIKRHRDLLRVCALIEEVFSPIILSMMLLSSIALCVDLVGIRGTMEKNNYEETAVVITLMMLTLLQILFYCTFAEKITEETRSLADTMYGCNWTMKNNKLGLYIHLMILRAQKPFQCTAYGFFPIGHSQLTTIINTAFSYYMMLQTTS |
| 299522942 | NvOR268 | MLPPTEDDFEYYFAFNLKLLSLVGFKCSLEKNEQSLSFINKLPSYMMCMHGIILSMCEVYFIRDIYSNENKTLVMQILSQGINNTLCISKGFFLAYSIERMQNVLQEIQFLWKTYRPSQDNRKIILADAHRTFLFCKIYFCVLASCCSSYFLCYLPALFNLAQQYRNRDANNHTYDFSQRLVLLKYPFEIPNIPTYFLIELEEGFYLFYSAALFFVSGDTLFAQTVTHICLQFKILKYDIDETFNSESTGERDHSILVNFVKRHRDLLRICALIEEAFSPIILSMMLLSSLSLCVGLVGVRGTMAKHSYEETAVVVTLMMLTFLQILFYCTFAEKITEETRSLADAMYSCDWTVKNYKLGLYIQLIILRAQKPFQCTACGFFPIGHSQLMTIINTAFSYYMMLQTTS |
| 299522944 | NvOR269 | MKTSVNQPTEDDLEYYIGFNLKLLSSIGLKCSLEKNLKSLGLINKLPTFIMCIHGLIFFIFENYFIRDIWSSDKTLAMQILSQEVSNIQCISKGFFLAFAIERMQNVFQEMQYLWKTYRPSQDNRKKILLGAHQTFSFCKIYFFVLLSCSISYFLCLIPSLFNLAQQYRNREANNYTYDFSQRLGLVKYPFEIPNIPTYLLIVFQEAFYLFYTAALFWVSGDTLFAQSVTHICLQFKILKYDIDATFNREDMRDHLSLVTIVKRHRDLLRICKLIEEVFSPIILSIMLLSSLNLCVNVVGIRGTIAKENYQETAINVTIFMLTFLQILFYCTFAEKISEETRSLADTIYNCDWTVKNYKLRFYIQLIIMRCQKPFYCTAYGFFPIGHLQLTTVLNTAFSYYMMLQTMN |
| 299522946 | NvOR271 | MKMTAINPEDYFGLNIKLMSLCGLRCSMTKTIGSFINKVPTFLANLVGIIYLVFQATFVMEAVRLRDVALTSQILSQLVSNIQCITKGFLFAVSIEKMQSILYEIRSLWERYQPDIEIQESILDDADRTLNFCKYYVIANFSCVLAYALPLVLNLFMQYQARESTNHTYDLSQMILLVKYPFEVTKVSRFIILVLLEEYLLVVSVIIWVSSDTLFAQTTTHICLQFKVLKQDIEKTFNYGGPNSKEILLKLVHRHRELLRMCMLLEDVFSPIIFFTVFLSSVNMCVNVIGTRETISDKTYLNTGIYATILTMTIFQILFFCIFAEKISEETTSLADMVYNLNWTAKDNQLGFYIYFIIVRAQRPFYCTAYRFFPIGHQRLTSIIRASFSYYMMLQTTDNK |
| 299522948 | NvOR272 | MKMTSINPEDYFGLNIKLMSLCGLRCSMTKTIGTFINKVPTFLANLVGIIYLVFEATFVIEAVRLRDVALISQILSQLVSNIQCITKGFLFAVSIEKMQSILHEIRFLWQRYQPDEEIQESILDNADRTLNFCKYYVTANFSCVLAYALPLVLNLFMQYQARESTNHTYDLSQMILLVKYPFEVTKVSRFIILVLLEEYLLVMNVIFWVSSDTLFAQTTTHICLQFKVLKQDIEKTFNYGGPNSKEILLKLVHRHRELLRICMLLEDVFSPIIFFTVFLSSVNMCVNVIGTRETISNQTYFNTGIYATILTMTIFQIFFFCIFAEKISEETTSLADMVYNLNWTAKDNQLGFYIYFIIVRAQRPFYCTAYGFFPIGHQRLTSIIRASFSYYMMLQTTDKK |
| 299522950 | NvOR273 | MQTNTEEKAITAVDAEYYFDLNIKLMSLIGLKCSMTETVTKFIYKIPTFLTNVLGIIYLIFQISYVREAVRSHDTSLAAQILSQTVCNIQCNSKGFLFVISIAKVQAILHEIRILWETYPPDDEIQKSILLVADKTVTFCKYYVTANLSCVLAYALQMGLNFFMQYQAREATNHTYDFSHIILLVKYPFVVTEIPTFITLFLSEEFLLIMGATLWAIIDTLFAQVTTHICLQFKILKRDIQEKFNTEGSNDKEILLKLLRRHRNLLRICMMIEDIFSPIIFFTVILSSVNMCVNVIGARETIASKAYFETCIYASIFLMTIFQIFFFCIFAEKLSDETTSIADTVYDLNWTTKDYKLRLYLRFIIVRAQKPFYCTAYGFFPIGHQRLTAIIRASYSYYMMLQTTDGKOR |
| 644998111 | NvOR275 | MQSKERDKPKVLDIEYYFDLNIRVMSLIGLRCDGPKITGFVHRIPTYTSNTIAILILIFEICLMSDPVCSSNMELTIQTASQTVSNIQCVSKGFLFVNAIEKLQVVYNELQVLSQKYPLEDEIQVLVFDIAEKTMNFCKYYAIAICSCILFYYTPIVVNVIVYILQDPSTNHTFDFTQTLFYLKYPFTIKTFPIYSTIVSIEAVNLIAQGIFWFLGDTLFAQVTTHICIQFKILKHDIQKTFNDEGSKSKEILIGLIKRHRQLISMCMLTEDIFSPVIFSVMILSSTNLCVNIIGASTAINDGDYMNAGVYATILLITVFQIFFYCIFAEKTTEETRSLADTVYHLNWAMKDHHIRLHILLIIMRAQKPFYCTAYGFFPIGHQKLTSILSTAYSYYMMLRTTANV |
| 644998125 | NvOR276 | MDYKMQTKEETIEVNAQYYFSLNLNLMSMIGLKCNMTENVGRFYHRIPTFITNVCALMYQSMTVYYLVEAISAKNTSLSIQIISQLVSNIQCFTKGFFLAFGINKIQFILQEKQILWKKYPPNNNNHHTILGIAQQTLTFCKFYVVAIFSCVMSYDVPLAINIFMQYLKRESTNYTYDLSRRVILVKYPFEVTEISTYVILCLQEALFVFIQCIFWVNSDTLFAQVTTHIGLQFKILKCDIEAAFNRDDAKNKEILIELVNRHRELLRICMLIEDVFSPIIFCTVFLSSINICVNVIGVRETISEKAYLDTGIYFTMLLITLFQIFFFCIFAEKLTEETRSLADAVYNLNWTIKDYKLRVYINLIIMRAQKPFYCTAYGFFPIGHQKLTGIISTSYSYYMMLQTTDK |
| 299522955 | NvOR277 | MKTLAKVQSTKDDIEYYFGFNLKLLSQIGLKFSMNEKTDKFTFLQKLPSYIFLVEGMILFILEVYLIRDTIQSDTLLSIQIMSQIISNLQSVSKGFLVLNKAKSIKNVLETLGIIWRKYPLNNSDRALLNAPSKIISLSKIYWGIAVALLVIYDLPPFVIFFMQYQNRDAMNHTYDLSQTILLLKYPFNITRKSTFFFLISQEAFVLYASGVYWIGSDALFAQFTTHICLQFKILKCNTKEVFNRGSKEAHSSLIDLIKRHRELLKICEMTEEIYSPIIFSTMLFSAINMCVNVVGVRETITRGFYQETGVYLFLFLVTFAQILLFCIFAERITEETKSLADLAYNLEWTKEDHKLRVYILFIILRAQKPFSCTAYGFFPIGHKKLSSIINASFSYYMMLQTMS |
| 299522957 | NvOR278 | MKSQENIFTKDDIEYYFDFIFKSLNTLDLKFSISKKTDEFKFRHKLPTIIGCLIGLIIFFLEIYFIRDALHNHTILPIQIFSQVISNFQSISKVILIVYKVNKIQQILEKIGVLWKTYTPDEGNRAVLYNTLQRTLSICKIYYAVLIATVLIYYVQPIVNFVGQYGARNSINHTYDYSQTLVIIKLPFKVTQKRYFFVISQEAYLLYMSGVYWGCSDTFFACFTTQICYHFKILKYHTKAFFDEKNNNSRLNLVTLIKRHQELLRLCVLIEDVFSPIIFSTILFSAMNLCVNVIGVQETILNGSYRQAGIYLFLFIITFSQILFYCAFAEATMEEAWSLADLAYNLEWTSKDYKLRYYIHVIILRAQKPFHFTAYGFFPIGIQKLTSIINASFSYYMMLQTVS |
| 299522959 | NvOR279 | MKSRENIFTKDDIEYYLNFILKSLRTVGLKLSLSKKIDEFKFRHKLPTIIGCSIGIIIFFLQIYFIGDALHNHTILPIQIISQVISNLQAVSKGFLVVYKINKIQRILEQIGVLWKMYTPDESNRATLYNILQRTRSICKTYYAVLIATVSIYYLQPIANFMGQYGARNGINHTYDYTKTLLIIKVPFQVTLKRYFFIISQEAVLLYMSALYWACSDAFFACFTTQICYHFKILKYHTKVCFDVKNENSRLNLVTLIKRHQKLLRLCELTEDVFSPIIFSTMLSSAMNLCVNVIGVKETISNGSYRQTGMHLFLFIITFSQILFYCAFAEAMTEEACTLADLAYNLEWTSKDYKLRYYIQVIILRAHKPIYCTAYGFFPIGIQKLTSIINASFSYYMMLKTVS |
| 283436107 | NvOR280 | MKTQENDLSIEDDIECNYSGYIFKAFHFMGLKLSLKKKTDGFKFVHKLPTTIGILQSIVVFFLQMNFIRDVVQCDSNPPIQIISQVISNIQAGLKQTLLVFKKIEDIQRMLETLGEFWKKYSPDKNYRVVLFRELGKTSSLCKYYFGTLVGIMIAYDVQPLVYFLTYYFEQNATNHTYDLSRRILLVKYPFEITRKSTYCFLLSQEAYLLYITAIYWANGDTLFAQFTTHICLQLKILKYETGKFFNQSNQEGRSDLLILIRRHQELLSMCDMIEDIFSPIIFSTMLLSAINMCVNVIGVTETIAAGSYEETGIYTFIFIATFLQIIFYCVFAETLTEETRSLSDFVYNLEWTSKDYRLRFLIQVIILRAQTPVYCTAYGFFPIGHQKLTSIINASFSYYMMLQTVK |
| 299522961 | NvOR281 | MKSQEDTKDDIEYYLGFILKSLHTAGLKLSISRKTDEFKFYHKLPTIIGCSIGIIIFFLQIYFIRDALHNHTILPIQIISQVITNLQSISKGFLVVLKINKIQRILEQIGVLWKSYTPDESNRATLYSILQRTLSICKTYCAVLFVTLLIYYLQPIANFLVQYRERNGLNHTYDYTKTLLIIKVPFQVTLKRYFFIISHEAVLLYTSALYWGVSDTFFACFTTQICYHFKILKYYTKVFFDVKNNNSRLNLVTLIKRHQDLLRLCELTEDVFSPIIFSTMLSSAMNLCVNVIGVRETISNGSYRQTGMHLFLFVITFIQILFYCTFAEAMTEEACTLADLAYNLEWTSKDYKLRCYIQVIILRAQKPIYCTAYGFFPIGIQKLTSVINASFSYYMMLQTVS |
| 289666787 | NvOR283 | MQIKVVENLTLTKHDIKYFFKENLKLLSKIGFKCSLTKKSEKFKFHHKIPTYIANFCGLIVFALQIYFVIDKIQTNTVLAMQSLSYAVINVQSILKGFMTANSIENIQQIFENLGIFWQKYMSRKPGRELILDRAYKTISLCKFFFVMAIVCYFLFVMQFLIKFSIQYLNREATNHTYDFSNTVDLIKYPFEIPNLPVYFLLISVEINYLFVCIVFWCNTDSLFVTLTSHVYVQFKALKLDTTLAFNNSMLKERSILIDMVNRHRELLRMCYLIEDTYSPIIFSTTLLSALNMCVTVYAVREYIDKGYYLEMGIPLFLFIGASLQILFYCIFAESLTDETRSVADSVYNLKWTTKDNKIKFYIQMIIMRCQKPFYCTAYGFFPIGHQQLTSIISAAFSYYMMLQTMSN |
| 299522963 | NvOR285 | MKIVGEKSSPNKDIEKYLGLNLKMLSCIGLDVSLENDDVIQERRILEKMPIFMTNGLGIFAAILQISLITDSMTHNRMFLATQVSSHLFSNMLCISKGYQLATAIAKLGEILREIALIWKQNPLNDEFHRNILSDAAKTLLFCKVFVVVTLCAVFGFGLPPLQNLFFQYLHARNSANHTYDYSQRVFIIEYPFPIQDVLTYSSVLLEEEYLLLASGLYWVCCDTLFAQLTTHISLQLEILQYDIETLINRESAEDRLNENFIIIVKRHRKLLSICELIESVFSPVILTTVVLSGMNICMNVFELSKTISEGNYAEAALHAFLFMNTFLQIVFYCTFAEKLTEQTSFVANSIYNCKWTEKNCKFRVYLQMLIIRSQNPFYFTAYGFFPIGHKRLTTVINTAFSYYMMLQTTS |
| 299522965 | NvOR286 | MTLLHETVNHPPQNNDIREYLGLNLKMLSFIGLEFNLNNDRPIKKSKFMQILPIFMTNVVSLTIAALEITFIAFVLRNHEEHLAVQICSELFSNILCIGKSLRMATAVASIQTALDEVSILWAKHRPNQHCKMEIMKKARNTLNFSRWYLGFIITGIAGFALPPIHNFVYHYFIRDANNYTLAFSKRIFLLRYPFEIKNVPLFFFVLTEEGYILLISAMHWVTCDTLFAQITTHTSIQLKILHYDIGALINHETVEHRLKAKILIIIRRHQCLLRVCRLIEDIFSPVILTTVLLSALNICVNIFETKAMNAEGNYARAALHANLVLVLFLQILFYCSFAETLTNQTSAIAESVYNCKWTEKNHKLGFYLRMIMMKSQSPFYCTAYGFFPIGHARMASIISTSFSYLMMLQSMS |
| 299528645 | NvOR288 | MKKTILQEYDKENQKAFDEAKTLITWNKYLMSALGLWPSHRYDFIFVSLFCYYIFHFLLDYAAFYFALRSFNLIKIIGATMENVTMAQIFLRLYTMRRYNRQYGEILEEFTRDFSVKNYKSEEERNTFLSYNSRSKFFIKIVVIFLGVTAILYFTKPLIRQLSLSKNVNTTKAFTYDLPYRIHLLYKITDIQTYIATYISRIPILYIIGFTQTAMDCLTLTVIAHLCGQLGVLSIRISNLDVVNKSNELNEIIQRHQKLIKIGLRLRRMYRLCLLGHFLGATIAICILVYQVLISIAAGQKTNLVTFFVFGFLNIFRLYTHCWVGEYLIHESINVSHAYYRCKWYKLPLKDQKSFIICIKRSQQPLSLMAGNFSHYSLVMFTNVMKSAMAYLSFLRNFI |
| 299782524 | NvOR289 | MMQNHQLQGQAELDDSSQVFRYNYILLTTLGLWPASLSDVRFFLNFGYFCYEMLLEYLDLFLFIDNFENVLMNLTENMAFSQIFIRMLMLRIYNSELGEIIGDAKKDFDAKNYTEEERKTFVAYHVKSRTFMKLLITNTALTASSYYVKPLLGQMGELMEYANSNGENSTFIFMLPYRFYTFYELNDAQTYFWTYGSQLPFVFISGFGQSAADCLMVTLVYHVSGQMAVLALRIASIDTHPSKCTQEVQKIVKAHIRLLRMGKVIQRTFSATLLGHLVGATSLVCILGYQILTSLANGERAILISFFAFIFLVLLVLYAHCTVGESLITESERVSQAYYDCEWYNMSKENARIIILCMARSQKPLQLTAGKFSMFCLQTLTDSIKASMGYLSVLRTVM |
| 299522967 | NvOR291 | MNISGSEQTILAKYKNDLQKASKILTWNRRLLSLLGLWPESPMDLLFCASAVYYIFYLGLIFVSFVLYLKKKILNVSIFIALLSYGHISARLLLLRRHNRTFGVLFAEMKQDYELRNYKSDQELRVFLKYNILAKSMIKFLLFCSTFFAIVFYVKPLLMTYNIHRAIRKSHRNATAPFVLAQNSFYQFYKITTVKKYAINYVSMLPFSVLTGFINCATDCLVLTIGCHLSGRLAALSHRIRNVEFCNGSQEFKAVIRLHQQVLRIGDMVENSLNTLMTCHILTAGVIMCFILYKTLIYLRPGKRIHLIHIVILLSLNIVRLYFHCCVGEFLMQESRVVHEAFFECKWYTMLLQDRKLIVLNLLRSQRPIRFAARGLGTFSIELFSEVLKSSLGYLSVLRNVI |
| 299522969 | NvOR292 | MNLYESDVNQKSLTKCKDDLKNASKILTWNKRLLLLLGLWPESPMDFLFCASAVYYIFYLGLDFVSFVLFLRKKILNVSIFIQLLAYGHISARLLLLRRHNKTFGILFTEIKQDYELRNFESDQELRMFLKYNRPAKTMIKLLFICSTFFGVVFYVRPFLTTYFVHRAIRKAHRNVTAPFFWNTYFYKFHKITTINVYAMHYVSEFPFSILTGIISCATDCLVLTLGCHLSGRLAALSHRIRNVNFRNGSQEFKAVIRLHQQVLRIAEMIEDSLSSLMLCHILVASILMCIVLYKTLICLRPGKRIHLINTVILLFLNIVRLYSHCCVGEFLIQESRAVQAAFYECKWYTMPLQDRKLIILNLLRSQRPIRFTAGSLGTFSIQLFSEVLKSSLGYLSVLRNIV |
| 283436207 | NvOR293 | MEFVRKAYGDKQRKRQASSKCISRADRVFKRCVFFHKFVGIWLEKDRSQRLLDRLKGYVSAAFTLGICIFQIVMLSVESSSVVTVLQNNLLILIRKTKEIAAPLHASKIKERKIVDRWLNNQDKILKILLTSYTFTFSSYSLFPLLKENGLPFTGRLPAICYVNPWYPTIFAAQLVFIIFRFFCVLSNDILCITFLCQLCSELELVKHLIVELGNGKDRNVKQIIIRHAMVLDYGEIICETYSATLIMQHLNCSIFLCLSGLVTMKTSDMFALLKIGSLSLIGIITMLIICFVGEMVMSSSLEIASTIESSVYKDYRNDVANLKLLNFMLMRAQKPLCMMVCTQGKLSLRFFSENINKVASFFIYLKTLVE |
| 299522971 | NvOR294 | MNIKSAESSLQSTTFEYEAIFHKVVGVWPGDDYFLARYSRIRGYVLAAFAVVVCVFQFTALLEANSDDVPENDFINLMRMTKEINEFSTLTDEEETIHIDWQSVQDKLMKIISRYYFLTVIGLYFVAPMFRNALPLRGIVPEVLRVTPWFQMIYVLQCLLLLSNVITSISSDAFSVTFMCQLCKQLELVQCSIKHLGSHTKVNLAETINRHAVALDYGQRVCNTLKTMFLLQHIFISMFLCFAGVIVLNTQNSLILMKMIVISVIFVSTLLIICFVGETITSSSLKIASATESSNYEIFLGDVSTLRTVSFILCRAQKPLRMAVSLSGSMNLSFFTETMNKLVSAFMILRTMME |
| 299522975 | NvOR295 | MGLIEVLESRKIFLWICGLWPKEYQHKPKLSQMKLYFIWFNMLMMCLLVFAGLVAIATPDNIPQASIRLPRKRMMKVIMETLKLEETSKFENDNDLEIIRSWRRTDGVLKYHMRIYGFISVAYCFLPIISQVNTYPAQTIIQASLFVSPWYEFFYGFHCAQLFLYLFIIIATDGLSMILIFKLCEELQRFECLLFERHRADDATLSEKYKSRGEFLRCIIRKHCTILDYGESICNLLTGALFTQYFLLSGTLCFSVFTILSSNSSAMANQMSIMAGTCIVQLFMISLAGELVSTRSLALADALLQSDFCCSIFGELKSSELRQTVLMQLRMQKPLKLSIGTLGVINIEFFSRIMKGVYSFTMLLRTSYV |
| 644995274 | NvOR296 | MYVKLLSTREPHGGDASDGQLINTELRPKHTNLLYRTQAKIKMNVGEEYDKLALPMTLSSRVVGSWPSRAELEGQGGRSVLVHRLHRYLAIVSIYLMSMGVAAEVIVFFGEDMNETIECALISSAFFMALTRIITFASHQPEMLYVVETMREDWIRSTDEERAILRDKCLFAFKLAKFFAISVTITCSAFILMPMLELKFVENAKRMLPYRGYFFFNHTVPGVYEYVYLVNSMLGVLGCSTIACATSFSLITSIHGAAKFAIVQKDFERIDQVTWNNSEIVGRCVRRHQECIRFAETVENIINVLALAQFVISTGLICFAGFQMTTMLTDRARFTKYASFLNAAVTELFIFSYGGQSLKSESEEVAEGVYSSNWIGSALSSNLRLIVLRSRKPCTITAGKFYDMSFESFLKVLSSSFSYFTVLLAMEEE |
| 645014749 | NvOR298 | MLGKSSLNSKIPIRERDFNYSMKLSRITLSIIGLWPFRENIRCSNFKFVVILVSILMTLLSSLTFVYQTDDDDKMFHSLINSLYMLMTLVKLLMMRCKNDKLEVILSEMRIDWRKYERFSDGNKRLVDLYTGKARTSSFVCIFFMEFSITTYFISRVAYALQQPAKIREWDLPYTAVYPFEVTSSLFVPMYLWQVFSAMCLGSVTISIDCLLVTTACHATGQLAALCENIKSYGHEQRHRDETLSSEIECSCIRCIIERHVDIVRYCRLVEDAYNLILLTEFIGTTFQFCLQMYIIVEHSHDKNIVGLLSFCIYLLVFNFRLFMYCNVFDAMVEMGEKVGASAYDISWYDFHPEAVRQLMFCILRANKPLNVTAGKFFSLNRNSYKNVIMTSSSYASVLLSIK |
| 299782528 | NvOR299 | MKTESSNARLKGKNVTFCTEVFECDEILQLDNELGLIQWSLKLMGIWPFWTRFSNVKFFLCGAILAFNVVGCFSGILNVNSDIEQFIECLLYFNVNLATLLKFLIVKYKRRSIEFILRCILDDCSRYSHLSVSCRSRVAGNIKKRKLMTLTLALFILAPVAGKRSFLSMLWSTAYIFSAFAITKYIEYRDDLAMIRELPIFSALPTFVRHSQIFYLALLSGLFGILMSTLVIVTIDSVFAILMIHATNQFIVLSEELKAYREDHLDACYKISKNMRNCKCMRCIIDGHVNILRYTLNFYWYSKFILYKIDSVFSIVHIAVILLSMNLHLFMYCVTSKSMTDASEQIGIRAFKMKWYSFQKTTVRSIVLMTLRSQIPCYVTVAKFINLSLETYTSVLKTSISYASVIIIAREHLVNER |
| 299782530 | NvOR300 | MGKGKVRSFSDYFWLSQGMLKFCGVLPMPERGLFVNYFLIMLSISSLVFLFFPGFYIIAFHGSEINAAAKVDIIAGEALEIWVTTIKALVLLPCRQTMLSVSRRATRLLVDIEDEKEQQLAEPYARRGYYLLYGFGGTVFFALLSIVIKPFGQQVQYGANGTILASKDLPYSIGIVHENQQLFNAWWIGQCFAGIIAIIAIIGIDTTLAIFVLHACGHFRILRSRFQAVAENSSSRRMSVSGRDDRRRLIDLIDKHQEIIQFVSTIIIIRYVITLRKCFLFSIKLLRRNRIRLQSGGTDSNDTQHLSDLRIRLQFAGGKSLYIYIYTSSQVGFEAYNLRWYDWIEDDKSLVTFLITRSQKPMLITAGRFTSISLETFSAVLSSAFSFFSILRKTL |
| 283945514 | NvOR301 | MFVRIIILNEINFLGNMDSEIYDSEYYHEVKLLLTYFGLWPNLSRFRKVVSFIAMVAMPISLVIPMSFGLKRAIRLKEPIQIIEDTIGILYFLAITTKYICTFIFEGRMIVVYEQIASDWKKIKDKNELEYLHGRAKEGKIITILYLGYGAVGCTIFASTPYLPLFLDLVIPLNVSRDKIYPYYADYDIVDSEKYFYTLYTLHGIFIIILVTMSAISIDCLFIMMVKHSVGLFQIVCYRLKKIGEEHNEKPHECKRLMDDKIIHTRMKEIFDSHKSSIECVDAIQASFDVSFLFIMTMSGVGVSLILFDLLLNLDDLTQILRINSMMFGVYIAVFVICYAAQMTLNSSEIVFNDTYCGYWYNISPNARKYTQMVMVRSMKPCIITAGGLINMNLQSFFAILKTSVSYATVMLSMQEESNMQN |
| 383865819 | MrOR9a | MVTSLVQEMVTPGSSRDQEVLELYVAIQAKDIDEVIDILPHIFVVIASLIKFGNIYLKKDRFKILLDLIVKDWETLTNELHVLDKVTATGDKLAYLYRMTLLSFLVAFNYIPLIPPTLDIILPLNETRPRQQLFQVNYIFFDVDDHFFAIYLHMSWAGSLTVFVIVTIDSLYMLIIHHASGLFDVCGYQVQMACKQKKMEDEMFKQCVITHHKALELVFDFLEECSQIMNLMLVGMNMIAISLTAVQIILYMDQPMDAMRFVLFLIAEKFHLFILSLNGQILLNHAVALTDKIFSSNWYDIPVKYQKSLYMMTIRCSKACMLSAGGLYDMNIENFGKTVKACISYFTMFLSVRG |
| 383865817 | MrOR 13a | WLRCVTLSFTMWSLLFGIYLQTTIMYHSLDDVENLIFGLLNLLSVLVPLIKMLALLPRRKKLFGLIAYMVRNFLKADYDDFETSILTTCKRKCTFFVCGSVCFTELTIVSYVCAPLLVNLFMNESERVLPFKMYVNLPIQATPYFEIAYITQVLALCPVGFSYFSLDNVLCIINLHIAGQFRILQYRLSDKYSGQVQNGLDQKSDLYLKNNASDVFKSCIRQHQTLITYCKQLQEVFGLIVLVQVMTFSMLICLDGFQVLLVDLVQRKVIFFFHLLTTVCQLIMFSYSCDCIIRESVNLATAAFSGPWLQLPASKRIENLKKDFIMLIMRSNKPCCISGSGFFIVSLETCTRVITTAGSYFTLLQQAQNNISS |
| 383865811 | MrOR2 | MGMEYTSDIAVRLTIFYLKVVGFWFAANRLQQLFRCLTVIYTIVMSVFALWIQSMGIYYCWGDYTMCTYIFVNILGIAISLLKLCLLLVQKEKFLRLIEFMQRNFWHSNYSQKEIKIFAGTKRICIYFVCSFSFISQLTVLLYSIRPVLLNIGKNESERVLLFHMWLDLPLSTTPYFEIMYVLQVLSLYQCDVGYICFDNIFCVMCLHSAGQFRILQYRLKNVHQLASKHEDNSNNSASYFSYKCFVVFKDCVRQHQIIIAFCKLFEEVFKVIVLCQVIMFSMLVCLVGYQIFLVNLNLPMRVSLTSFIISNLCQLWVFTYSCDTMTRESLSVGTAMYEAPWPQLPTDKFGKMIRKDMQIVIMRSKRGCHITACGFFPISLETYTKIMSTAMSYFTLLKGSTIDVDT |
| 383863174 | MrOR1 | MENRTKLKKIKPNKHLENSLSIIYYMGMWPSESKYKRLYMLYTVFSFMFLLGIFLASQIAYIIVNRKSVDKIIAGATLLMTNATHAYKAILIICHHKRIKDLTDITRSETFIQDNGKYEKIVRHYTWQGVFHHIAYQSFGLMAVISWGVTPVLNLLTQRSKELSMEGWYPYNTSSTPAFEITSSYQAVAIFLCCINNVAIDTLITGLITIACCQLAILSSNIASLNCAENTEPIGINNNTDLEISTSKNYNKLYEDLKSCVEHSNMIFDFSKQIQDTFGTVIFLQFLVNCIIICLIAFNIAQMKDYIPYVLCGMLMYMCCMTYQIFIYCWHGNELYLHSMNVTFSAYANNWWYNSKDFKQAICIIMVRVQQPLILTAGNVMQLSLQTFVRILRMSYSIFTVLQSSANS |
| 383862405 | MrOR 82a | MKKPISLTIESFFDENVLSWSKRLLSLSGLWPENRNDVQFFFYITYVVIFTWLEIVTLLQNIHDLERSLKNITLSFPTILIVLKAVMFRLNMHLVLPLLALVKRDVEQGLYQTQEERQTAVWYNVAATLFSTSSALSLFFVPTLFYTKPVVGCFLSQFVNCTLPYELPMKVNHIYEVTEMRTYALFCVYLIPVSMMLTIGATGADSLLVTLTFHICSQLSILSQRVRSIDLEPQIYFPKMKILVERHTELLRLANVLSETFSSLMFVQTLGLIFSLCIVVYQLLMTSESGEDMNTVHFIIYSCAVVLLAFCYCFLGECLINESSEIQAACYFTNWYNLPDKYVRSLMFCIARSQKPLYLTAGKFYVFSLETFGVIIKASMAYLSVMKSMV |
| 383861646 | MrOR 63a | MKKQSNNSIDYYILPNKILCTTLGISLSDKKRSLCGQIFAYLRLFVAVASISSFVVPQAMLLFIKWNDLKILSEVGGILTTLAQFEFKLIYLAIRREKTYKLYKEVRSLWNSTDDPEEKRSYEEFAYWARRFTIIFYSFGTWTTVIYTASAAVDCIIIQYSANNDTITRSLPFDVWYGTNVSESPSFEIMFTVQTVSAIYNSAAVWGIETSCMTVILHVSGQFKLIKTWINNIGVKIKNEPKDHYYKCPPDIEDGLVRCIRHHQRLVNVVNELNDLLIPIIFIQLLTSGIKICLSGFAVMNNNTNAELIKAVLYLFGMTTQLLLYCYPGEILIRESEEVGDAAYLNVCWYKLPPSNRRQLLLTILRAQKCCSITAVTFQRLSFRTLTGVFNTAASYFTLLRQMQETSM |
| 383861638 | MrOR 92a | MEKVSKDTIDYYILPNKILCSMYGIWPSTEERSTIKKVFSVLHLVLSVMIACSVLVPEIMLISSNWRDLSVVAGAGSLVVTVGQFLFKTAYLVAKKEKACRLLDELRSLWNSTDDPVEKKSYEVFAYWGRTCTIAFFVSGMSTTSMFMISGVLDSLNQQDLDNSSNRYLPYDVWHEMEYSKSPEFELLYAGQIMSSFISCFGLCGLDGMCLTTILHVSGQFRLITTWLNNIGIEMKCKPIDLHNCPVKLTADLVRCIRHHQRLINVVEDVNTLLAPIIFVQLLTGGIQICLSGFAVLSNNVGDDLVIFIAYLASVTIQIVMYCWPGEILIQESQKVGHAAYLNVPWYQLPLFHRRQLLLIIIKSQKYCCISALTFKSLSSHTLTNVFNTGSSYFALLRKVQERSM |
| 383861632 | MrOR 22c | MGKVAKDSIDYYVLPNKIMCSAIGIWPPDEEQSFGGRLFVGFRVVFSIAAVCTIFVPEIMMIAVNWGDLRILTGVGCVLTTVAQLIFKMIYLIARKERSYKLYKELRSLWDSSHDSKERQCYQGLAYIARNCTIIYHTSGLLTVAVFTVSAVFDYVKFGQDNNAANRHLPYDVWYGTDVTDSPGFEIAFACQVLAASICTIGVTGLDTTCATSILHICGQFRLMCMWISNIGIKINCDSPRTVTTDLIRCIRHQQRLISAVKDVNNLLTPIIFVQVLTSGIVICLCGFAVLRGTGDDLFKFIVYLTAVMIQLMFWCWPGEILIQESLEVGYAVYLNIPWYNMEPACRRQLLLVILRSQNVCSISALTFRTVCIHSLTTVFNAAASYFTLLRQMEEKAMSK |
| 383860612 | MrOR 7 | MMKFKQQGLVADLMPNIKLMKASGHFLFNYADGSGKSMQKIYSSVHLVLILMQFAFCGINLVQEREDVDDLTANTITMLHFTHTIVKIIYFAVRSKLFYRTLGIWNNPNSHPLFAESNARYHQIAIKKMRILLLAVMGSTVLSTLSWTILTFIEDPVKKVTDPVTNETMFVEIPRLMVRSWYPFDASHGMAHVMVLIYQFYWLLFSMASANLLDVLFCSWLLFACEQIQHLKNIMKPLMEFSATLDTVVPNSGDLFKAGSATQNQVPDQEPPPPLTPPAGDNMLDMDLRGIYNNRQDFTATFRPTAGMTFNGGVGPNGLTKKQEMLVRSAIKYWVERHKHIVRLVTAIGDAYGIALLLHMLTTTITLTLLAYQATKIHGVDTYSASVIGYLLYSLGQVFMLCIFGNRLIEESSSVMEAAYSCHWYDGSEEAKTFVQIVCQQCQKAMSISGAKFFTVSLDLFASVLGAMVTYFMVLVQLK |
| 383857821 | MrOR 43a | MENFGSILQFVSASREQDIDTMMECVPVSATLLGTMIKMINHNNNRKRFENIFNLMAELWETAETNGEVHVLNEITEQGSKMGSLYRKSIWGFVMLFLCVPLFYPLLDYVVPLNETRPKAPLFKLNYLVNTDDYFYTVYFHIALCCLITVLIVTTIDSLYIVIIHYTCGLFAVCGHQVKKAAEIEGRNSHSRGRNQDLLKRCVITHYKAIRNHLICYTLRFHEYMEESTRMSYLFEVCLNMIGMTVTAVQTVMYFDKPEEAFRIVMFLLGQQFHLYIISLPGQMLIDQSLQLVNDIYFSKWYQMPVQFERTLHIMLIRCSRPCKLTAGGLYEMNIENFGSIVKTSMSYFTVLLSFRE |
| 383857819 | MrOR 49a | MLNLRSNNGEHPLKTCIAKYHRNERQTYDVSRIPCLRVFKKLLMYMGQYPCQTKINSQIRVTLLIVTLISLLLPGVSINNQEVLQFLSAMQEQDIDTMMECVPISATVLGTMIKMISHNSNKKKFENLFNLMAEVWETAENNGEVHVLNEITEQGSKMGSLYRKSIWGFVLVYLGVPLFYPIMDFVAPLNETRQKPLLFKLNYMVNTDDYFYTIYVHIVWCSIIIVLIVTTIDSLYIVIIHYTCGLFAICGHQVKKAVEIEGRNMHSSDRNQDLLKSCVIMHYKAIRFHEYMEESTRMSYLFEICLNMIGITVTAVQTVMYFDKPEDAFRTVLFLLGQQFHIYIISVPGQMLIDKSLQLVNDIYFSSWYHIPVQLEKTLHIMQIRSCRPCKLTAGGLYEMNMENFGSV |
| 383857769 | MrOR 94b | MQTLKWSYKILDILGFSLTSHWTSLWRRALYNSYGMILVISLHFMSATQMLDLFNVTNQEDFVDNLYVTLVFLCDCCKTIMLLRRRGNIAKLIDELKEEPFATLNAEETEIQRKFMQQIERNTITYALIIDVYVVATIIISFFTDYRHGGLKFRAWLPYDYSSPLLFTVTYVHQMVVMVFATNFIVACDSLFSGLLVNIYCQFELLEYRLKNVEKYSYYSLKLCARHHRRIYEFATAVNEVFTAIISIQFIVNTFALCFNHYRLSQLEFGAKFGEAAAFMFCVLAQIFYYCWYGNEVKLKSLTIVDVALDSTLMSLDNSTKKMFLTITMRAMEPIQFTSIHIVSMNLESFITLVKTSYSAYTMLQQMH |
| 383857763 | MrOR 71a | MGKIIIYSFFILQVLDIFFNVQNQDEFTENFTLTSMALNVLLKRHMLSTRRTNILSLIKRLDKSHFLPVTKEEMKIRSKFENIIECATKMYATGLAFFAFSVPFISIVIDFKSRKLYARMWVPYNYSSASLYLLTSSYEVVASIYGVSISIACECLYTGLILHVCCQFEILEHRFKTLNGKDTRVVNQCASHHNLIYKFADVINNEFKTVMSFQFFNSTAMICLSIYQLTYAKNSTAFMEIMMYLVCVFLQILFYCLCGNMVKMKSIEFSDNVYSSDWPSWNNSSKMVLLMVIRRSRTPIEFTSMHVVSLSLESFMSLLKTSYSAYNLMKTTR |
| 383863503 | MrOR 67c | MSTILIFQYWYCINHIKTDSLTDLLDGLCITCSNTLLLVKFLIIWSRRRVVSEILAIMAEDWTNCKSEWNLEAMIDKATLSYRITRIMLVSFLSSSILYTLGVFFGSDNDDGTSNPNERKFVLRMEFPFEATISPIYEVIVIVQIIAQATFAVMAGMLMTLNATFVLHLVSQVDIICERLTQILNDNNEEKSRVGIIKKIILKHQRILDLCNNVDYVLTFISLIQFFLNTVVICFLSFILVTSLNTDEAAIIISKCFPYFVVIHLEALILCYTGEYLTTKSKSISWAAYNSNWYQLSIRECRALLLLILRSQRPMTLTIGKYINLSLETFANITQAIQCDKCCIYIVLLMLKSENSTVNA |
| 383857343 | MrOR 30a | MNQGIYSDLSIVTAKYFLKLAGVWFTMDDAEERQRLVALLWVFYTGLYCVVVNVKNISHYWGHDMSCCVFGMSNLLIITMAISKVIVLRIRRLELADVVIYAERHFWHYNYDSEEQLIFAKCRKFCKIWIMFLFCILPASMSGYMTTPIVNNIGRNKSDREFPLEIRSDLPITETPYFELIFTFQTICVLTLGVAYICPDAILCILNMHVVSQFRMLQYRIMNCWVCENKQKDTLEYVNHCSAAMKECVRQHQSLILFCEKLENVFTFTIFWHMVIFSLLVGLNCYIILLADTPFARKSIFMFHVIGSFVHLIIFAYCCNGLMEESLNVCSTIYFGSWNTLPMNRIGRMLRLNVRMIMLRSMKPCYLTAGGFFPVSLETATSLLSSTFSYFTLMRERFLRNDNQ |
| 383850740 | MrOR 67a | MHARVSNGVSPNKYYDTDIKYTFELCQWILKPLGIYPFVYSNVSKLERGVSVLLLITCCSIIQFVIVPFGHYVLFYEKDINARIKFLGPLTFCSTALVKYVYLCLKAPAFKRCIEHVERDWKKLQDQVYRDVMINYISMGRNLITVCAMFFYTGGLSYHTVMPLLSKVKTENVTIRPLTYPGYEAFFDVQRSPTYEIVYFMHCIYVMVAGNITMVAYSLATIFTSHACGQMKIQILRLENLTDERVTEKRGEDRLTIIVKDHVEILRFTKLVESAFREICLIEVIVSTLLICLVEYYCLMEWETSDSVAILTYAMLLISFIFNILMFCYLGELLLEQGNRVATASYETLWYNLPAKKARDMVLLLAISKTPPKLTAGKIFDLSLYTFGVVLKSSMVYLNVLQTMIEL |
| 383850676 | MrOR 83a | MQKTSVYKKTIDDYEKNVNLSIQWCRWILKPIGVWPTSDVTGTQKCMYRLINVMCYAFLSFLCAPCSLYVILEVEDVYNQIKFFGPSSFCVMALMKYYLLILHENDIRDCIKRIELDWKDISHHDDKKIMMENAKFGRQLIALCTFFMYSGFAFYYIALPISVGKIQAKDENLTFIPLVFPFSSLIIDTRYSPTNEIVFFLQLIAGAVMHGITSAACGLAAMLAVHACGQMEVLMNWLKHLIDGRSDIGDTVDARIASVVNQHVRILKYLTRTQSTLQLISFVEFLGCTLDICLLGYYVIMESKSNDITSTVTYIILLTSLTFNIFIFCYIGELVAEQCRKIGEMTYMIEWYRLPGNKKLCCVMIIAMSNSSIKLTAGNMVELSIETFTNVVKTAFAFLNVLRTMT |
| 383850661 | MrOR 49b | MVYRNASYKLDTEYTIRFPKALLTPIGIWPLYQDDTALRKTRRQVQIALIFCSMCFLLIPHAIYTYHDCEDLKRYMKVIAAQVFSLLGIVKFWTIIINKNEISFCLTELELQYRDVECEEDRKLIRESAKIGRFFAILYLGLSYGGALPYHLILPLLSEKVVKSDNTTQIPLPYLSNYVFFVIEDSPFYEMTFAFQMFISIIILSTNCGIYILIAGITMHCSGLFEVINRKIDLFMKETNGKLRDRLRFIIQRHVQATEYAAMIEKTFNVVFLSEMLGNTVIICFLEYGVLVEWEDHKTLSTMTYFILMTSILSNVFIISFIGDRLKQVSTRVGRTAYFLPWFELPMDVVKDVSMVILRTSRPSSLSAGKLFDLSLQGFCDVFKTSAAYLNFLRTMTA |
| 383848904 | MrOR 46a | MIANLNQVLCVLELAGTFTCTWPASNSSKFRTVLRNVRWTFVMINVILLTVSLVFGIYYYRSDIVILTKSISELTALLEVILDLLFCKMNHRRLQGLIGRIRMYLQVADEQENKIIQSYVDRYKKLFSVIAIAYISTGISFSLAPLFSGQKLPADGWIPFSVEFVGIYWVVYLVQVYCILQTALCIGVDFMITTLFCFTAARLDILGSKMKRVNRYDLLVSCVKEHQEILGFVDDTKAAVQALLFKTNITMGSALICGAFPLIYNQSLAVTSQFLCMVVSGCGHLYVISWPADDLKESSLRFATSVNDIQWIGQPRKMTNVVLIMMQRSRKPCLITMGGLLPPLSLEYYAHFLTSISSYFMAMRTMIES |
|  | CcOrc0 | MMKMKQVGLVADLMPNIRITQAVGHWLFNYYSEGMRFPHKIYCMVTLFLMLFQFGTMALNLVKESDDVDQLTANTITVLFFMHPIVKVVYLAARAKIFYKCLGVWNNPNSHPLFAESNQRYHALALSKMRKLLFCVCGAVTFSVICWTGITFFDDAVRKIHDKETNETTIIPLPRLMIRSAYPWNAMSGAAHIFSMIYQFYYLVITMGICNMFDVLFCSFLLFACEQLQHLKAIMKPLMELSATLDTVVPNSGELFKAGSADHLRESSGIQPSSNGENVLDVDVRGIYSNRQDFTATFRPTAGTTFNGGVGPNGLTKKQEMLVRSAIKYWVERHKHVVRLVTVVGDAYGVALLLHMLTTTITLTLLAYQATKVNGVNVYAATTIGYLLYTLGQVFLFCVFGNRLIEESSSVMEAAYSCHWYDGSEEAKTFVQIVCQQCQKAMSISGAKFFTVSLDLFASVLGAVVTYFMVLVQLK |
|  | CcOR30 | DDMNETIECSLVASAFHMAMLRFILFSLRRKDMLYVLNVMRDDWTVNYRDPDDARLLADKTLAAFKLSKLFTLNVIFAGSTFAVMPALEQALSSGADHKTGVLPFGGYYYFNHTSSNLVYAISYTFNSMLGIIGCSTIAGATSFSLISTMHAAAKFALVRRHFQSITHREWMQQPSFKFNLCVREHQQSIKFAETVEKIINLLALAQFVVSTGLVCFAGFQLTTMLEDRARLTKYSSFLNAAILELFVFSYSGQRLKTESEAVADFAYSSDWLGAYRKSIDIRMVIIRASKACTITAAKFYDMSLESFLKVLSSSFSYFSVLIASQDK |
|  | CcOR77 | SDQSGFDVAIGPCRAVLNFLGAWVDPVEPQTTFALFRFFLSTSTMIIFSCLVQTIEVFRGWGDLNYVIEILIIDDIPICVAVMKFIVSFYNKDILKRLVVLMAKDWQSKYADEDWKIMWETAQFSRKLSLVCILLAEGTITAQCMVVLMFDLYSKDKKERPLYMHSYFPFNTQTSPNYEFTWFGQFMCTVYAASAFSSVDAFFGVLVLHLCGQLSVLKRNLKVLTHNDKENLDSDEFTRRIAALVDRHDHLNMFAKTLEDSFNLMFLPQMIGTSLAMCLQGYQLVVISTGSEEGLPLLQIFHMVYFTTCFSFSLFVYCFVAERLQFESTEIDYAAYQSEWYNWAPRDTRLLLLLMGRARKPLEITAGKFCVFSLGLYCSILKTCGGYVSMLLAVRRRLV |
|  | CcOR21 | MEAEVESYYREYKDGIVSLLTLSGLWPEDSRQIKYVRVALFSVTLIFELVFAGAVCNFAMVNSHKMSAVVASVAVLISFIFVIAKLLVMLIYKEDVLYLNRNLASYFESDLEITEYRPYLLAYYRVFYRFFKAHYYYFNAILLFGMTAPIVAAFYGKSARVYPIHPPFHYEPGGTRHWMIFVFEVISGAYSVTTTVSYDSLFGLYAFNLVGEIRLLTHRFRNLTPGKSYRNQLKECVDKHVILMNAKNVMQRLFGAMSVMLAITCAVIICAQIFTLTTNKQLPVFKVAFLIDYIIAKLLQAYMYAWFGNCITVESEICLDAIYNAEWAGSGELRLMKDILIVQSQNPMQFETMGIMKVRLDMFLKIVNTSISYFFLLKTVE |
|  | CcOR31 | RQQATFDDYTFLNRWGLTFLGIWRNESKADRSFRKVLHNLHVLVLFGLMTLLLIPQWLDLYVFWGNIDANAETFVLNVFTICAMNKLYYFHSARRVFKDLVSTMADNWEETMSETGPDGVKHRDILLDMASKARVYTMRYGFLMYSTASMYFVSPFMGMQPDDLRVRKYPFFGWYYFDRFSDVYYGLCYLSQLMAGVSVGTSNYSTDSIFLVAIYHSCAQLRIIQHDLQKLGEESNVSQAKIIRLVDKHQRAIRTARKLEAMFSGSSMQQLLVSCIIICIIGLKIIVALDQGGFQVLVYVAFMFVALLQIFLYCQPGDELVSQSEAVGYAVYQSMWYALDVPAKKKIQTIILRSQRPLKMTAGSFYVLSLPNFTKILKTSMSFLSLLRAMY |
|  | CcOR15 | MKTEFKKYETYVGNVETMLRYCGLWPVVVNRTISRSLSFLAFITTFSTMLSVLNFCYHHSNNIIVLTKGAGLAISLCTACLKVCIFVYHQQDLLYLHENLTTRYLMDMKDVNNRARLLNRVSLYSKFFWIGTVAAFATIALYASISFIAWAKYGKYVRVFPAIYPLVGKPTGLVHWAFYVYEMTTGLYLSFVTVAVDCCFGMYSMQMCGLFRVLSDRFRNLKSDRDYKMNIKDCIQRHHTLYTSKRKLENLFGILAIWFAVTAAVVLCTLIFQFTQTIKMRTTWLQLGLLTLYFLLKCLQAFSFSVYGNAITVESTLCLDAAYNAHWPDLYNVSLKNDILIILAQKPITLVAKGCMLIQLEMFAKIINTSVSYFFLLQTLEE |
|  | CcOR14 | PELWALTMVFDQFQLVIDNLLTSSPTFTSCFKLLFMWREAHVLEPVVESSARDWIVDPKKRNYRELEIMRRHAYRARIVTIVDYFIIMCCFAFFVYAPLFGLNIRIINNITDYAERRHLMVQTYYPYDYSTSPLFEMTVFVQQLSCLFIAMAVSIPDNYFVALVLHTSAQYEILGADIENFFPHSSSSPSVGASFFIDEQLNDRILASFVDRHVHLNRMVSLIEQSFSFVTAVQVFCMISMVCCLGFETLGMLDPNMTSSKPTPLQILTILAMLFYIMLHTLVNCLASETLALRSEAVHSNIYNSQWYTMPCKQMRYFIPMMIVSKNPQQIKAGRILPMSMETYCSIVKTTAGYLSVLIAVS |
|  | CcOR19 | IELYHKTLEINTYLKETSAEKQITYRWLSLQNKLISNVIISYLFIVGIYFLYPLYRGDAFPITPGLVPKVFNKIPWNILVFVYEFAICTLRLIVIMSCDIFLVIFMCQLCSELQIVGNAMIEIDVHDKRTIFRAIKKHKRVLDYGRVICDEFSPAIVMQHLGMSFAACFSAISVLYTKEQMLLLKFISIGLIIIAEILLLCFVGETITGESLKIADYLENKFEVFLDDATSLKTICFMLFRAQRPLSLRVGGNTIMNLNLFRLTMNKIFSCFVILKRAM |
|  | CcOR73 | RVNPKPRGQREVYVNAHYVQDTEYVVRVAKTLLSPVGIWPRTGDNDPTSNLIFTVRVILIFCLMLFLMTPHLIWTWFVADNLRKLMKIIAAQVFSSLAVLKFWTLIINKRDIRHCLEVMENDYKTVESEDDRQIMIKNAKIGRFFTVAYLGLSYGGALPYHIIMPLLEPRIIRSDNTTMIPLPYPSEYVFFVVEFSPLYEIVFVTQIFISALILSINTGVYSLIACVVMHSCCLFEVTGNKIDKWLANWTSDQPLSNALTRKLSKIINFHVQAILYAETMENALTIVMLAEMGGCTLIICLLEYGILLDMEDGDYLGCATYAMLMTSIFVNVFILSFVGDKIKEQSELIGFSAYSIDWLELPKEVILKDLKFIMARANQPTRLTAGKLFDLSLQGFCDVAKTSMAYLNFLRTLEIT |
|  | CcOR67 | SISEDWKTTKKKSELVVMWQKAKTSRRLSIVSVILGEGTTLAYTVRMFYVLFYNEEQVKPLYMHGSFPYDTQKNPNFQITWVLQIIATLMSSGIFSAVDALFISFVLHLCGQLTNLQVAFSEMGTKGPIAGPAFFKSMEILVRKHQRINEFADIIEYSFNIMFLCQVFLSTLLLCLQGYLFVITITSGEIVIVEMIFMLYFTICFTFSIFVYCYVAELLREEVAKLGTSIFYCKWYNLPAKEARLLILSLLRVKKPLEITAGKLCVFSLNLFCVILKTAAGYMSMLLAVKDKLT |
|  | CcOR51 | SQEFPTDAVYPFAVNNIYIKILIYCHQSIVGLQTSAAVLLDCLVAVLLWFVCARFEALAESIGTYSRFDGIKIHIQHYQTLLRYVNDVKKTIDLFILATILTSVGGVLFSSIQFVVDQPIAIKGQYAIVAVTASLGLFICSYAADTLLQLGYQIGTNIFYSNWYTMEKKSKLCIIYLIAQSQDPITMRANRIFPALSLQFFSQFLQMSFKFFTSMRIMV |
|  | CcOR48 | VIGIAVLLVMLIKLIMPRIKRHYKQQVYEKIRGHFRFTTDLAEARVLRNFYDKGWRFVIAYTVINFIISGLFYGMPMLKARFNHFFNNITPKKEFPTSVDYLVDQDKYFYLILIHIY |
|  | CcOR25 | TVVIPEVIFFKEMIGERNWNKVVDCLTALIATYGAFVIIANSIFQFHKIRQTVEQMQSNWDNLDDSNELAIYTDYAKTGKLLILTYLILATGTFSFFCSIPFLPFLLDIVIPLNESRPVNNLLQVQYYLDTRKYFVPIYLHGVQAAISVVYTIITFDFYFIMIVQHAC |
|  | CcOR22 | MEETSSFYQRVRRVQTRVLRFAGLIPLEGRTHYFIGTIIMSCYVNFAFAAVSSVYVWAFVEDCINARFNPDITSELFSFVGFHFRFMYIFSRRKKLTKMLRFVEDLWKGVESEEKVHVRKFVRKVSKLSCCYSGIILTTITLYVLSSQLPQLTAESSNETLHRVLPYPFYFDVQSSPKYEVLLAIQIICLLTVTQTSVCVDTSIAFLIMIACGHFRLIQVRLSLVARDIEDNEEERRRPIAAKDEIRNNEEGVTGVFNVQTTDEGIRKRIRDCVAYHSEILEFCDDIYALSSEIFMIELISTTYNLSLIGILLAGNMPLAEKFKFAPVLLILTTQLFVCQYPPDLLLQESVGVADAAYMIPPFRNDRLRVDRLLLTLLTRSQIPYQLLAGGQIKLSIESFGNMIRGAVSFFTVLRNFN |
|  | CcOR41 | ITRTNEPVHMLQFFMDTVSIIFRLFYICWPGQKIVDHSSQFNYVLNNIPWYNYPVKSKKLLLLMLLRSSSPCYITAGKM |
|  | CcOR62 | LSKQSLFSAEFFAVFVYLCCAMVQNHFYTWYGYKLTQFTAEIPCTIFTMDWTILHIKSKKMLYFLMLSASNSITLFHNFIVNLSPEMFLKMIKLSYSAFNLLQ |
|  | CcOR57 | VFQYAYYSQWYNLSINSRKMIMIILQNSMKPVALSAASICDLNLEMFASVVKTSMSYATVMLSMQQQ |
|  | CcOR66 | SFHCEWFNNSTRTKQLVAMMMMRSTVPCSFTAKGLCVLDLETF |
