# Supplementary material for: Transcriptome and Expression Patterns of Chemosensory Genes in Antennae of the Parasitoid Wasp Chouioia cunea
Source: PLoS One. 2016 Feb 3;11(2):e0148159. doi: 10.1371/journal.pone.0148159 (PMC4739689; doi:10.1371/journal.pone.0148159)
Supplement: S10 Table — (DOCX) [file pone.0148159.s015.docx]

S10 Table. The protein names and sequences of GRs that were used in phylogentic tree analysis.

| **GI Number** | **Name** | **Protein Sequence** |
| --- | --- | --- |
| 283135108 | NvGR2 | RQSSNEDEPECFHRAIGNILLMSQFFGILPIRYIRSSSVRNFSFYKFAPRVIYSYFVLLAISVMTSISFLHLFRTLNANSFQTKGGIADATVGAMFYGNSLLGNLMFLRLCPKWISIQHDWRAMERLIDNNGKWKGPVLRWRFTLISSTILSLALLEHILSMVNNTPSDVWFGKKNLEDFLIIYTNKSHRFIVRNVDYNFTLGLFIFFISKVSTFTWNFTDLFIMLVSTGLAERYKRLNARILEATPAQLSVTDWHELRECYAVLSALVKKVDNEISGIILLSFTNNIYFICLQLLNGLSPSTAEHPIINSIYFFGSFIFLIGRTTAVTLLTARINDQCKLILPILYNCPSVNYCREAQRLQQQIATDDVALTGHRFFSITRNFMLAVAGAIVTYEVVLLQFNIALQRDEELNNMAAGSNGEKRKEGLYNKTRRA |
| 299528643 | NvGR18 | QTFPAMRRSKCDDLFLKCLFYVLKFLGVAPMAIDNSPAPEKDSPRYVRFVASKLGVFYNGIIACLTVYPSYMTVRYLTSSEYTKNIELEKVIDEAQSTFAMITSTFIIVNICVRQKRAAILANQLSSIHSVIMDLAIDVGDDVRRNTIISYIKKIVFVNLVTTIVWVASTPPEEYQYLSYFIVMSFYNIIIHAMLLQYSLVLKLLQQLYRSVNADLSSLLKKSSSISDDNNCHLMLKRLKHLRQIHATLCQISQDVSNFYSLPMLFCVTHVFLTQIIYCYYVVMTLIVWNINEKPILVILNCVTLVTLLAVSMTILVRDAGVTAVESKSTGEIVSGSIDDCQDHEIERQLNVFSNYLLHKDVHFSVFNLFPLNESLLISIVGSITTYLVILLEFEVDSTKK |
| 299523203 | NvGR51 | MGDIFEDMEKPFLPMLISNWIFGIGIIEYPIRRQLKLLSIVYSTLILVVYAYLVYICHAHIYLTAVAQIKPIIEMLHYYTNILITISIIIFGWFQTQGLQKCMLKAAQANLLMQQIGIFKNHSNILKNELRKFMAFFLFILSIIIINSTVTFYNFVPNYQQIIFIVILQNVPLLYGYIADSSFLNIIGYAYFKFDSLNKLLKSISITKADNPMHKIIAKQPFYEKVYPQVYSSIDYKDYTFLIKKIKLAHLRLVKLCREANNLYSFHILLSIAIAFVMIINKIFNIYVVLNDDDIDEGSKFRTIVRSVNWLIYYIVRNLTSCCLCTTVLNTATKTGDLICELYDEPYITENTRAEIRYFNIELVQNKLEFSAYGVVNIDLTLLQVMASTIATYIIIIVQFQKLHFVPNALVGNQTNTYRI |
| 299523199 | NvGR49 | KVSPPASTSKKAPISFRDTCLPILWLNRILSMVVIEVPEGRPWLTLSIIYATAKWVGYGYLLWYTVKNDESRINSIPIMAAVFQVILYVNVVIAVLSTYLGLANYKKYELYFKKIELADETLEIFGIDPEYSSGFKDYMKITAIWSLGGALVCATDFAIACYTFSSVPYAIIRILVFEIPMVLNPMVELNFSLMINAIGTRFERLNALIQSVAVTPLQSMNSRNFDKYQNILNRNQSKIAVKPNYYYYKNRNNIELLLRTARQLHLDLCATAREVNDVCSRQMSMQMAAKFLLITGFAYCLYLIYNDPNIPLSGKLQHYVSLGAWIVINIARMIFVVRTSVNAQKTSQIAHEIQVSKSQSNLIDEIHQLSLQIMQHPLFFTASGLLVLDFGYVRGFVGSVTTYLMILIQNQSDMLKAATTLANPNNDTSATTPSP |
| 299523195 | NvGR43 | MILRKSTEDIIFFTTCLFYYFKVLGIAPISLYIKSTKKSASQCVVFTRSNRALVYDVVLILNLVTANIYKILYLCLRVSSTKIITIEAVTNCLEDFVTCLSAVFILIIICFSREKLSAMVNAISGLTECLDGFGVENPKKHKLQLEIGMIILVNITTWILVFVTTAVAEFSYLLYDTIMYSNVIVVNALLIQYGVVLKLLRHNFKMLNENLLVISQEVPIKIQSPVESNRRVERLSQLRKLHASMCKVSRDVSNYYSYPALACVVCVFYTLIYTCYYLTRPIVLYDQNLRGDMFVMSLVYGLLLVFSVVILTKSVTATIDESDRTKEIINAGLLRFEDDEKMSKKLNQFSSYLLHTDVKFKVSKLFSLDDSLLTSMASSIATYLVIVLQFLQK |
| 299523189 | NvGR42 | MFLKCLFYAFKLFGIAPMAIKALTSKKNKACHFLFVSSRLGILHNCILLCISISTIYFIIDDTLSRSLFTDKSNLELVLDTACGICVALTSVVILLKMSINREKAIIIINKLNIIYQRKVESDKKNPSILLVGSVKIIIFSIFIPTIFAAIALGLEQSSILISCLPFTTYQMTIIQYTLILKLLHYLYQSTNTELQSVLTSKVPISIVQNRLLGMDTQRVSTKIELLREIHVVLSHLSNEVSGFYALPMFFCISNKFLVLIQYCYYIATVLSHKQDTTGQYEIILHCMSFSTVEALSIVYLTRAAGLVVTESKRTGEIVSQLIVDCPNKLVLKQLNGFFSYLLQVQVDFSVFNLYQINESLLTSITSYITTYMVILLQFKETSCRSGSTEHTENMSTPPSVD |
| 299523187 | NvGR41 | MFLKCLFYAFQFLGTAPITIKALGTKKNKACHFLFVSSRLGILHNCILLCISLPTIYLMIEDILARSFLRKKTNLELVIDGVCAIYVVVPAFAFLLKISISAEKAIMIINKLNVIYQKRKIEFKKESPPVLLLRPVKIIVFTNVVPLIIGCFVSGQHHISNLLPLPSFLTYQMTFMQYTLILKLLHYLYQSTNTELQSVLRSKVPIFIVHNRFLGIDSQRISNKIELLREIHVLLCHLSKEVSDFYALPMFFCISNKFLILVQYFYYAAAILSDKQETATQYEILILTIWFTIVEALSLVVLARVAGLVVKESRRTGEIVGGLIAECPNKLILKQLNGFFCHLLLVQVDFNVYNLYQINEPLLTSFTSYITTYIVILLQFKGISCPSDSTDHTENVSTPPSVN |
| 299523158 | NvGR29 | YFSIDLLYAKCLYYYFKCVGLATMSVSFKSTVENKKVPYSLFSPSKIGFLPNLVIVLIVIGTHFFSLKMAFEVDEIETSVKFDRTVESVRLTFGVGVSVFILVFFCAKQEAAIDIANNIKKASVLSANFSTKTVSQKELFSVYRATGWIFSAHMVIWFLIYCSTPWSFGLMIYYVSLNIYELVITSTLVQYSILLKIVRQIFRNVNANILDIFGDSCAIDFHTVGTIGNNRSEVRFRRKMRKFSQLKDLHISVCDVAASLGQFYSIPALFCIKYEFISFTFYFYFVTKLFTGMYHETITIHTIFFYVFGILHFIVPLIDLVGSTSAVVNEGKTSVELISKWIEVVKDQEQSTVRMSHFPNYFAQKKLKFTAAGLFPLDGSLILSIAGSITTYLMILLQFEGIKPYSS |
| 299523137 | NvGR15 | MVKGTKYLFDIFKVFGLATMSMTDCTKKNNFKNRKMFSYSYHGIIYNGVLICFLIIAGIYKMYYIRDKLIDQSRMSEVIDVFGNFIIYAVSVVLLSKYMISQTLAVRIGNNLYSINLVLKRFNLKYKNQYMIMHYKLVLLFDITIWLGVIIIGSFSDCTFIAAILTYIPNFIINCLVIQYVVIIIFIYGEAKALNNQLRKYVDRAFSNTLLYQFRRPVLSVHYYLPENNEIILLQKSCLSIYEVSNNVSKFYSLSILICIVKLFFSIILNTYFFLKPSIFGKSMITSTMNHVWSISWLTLDTFSLCILTQYITMTVNEIKKTGDIVHQILRHSTSLGVIKQLNNFSLHLLHKNIQFTAMDMFSLDCTLLHSIVGSITTYLVILIQFQENSSEKHKP |
| 299523126 | NvGR11 | MKTTIINCILIKCVFYFMKLIGVCPFVLDKKEILKSSTSGKMYNLLLIVSYIYSYVIVIKCRRNLHYSEETQLGIIIDMIGITLKYSAVIVCWYTLAVHQTQVKSIIQHLKVIANNQTMLASKCRREKINNEFKTFRYGLIVINILGLTILTQNNFINNYYKNCTTDFTFTLFDIFQIVIYNVIFIFLRIVLYTQENYRIINKALNKCTNYNELDNVNVTYDTILSLKKLQSAGLAHKNISDLLENIVDFFRLPVLLIITAVFVQILIDVHLILYFIKTENWNHIKYYSLIHLLITFAIRVSATYFICSISDSTGIEGNNTKNIINVILNKWRFTKSHKNLAKMFIFNLHEHKIQISLYGLFNVDYSLLKNLCSSTIMYVIFMFQLDGIIK |
| 299523122 | NvGR10 | MVNLSDIKPLFYAARFFGCAPHRVTDSDVLLTTSGLIYSGIWALGFVCCCCYGLRLICAGVYTGERNMLALTAVRTLLAYVCFLADDALTMRWNERLRSALLQLRNFDVAVSYGRKRSVNWKLRCCCWMLVGTIIAYWIGVGYVTYKCEMTNPLFNAITYVIANAAISMQLIKFAGLLILLRQRFRYLRELLPLEAAHPNSARRAIQLQDIWWLHCSLANAAETINSCYSLQLLLWIFTMWLNALSRIYAMNETLVDSGQFLLMLRESLLVTACIGNLMLIALACHYTAREANSVGRAAFAPQTSFSRKRSLLEHSLEVGVYFSLRQLHFSAAGGFIQVDLPLLLSIAGAMTTYLVVLHNNS |
| 299523092 | NvGR7 | KNSSSGSSPSSSIQDIRPTFLIARVFGLAPYAITNSSINVSKRGILYSVPWLGFYLYALYNRLNLYTHSDLETKFRILSVTRTALAVIALLVDLVVCTFRDDRFQNALDCVRKYDLAVKYDVETNARLMRIHSWTIYSFMITYYLAIGWFTYVDEPYEGVMAAVIYVYLYLPLSIAVMKFVALITSILLRFRHLHRMLLPGFLSIMMELDSEPKRLHLRDVCWLHSCLCAAAANVNSLYSLQLMLWFANLTFNTISRINDFGQPQNSIDAFKLARDAGLVLIFVTLVFFIAGVCHVTSTQANKVGAVVFSPGSRYFRSRVVDHQDKEDKFYIGQYFALHPLHFSAASGFFQINLSLLLKIAGAMTTYLVILKSPSNC |
| 299523065 | NvGR45 | KNMLFGKQIKKFDYLKLLYYYFKVFGLATMTFVTDSTKTTPNRFGTFSRSKYTIVYNVVIILVFVMPCLYNMTIFCVGTNRVKFEDFADCIQINMALFVTIFILSKFCISSDSLISIANSISRITESLLTLSSISLQKRIKVSFEIKQAFIVNITMWIAFIVINLSEIEPWMKNAVNMYVSNFLVSVLILQYSVILKFLQYDFKILNENLIEFRNEDSMKIRSPTETKAKIDGLLKLQKLHESLSDTSRRVSMFYSYLMLVSVLNIFIMLIFVCYYLAKPIILTHDSNFSSIMLLRCFWYGLLFVVLLVTLTKFVTATIEESRRTKEIISSCLMIPDADEKLLNKLNQFSLYLLHRDVKFTVWGLFTLDESLLTSMAGSITTYMVIVLQFQQKD |
| 283436203 | NvGR58 | MGKKPMTRPILPLLISNWVLGIGIIEYPIGTPRPTFSFIYSTTLLVIYCTTSIMIRHEIFRVSIILKNNTVPMTIVFYTNIFLTISIVTLGWYRSKGLRRYVAKAAVADDLMERIGIPNNHGKMLRAVAGQVIKGFFLVTVLIAIIAVIVLVEDAPLQTKILISSVMSFPLFTMFVSDAMFTSCVRCACYRFTELNKVLKAVLTSTHAFPQHKRVCSSVFESGGQDSNFVINNVSQRKNPAVIVKLAKEIHLQLISACQEINNTYGLHLLLSIIFAFAVITGNMYLCYMSSRNSNIPHYILVKTLVVSGIWIVHYGMKICYFSIVCGCCTENSIKTGDYINEFYDEPSTTNETKLKIRQFNMQLIQKPCKFTAWGFVDLNCHLIQVMIGTITTYLMILIQLGTTTYVADDSYSKYLKSFSSLTY |
| 283135122 | NvGR47 | MFFGKPINKKIHSFKLLFHYFRALGLATMTCDLAPAKNATKYYWSFASSKSAIVYNVILVLIFVTSNGYSMTYFCSSNYQVDFDTIADCGQTTIYSFVALFILVRSCISRNGLINIANSISQITESLQSLSSIEIREKNKVGLDIKKLLFVNITIWTGLFVSTVLEVMPWTKYTITVHISNFIIIVLMIQYSVILKFLQYDFKVLNENLVEFRNEELTKVRLPTETKTKVDRLLKLEKLHESLSDASRDVSMFYSYPMLASFLEVFIYLIFVCYYLTKPMILGNTYTTFMLIRSFWSGFTYAILLVTFTKSVTATIDESRRTKEIISSCLIIPVDEKVLSKLNQFSNYLLHRDIKFTVFGLFSLDQSLLTSMAGSIATYMVIVLQFQQNNRLLFHVPYKSCYNKKYAK |
| 299523207 | NvGR53 | MSDIINKLEKVVLSIAYYTNWFCGIGIIEYPIGKQHRVMSFLYTGVVLIVYSVLSVYVYSDFAIVSRDYEVNQTMMKGVYCATFILTFSTIVMGWYRNKEIRSILQRMNIAMRIIDKLGASKNYTKAFTVQVGYAVGTLTFLIIIVIINALVVYKRDPKHDSHVLTVMAVNYPLIILQVVDTLFINIIQYARKNMRVINDVLREMLTSTQDFPQHTKIVRRYLRTLDSPELDTDIVDQKTDAEDKMYTINMSKKAHLTLVKICQETDDTFGLHILLSVIVAIITITVSIYHIYMLVDYLSISRAIYDNTLLPVCILLIYYYVKIHAISHFCSSTSEEVAAVITGDIISELYDDSSIGIESQTEIRQFGDQIVQNSLTFKAHGFVTLDFTLIQNVVGFVTTYLMILIQFGSSTSIEISR |
| 299523162 | NvGR30 | GNMLIKNLSLNETVLEKCILYFFKLSGIATLNFDFKLSTNRSKKFSSTFTRSKTGIAYNAALISLITIVTNYLIEFQINHNMYKNFYDKLDIGYAALISVTAVLILVKFSFQQEKTLTIANELNEIRDSLSLNDCSVDGKGHALRRFIVLVFLAHFLFLTILFSTSYVLNNTTINIVRTTLNYIAIYLSNFIMHSMMLQYSIILKLIEHLSRGINDDLVEFSRPPQGLNSLTFTKKTTSQRVGQLANLRKNFSSLCKVSQDVSEFYSWPMLLCLSCNFIAFVRAAFYIAMPIVHGTDAFTANIYVRCCCYISHNAFSLIILTKSVTASMTENRKTREIVNDCIENCDDQEILKKLEKFSCYLMHKKITFSVFNLFSLDESLLMSVIGSITTYLVIILQFQNNNAE |
| 299523155 | NvGR28 | PKKFYQNARISDLWFAKCVYYYFKTVGLATVSLRLKSVKKNKKNSSSLCTSSKLGILINVVLSLIVIAIFSYTTIVIAEGTFKNSLKFDRAIGVIRIILGSSAALIILITFSCKQGSITEIANNMQVLTIFSVLSANFKTKIGNTNESFSIFRETGGVFFVNIIAWLLLFVTVPTTNWKVFAVTPYVPEVIMTSMLVQYNMVLNLVKRLMEVVNANLLYTSQYDDKYEDNQITMIKNDRNENSFKRKIIKFTQLRDSHYMLCDISEDLEKFYSRLVLLCITYIFGSLILCSYFNTKEVLKQGVEFLTLRATLFFGVTVIHYIMPLVNLTRSTSAVIAESKRTVKIVNRWSGNFHNQPEIAMFNQFPNYLDQPNLEFTAGELLALDGSLLISIAASITTYLMILLQVQDTSPN |
| 299523142 | NvGR21 | MICKPHYTSDFFFLKCLFYWFKIFGTSPMGIDFTSAVKSNDVPQDVHFVFSKLGILHNAILVCLAIIPSCVTIKEAYHTEYTDRLQLERVIDTVHAVATIMTFYFILINVCINQKRAVAIANQLNSIYSQSTSLFSKSKIRPNRAFRSVKKIVSIHMVLMLLLCLFAFTFVHQNLIYHLFINFTNVTIYVMALQYSLVLKLLQHLYRSLNADLRSFLITRDRRDPSSISLPIDELQCRLMQVYEIHASVSRVSQDVCDFYSLPLFFLFAIAFFTLVLFFYYFMLVLLLMNKMIDYGFVLPFFLTLLGLTILAISTLARIAGGTVKESNITREIVSESIMNQRNQPISGQLRDFLHYLQQKNAKFTVFDLFPINESLLMSIASSISTYLVILLQFKESNSTQQTAPSSAT |
| 299523134 | NvGR14 | MKKTLYVCVYYFVKILGLCPFTRKKGRFVKVSHVGMSYNVLITILYSRAFVKAIQNRHSIRLSQETPLAVIIDTFTHVLSYSTIVSSWLVCAFRQKIFIKVFQSFKNVENLENDLLPSMHCSKNGLEENLKEFRARFIVVNLICIIFTGSTVVIISMCEDMKNQSWFWFIYNIPINVTFNVAFILTEFMRCLRKHYKIINREASKLARSRKRSFARMPMAFSKKLQTIGRIHSDLTELGETVVKLFSLPVLMTVHGHSANIILAIHGLYRILKSDQSMGGPCVLYAPTVKFMIYSIIIFFICSIPVSTCNEADKTLRIIGQIPYEWHEEEMHNKMIKNLMFQLYQKRLQVSIFGFFNLDYSLFRNVWIVIIMYLIFVLQLDPSSFIFAILK |
| 299523089 | NvGR6 | QKKSPGNEESPLYEVVCPAVYLARVFGLAPYELEDARPDNNRPKRLGASTVYCFFSIFWLIVYTYIVVISLIRFGGLDRDKPVLGVTEDGKLILNYLVSMVDTLLCIRCRERFVHVWNSIQDFDESFQLGNVPAGRDSPRYSPILRRARFWVWTILTTNVVGWTMINQLGMHAFGEPYLQNIGYMLTYVGTCVAVLKFVGVVMLLGQRFAYLNEELARQRKKEEGRRSRAADEIVKKIESSYNKLLSTSEELGNIYSFSLFLYLLNLFCHAVSNMYFFTIWTILDPGYLKNPKIVFCLFSWLLIYLVQMLLIHVACHFTSLEANRMASVLLDWRRQAYRQSKYEFSSTLHYLNRRLNFTAAGCFNVNLPLLTSIFGHLTTYLVILLQIPDSSNS |
| 299523084 | NvGR4 | MYKDLYNTLEPIMWVWRFVGAYPFVIRGPIGSQQYVLSTYSIMLSLIFLIVTLQYCYKSVDFINQSATEYSLFLITISVQQVSNVICFFDSVVLKLFFGKKITRSIENIAIQDKKLSKIGCSLNYSQAVRKGRLYIFILFIFLCFHIHSELVYFNGGAPLLLRIFGNYLAMLQVANVILFAWLMFNVGLRFQVINCKIKTCIFDIEWADQNSSCLLVLRTTAKAHAQLCQAAKIINGTFVLSIINCVLSAFIFTTTMLYYIFMELKNTLPFSHAVYYCSVILIHATTIVIIVQSCNWVHRNAHATVKVLHEFSKENNRFDDDKHLNQIIHNFSMQILHHNLTFSAWGLFPIDSTLLQSLAEAVTTYLVILIQFDPLVT |
| 299523068 | NvGR46 | MLFLKKSNNKNLDYLKLLFYFFKVFGLASMTIDATTAKNTRNHFWTFTRSKSTVIYNVIFILVFVISNIYSMTFFCRGTYVVNFETIGDCGQTTLSLFVALFILTKSCISRNTLIIIANSISRITDSLLSLSSTSIQENSKISSEIKKMFIINISTWTVLFGTFAFDLKPLTKYGIVVFFSNCIINHLVIQYSVILKLIKHNYKILNENLIEFGDQESMAIRSPSEAKIKVDRLLKLQKLHESLSDTSREVSNYYSYPMLVCVLHVFIILIFVCYYFFKPMILHSKNLSTFTFLRTIGYGFAYGLLLVTLTKCVAATIDEQNRRTKEIIGSCLMISADEKVLNKLNKFSTYLLHRDIKFTVLGLFSLDESLLTTMVGSITTYMVIVLQFQQNLKR |
| 299782493 | NvGR23 | STHCFLFNFDEMFLKSLFYFYKIFGVAPMTLDSTKNRPSDVSFAYSKFGILYNLFLVILTIFAFYQCAILVHFDVSVGMDFQRFVNTTHLIFFVFTTLIVLIIFCVRQERAVALANRLSSIYYLNKNIKLSAILPSIKGIVSMTFITTIMWLVTTPYDDAHLLTYYVAISLYNFVINSVFLQYSVLLKLLYHLYRSLNTDLRSLLESLDIAIEMDRHSNNEIKLTSGRLKRLREIHMLLCHLSGDVADFYSLPIFFCITNAFFVLIIYSYYVFRGFAIAIMPVLVTVHCTTMMVVIVVSLTILLRTASVTAAESRVSGEIVSESMASCSNQFIGRQLEVLSIYFLHKNVKFCVFNLYSLDESLLMSIVGVITTYLMILLQLDGSSNCK |
| 299523205 | NvGR52 | MVDIFVDMEKPFLPMLVSNWIFGIGIAQYPIGVPHRVLSFTYSLLNITLYCVVAFFAYPYYIKFIDVTKSTLTTMIFSFSISILLTIIMITSGWFQAKGVRKCIIKAAIVNYLMQQIMIPKESTVIFFKEFIKFLIPLTSIFLIITFNLFITFSESFAHIGQIGANFTMNYPIIVMFIIDSSFVNIIGKVKNYILILMYANFKFIKLNELLYSLLTSSADVPQHKRTFKEFIYKEKNSKFQYLKSIRKDYSDVIKCAKQIHLRLVELCQHASNTYGLHFLISTAFAIGIMIYNTYNIYNILASIVITSAEYNIHIMKPILYNCNWLTYNILKIVRLSWFCDSICKESVRSGDIASELYDEPFISENTKSEIRDFENELISNKLTLTAYGFFHLDFTLVHAMICTVATYLMIIIQVKSTCR |
| 299523201 | NvGR50 | RNNKINPRKGRPILNIADSFRPFVWINGLMGFGMIEMPYGRPWPKLSVFYGLLRAIAFSILSWYVFKNIQPNTRISALMFLIYKIIIAASVGAVVISSIMGLINHEKSKKLYKKIKLVDETLKMFGVEPEYASDLRRNRNIIINYYRAIIVLFIIKLGSSYIFSREAFCTRNILNVLYFNIPSIINPLVDINYTSKIHVLEKRFERLNALIHNVTTSPSKMMHTHDFKKYEMILNNRGVISVIPKYCFKNRNNIEHLLKVTRQLHLDLCNTARNMNNLIYTQMSAQLSAIFVHLTAGTYCFYFIFNEKTIIPLQAKIHSYVLLIFSIICGIVRIILITYATAGKISKILHEIQIQNTEKKLTNEIHQFCMQLKQHPLSFTVCGFVELNFSYVTGFVGAVTTYLMILIQNQTDMIEAAKTMVDPIKSNSTSVTPA |
| 299523197 | NvGR48 | RRTKPDADALPSVENVTRTLTPVLWLSRFSGLTVFEMPAGTPWPKFSAAYALFLCSAYGTMIWFGETFIVKETTSVPLVIFIYALVKYVNAFLAAVSFAVGLLHYKKTMKFTKRLKHVDETLKVFGIEPEYAASRKENIRIVLIWIVATVFQIIGDAAICFVIYDPAYIAILKFFIFHIPFQSMSLLELTFAMKISTVRSRFEKLNALFQNVLENPVLPMHFKHVNKYHNILRRQHSEEGDRNRKNLELLLMTSRQLHLELCAITREINEVYGKQLAMSIAAKFIYVTGYGYVFYLYYNEPSISLSIKILNCTYIAYNLTFITVMMIYFIGQSVAAQKTTQIAHEMPVTQSQTKIIDEIHQFSLQVTQHPLEITAASLFTLNFAFMRGFIGSMTTYLIILIQYQPNIAAAAKSMIDEIMANMQNVSAFYHFANFST |
| 299523185 | NvGR40 | MFIKDFINKKDKVFVKFIFYYFKVLGTATISFNTESTKSRKRNEWKFTHSKSSIMYNIGLIIFVTTVSSFGFIYASFQHQTNFKKFERVTDRAEDVFNILSVTIIMMMFCFKYKNMAGIANKMSMIYQSLISSCPQTLYKSFTDNILLHIILIISPYIIIWPFIIVFNFINFPEFEIYNFTIFMNDIVITALLMHYSTVLILLKYFFKMFNVRLSFMLEEQDYLCEIQYLNCNNRSKGKIKELFHMRKLYASLYEVSQDVSSFYSGPMFLCIFKILVSATLSLYYVAKPIIIDSHTILNVEIIRNSMFGLIYATALLIFTTLVTQTARESVKTREITNRCIINFENKYIIKELSQFSTFLLQADVTFTVYGFFSLNQSLLTSMTASMTTYLVIILQFQQNN |
| 299523182 | NvGR39 | MDILKITLNFFKMCGLATMRFDAGTTQNTSVQSSWCTSSKKGQVYNLFLICLITASNGYVATIVYEYNISTQEFDKSFDVAQYIYTSATTVAILMLYCFCQGRAVSIVNNLKIMHKLVTNVNSRLSKEEEPTMGGLKRIAIMTTVIWFVVVFTSCNLSFGVVMYYLTLYPCILIINCTFLQYTMILHLLKQLFAILNANFRYVSRQSIVPKIAVAATNSMLQISTKKTQPFSDLCELYTSLCDLSMDISKFYHLAMLFCVSHVFITLTTWLYYITKPLVTGAIELSIIDYTHSLLILMHNFFMLFILTKSVDAVVDEKTGKITNRWLANLQDQHLVNELNLFSNYLLHKNVSFTVYGLFSLNETLLMSITGSITTYLVIILQFQSGVQK |
| 299523179 | NvGR38 | MLSIKHMFYFFKLSGVATMKFNTNLIETGRVQGSWFSGSRKGIAHNIVLICLIFVFNCFGVRTVYYWSHIQFERVIDVVLYAYTTVIAILILIVYCFRQKQAIAIANKLQILREFTMSINGQLNQGEQLVVSSLKRISVAHLVIWFALIMSTSTEMYMLVYSIATYPCILIINCTIVQYSVVLKYLRQLYVILNANFFNCSKQFSANTSSVRTNSLSDLRELYMSLCNLSADVSEFYHLLMLLCLSYLFVTLLLWCYYIVAPIFVKATVLPILYGYVRSLIIVIHHVLMLVILTQSVSALTRENKKTGEIINKGISNLGNQRVLNELNMFSNYLLHKDMKFTVYGLFELNESILMTFAGSITTYLVIILQFHSRD |
| 299523175 | NvGR37 | HQSFQYKLSEKNMITLKRIFYFFKICGLATMKFDSNMAVKERLQGSWFVNSCKGKVYNAILIILLSIATYYVTAFVYDLSISSGEVGKLFDIVSYVFTTITTIVILAVFCLCQKDAVLIANNLRRTHMLIANINSQLSNEKINILRTAKIICIVNLVMLILVFITTLRQEFGIIMYYTTVYPCLFVINFTFLQYSLILQSLKQQFTILNRNFHYVLRQCIMQKNLGATGSSFQTHKVRAQSLSKLCELYASLCDLSTDLSKFYYPTMLFCVLYTFMMSTTWIYYTVEPVIVGKTKLTTFKYIHSLIFLIHHISMLIILSKSVNAVVLENKRTGEITNRGLANMQNQQTINELNFFSNYLLHKNVSFTVYGLFSLDESLLMSITGSITTYIVILLQFQSSVQ |
| 299523172 | NvGR35 | LFMILKNWKISDLLFLKCVFYLFKLFGLATTSIQTKPSTSRFHLPLFTRSKLGQIYNVILAIGISFVYACLIRWTIKHYEHHIISRSHQAIDYTHTTMAMITAIFVLLVFCIQQEKFLNLGNRTVRLGELLVGFYEVQRPSKQKTLTKHVKEIYIMVGMTWLSIFVTTETGGYFKNVVYFSMIYLCNQIITLTLLQYSAILRCLQQILWFVNENFLQFSKEPCQMDKIKIQFSKLRELYLSVCEIAEDIERFYAKPMLLCIVYVFVTLIFFASFITKPMVSAVVISDFQICHCSFRILHYVVALIILVKSVTAAVSESKRTGKIVNKWLGDCNTLQVDPKIYHFSNYLLHHNLHFSVLELFSLDGSLLMSITASITTYLVIFLQLQE |
| 299523170 | NvGR34 | LKMPFNNPLTSGAAVLKCVYYVCKACGLAPIAIRSNDGRKLRFPPFEHSKAGLLYNAVLILIILSMSAAIIQCTFVYRTKAEILKFDGVIDVTHNTMASVTAIFVLTVFCIQQRKILELANRMRVLGEMSQSLCDVEICGGRKRLLRDVMMICLTTCCTWLSIFFTTQVESYKGLLYFSYIYLCNLIITLTLMQYSIVLRLMQQILRVVNANFHHFSTEPSQRKLKVQIIETSCQGIGRFTRLRELYLSLSEVAEGFEEFYSQPMLLCIAYIFLTLIFYAHLITKPMVVGTRSVTNPQLCHCVFRIMHYVISLITLAKSVSTVITESKKTSKIFNKWLGTLDSLQLDPKLYLFSNYLLHHSLQFSVFQLFSLDGSLLMSITASITTYLVIFLQFQNHQTTDS |
| 299523166 | NvGR33 | MFGKFDETILEKLLFYFFKMFGMATMKYEISTIKNEKIKKRRLFTYSKIDIVYNSFLILVTTILNIILVGLKIDDKTSLPNVRGVQKITDVVQFGFATLTCVFILVYFCARQKKAIEMADQICQIYGSMIVNNCDTGEKRSILLITLFVFSINFIFWLVMIITSVNVTMATPNNLIFYYLAVYSCHVIMQTLLMQYSIILNMIGYFFLHINKSLVILLKKPNQLFLDAQCRNIDKTRGERLLKMRKTYLILCKISEDISDFYSPLMFFCLSVTFVTLIRSGLYIAVSIADKESNLTIKGMIHCIGYVVHYYFLLVMLTKKASKIVTESKRTGEIVSDCVHYVDNQEIILNQFSNYLLHKKIKFTVFNLFVLDESLLLLFAGSMATYLVIMMDF |
| 299523164 | NvGR32 | MWFSNFHDTREAFFVMFVYLFFKAFGLATVKFNFESIKKTLIAGKEASEVLKPSRVGIAYNMLLIIILSILNYVAIRVSYERPDFTDRSELEMKIDTVKAVTACFSSFIILLIFSFQQEKYVLTVHEVLSIRQSLISINSAIYFENESIWKIITKLLIFMFVNWILLFITIEIQNDYQFLLYFVTTNLCDMIMTHTVLQFSIALKMIEQLFRVTNANFDHDSKASFRLNDEICLNVALKKVQVILNKLSRLQDLHLSLCNVFEDLAGFYAQSMLLCVWYIFVSMILSAFYVTKPIITGNTGLSVVMYLRTVIHFLHHTSLLIMLTKCVTDLIAEREKTGKIISVWLAKIDNQQFEKKLTKFSIYLMHQKVKFSVFGIFSLDNSILLSIIGTITTYLIILQQENLSSNSNNSNCH |
| 299523152 | NvGR26 | MHIRNIHCKITIGKVLHYSLKIFGLAPFSLDVEFLSNNKNQVSSEALTCSQLGVIYNLILAVLIMVITYLTFKISNKTHIFGSGTDLDMAIEAIKTVWACISSVIILFLFGTQQKKLVQCGNIMLMIRERLITINETLYLENKFLWKSITKISLVVIVMYILIIVTLGGYIDLARLIYVIGATLCDVIIIFTVIVYGIKLKMIKQLIKIINANINSISTEFNRASNNILQNNEMKNAQIICDKLSRLQNLHLLLFNATEDLTNVYATSILLCTLYIFLSIILNLFYILKNVMTGVASLSAILIRHIFIQFIHCTCSLIILTNSVTDLVLESNRTGKIVSEWLTKLKNSRVENEVTKFLLYLKEHELQFSVFGIFSLDTSMLLSITSSITTYLVILLQLQFQQN |
| 299523149 | NvGR25 | VKMLHKNIVNKESIILKFIFYYFKLVGLSCVSFSSKSRLDICFLTSKLGALYNVILALLITCFNYYVVLIVVKVSFGSLHFDRAIDFGRVCLAVISSVFILITYCFKRKKATIILNRINTIAELSVNLRSNGKSGHDGLCKPAKRIFFVFLVTWLVLIAVTPKLKFYALLYFAAQYSCEMVIMCMLVQYSMLLSILKQLFETINARFTISSEANFQVRRFSQHRSNEFGLEVKLNRFSYLRELHLSLCEVAEDLSQFYSQSLLFCIAYVFSSLVLYAYFFVKIVTQKGDGIINTATTRFIIVKLLHYIGPTVTVTWAACAVVNESNRTGKIVNKWMGDSRDQYVAIKLNQFSNYLLHQKLSFRAAGLFSLDGTLMMSIAASITTYIMILLQFQDSTKR |
| 299523147 | NvGR22 | MFIKCLFYAFKIFGLAPMVIDTTSTEKNNKEAHKIIFLSSKLGVLYNAALAILITLPTYLAITFAYSDYVGRLEFEKITDTIQSVFTIFTSVFILINVCVHQKRAVDLANRLQTVNYLSMTKVSCSDKSVKLLSSIKRIVLANAVTTILYFAVTPSNETRVLIYFLVINVYNTIIQATLMQYSLILKLLHHIYRSLNSELSSLNKSLIFVGEFNQFLNNSVQTILRRLQTVWVTHLLLSHVSREVSDFYSLPMLLCLSNAFLTLIMYSYHFVRALFIWKEHSGNMVVLILRTFMQVVTVAVSLTILTRAAGLTVSESKRTGEIVSESTVYGHNRQIRSRLKEFADYLLHKELKFCVFNLFALNEELVMSIAGSISTYLVILVQFNETSSIEN |
| 299523140 | NvGR20 | MIVSKCVFYFFKIFGLATMRLDENETVRDSWCSGSKKGQVYNAILTCSIIASNCYVARLVYKENLSHREFEKTFDVVQYVYTTVTVAVILTVFCFCQGRAVLIANNLRKTYVLVENINSQMSKKEEDPVISGLKRIWITSTVIWISVVFTTSKLQFAVVMYYMTVYPCILIVNCAFLQYTIILHLLKQLFTILNANFLYVSTRQSVVTRKVEAAHSSFQAAEQSSQQFSDLRRLYMLLCDLSMDVSKFYHLIMLFCVTYVFSTLTMWLYYITAPLVTGTTPSKLQYIHSLMIVTYHIFMLIILTKSVDAVVQEKNKRTGEITNGWLANLQNQQLINELNLFSNYLLHKNVSFTAYGLFSLDESLLMSITGSITTYLVILLQFQ |
| 299523130 | NvGR13 | MQSRHTYTKLFVRCFLRIIGLFPLFLDINGNPIFSYLGLFFNLCLVFAYIFMSIIAFQKRMTLVLPKETVVAQIVDMIADGLENLNIISCLLVVAFRQKCLVKFYEKLKAIDLRLYDINVKHCKTFDINMNIISLGRKLTIVGISFFIICTVDHLRLLFDNYLLSIRFWIAYQSTKIVIYNLIIVFCETMIFFRKSFAKLNLLFQQSSSSYDKIQDIGQLHKILSELVDDFVGFYSFMVGSTIVHNFIHLSSNMYRIYLFLKFNGDWSLLDFLDFTSIMIWLNVKILILYFLCALPAAVSEEANKKSIFIHRMLKAVRENDLTSNNKRIARLLTILYQTNLEISVYGVFILDFKCFQSILTTSTMYIVFMIQLEHLK |
| 299523128 | NvGR12 | MKKVNGIHIIKTLYFYEKLMGLCPFILSNKIVIKFSYIGAVYNLLITLIYTYYFILIIGLRFELHLTRESTLSIALDAFGLAFQYCSIVSAWLTLTFRQECLKKILVTFAKVNLLANNLSMTLTRYCLRKLQYIAVRLMLINLMYIVIFLSEHYLLKTYKKFEEHASTWIWFNLPKLVIYNIFGIFIELMIILQQDYRALNKVISYSFSEKIDATSFCNFSESPGVISKKLCTIAEFHENLSDILEYTTNLFSLPLLFALLASFLHLTLDSYIVYQHLLSKRMWEFNDFSSYVICLVWISTKILGFYFLCSVPDSTSAEANHTVIILIKIINNCYKVRSCRDMMKKLMLQFKQKKNYASLYGLFSLDYYLFKNIMSTSVMFLVFMFQLDDLVT |
| 299523097 | NvGR9 | GKKWKIFSATDFLSLIKPSLLVCRFFGLISYKILNGKIEQSKNCGSYCAIVTFVYICASLLILYIINVSPYMNRASTWMLQGNCFYTLVNFMLVSNFVFKSSTIKILQNLADTTAKLPSEKFVKISKWIHSKDLVLYLLLLLHVPKVFVGNIYAVLSKIIGTYAAMTIYLLDFQYNSYVFIIASCFEHINEELVQLNYNACKERGHLLRRVYHHQFNPLLFVKLRYLKQWHYELNEIIRKINSNFSLQVVATVIMTFTELTFGLYFYILDRRHKVRSLDKEIWYFYYHTMVMYFSTKLLLLTLTCQYANNENYKTRTIVNEIIISTDNKLFKEEIYLFSLQLLHTDNKFIAKGVQLDATLLTGMAKGIFTYLLILIQFLITN |
| 299523094 | NvGR8 | CKNLTFVQITNIYQLMRPYFFLYKLYGLFPYKISKNQIHSSKIGLCHTFFVAMSCIVYFVIAMYQCFYSLDIVFDTTESLMQFTSYFMLGTFIAVYSCASNKYKFLLLKKLILLSSMLSEKEFFEVAKVIYFKDIIGYIFLMGQIFNIASEDLTAQNISKMFALHITMIVFLMDMQYSNFVFLLKSCLKNVNNNLQLLTKSYEGCEIISCNKSMQLLQFNNLQLIKLRKLQHNHHHVSCVIKELNTVFTLQIIATVLMTFAEVTFGLYFFILHIQGKKGIDLDKQLWFNYFITSVTYYSLKMAVMVWICQETKNESLKTGIIVHDVILNNNNEQLKSELSLFSLQLLQCNNEFTSKCIVMNANLISGVVSGIATYLLILIQFLNTKKSTSKNNEQ |
|  | CcGR8 | NRSRPLTLKVFEVYLSLVQTSNVMLFAWMILNVGCRFQVMNQGIQSRMSKTNINNYTVSANYMFLRTSAQAHSELCKIAKRAIEPFVISIINCVIMAFTITTSIVYVIFSELKNTLSVNHALYYFTLIMTSILLTILIVGSCNWTTRKAAETMKILHKIMLANISTDNKHLDETARTFCMQIIHHNLHFT |
|  | CcGR2 | IKLVIFIYIFLWLVVTATLQYFDVYNIIIYTVLNLNEWLRMTVVIQYFIIVELVKLMFVRLNENLLGLLRPIDPLCEVAQGLEWEKQFSQLWKFHADLSKLSRDISSFYSLSMLLIIVGKFINSILITYNMVKPIFLGGQYLTEYQYVNRSMCMIVYLIPIVLLTTSVSDTLNE |
|  | CcGR9 | VFACDLTMNCIVFDDAHEAILVTIIFDIPLLTNPMVELNFGIAISILGKRFERLNALLQSITDTPMSSAHPNDANKYENILKCNQKKVAVRPTFYHQNRNNLELLLKATRQLHLDLCGISRRVNDTCSKQMSMQMAATFLLLTGFSYSFYLVYNEPYIPLEHKVQHYVSLAVWIIISIFRMIYVVRISVNVTTEAQKTSQIAHEIQVPRSKSKLIDEIHQLSLQIMQHPLYFTASGLIVLDFGYVRGFVGSVTTYLMILIQNQPDMIKAANTL |
|  | CcGR7 | TAGITSLLFLKLARQWPTFAVSWENMERELAARHNPQKQNSLNLALKFKILSIVVMVFALVEHTLSILAGYFSALECANIRGDENIWATYFMLQFPGMFTHHNYAFWKGFIVQFINFLSTFSWNFMDLFLILVSVALAEQFRQLNHRLYSIRGKTMPDWWWAEARIDFNRLATMTRRVDSQISDIVLLSFSTNLYFICIQLLNSFKPMPNAIQTIYFCFSFGFLLSRTAAVSLYAATVHDESLLPAPILYSVCTESYSKEI |
|  | CcGR14 | VTFNILFFITGTKYFYEYLNMVKRRYAFSMDDDSLSILILTIRQFFNIVCTFDAMILKIIYSKQVTLSMENLAIQDETLASFGYQFKYKPAANLSVVVVTTILIISYSTVNLEFLVLVQVPVP |
|  | CcGR11 | KYQILNRTLTNMLGEAPRHKFLMNHLINEQTKLRLRSISNTVKEATTLIKLTKEVHLQLLKLCEQVNNTYGLQILLSIVVAFGVITGNIYESYSLLQDGYVKRHLAFKSMTNSIVWTLYYAIKISNFIEACSRCKQHAVETGDLLNKFYDDPFADEQVQSEIRDFNVQLIQRPVQFTAAGCIALDTRLLQIMVSSITTYLMILIQL |
|  | CcGR17 | YIFLLAQFPHIYSNNLVLFISKMYAMYITIVVFLVDMQYMTYVMILKICFKNINNYLLKLKMINDQKSVHTDENVSCLGNSVKLQFVKLRKLQLRHHNVSNAVKQLNKVFALHVIATVLMTFVEVTFGLYFFILHSQGRKGIDLEKQIWYNYFITSVTYYSLKIVIIVWVCQKATNESAKTGIIVHDVILNNDNDQFIAEVKYISIHYLNHCSQVMNSSFFLSLNLFSLQLLQCKNEFTSKCITMDAKLLTAVCYEILLIFFHF |
|  | CcGR6 | LDNIVTFLLEGLTRWDAQYFIHIAKYGYTYENTLAFFPLFPLSMKYMARVFRIQPPILNYSNVIVICGVVINFVCFVKAVLVFYDLSLVVFKNIKVAYRAAIFFCVNPASIFFTALYTESLFAYLSFYSMLESISNNPCVFLPLSLSSLVRSNGLVNLGFPIYFWLRNLLITVLPNYVLENRHFHGNSKSLLFNFRHVFISLSQIIFVIVLSLLPFGYSQAYNYTKFCKPELNDSLLPYHVQEYAIGNNMSLPGEHDFSWCNSKLPIAYSHIQHKYWNVGFLKYYQFKQIPNFILAVPVIYLMLKCCIEFFNEHKSKFFTLEFFTGKSRASDNIKQYPLEMFVFVVHALFLTIFCIFFVHIQVSTRLLCSASPVLYWYCALATLRKTKTSKKLKEIEYESSENLYSRWKVFFITQQHYPYQEKLILGYFLGYFAIGCFMYVNFLPWT |
|  | CcGR3 | ALLVFVVVVAHPANARPYQYIPEIPGWIPVYIRQGDQPLSEIHPALAEAFHEDIQSIRLDESASKNLLADGNEVSAELKPVKENAQSIGANPEENDFPQKTKVFEEDSVVMKIPKPLAFDKAELKRLTKELKHYKKNE |
|  | CcGR16 | NAFILQYCIVLKLLCRKFNIINENLLRMSRIIDANDLLLHNKILEQWDLYSKLSDMCQAISKFYSFCTLMTVAYRFLTLTTTAYFLVKPIFVK |
|  | CcGR13 | SWVLKLSQDISDFYSHLILLCILYVFVTSVLFGYYILTPIVKGRYPVSLNDSVHCVFYLLLLAVSLKMLTNIATATVKEHRQLRDILNESFDKINDDSVIKKVTNFTIYLSQKDIVFSVYNLFSLDD |
| 861722557 | MmGR6 | RSGLMRNSDSLHVALRPVITLAQCFALFPVNGINAPDASGLSFTWGSFKILYCALTLIMSAFMTVASIIRILSTKFHTTKITTLVFSVTSCLTSLMFLKLARKWPKFAKSWEKIEGELTIRYNQPSKYSLVKRFKVVTIIIVTLAFLEHALSLASGYISARECACLLGDNDVAAIYFKTQFPQVFNKTNYALWKGIVVQCTNLLSTFSWNFMDLFLILLSTALTYHFNLLNKRLNNVKNKTMPEWWWAEARSDYNNLASLTRQVDSYVAHIVLLSFGTDLYFICIQLMYSFDRMTSVMRTIYLSYSFGFLLGRTTAVSLTAASVHDESLLPAPVLYGVNGSSYSSEVIRFLTQVTTDNIGLTGMKFFSITRSFVLTVAGTIVTYELVLIQFNNVQQVNHLNLTNVCEVK |
| 861722554 | MmGR64f | KKKQFLGENSPLYTSVCPLVYIIRGFGLVPYEFEDNQLVPCDSYMIISFFWLFMYTYIVSGFIIEFIESEKNRKKVLLYAEQARTVFNFAVVISDLLLCMRTRKEITWIWNKIQDYDQAMRDLGYAKNEKSARMWVWFIIGGNTIIWAVVSSSGMNAFNEPWLHNVSFMIIYVGAAAAITKFSGLVMILGDRFKQLNEIARSSVQRSRWIHSYPIIDDKLIDCLHSELTVIGNNINKVYKFSLLLWCANLSFHSVCCGYFVLNWLLDGNFRWKYIECLTAWFVASVYQLFLIHYSCHYTSSEANCMSYIMLGWKRWLYTHDSKMEVETSIHLVNRQLHFSAAGCFYVNLPLLHSTAAILTTYMVILLQID |
| 931257826 | Ssp.GR6 | MSGVLTVANFKGTISTLSLCLWICLIFLKLFITNRMCAAVSNEAALTKQILQEIKPSYLKGDLREEVQQFALQLELNPLRFHAAGFFVLDNGFTKRFFGTVATYLVIFIQMSSSSNVYDEI |
| 931257812 | Ssp.GR5 | MLIAIWIMSFSLLIPPLLSVWGTLGLDRPTFSCTILKKNGSSPKKMLFVLAFIVPCVVISVSYLCIYWRVRKSRKNLEAHAGPGKRKGGGFQRREDSRVTRLMLTIFLCFLLCFMPLMLANVVDDKIRIPIVHVIASILAWASAVINPFIYAGTNKLYREAYRQVLCPASSKITMTGPKPTHSHSSKVSSPQTT |
| 931257794 | Ssp.GR4 | MADNISELITVHASLCDTVTLSNQAFGVAMLAATLTCLLHLIITPYVLITEAADKSEGLFLLTQGTWIIFHMIRLFIIVQPTYNTVTKAKRTAILVSQLLSSNPDSDAQKQLEIFSLQLLQRPLEFSACGLFSLDRPLVTSVKIFFVIY |
| 931257782 | Ssp.GR3 | MNDNEQPSMAIIYFFLYDFSFLLLFLLLGAIVWGLYRDLKDGWINSTRFKNPTAVIVTCSDVLSVILLTSVSILGSPFRWKHLQLVTDKLIQIDEKLGIVAPRRTRKFSIILTAFTLIYLLMISSLDICIWDRASRQTKKMIDKGPINYSPLYFMYVVIIIMEIQYAVSTYNIGQRFIRLNKCLENVLRTGSITDHFRKDLGLGKLNP |
| 931257780 | Ssp.GR2 | MLTTTPESPQHKRVLRMRDDWNKGVASTLDENRRTKENVDTMRAAKQVHLELVKAARNTNDIYGIQILLSMTASFVLITSLLYNAYVIIWLKLSSEEFSREMIPLSCWVFFYASKLFAINHVCAKTSAEAANTGDIICELYEPSTSKEFRAEIRDFTLQLIQNPLTFTASGFFNLDYTFIHGVIGSVTTYLVILIQFGDIQKPDAILNSTMFTNYTNTTEM |
| 931257778 | Ssp.GR1 | MENATISTIDAINTISTISTIIDGELSRFPKPLRTFAAVVAILITIVGLTGNLLTIVALCKYPKVRNVAAAFIISLCVADFVFCLLVLPFDSIRFIDASWANVRFLCVLVPFLRYGNVGVSLLSVAAITINRYIMIAHHGIYGKVYNKYWIAAMILFCWLFSYGMQLPTLLGVWGRFDYDRNLETCSIVRDAQGHTSKTFLFVMGFVIPCLVIVGCYAKIFWVVHSSESRMRKHATPNVKSPHAPGRDTREIKQRRSEWRITKMVLAIFLSFVACYLPITIVKVADSDVRYPGFHVLGYLLLYFASCVNPIIYVIMNKQYRQAYAGVIGCARIRASLTPFGSSAPAAMMNQQQDYGQGNVEQRRVISLTKL |
