# Supplementary material for: Transcriptome and Expression Patterns of Chemosensory Genes in Antennae of the Parasitoid Wasp Chouioia cunea
Source: PLoS One. 2016 Feb 3;11(2):e0148159. doi: 10.1371/journal.pone.0148159 (PMC4739689; doi:10.1371/journal.pone.0148159)
Supplement: S11 Table — (DOCX) [file pone.0148159.s016.docx]

S11 Table. The protein names and sequences of CSPs that were used in phylogentic tree analysis.

| **GI Number** | **Name** | **Protein Sequence** |
| --- | --- | --- |
| 126508768 | MmCSP1 | MKVAIIFLAIIAVALAATTKTYTSKFDDVDVDGILGSDRLLRNYVNCLLDRGPCTKEGVTLKEILPDALATSCESCTEKQKTKSEKVIRHLVNNKKELWDELAVKYDPNNEYRKKYEDQAKAKGINV |
| 55978944 | VcCSP1 | MKTTFIFLTVLAVIVVSLAQESMYTTKFDNINVQEILHNDRLLNNYVKCLLDQGRCTADAIELKKSLPDALETECSKCSPKQKEFAEEAMKFLSHNKKDIWEKLLAKYDPEKKYRSKFEDRAKEADIKI |
| 215254094 | SiCSP12 | MSVLTCVLAEELWFYSGEFDDMDVLSILEAQAEQEVDCYMKRGPCTLEQQRIADSIREAIRTNCRRCTPKQKQQIQLITDWYKSRMPQNWELIVANVDL |
| 215254092 | SiCSP10 | MNVLMCVFGEELELYPREIDDIDVLKILSDDAWRRRAEDCYFKRVPCAKEKQYLSDIFKDMLKTKCEKCTEKQKKLVKTATEWYEQNEPDTWKLILEDAHS |
| 215254090 | SiCSP9 | MCVLAEDLHSELDDLDIPKILANDAERQGVIDCILENASCTELETKAAAAIKDALKTNCQACGDKRKENMKIITDWFNQNQPDTWTLVVAKVNS |
| 215254088 | SiCSP8 | MCILAEELQPYPSEYDIYVPKILANDVVRQKAVDCYLKKGPCTEQEKLATDLFRDALKTNCKKCGEKQKEHVKILTEWFVKNQPDTWKLIIENVDS |
| 923142116 | CjCSP13 | MARLNCIIILISIASCVLAEELYSDQYDHIDVNNILNNDKLRDQYFNCYMETEPCLTAEA  KFYRDIASEALQTKCKRCTEKQKEIIDAVVDWYTQNKPDKWQKIVEKSLEDMKKKNAGQ |
| 923142114 | CjCSP12 | MARLICTIAIIGIALMCVLAEEEKYEDKYDDIDVHEVLENVKLREQYYKCFMATGPCVTA  DQKFFSKIVSEAFQTKCKLCTEKQKYMLDEISEWYTKNDPEKWNAFIAKTLEDMKKKAKE |
| 923142112 | CjCSP7 | MKVLALLLIAVACALADDKYTTKFDNIDVDAILKSDRLLKNYVNCLLDKGNCTPDGKELK  EHLPDALETECSKCSEKQRTGTEKVIRFLVNKKPETWEQLKKKYDPNGEYSRRYEDEAEK  RNIKA |
| 923142110 | CjCSP4 | MKHLVVALITALSFSVVLAEDVQYTTKYDNIDVDAVINSERLLNGYVGCLLDRTPCTPDA  AELKKNLPDALEHDCAGCSEMQKNAADKISHHLIDNKPDDWRLLEDKYDPTGAYRRRYLE  NKSHEGGRLD |
| 923142108 | CjCSP3 | MKFALVCLFAISTIVCVYGRPQDHYTDKFDNIDVDQILNNDRLLKRYVDCLLERSHVKCP  SEALELKKVLADAMATDCAKCTDRQKEIARKALDFLIINKTDMWNDLKSKYDPEEKYAKK  YEDRALKKEN |
| 923142106 | CjCSP2 | MALTIKFLILVCALFTATMAAESDNSEGQQSGRSRVSDEQLNIALSDKRYLTRQLKCALG  EAPCDPVGRRLKSLVPLVLRGSCPQCSPEETRQIKKVLSHIQRSFPKEWSRIVQQYAGVS |
| 923142104 | CjCSP1 | MDKSSLCLLALGVLAAVIAEEMYSDMFDHINPDDILPNDELRNQYYNCFMDTGPCVTEDQ  KYFKEHAAEAFATKCRKCTEVQKKNVEKIVVWYTENRPQEWQAMVQKLMDDAKKLNIPFT  R |
| 401786704 | AcCSP6 | MKIYILLFVLVTITCVIAEDYTTKYDDMDIDRILQNGRILTNYIKCMLDEGPCTNEGREL  KKILPDALSTGCNKCNEKQKHTANKVVNYLKTKRPKDWERLSAKYDSTGEYKKRYEHVLQ  FAKNN |
| 401786702 | AcCSP5 | MKIKILLFFTILALINVKAQNDISKFLMDRPYVQKQLHCILDRGHCDVIGKKIKELLPEV  LNNHCNRCTSRQVGIANTLIPFMQQNYPYEWQLILRRYKIMKYY |
| 401786700 | AcCSP4 | MKTILIALVAVCFLLGEVFSEDKYTTKYDNVDIDVVLNTERLLNGYVNCLLEQGPCTPDA  AELKKNLPDALENECSPCSEKQKEIADKVVQFLIDNKPEIWVVLEAKYDPTGAYRQHYLQ  NRVKEESY |
| 205326627 | AcCSP2 | MASAIKALLIVCALLVYTVTAETEEGQSGRSRVSDEQLNMALSDQRYLRRQLKCALGEAP  CDPVGRRLKSLAPLVLRGACPQCSPEETRQIKKVLSHIQRTYPKEWSMIVQQYAGVS |
| 205326625 | AcCSP1 | MRHNYIVILILSLLTWTYAEELYSDKYDYVNIDEILANDRLRNQYYDCFIDAGPCLTPDS  VFFKSHITEAFQTQCKKCTEIQKQNLDKLAEWFTTNEPEKWNHFVEIMIKKKDEGA |
|  | CcCSP2 | VILFVFLAFYAVAAEQLYSDQYDYVDVSKILSDDALREEYYNCYMGTSPCLTADAQYFKE  ILPEAALTKCVKCTDKQKDNFQKIATWFTKNQPEKWDAYTKKAV |
|  | CcCSP10 | MATKLVFVLAICALAAVVCAKELYSDKYDNINIDAILANDSVRNEYYNCMLDFGPCVTPD  AAYFKGLLGEIIITNCRKCTDKQRYMFKQVLKHYTLKEPQKWQELVLKVLKELPKLK |
|  | CcCSP9 | MKQLCTLVFCCVALLLAVNAAEYNSKYDNVDVDRILQNGRVLTNYIKCMLDEGNCTPDGR  ELKKTLPDALATGCIKCNEKQKATADKIINHLMKRRPADWEKLLRKYDPKGEFKKRYEAQ  GRKI |
|  | CcCSP5 | MLRRGVYHALAIATMFLAGVVVAQDTSEESTEITTELPFQKNNRTNEFYPISWTKYNYKF  IVDNERLFRKYKQCLLVDKTTGCAHDVLQLKKIIPEVLESMCAKCLPVHVERFKEIVEYV  CKKRRADYDEVRKAKDPAGLLQKKFEDKFGKVNC |
|  | CcCSP8 | MLVPSRDYYQSSCLRVVGDVQEDRSNSLARSSSRYSRRNIVIFGTIF |
|  | CcCSP4 | TSSRLQLLQARFQQKQLQEKEQKLLQLYDQQQQRAHQVAQRGSAGSNGSNHSITANANKV  KQLFAEERRQQNGVKGIDKSYPLEPLKSKKTSPINKTTNAKSTSTSTIIANINNNNNNMI  NRKSNSNVATKCVIKADANSNVSNSRSINVQQQQHERENNFASSSRQENLSRESYGNSIR  YNEAANSSESIRQVNGHTATKNGYHYEINIDEVIDNEALQRNRMLAKFQPGDIERRRRQL  SADIIDDDDDNDER*IVSLLRRIRSTRYKLALTSSALSYVT |
|  | CcCSP3 | MSRPEINLEFVVSITLLALCIARAADEDNVPLKKPIDMAELFDKNAMKDHYPIAWTEVNT  KTIIDNDRLFKKYKECLTNEHPVSCPRMVMEFKKLIPEMIDTLCAKCLPIHIEKFKEAVE  YICHRRRAEYDQVRREKDPDGAIQKKFEEQFGKVNC |
|  | CcCSP6 | MDKRMCWLALCWLLGGCLDKPINNEAIVNGYPWPEPGTYMTRWDKIDLNELFKSKRLMRH  YFNCLVNKGPCPPDGRELKRALPEALENGCAKCSKSQLESAIKIIRYLREFEPVKFEILA  NKFDPKGIYRKRYLDPTPDETNNSITDENSVDENDQKLKRLIKR |
|  | CcCSP7 | VVVLMLLLLLAIVASAQDVNILLQNKNLVSREIGCVLQRNPCDVIGKQIRGLLPEALNNG  CGRCTPQQATNAKKLIAYMKKNYPNEWVMIAQMYGRAKAVY |
|  | CcCSP1 | IVVATALLVLVAGVVRAEDKKYDSKYDNLDVEAILQNDAERNIYYACFMDTGPCPNEAAI  FFKGHAPEAVVTSCRYCTQKQLEMFEKIVSWFVDNSPQEWNALIEKTINDARKQGL |
|  | CcCSP11 | MHSYYSSRSTRRAYTLNGFYERLFLLEMFLRVCNLVYFIAGALPEALENGCAKCSKSQLE  SAIKIIRYLREFEPVKFEILANKFDPKGIYRKRYLDPTPDETNNSITDENSVDENDQKLK  RLIKRHRSTIA |
