# Supplementary material for: Transcriptome and Expression Patterns of Chemosensory Genes in Antennae of the Parasitoid Wasp Chouioia cunea
Source: PLoS One. 2016 Feb 3;11(2):e0148159. doi: 10.1371/journal.pone.0148159 (PMC4739689; doi:10.1371/journal.pone.0148159)
Supplement: S12 Table — (DOCX) [file pone.0148159.s017.docx]

S12 Table. The protein names and sequences of IRs that were used in phylogentic tree analysis.

| **GI Number** | **Name** | **Protein Sequence** |
| --- | --- | --- |
| 861722575 | MmIR25a.2 | MYNFHWRNILAFILTQCLIQTLQQNQPQNTVRSVNLFIINDEENIIANNSLNNAITNIKDKDPTVLGNVIVVQINGSDPKPALEKVCAVWDPIVRKGGPGVPDLVIDTTRSYFGTKTVNIFTSSLGIPKISGRYGQQSDIKHWGNLTADQKNYLIQIMPPVNLIPKAFRQLECEINASNAAIIIDNNYVRDPAFKSLQQNISTRHVIVQAQINNDDIDKQLNRIHDLDIVNYFVLGRENTLTNYLDVADDKNLTGRKYGIASNQQYLRKLTAQNLLPAPWLASTFYYDFVHISVEAMRSAIEGNLWPKEPRYFTCDEYNGTNTPMRNFDLLSQLRNATTNGIKPTFTRFHWGRSNGEHHAEFNRRVSMVVIGDGNSIYSDDLGLWNSEIRSPLNLTENFDTGLAHYDSIPIYRIVTVRRPPFIDFNNETNDWEGICIDMLKEMQKFMRFEYKIYESPDDKFGTMDESGNWDGMIKELMLDNADIALGTISVTAAREYVIDFTIPFYEPVGYSAITKRLLDRTRLFSFVGSLSVKVWSSAIVGFFTSSILIWVFDRWSPYSYRNDPNGRFSENYVRKFRLIDSFWFVFSSLSPQGGGIPPKNLSGQMVAGVWWLFGFITLAAYSANLAAYLTVSRLQPILKSWDDLKEQFDIQYSPVNPSDAYTYFERLKDIEERFYFVWKDISLNESLTDRERAALAIWEYPVDDEHTRLLGQMNEYGFPSSTEDGINRVLGLPPYPNDSSYALIAEATTVRYLAMTDCRFLLLEGEFSRKPYAIAVQQGSPLKELFDNAILKLLTEDKISELKKKWWDNNPKRQPWCPQRINKSDGMDIRQLGGIFIVLWVGIASAIVTLAVQYWWYRYRSRMRFKKDLENAGSLDNVEPKKFKVKPALRRISRVTNN |
| 861722572 | MmIR64a | MKSRTSVYLLFIIFLALSLSAIEGYDVLAKFTGDYFKKFYVTQIVVFGCWENHISVEFSKSIMLNTHSKLMYVNINDNLNLDKLLKVDYWSLGIVLDLDCQRSHIIFNQFSEQNLRHNESYFWLMPTSKEKIPDYFHKLPLNIANEMTLAVRKNNNLQNSGNETNNNNISYILYDVYNPSYRHGGKLNVTYMGHWSLNDRDEGTLTISLTQYKYKRRGNLYGLVLNASIVVDHPPVPDYNTYIHNPINPHLDTMHRYNYALTLQLRDYYNFTMNLKRGSTWGYLINGTFNGIIGDMMKGLVDFGATPFQYKPERLDAIEYTVQAWMARPCFIFRHPTTNELSNPFLKPFEMKVWYVIGIFAAVNWIFLYTSVKLEHKLVMKQPVCTLDTYPASEIIMITTSAICQQGLSDGPRFYAGRIVFIFLFIWGFLLYQFYSASIVGSLLAGKPRWINTLQDLADSNLEVGIEDIAYNYDFFATTTDPVAQQLYREKVAVNKKRKREPYYTIEEGLQRMQKGGFAFHVDVATAYKIIEETFDVNEICDLVEIQLFPPKHTATATARFSPFKKMVTYGLRQVVEHGMARRLRNVWMHRKPECPESHKDDPVPIMITEFSPALFLMSCGWFVSMCIVIGEKLAMKKQEIGEMKDGDADAKGYEDPEDFDRETASTNSQTSRKSG |
| 861722563 | MmIR76b | MRGIELMLIGIYALTSNNTIDYTLPGRYDWEENLEEVIKKSFLTSVQKEQEYNEIPKEIKVTSWDESLYSSISEVNGEYKGGGYALKVFDIIAAKLDFKYKIVLPEKPILGDNVTGVIGMLNQSKADVAVAFIPIIPEYLRFVQFSPILDYLEIAVLLERPLQSAIGSGLLAPFSRTVWICIFVSLALVGPIIYIVTSYRAYLWGRTKKDKYKFIDCVWFAYGAILKQGSALTPEADSNRLMFASWWIFITIITSFYTANLTAFLTLSEFTLAFTSVKDIVSQHKTWSAQQHYIVDITINRNDPEELTPLRTSYRQHRGIFYHISNKDNKTEKVASYLTNRRLFIDEIQFVENFIKNDYLNRTRQNMVEKKKCFYIKMPNPVFQQNRGFAYPSNSTISKIINRELLHLSESGIIQHIESVNRPMVTYCPLMLGSTERKLDNNDLELTYQVIATGFLFSLISFIVEVSQHYFRCRVCLCCADSCTCCKTRMKEPVSPSKRPQLPLPPPPTITTVPIKTEFSSFTKPPVIPIYDTIDNFKNSKIHYVNGRDYFVVHETTGERRLVPVRSPSALLFQYNH |
| 861722560 | MmIR75u | MFSAPTKWLILQDIRYDNDSNSSKNYNDQENKLKMFSAPTKWLILQDIRYDNDSNSSKNNDDQENRLKDKLKDFEIFPDSELFISQRFDDDKIKILSIYRPSFYVDLIIEEQATWDINNGINFFDSIPTSRRRRNLQLTPLKTCLVLTNPDTLNHLTDYKDKRIDAVTKANYIWMMHLVNQMNATVTYTWRDTWGYPDKNGTWSGMIGLLDRGEIDFGGTATFLIKQRIGVIEYLQLYTPVGSKFVFRKPPLSYVSNLFTLPFGHTVWYAIGVMSCIVLGFLYITMKWEWKEIEKSPGEQDELKSNPTISDNLLILLSAISQQGLSYEPRTISSRIVTFMLLIAALSLYASYTANIVALLQSTSSSINTLKELINSGLKLGIYDIVYNRYYFGVLDDPIRREFRERFVTNKTSIWITLEDGIEKVRQGLFAFHVDTAAGYQLMQETYEEEEKCGLQEIDYMGVLDPMLVIKKKSPYREIFRVGSLWLRETGLQQRDTPRLFTKKPVCVGHTSFISVGTTECYAAFYTITYGAAIAFGFFFLEIILYKCFGNKMTDDEESTECDADIEINTTEISRQSSESLEATLE |
| 442625954 | DmIr25a | MPRNAFGQCTLTDVIPSLWIVFINEVDNEPAAKAVEVVLTYLKKNIRYGLSVQLDSIEANKSDAKVLLEAICNKYATSIEKKQTPHLILDTTKSGIASETVKSFTQALGLPTISASYGQQGDLRQWRDLDEAKQKYLLQVMPPADIIPEAIRSIVIHMNITNAAILYDDSFVMDHKYKSLLQNIQTRHVITAIAKDGKREREEQIEKLRNLDINNFFILGTLQSIRMVLESVKPAYFERNFAWHAITQNEGEISSQRDNATIMFMKPMAYTQYRDRLGLLRTTYNLNEEPQLSSAFYFDLALRSFLTIKEMLQSGAWPKDMEYLNCDDFQGGNTPQRNLDLRDYFTKITEPTSYGTFDLVTQSTQPFNGHSFMKFEMDINVLQIRGGSSVNSKSIGKWISGLNSELIVKDEEQMKNLTADTVYRIFTVVQAPFIMRDETAPKGYKGYCIDLINEIAAIVHFDYTIQEVEDGKFGNMDENGQWNGIVKKLMDKQADIGLGSMSVMAEREIVIDFTVPYYDLVGITIMMQRPSSPSSLFKFLTVLETNVWLCILAAYFFTSFLMWIFDRWSPYSYQNNREKYKDDEEKREFNLKECLWFCMTSLTPQGGGEAPKNLSGRLVAATWWLFGFIIIASYTANLAAFLTVSRLDTPVESLDDLAKQYKILYAPLNGSSAMTYFERMSNIEQMFYEIWKDLSLNDSLTAVERSKLAVWDYPVSDKYTKMWQAMQEAKLPATLDEAVARVRNSTAATGFAFLGDATDIRYLQLTNCDLQVVGEEFSRKPYAIAVQQGSHLKDQFNNAILTLLNKRQLEKLKEKWWKNDEALAKCDKPEDQSDGISIQNIGGVFIVIFVGIGMACITLVFEYWWYRYRKNPRIIDVAEANAERSNAADHPGKLVDGVILGHSGEKFEKSKAALRPRFNQYPATFKPRF |
| 442628860 | DmIr40a | MACNELHNGYRAKFLTIVYWIAATYVLADVYSAQLTSQFARPAREPPINTLQRLQAAMIHDGYRLYVEKESSSLEMLENGTELFRQLYALMRQQVINDPQGFFIDSVEAGIKLIAEGGEDKAVLGGRETLFFNVQQYGSNNFQLSQKLYTRYSAVAVQIGCPFLGSLNNVLMQLFESGILDKMTAAEYAKQYQEVEATRIYKGSVQAKNSEAYSRTESYDSTVISPLNLRMLQGAFIALGVGSLAAGVILLLEIVFIKLDQARLWMLCSRLQWIRYDRKV |
| 221331072 | DmIr68a | MRCLWILIVAFISLAMATSIPIPIANPAPLSGYEMQLKILLQKILWVANVKRCFAVITDDLHYPIYDRIFFESVGRRVIPFFVMRTNESDDLQRPSRQVELFVKAIKSSDCELNVITILNGWQVQRFLGYIYDNRSLNMQKKFVLLHDLRLFESDMIHLWSVFIDAIFLKRQLDNKYTISTIAFPGILSGVLVMKNIANWELGKGLNGRILFADKTSNLFGTSLPVAISEHVPMVLWANATKSFQGVEVEIMNALGKALNFKPVYYKPNQTENMDWTELDGGASVAYGSGNPDGYAQNGTHIDSMLVDEVAAHSARFAIGDLHLFQVYLKLVELSAPHNFECLTFLTPESSTDNSWQTFILPFSAGMWVGVLLSLFVVGTVFYAISFLNAIINGNVSSEFFRCLRPNRNVPMDPKIYRRISFRIAISRYRSSKGDRMPRDLFDGYTNCILLTYSMLLYVALPRMPRNWPLRVLTGWYWIYCILLVATYRASFTAILANPAARVTIDTLEDLLRSHIPPSTGATENRQFFLEANDEVARKVGEKMEVFGYSDDLTSRIAKGQCAYYDNEFYLRYLRVADESGSALHIMKECVLYMPVVLAMEKNSALKPRVDASIQHLAEGGLIAKWLKDAIEHLPAEALAQQEALMNIQKFWSSFVALLIGYVISMLTLLAERWHFKHIVMKHPMYDVYNPSLYYNFKRIYPQH |
| 442627289 | DmIr31a | MNLLISMFILILAAGEGEIIPSMEESVVTNFVKSLVKTKQAIVFSCLFKDFKEISLALMRINQFVSVVNLNQSYSLTSILTRENYARTSVMVNARCSGSSELLFEASENRYFNKTYQWFLWGVDLEVQSLFPLNLNYVGPNAQITYVNETADGYAYWDIHSKGRHLKSNLEINLIATLINDTLNIARDIFHLQSIDFRGQFNGLTLRGASVIDKEDIISNEQIESILSRPTKDAGVAAFIKYHYELLGLLRERFNFTVNFRNSRGWAGRLGNTTFRLGLLGIVMRNEADIAASGAFNRINRFAEFDTIHQSWKFETAFLYRYTSDLDTHGKSGNFLSPFSDRVWLFCLLTLGAFSIIWVLFEIIDYKILRIRVNSQKLEHLNQKSSVICIKTTCIERILQTFGACCQQGLDPNPVDRSVRFLVMTLFLFSLVMYNYYTSSVVGGLLSSSDQGPSTVDEITASPLKISFEDIGYYKVLFRESQNRSITRLIEKKLSSSRSLNELPIFSHIEDAVPYLKAGGFAFHCEVVDAYPVISEYFDANEICDLREVSGLMEVEILNWILHKNSQYTEIFKTAMCNAQEKGFVERILRRRQIKKPACQSLYTVYPVSLSGVLPGFVILICKSINKFS |
| 386764010 | DmIr7c | MLHSAVHNVSLVYALVWAIDNYYGMATSTPLAVVQFPTSRESRRLHNDLIDAALGRSSGTGRIQFLLEDDRVEMTETDTDPPPPSGLTGRPIAIWFLDSLRSYFRLEMYLNQLGSPYKRNGFFLVIYTGLEDQPMESLKIMFRRLLNMYVLNVNVFLQRDGTVHLYTYYPYGPHHCQSSLPVYYTAFQDLAAPANGFGLTKPLFPRKLTNMHGCEMVVATFEHRPYVIIEDDPKTPGGRSIHGIEGLIFRSLAERMNFTIKLVEQKDKNRGEILPDGNFTGILKMMVDGEVNLTFVCFMYSKARSDLMLPSTSYTSFPIVLVVPSGGSISPMGRLTRPFRYIIWSCILVSLIFGFVLICLLKITALPGLRNLVLGRRNRLPFMGMWASLLGGLALYNPQRNFARYILVMWLLQTLILRAAYTGQLYLLLQDVEMRSPIKSLSEVLAKDYEFRILPALRTIFKDSMPTTNFHAVLSLEESLYRLRDEDDPGITVALLQPTVNQFDFRSGPNKRHLTVLPDPLMTAPLTFYMRPHSYFKRRIDRLIMAMMSSGIVARYRKMYMDRIKRVSKRRNLEPKPLSIWRLSGIFVCCAGLYLVALIVFILEILTTNHRRLRRAFNVINRYAA |
| 281366466 | DmIr76a | MENLLVESYYFSTVLSFFAQQFFADSHATCIFWHPAFDFRLETVHPMPLIIMDWHRWANRSDQDVYDYKIKEDEFEGKGIPYNDWTLRLTVAIERSHCETFIAFQEQIPEFARYFYHASIYSIWRSLRNRFMFVYTKEFEDKKDSYLSGYIFQDQPNILVITSQYLNSSTFEIKTNRFVGPRNFNKNPEPVEFYILQRFDAKGTKATWETQSAMSSKMRNLKGREVVIGIFDYKPFMLLDYEKPPLYYDRFMNTTDVTIDGTDIQLMLIFCELYNCTIQVDTSEPYDWGDIYLNASGYGLVGMILDRRNDYGVGGMYLWYEAYEYMDMTHFLGRSGVTCLVPAPNRLISWTLLLRPFQFVLWMCVMLCLLLESLALGITRRWEHSSVAAGNSWISSLRFGCISTLKLFVNQSTNYVTSSYALRTVLVASYMIDIILTTVYSGGLAAILTLPTLEEAADSRQRLFDHKLIWTGTSQAWITTIDERSADPVLLGLMEHYRVYDANLISAFSHTEQMGFVVERLQFGHLGNTELIENDALKRLKLMVDDIYFAFTVAFVPRLWPHLNAYNDFILAWHSSGFDKFWEWKIAAEYMNAHRQNRIVASEKTNLDIGPVKLGIDNFIGLILLWCFGMICSLLTFLGELWRGQG |
| 24640783 | DmIr8a | MELPLLVLLLALRFAGSEVLKITFWIEPVQRAEFDTDIAMVLKELDALRLDVKVDDTTLTLTRSEDGLDMQRFCEILSTVGASAVIDLTYSHWEEGYNLVRSLGIGYVRLERIMRPFLDMFGDFMRQKRANNVAMVFMNARDAVEAMQQMLVGYPFRTLIMDASQTDPGQHFLERIRSLRPAPTYIALFARAAAMNGIFEKVQKADLFQRPLEWHFVFLDTRDRVFKYRRQAELCTRFTLNPRAICRSMPMPDLYCGSGFTMQRAMLLNVLRSLINAAQVSPGYPLAIYQDCNATASSSEVSDPLEKDDYNWLDMVHWSNFLAYAPPLPHIQDQFQSPVPGLTFAVNISAGYYSSEHEAKTDLAAWSSVGEMRLLNETISPARRFFRIGTAESIPWSYLRREEGTGELIRDRSGLPIWEGYCIDFIIRLSQKLNFEFEIVAPEVGHMGELNELGEWDGVVGDLVRGETDFAIAALKMYSEREEVIDFLPPYYEQTGISIAIRKPVRRTSLFKFMTVLRLEVWLSIVAALVGTAIMIWFMDKYSPYSSRNNRQAYPYACREFTLRESFWFALTSFTPQGGGEAPKAISGRMLVAAYWLFVVLMLATFTANLAAFLTVERMQTPVQSLEQLARQSRINYTVVKDSDTHQYFVNMKFAEDTLYRMWKELALNASKDFKKFRIWDYPIKEQYGHILLAINSSQPVADAKEGFANVDAHENADYAFIHDSAEIKYEITRNCNLTEVGEVFAEQPYAVAVQQGSHLGDELSYAILELQKDRFFEELKAKYWNQSNLPNCPLSEDQEGITLESLGGVFIATLFGLVLAMMTLGMEVLYYKKKQNALEITQVRPVNDSSGSGGNSSTAPPTATSTTKQAWHIPVLEAEEKPAKVSPPPSFETATFRGKKLPARITLGDGKFKPRHGLYARRNLGASDSHSGYME |
| 221512773 | DmIr75a | MQLVQLANFVLDNLVQSRIGFIVLFHCWQSDESLKFAQQFMKPIHPILVYHQFVQMRGVLNWSHLELSYMGHTQPTLAIYVDIKCDQTQDLLEEASREQIYNQHYHWLLVGNQSKLEFYDLFGLFNISIDADVSYVKEQIQDNNDSVAYAVHDVYNNGKIIGGQLNVTGSHEMSCDPFVCRRTRHLSSLQKRSKYGNREQLTDVVLRVATVVTQRPLTLSDDELIRFLSQENDTHIDSLARFGFHLTLILRDLLHCKMKFIFSDSWSKSDVVGGSVGAVVDQTADLTATPSLATEGRLKYLSAIIETGFFRSVCIFRTPHNAGLRGDVFLQPFSPLVWYLFGGVLSLIGVLLWITFYMECKRMQKRWRLDYLPSLLSTFLISFGAACIQSSSLIPRSAGGRLIYFALFLISFIMYNYYTSVVVSSLLSSPVKSKIKTMRQLAESSLTVGLEPLPFTKSYLNYSRLPEIHLFIKRKIESQTQNPELWLPAEQGVLRVRDNPGYVYVFETSSGYAYVERYFTAQEICDLNEVLFRPEQLFYTHLHRNSTYKELFRLRFLRILETGVYRKQRSYWVHMKLHCVAQNFVITVGMEYVAPLLLMLICADILVVVILLVELAWKRFFTRHLTFHP |
| 221468675 | DmIr60b | MRRSLYLIIAIGLVDVHCVSLRYILNALENELQYRAILLVESASEIESCWEQKYIQGAVPILNFNANQSLYLKDALNTNILALVCLNENVESTMQALYENLEDMRDTPTILFVLSDSKVQDVFLECLRRKMLNVLAFKGLDRGFVYSFRAFPTFRVIERNVMDILQYFEQQLEDLGGHTLTTLPDNIIPRTVVYKSPDGSRQLAGYLYPFLRNYVSTINATLKVCWHLVPEDGMIQLGEVVRLSEIHDVDFPLGMHGIEHGSTSQNVPLEVSSWFLMLPMEPSLSRAQFFIMLGFEKVTPVLLLLTILLSTAHRIEMGLRPSWRCYVLGDRVLQGTLGQAFFLPRRLSVKLMLVYSLILLNGFTFSNYSITSLETWLVHPPSGHPIHSWEQMRTLNLKVLIVPSELDSMTKALGKQFTESNSDLFELSKSGNFQDKRLAMDQSYAYPVTCTLWPLLEHAQIRLPKPEFRRSREMVLIPLLIMAMPLPKNSMFHKSLNRYRALTHQSGLYEFWFKRSFNELVALRKIHYKVNGDHQIYRDFEWQDFSYVWLGFVGGTIASILVLLAEIGYHRWQLNQN |
| 24655838 | DmIr56b | MLLDTDLASGVIRSPYSFDIPHAFIFNETQFVVPKFCGPYMEIVKHFAEVYHYQLFLDSLESLPKKSVVEQDIISGKYNLSLHGVIIRPEETSDFFNATQHSYPLELMTNCVMVPLAPELPKWMYMVWPLGKYIWTCLFLGTFYVALLLRYVHWREPGNATRSYTRNVLHAMALLMFSANMNMSVKLKHASIRVIIFYTLLYIFGFILTNYHLSHMTAFDMKPVFLRPIDTWSDLIHSRLRIVIHDSLLEELRWLPVYQALLASPSRSYAYVVTQDAWLFFNRQQKVLIQPYFHLSKVCFGGLFNALPMASNASFADSLNKFILNVWQAGLWNYWEELAFRYAEQAGYAKVFLDTYPVEPLNLEFFTTAWIVLSAGIPISSLAFCLELFIHRRKQRRPQYERFECYDY |
| 221330374 | DmIr54a | MWTVITGIVLWAPVLVAGSAVDFIFRAAAEHSLSVIMIRIDYCPYNWAKDIFENQTIPVVVLSDSETFINIRMFSRPLHVACLPGHELQKDLALLENFTSSLMDFPSQKKIVYISNNFSDPTRMDYIFETCYHRRIWNIVGLLASDEHRYFYRYHLYPSFRTEYRSLESSTIFDKDFPNMHGHPLTVMPDQWLPRSVLYVDRRTGKQILAGSVGRFFHVLSWKLNATLQLSKKVTTGRFLNATALKELSESFSVDVPASLTIMERVEQLASTSYPMEVTHVCLMVPVARRIPIKDIYFILSSASNMFLAIVIVSSYGLALNLLRNMTHRDVRLVDFVLNDKALRGILGQSFNLPLSRSFSTRLIFLMLGIVGLNVSSIFGAGLDTLMAHPPRQFQARSFAGLRRTKIPLVTTEEDFPTWMKLRVPMLVVNVSEYNHLRNGRNTSNAYFASRLYWNLFSEQQKRFTRELFIYSTDDCLWSLALLSFQWPQNSLFTEPVSQLILEVNANGLYDFWVGMHYYDMTAAGLSGLEDPSLQLKEREHPTSLRIVDFQWMWQAYGTFMVIAILVFLLEVSWHRITSLFVSLVY |
| 24645175 | DmIr85a | MSIQWLKHILLLAILVNLAGTRENHIPLDLKKSSIVMVKMSQILCKARIKVLFVYFENQTSHEHTGQILKEVTKCDISNQNTPLEAVKDDGILMYMVMITTNISQPLELSLIRKKSAAKHRSHVFLLVRDADTVSDAWMRASFRQFWKIWLLNIVILYWRDGRLNAYRYNPFMDNYLIPVDNKPNEVPTLEQLFPKTIPNMQRKPLRMCIYKDDVRAIFWRQGTILGTDGLLAAYVAERLNATMMITRPHSYNNHNLSSDICFLEVAKEYVDVAMNIRFLVPDTFRKQAESTVSHTRDDLCVIVPKAKTAPTFWNIFRSFGSLVWALILVSVLVANVFCYILKSEVGRVPMQLFAGALTMPMTQIPPNHSIRLFLIFWLYFGLLICSAFKGNLTSMMVFQPYLPDINQLGALARSHYHIIIRPRHVKHIQHFLTLGHKHESRIREQMLEVSDTQMYEMMRNNDIRFAYLEKYHIARFQVNSRVHMHLGRPLFHLMNSCLVPFHAVYIVPYGSPYLGFLDSLIRSSHEFGFERYWDRIMNSAFIKSGVKVVNRRRGSGNDEPVVLKLQHFHAVFALWLVGIGMACIVLAWEHLTHNYNLAVTKRRD |
| 442631348 | DmIr67a | MLPILVPVLLLFNETSWINPILTSIYKDRHHETVLLLQHSQHGNASGLERFPWPVFSFNEQMDFYVRGKYNSEMLVLIWQTGNSDWDLDLWQALDRSLLNMRKVRVLLLRKWEKIPTADVAATAEHLLFLHVAVIGQGNRIYRLQPYAPQSWLQVDPIESPIFIKIRNYFGRYIVTLPDQFPPRSIVYRNPKTDEIQMTGYVYKFLLEFIRIYNFTFRWQRPIVQGERMNLILLRNMTLNGTINLAISLCGFETPSELGVFSDVYDMEEWYIMVPRAQEISIADVYVVMVSGNFLIVLIIFYFIFTILDTCFGPLLLKERVDWSNLMLNERMISGIMGQSFNMSARNTISSKVTNATLFLLGLVLSTLYAAHLKTLLTKRPTSQQISNFKQLRDSPVTVFFEEAERFYLKHAWDRPIRYIKDQLNFRETIEYNALRMGLNRSNAFSALTSEWMIVAKRQELFKQPIFTVQPELRVIQTSVLLSLVMQSNSIYEDHINDLIHRVQSAGIVEYWKHQTLREMITMGMISQKDPFPYVAFREFKVGDLFWIWLLWVSFLFMSFVIFLCELLVDCFISKTLIRNKRPH |
| 161076594 | DmIr21a | MSYYWVALVLFTAQAFSIEGDRSASYQEKCISRRLINHYQLNKEIFGVGMCDGNNENEFRQKRRIVPTFQGNPRPRGELLASKFHVNSYNFEQTNSLVGLVNKIAQEYLNKCPPVIYYDSFVEKSDGLILENLFKTIPITFYHGEINADYEAKNKRFTSHIDCNCKSYILFLSDPLMTRKILGPQTESRVVLVSRSTQWRLRDFLSSELSSNIVNLLVIGESLMADPMRERPYVLYTHKLYADGLGSNTPVVLTSWIKGALSRPHINLFPSKFQFGFAGHRFQISAANQPPFIFRIRTLDSSGMGQLRWDGVEFRLLTMISKRLNFSIDITETPTRSNTRGVVDTIQEQIIERTVDIGMSGIYITQERLMDSAMSVGHSPDCAAFITLASKALPKYRAIMGPFQWPVWVALICVYLGGIFPIVFTDRLTLSHLMGNWGEVENMFWYVFGMFTNAFSFTGKYSWSNTRKNSTRLLIGAYWLFTIIITSCYTGSIIAFVTLPAFPDTVDSVLDLLGLFFRVGTLNNGGWETWFQNSTHIPTSRLYKKMEFVGSVDEGIGNVTQSFFWNYAFLGSKAQLEYLVQSNFSDENISRRSALHLSEECFALFQIGFLFPRESVYKIKIDSMILLAQQSGLIAKINNEVSWVMQRSSSGRLLQASSSNSLREIIQEERQLTTADTEGMFLLMALGYFLGATALVSEIVGGITNKCRQIIKRSRKSAASSWSSASSGSMLRTNAEQLSHDKRKANRREAAEVAQKMSFGMRELNLTRATLREIYGSYGAPETDHGQLDIVHTEFPNSSAKLNNIEDEESREALESLQRLDEFMDQMDNDGNPSSHTFRIDN |
| 442624658 | DmIr60e | MVIKMISFLLVSVLLCLVGASDSESMQVQVLQDLNLALQTELNVFIDFECCATSEILHKLDSPRILLSSNSREARDLRIRGNFTESTLIIVSVMDSDLNPLVASLLPRLLDELHELHIVFLSNEEPGFPKQDLYTYCFKEGFVNVILMSGKGLYSYLPYPSIQPISLSNVSEYFDRARIIRNFQGFPVRILRSTLAPRDFEYSNEQGGLV  RAGYLFTAVKELTYRYNATIESVPIPDLPEYDVYLAVAEMLHTKKIDIVCYFKDFSLEVAYTAPLSIIREYFMAPHARPISSYLYYSKPFGWTLWAVVISTVLYGTVMLHLAARGARVEIGKCLLYSLSHILYNCHQKIRVAGWRDVAIHGILTIGGFILTNVYLATLSSILTSGLYDEEYNTLEDLARAPYPSLHDEYYRSQMKAKTFLPERLRRNSLSLNATLLKAYRDGLNQSYIYILYEDRLELILMQQYLLKTPRFNMIRQAVGFTLESYCVSNSLPYLAMTSEFMRRLQEHGISIKMKADTFRELIHQGIYTLMRDDEPPAKAFDLDYYFFAFVLWTVGLISSLLVFFAELVSGHL |
| 442622278 | DmIr41a | MFIDLSWSLVLSAIVGKYLNESTICIFWNDKFEFQLLHKSDYISFVGINIKSFDDNGGHYIIDTGLKKKELQNKHLFLDELVIKIIISIEVTHCETFVVFDKDIDRFVNAFNKASVYSIWRSLHNKFVFAHIANESPESRNHFFEDQPNILFVVRDHSSASSFDIKTNKFVGRKAENPSQMILVDRYLASEQRFQFGKSLFADKLNNLQGREVIIAGFDYPPYTVIKHNMSTNAQDMGVSGESDFKNVYIDGTETRIVLNFCEQFNCTIQIDSSAANDWGKVYPNMSGDGALGMLINRKADICIGAMYSWYEDYTYLDLSMYLVRSGITCLVPAPLRLTSWYLPLEPFKETLWAAILLCLCAEATGLVLAYKSEQALYVLPGYREGWWTCTSFGVCTTFKLFISQSGNSKAYSLTVRVLLFACFLNDLIITSIYGGGLASILTIPSMDEAADTVTRLRFHRLQWAANSEAWVSAIRASDEALVKDILYNFHIYSDDELLRLAQDQHMRIGFTVERLPFGHFAIGNYLGPQAIDQLVIMKDDIYFQYTVAFVPRLWPLLDKLNTLIYSWHSSGFDKYWEYRVVADNLNLKIQQQVQETMTGTKDIGPVPLGMSNFAGFIIVWILGSAIATLTFLLELSLTYILKQSNLK |
| 442620407 | DmIr94c | MSKVFKLLVLPLIYLSLTKGSKNPQLKFLRELINVIEEGREIRTIMVIKHSRDEYCHLDQWNPRGSPILRTNEMGSIRISGYFNDQAVILACMGENSDYGLLKSLANAMDNMRQERIILWSEREPTKMLMDYISQQADRYNFAQIIIVTMNEDVDAVPSLHQLNPYPTPRFRQITNISNIRRTSFFGCGLSFQGKTAILKESVVSNIRFKVWSPSGPIPLSELKDYEIVQFAVKYNLSLKLYDQNESKSDHFDIQLGPLFITKDFPTQMAFVSPNTACSLIVIVPCSPKWRFMDVLHKLGVLKLIGCLLIAYAVFVLIETLILWLTHRISGREVRLTSLNQLLNPRAFRGILGLPFPEFRRSSISLRQLFLVISVFGLVYSNFVSCTLSALLTKPAQNPQVRNFKELRDSGLITIMDKYTHSFIEKHIDPEFFDHVLPHYLILQKKEALRMIWNFNDSYSYVMYTTTWKSLNTVQKSFDERVFCESESLTIAWNLPRMYVLGNNSVLKWMLSRYITYMPQTGIPDSWTEQLPKVLKLLYNVTSPRRIKEGAVPLSIQHLSWIWHLLFIGESIATLVFIVEILLQKSNQHTSNMRERSSEDDDFV |
| 442620405 | DmIr94b | MSLIFNLLFILILSQAVSQETEFLQLKYLNNIVRSMIKLHKMETLVIVKHHLDNNCSLQNWNAHGMGIIRTNDQGKLIMKDTFNSRTLAIICIGQNSHITLLRNVFETFGKVQQKKIILWTQMELKEKFFQEISKKSRDLKLLNLLVLKAVTKDKLLIYRLNPFPSPHFKRIENIWTPNDTLFMDTKFNFHGMTAVVKHDYNWTIQMGNIRKFPISRIEDKEVIEFALKYNLTLQFFNDVERFDIELRKRIILKSNSTQPIDSGIPMVFSSLLIVVPCGNYLSIQDVIKVSGIEKWIFYIILVYVIFVLIEITFLGVTILISRQSRHQMIPNTLVNLCAFRAILGLPFPETRRTSLSLRQLFLAIALFGMIFSIFINCKLSSMLTNPCPRPQVNNFEELKTSGLTVVMDHDAENFIEKEIGVDFFNQYMPRKVTLTFTERAKLLFSLKGNHAFTLFSESFAIIESYQRSKGLRAHCTSEDLIVAERVPRIYILENNSILDRPLRRFIRQMQESGITNHWLKNIPSSLEKNLMQITIPYDRERVHPLSIEHLTWLWCILILGYSISMIVFFVEMSLKRRKKNLENRAPNICIC |
| 442620186 | DmIr93a | MNPGEMRPSACLLLLAGLQLSILVPTEANDFSSFLSANASLAVVVDHEYMTVHGENILAHFEKILSDVIRENLRNGGINVKYFSWNAVRLKKDFLAAITVTDCENTWNFYKNTQETSILLIAITDSDCPRLPLNRALMVPIVENGDEFPQLILDAKVQQILNWKTAVVFVDQTILEENALLVKSIVHESITNHITPISLILYEINDSLRGQQKRVALRQALSQFAPKKHEEMRQQFLVISAFHEDIIEIAETLNMFHVGNQWMIFVLDMVARDFDAGTVTINLDEGANIAFALNETDPNCQDSLNCTISEISLALVNAISKITVEEESIYGEISDEEWEAIRFTKQEKQAEILEYMKEFLKTNAKCSSCARWRVETAITWGKSQENRKFRSTPQRDAKNRNFEFINIGYWTPVLGFVCQELAFPHIEHHFRNITMDILTVHNPPWQILTKNSNGVIVEHKGIVMEIVKELSRALNFSYYLHEASAWKEEDSLSTSAGGNESDELVGSMTFRIPYRVVEMVQGNQFFIAAVAATVEDPDQKPFNYTQPISVQKYSFITRKPDEVSRIYLFTAPFTVETWFCLMGIILLTAPTLYAINRLAPLKEMRIVGLSTVKSCFWYIFGALLQQGGMYLPTADSGRLVVGFWWIVVIVLVTTYCGNLVAFLTFPKFQPGVDYLNQLEDHKDIVQYGLRNGTFFERYVQSTTREDFKHYLERAKIYGSAQEEDIEAVKRGERINIDWRINLQLIVQRHFEREKECHFALGRESFVDEQIAMIVPAQSAYLHLVNRHIKSMFRMGFIERWHQMNLPSAGKCNGKSAQRQVTNHKVNMDDMQGCFLVLLLGFTLALLIVCGEFWYRRFRASRKRRQFTN |
| 442620044 | DmIr92a | MLLQPLVMHLSQLLRIIVGQYFAEFPSILIVYNNSASTTPLQLEYLSALELVLRELSKPIRLQWINVAFLKDLNDLEDQVMGALNSSVTEGFITILSQTHHFIHARYYATRNANVRLKDKRYLFLCEDESPAELLCMDILQFYPHHLMVRPGTETAPTGPTGPHPDPRRGGGASVSTKNKDDGEGGAGNKTTSPYRDINFELWTQKFVGAVGNLDALLLDAFLPNETFANRVELYPNKLLNLQRRSLLVGSITYVPYTITNYVPAGQGDVDPIHPQWPNRSLTFDGAEANVMKTFCQVHNCHLRVEAYGADNWGGIYDNESSDGMLGDIYEQRVEMAIGCIYNWYDGITETSHTIARSSVTILGPAPAPLPSWRTNIMPFNNRAWLVLISTLVICGTFLYFMKYVSYRLRYSGTQVKFHHSRKLEKSMLDIFALFIQQPSAPLSFDRFAPRFFLATILCATITLENIYSGQLKSMLTFPFYSAPVDTIEKWAQSGWKWSAPSIIWVHTVQSSDLETEQILARNFEVHDYSYLSNVSFMPNYGFGIERLSSGSLSVGDYVSTEALENRIVLHDDLYFDYTRAVSIRGWILMPELNKHIRTCQETGLYFHWELEFIDKYMDKKKQEVLMDLANGHKVKGAPQALDVRNIAGALFVLAFGVAFAGCALVAELLIHRMDLSK |
| 442618894 | DmIr87a | MSTPEQRFWLAALLFLLSQHSEVRGFGINLMKVQTEDKGQEACILALLRKYFDSGDGLSGSVLCINRNYQLPNIEEQLLRGVNNYENYPWSLLITNSREGPSPAKFLMNEKPQCYFLIVDNLEDEDLDEVFEHWKGMVNWNPLAQFVVYLASLEETDEEMNDLMVELLLTFINKKIFNVNVIGQSEENQFYYGKTVFPYHPDNNCGNRVISVELLDACDYPSEETDSEDENDEDEGDGAQEEDDGPQEEGDGEQEEEDGPQEQEDGDQAKGDEGQENDDGGLENKVENEFRIGASDDDELENDLSSNSSEPEAIIEEFFRAKFEDKFPRDLSGCPLTASFRPWEPYIFRNSEEQPVDDYYYGLQGDEDDYNDTSPNYGESDDESYADPGEDGDGAIPDTETQSGGKLKLSGIEYEMVQTIAERLHVSIEMQGENSNLYHLFQQLIDGEIEMIVGGIDEDPSISQFVSSSIPYHQDELTWCVARAKRRHGFFNFVATFNADAGFLIGIFVVTCSLVVWLAQRVSGFQLRNLNGYFPTCLRVLGILLNQAIPAQDFPITLRQLFALSFLMGFFFSNTYQSFLISTLTTPRSSYQIHTLQEIYSNKMTVMGTSEHVRHLNKDGEIFKYIREKFQMCYNLVDCLNDAAQNEHIAVAVSRQHSFYNPRIQRDRLYCFDRRESLYVYLVTMLLPKKYHLLHQINPVIQHIIESGHMQKWARDLDMRRMIHEEITRVREDPFKALTFDQFRGAIAFSGGLLLVASCVFAFELCYVKYVYRTEKRERKTKKITKKVHNIKIQHD |
| 442617872 | DmIr84a | MIKLQVKVISWPLIILTAFLRVLQIESINTNFLELAAFEDFLRSEHLSHVLVVRGDDADGDWKIECHQKLLANYRVQFYRPEMSANFEDLMFYGSPRTAVLVLNSEHVLVRRQVFGVASEAGYFNNSLAWFILGSGRESLPVEQLIDQLLSGYRMGIDADITVALRGPDNASMLFYDVYRISRQANTPLIIEKKGLWTHSGGYQKFGNFKNTWVIRRRNFLNVTLIGSTVLTEKPPGFGDMEYLADDKQLQQLDPMQRKTYQLFQLVERMFNLSLAISLTDKWGELLDNGSWSGVMGQVTSREADFAVCPIRFVLDRQPYVQYSAVLHTQNIHFLFRHPRRSHIKNIFFEPLSNQVWWCVLALVTGSTILLLFHVRLERMLSNMENRFSFVWFTMLETYLQQGPANEIFRLFSTRLLISLSCIFSFMLMQFYGAFIVGSLLSESARSIVNLQALYDSNLAIGMENISYNFPIFTNTSNQLVRDVYVKKICKSGEHNIMSLQQGAERIIQGRFAFHTAIDRMYRLLLELQMDEAEFCDLQEVMFNLPYDSGSVMPKGSPWREHLAHALLHFRATGLLQYNDKKWMVRRPDCSLFKTSQAEVDLEHFAPALFALALAMVASALVFLLELFLHWLPDFRRRLGTMST |
| 442615429 | DmIr7b | MKYWLYILSCCSLVASTMESSSDWDLAEALAQVVANSEMGRFKTLYIYTHTNSQSTGGHLEELLDQVLMIVPNNLQARRLLLQQSMEYKPYVHAVLALVDGLPSLSAIYARIRATQDLSHTLIYMSMPTDAYGEEMQATLRFLWRLSVLNVGVVLRPPGDHILMVSYFPFSALHGCQVISANVVNRYQVGTKRWASQDYFPSKLGNFYGCLLTCATWEDMPYLVWRPDGSGSFVGIEGALLQFMAENLNFTVGLYWMNKEEVLATFDESGRIFDEIFGHHADFSLGGFHFKPSAGSEIPYSQSTYYFMSHIMLVTNLQSAYSAYEKLSFPFTPLLWRAIGLVLILACLLLMLLVRWRHHHELPRNPYYELLVLTMGGNLEDRWVPQRFPSRLVLLTWLFATLVLRSGYQSGMYQLLRQDTQRNPPQTISEVLAQHFTIQLAEVNEARILASLPELRPEQLVYLEGSELQSFPALAQQSGSSARVAILTPYEYFGYFRKVHPMSRRLHLVRERIYTQQLAFYVRRHSHLVGVLNKQIQHAHTHGFLEHWTRQYVSAVDEKDESVARIASTSYSTLDGIDGDPSLSESEEDQQVAPVRQNVLSMRELAALFWLILWANLGAVVVFVLELLLPRIKLRKILRKMKSDIKKQISKLVRK |
| 386771401 | DmIr75d | MKVQVAHWLPLIFFLLVSGTPRVAGSWRSEYSRQDPDPKTRWGNQLPDMLVAYYRHHGVHSLMLVVCHTDIADFRLWKLWQHFNLNNFYVQVSTESSLRDLQHVDALDEHKDAPPPKSFHANNSTHWETSFLLPALPYKMGILLLEFSSECALNLLRWSAASEHNYFTTNRFWLLLTEDPGDIDLLEDPEIFIPPDSELRVLHYENVGNFSCSLIDLYKVAAWKPLKRTLVGHNIRNSRHVIHALQHFGSAITYRQDLEGIVFNSAIVIAFPDLFTNIEDLSLRHIDTISKVNHRLMLELANRLNMSYNTYQTVNYGWRQPNGSFDGLMGRFQRYELDLAQLAIFMRLDRIALVDFVAETYRVRAGIMFRQPPLSAVANIFAMPFENDVWVSILMLLIITTVVLVLELFFSPHNHDMSYMDTLNFVWGAMCQQGFYVEVRNRSARIIVFTTFVAALFLFTSFSANIVALLQSPSDAIQSLSDLGQSPLEIGVQDTQYNKIYFTESTDPVTKNLYHKKIASKGENIYMRPLLGMEKMRTGLFAYQVELQAGYQIVSDTFSEPEKCGLMELEPFQLPMLAIPTRKNFPYKELIRRQLRWQREVSLVNREERKWIPQKPKCEGGVGGFVSIGITECRYALGIFGCGAAVSFVLFLFEFIFRHFKQVYRIIKGYREVQR |
| 24667182 | DmIr76b | MATGIELLVAAALCVACPPLNDSPPTNLIQMGENGTLSPVTELPMDVDASEAGFDADAPVETLETINRKKPKLREMLDWIGGKHLRIATLEDFPLSYTEVLENGTRVGHGVSFQIIDFLKKKFNFTYEVVVPQDNIIGSPSDFDRSLIEMVNSSTVDLAAAFIPSLSDQRSFVYYSTTTLDEGEWIMVMQRPRESASGSGLLAPFEFWVWILILVSLLAVGPIIYALIILRNRLTGDGQQTPYSLGHCAWFVYGALMKQGSTLSPIADSTRLLFATWWIFITILTSFYTANLTAFLTLSKFTLPYNTVNDILTKNKHFVSMRGGGVEYAIRTTNESLSMLNRMIQNNYAVFSDETNDTYNLQNYVEKNGYVFVRDRPAINIMLYRDYLYRKTVSFSDEKVHCPFAMAKEPFLKKKRTFAYPIGSNLSQLFDPELLHLVESGIVKHLSKRNLPSAEICPQDLGGTERQLRNGDLMMTYYIMLAGFATALAVFSTELMFRYVNSRQEANKWARHGIGRTPNGQSVAPSRWLRGWRRLNSGHGQLLGASTHGQNVTPPPPYQSIFNGGSHGDPLNRWRRPLANGNALGNGVLLGGDSEGGVRRLINGRDYMVFRNPNGQSQLVPVRSPSAALFQYSYTE |
| 281366391 | DmIr75c | MTSWPLYRLIVFNLLEINLSNLMVFHCWSIKEAFPLVEMLNQNGIFSQYIDVQNPDNLANVHKEYLDSDLVRLGVFLDLGCDKAELVTNQSSRARLYNQNLHWLLYDEAGNFTKLTQLFEGANLSLNADVTYVSREDEERFILHDVYNKGSHLGGKLNITVDQTLQCNRSHCQVKEYLSELHLRPRLQHRMDLSSVTFRLAALVSVLPINSSEEELLEFLNSDRDSHMDSISRIGNRLIMHTQEILGFKLHYIWCGTWSVQDAFGGAIGMLTNESAELCTTPFVPSWNRLHYLHPMTEQAQFRAVCMFRTPHNAGIKAAVFLEPFMPSVWFAFAGLLIFAGVLLWMIFHLERHWMQRCLDFIPSLLSSCLISFGAACIQGSYLMPKSAGGRLAFIAVMLTSFLMYNYYTSIVVSTLLGSPVRSNIRTIQQLADSSLDVGFDTVPFTKTYLVSSPRPDIRSLYKQKVESKRDPNSVWLSPEEGVIRVRDQPGFVYTSEASFMYHFVEKHYLPREISDLNEIILRPESAVYGMVHLNSTYRQLLTQLQVRMLETGITSKQSRFFSKTKLHTFSNSFVIQVGMEYAAPLFISLLVAYFLALLILILEICWARYAKKKFSTIIPQNQ |
| 281366389 | DmIr75b | MLQLHNLILHNLIHMAKLSHVLILHCSLSHLALLAQSKNIFTQFQPLHSDIQLNDDFLNHNILKLGVFLDINCDKSGTVLDMASAKRFFSHRYHWLIYDRSMNFSVLESHFKEAQIFVDADVTYVTHDPFSKNFLLYDVYNKGRQLGGELNITADREIFCNKTNCRVERYLSELYTRSALQHRKSFTGLTMRATAVVTALPLNVSIKEIFDFMNSKYRIQLDTYARLGYQARQPLRDMLDCKFKYIFRDRWSDGNATGGMIGDLILDKADLAIAPFIYSFDRALFLQPITKFSVFREICMFRNPRSVSAGLSATEFLQPFSGGVWLTFALLLLLAGCLLWVTFILERRKQWKPSLLTSCLLSFGAGCIQGAWLTPRSMGGRMAFFALMVTSYLMYNYYTSIVVSKLLGQPIKSNIRTLQQLADSNLDVGIEPTVYTRIYVETSEEPDVRDLYRKKVLGSKRSPDKIWIPTEAGVLSVRDQEGFVYITGVATGYEFVRKHFLAHQICELNEIPLRDASHTHTVLAKRSPYAELIKLSELRMLETGVHFKHERSWMETKLHCYQHNHTVAVGLEYAAPLFIILLGAIILCMGILGLEVIWHRHCTLH |
| 281364203 | DmIr60d | MRLAIYVAFLSSIGNRSGFLSSLLMSLGKELHYKTILLVGGSSTCWSLEPFETGVPILNLRGENNAYPQDTFNSQMLALACLQTESEDAVKLLYRSLKDMRDTPTLLFASSEEHIHDTLFLGCFRENMLNVLALTASSKEFIYSYQAFPTFRVIKRKLVEIHRYFEPQLKDLGGHIVSALPGNIMPRTMCYRNAEGERQLAGYLNTFIRNYVESINGTLRISWGLVPEDDMRHLTISRLSKIQHVDFPLGIIPLYNKTDKQHVYMEISSWFLMLPMETSVPRAHLFVKLGLERLLPIIVVVGAVLGNAHRIEVGLGPSWRCYYLADKVLRGALAQPIVLPRRLSPKLMLIYSLLLLSGFFLSNYYMASLTTWLVHPPASDRILEWDQLRYLHLKVLTIPEEFKYMSLILGTDFMTAYGSIFQLTNSTDFQRRRISMDPSYAYPVTTSLWPFLELSQVRLRRPLFRRSYDMVLQPFQVMSLPLPRNSIFHKSLLRYAALTRETGLYYYWFRRSYYELVALGKISYKEEEGNPYCDLKWNDFRIVWLAFLGGTIISCLALLLEVAHYRWHLGNSSL |
| 221458669 | DmIr94h | MLSNISFSSAPELVDLYGLVLKFLVSSETTLFYFNPTGQKCSWETLPRTILSNHPQIIWFREETYPGLYKRHSSNLFVMACLSSTSYDGQLQLLAESLTRYRSVRVLIEVQDKEGSFLASQILLLCQQHSMLNVVLYFSRWTRTLNVFSYLAFPYFKLLKQRLSGSLRPKIFINQLKDLQGYKIRVQPDLSPPNSFSYRDRHGECQVGGFLWRIVENFSKSLKGDTQVLYPTWAKAKVSAAEYMIQFTRNGSSDIGVTTTMITFKHEERYRDYSYPMYDISWCTMLPVEKPLSVEILFSHVLSPGSALLLILAFILFFLIVPQLIKCLGITFRGRLIGMASRIFALVMLCSSSAQLLSLLMSPPLHTRIKSFDDLLTSGLKIFGIRSELYFLDGGFRAKYASAFHLTENPNELYDNRNYFNTSWAYTITSVKWNVIEAQQRHFAHPVFRYSTDLCFSSETPWGLLIAPESFYREPLQHFTLKINQAGLITQWMTQSFHEMVRAGRMTIKDYSRTNLMKPLRIQDLRKCWVIFAVGLGTSTVVFTIELLLIYTNVFLNSL |
| 221458665 | DmIr94g | MSTAVNSVHSKLVSLISRGQELTSIFFYAPAKEKCHLEDTISSATWGLPLVIWRTDRTVILNGFIGEGLLVLACLPGFHWRALLGSLARSLKYLRQARILIELMQDRDEFLVSEVLQFCLSQDMINVNAIFDDFPETENLSSFEAYPSFEVVNQTFTPDTQVSDLYPNKMLNLRGGVIRTMPDYSEPNTILYQDKEGNKEILGYLWDLLEAYAHKHNAQLQVVNKYADDRPLNFIELLDAAQSGIIDVGASIQPMSMGSLSRMHEMSYPVNQASWCTMLPVERQLHVSELLTRVIPYPTLALLLLLWIFYEVLRGRWRRHSRLQSIGWLVLATLVSSNYVGKLLNLFTDPPSLPPVNSLAALMESPVRIISIRSEYSAIEFTQRTKYSAAFHLALHASILIGLRNAFNTSYGYTITSEKWKIYEEQQKRSSKPVFRYSKDLCFYEMIPFGLVIPENSPHRAPLHSYTLLLRQAGLHDFWVNRGFSYMVKAGKINFTAVGERYEAKTLTITDLRNVFIIYVSVLLISLILFTCELFVSWVNYWLGF |
|  | CcIR25a | ILLWLLVVGEGGPVTAQYNDVINRPVNVFVINDAGNDVANKSVTNSLRTLKDKNPDKLGQVYVVQVNISDSKETLNAICDLWKSAITDNQDNIPDFVLDTTTYGIGAETVNKFTAFLGIPTLSAQYGQEGDMLGWREISEDQKQYLVQVMNPADLMPEVVREQCSHFNISNAAILFDESFVMDHKYKSLLLNVPTRHVIVPTKPAGTALREQISRLRDLDIVNFFVLGTEATISAALQAASNLGFTDHRYGWFGINLNEDFTIQCPDCKRARILVFKPEMSTSQQQLNELTSKGALPKPWIQSAFYYDLTKIGILAMKAAIDAGEWKLDRRRFMLCDDYNENVTTPLRNLDFRKRLKLVTGGGSVFMPTYAGFGWGNKNGESQAKFEANGVAIQIRDSRVTSEEIVESWQAGVDVPLTIKNPGIAANQTAVTSYRVVTVIKPPFIMHDNKTGEWSGYCIDLLNKIREHVKFEYTIEEVEDKEYGNMDEQGNWNGMVKVLKDKKADIALGALAVMAERENV |
|  | CcIR75q2 | MRLFPGIWLWLISMRTSGFIVDGCESDQAKELVELLIELKTRGLSTFSRVTMFGCSDDKSQVARRLSRALVANSMHELDSNQFSWNLQKSVASTPDHHLLIVLEVDCPSSERLLLEAKNATMFVSPYKWFLISRGCSSVDCVLSKLSELRRNVLPDSELLIWLKQSQRLVSVYKVSAEQGRDWQIEERARSIACDNATFNLLLDDDSAMITSRRRSDLRRTHLKSSLVITDYDSLQHLTDYRNREIDTSSKCNYPWAKFLASMLNATISFKLAPTWGYPRANGTWDGMTGMMQRGEIEFGASHSFVTGERLKVVHYLAGLTTPSHSRFVFRRPALSSVANLFALPFRGSVWIGVLALALLFGFLLYPAMVLEWRRHRDGTIEPRVGDETIVVVGALAQQGFWYEARAASTRVLVLAGLLGFLSLHAAYAANIVALLQSTSSSIKSLKDLLASPLDLGVHDTVFNRYYFKSLGSLEPKIRGAISEKSEWLGLEEGVRRLRRGHFALHAVLGWAYKVVHETFEEDEKCDFEEIDFLNVFEPHMVVAKFSPYKELLRVKALRIRETGMRTREINRLYSTRPRCDAAASSRRFISVGYAECKGAFYALGYGLVVALLLFAAEIAHRRLRLGIRSNRLKR |
|  | CcIR93a | MMLSLLLPFLLRLGWTNGYNDFPSLMTANATMAVIIEKGFFKGQDEYRNALTDITDIVIGIIKKNMKQSGIDIFVFGDTNINLGRDYTILLSVATCQTTWNLFKRAQKEKLVHLAITGPDCPRLPESDGISLPFIDPGEELAQIFLDLRMSSALAWAKVNFLHDDTFDRDTISRVVKALSVELPNKKLLLSTRAIFSVQYDKSDSVMKQRIHNMLADFHVDQLGSCFMVIITIDMVSSMMEVAKSLKMVHPRSQWLYVISDSASRDTNVTMFLDLLTEGENVAFIYNTTSLDLECKLGLTCHIKEFVRALAKSLENSLKTELELYDSVTEEEFEVVRLSKAERKDEIIKSINRELSELRAGTTNTCGECISWKIASAITWGASFSINNEAKQQEQKQKQEHHKNKTELIKKSVGELVDSGTWSPAPGIKMNEMLFPHIQHGFRGKSLPVSTFHNPPWQILSFSNTDKPEFRGLVFDILNYLSLKLNFTYMVQLPSGYDMLSKFQPNNTTVKGKKINKFDVNEAAISVARKVPAEVIELVGDGRVFLAAVATTVSENTKNVNFTYMIAQQAYALLSAKPKPLSRALLFMAPFTSETWACLSSALLLIGPFLYVMVKLSPKPIELNEVVGLSTTWQCSWYVYGALLQQGGMSLPKADSARLVVGTWWIVVMVVVATYSGNLIAFLTFPRMDDPIDTVDNLIARQNQFTWAYPNGSAFENYLIAAAQDTEKYKILLDGASQEDPSEPKRVLAKVKDSNHVLIDWRTSEAFLMRLDLMDTGVCNFHVGTEDFLHENMRLIIADNSPYLELVNAAIIRMHESGLINKWSLDILPLKDKCFVTKGNQEVTNHKVDMGDMQGIFFVLAIGFT |
|  | CcIR8a | MSRARRINVQLVFLLSFGLALVASQAPVTLLLVIEQPDAEIMGNLNDVVSEAESQFGANLIKIDVKLVQVDREFVDENYDRVCAHLYNGITMILDMTWTGWDKLRDLARDFNIIYKRADTTISSYVQAVDQTMMYKNSTDAALIFENEKELNQTLYYLIGNSIIRLVVIESLTAREVDRISNMRPLPSYYVIYARTKQMEELFKTALEGGLVKRDMVWYLAFTDNNHADFSYFRDSNNPNVSVNVFTMKEEVCCHLMYTVAPCNCPADFVIFHHYFRRLVQLIVETMSECQAANRLQEPQSGQCQNKNATENSVNATLSEFDKRLLAKIEKNDTFEYVTARTLVTYKAAADLKLLKKGQLETIGTWSRDTGILPLPNKTIQAARRYFRVGTVDAIPWTYKQLDPDTNEPIKRPDGTYVYEGYCIDLIDRLAEMMDFDFDLVIPQDGEFGQKVNGFWNGLVGDLSKGQTDIAVAALTMTSEREEVIDFVAPYFEQSGILIVMRKPVRKASLFKFMTVLRLEVWLSIVGALTLTGVMIWVLDKYSPYSARNNKHMYPYPCREFTLKESFWFALTSFTPQGGGEAPKALSSRTLVAAYWLFVVLMLATFTANLAAFLTVERMQSPVQSLEQLARQSRINYSVLENSTIHQYFKNMKMAEERLYQVWKEITLNSTSDQVEFRVWDYPIKEQYGHILQAIAQVGPVKTIEEGFQKVEASENAEFAFIHDSSEIKYKVTQDCNLTEVGEVFAEQPYAIAVQQGSHLQEEISRKILDLQKDRYFEQLSSKYWNQSLKGSCSNADDNEGITLESLGGVFIATLFGLALAMITLAGEVIYYRRRNTRQDDSLQQSKNGTNNSGSISGSIQDVKQTKELDIQKLAARLQLKPAPPVAFEQKPSSLNTTKPRVSHISVYPRPFPFKD |
|  | CcIR75q.2 | MHRYIRQQCVYVSNNINMKRCTNSLWKCLLFSLIRLEILRVGQVSCRHIDESISNLVVEVSTVSFFPSAAISSLRCRHSDDNVIFSRILSENRMLHNHLSFDDFQKDDFPLHKIVIFLDFKCHGAREFLLKANSSEMFSAPYKWIIFQDLEHSSPDNCTDGCAFKDFYSYAMYPDSSVVILQKLSKERVQIVSIYRPSPVRDMIVENLGYWSSTNGTKWHNLNIASQRRKNLQKTPLKSSIVVTNPDTLNHLTDYHDKHVDTITKCNFVWLHQLIDAMNATVTYSIVNTWGYRDKNGSWTGMTGQLSRKEIDIGGTSMFIIGDRWNDVHFIPLSTPTRQAFIFRQPPLSFVSNLFTLPFRPSVWIAIGILLMIIFAMLLLATKWEWRKVYADREFSENEPKPNLSDQLLLILGVCAQQGFGRSPYTVPSRIVLLMLLLAVLNLYASYSANIVALLQSTTTSITSLKDLLESPIKCGANDIVYNRHYFKLEKDPVKRAIIDKKIEPKGSKANWMTADEGISRVRQGFFAFLIETGPGYRILQETFEEDEKCGFREMYFIDHFDPMFAIVKRSPYKELIRVNSLKIWESGLKSKEMSRLYTKRPPCNGRNKFVSVGLNECYFAFYIIGYGVLFAILAFLVEILSKKSGSLRKRQPVESTARTSFAQRNLQQNSARESPFSAS |
|  | CcIR76b | FPSHITVTTYSDMPYSRYRKLDNGTFVGEGFAFELLALLMKKFKFTYTIIPPAKDIIGDESSGMIQQLYN |
|  | CcIR75p | SENEPKPNLSDQLLLILGVCAQQGFGRSPYTVPSRIVLLMLLLAVLNLYASYSANIVALLQSTTTSITSLKDLLESPIKCGANDIVYNRHYFKV |
